# Synovial cellular and molecular signatures stratify clinical response to csDMARD therapy and predict radiographic progression in early rheumatoid arthritis patients

**DOI:** 10.1136/annrheumdis-2018-214539

**Published:** 2019-03-16

**Authors:** Frances Humby, Myles Lewis, Nandhini Ramamoorthi, Jason A Hackney, Michael R Barnes, Michele Bombardieri, A. Francesca Setiadi, Stephen Kelly, Fabiola Bene, Maria DiCicco, Sudeh Riahi, Vidalba Rocher, Nora Ng, Ilias Lazarou, Rebecca Hands, Désirée van der Heijde, Robert B M Landewé, Annette van der Helm-van Mil, Alberto Cauli, Iain McInnes, Christopher Dominic Buckley, Ernest H Choy, Peter C Taylor, Michael J Townsend, Costantino Pitzalis

**Affiliations:** 1 Centre for Experimental Medicine & Rheumatology, William Harvey Research Institute, Barts & The London School of Medicine & Dentistry, Queen Mary University of London, London, UK; 2 Biomarker Discovery OMNI, Genentech Inc, South San Francisco, California, USA; 3 Bioinformatics and Computational Biology, Genentech Inc, South San Francisco, California, USA; 4 Centre for Translational Bioinformatics, William Harvey Research Institute, London, UK; 5 Department of Rheumatology, Leiden University Medical Center, Leiden, Netherlands; 6 Amsterdam Rheumatology Center, AMC, Amsterdam, Netherlands; 7 Department of Rheumatology, Zuyderland MC, Heerlen, Netherlands; 8 Universita degli Studi di Cagliari, Cagliari, Italy; 9 Institute of Infection, Immunity and Inflammation, University of Glasgow, Glasgow, UK; 10 University of Birmingham, Birmingham, UK; 11 Institute of Infection and Immunity, Cardiff University School of Medicine, Cardiff, UK; 12 Nuffield Department of Orthopaedics, Rheumatology and Musculoskeletal Sciences, Kennedy Institute of Rheumatology, Oxford, UK

**Keywords:** early rheumatoid arthritis, synovitis, dmards (synthetic), inflammation

## Abstract

**Objectives:**

To unravel the hierarchy of cellular/molecular pathways in the disease tissue of early, treatment-naïve rheumatoid arthritis (RA) patients and determine their relationship with clinical phenotypes and treatment response/outcomes longitudinally.

**Methods:**

144 consecutive treatment-naïve early RA patients (<12 months symptoms duration) underwent ultrasound-guided synovial biopsy before and 6 months after disease-modifying antirheumatic drug (DMARD) initiation. Synovial biopsies were analysed for cellular (immunohistology) and molecular (NanoString) characteristics and results compared with clinical and imaging outcomes. Differential gene expression analysis and logistic regression were applied to define variables correlating with treatment response and predicting radiographic progression.

**Results:**

Cellular and molecular analyses of synovial tissue demonstrated for the first time in early RA the presence of three pathology groups: (1) *lympho-myeloid* dominated by the presence of B cells in addition to myeloid cells; (2) *d* *iffuse-myeloid* with myeloid lineage predominance but poor in B cells nd (3) *pauci-immune* characterised by scanty immune cells and prevalent stromal cells. Longitudinal correlation of molecular signatures demonstrated that elevation of myeloid- and lymphoid-associated gene expression strongly correlated with disease activity, acute phase reactants and DMARD response at 6 months. Furthermore, elevation of synovial lymphoid-associated genes correlated with autoantibody positivity and elevation of osteoclast-targeting genes predicting radiographic joint damage progression at 12 months. Patients with predominant pauci-immune pathology showed less severe disease activity and radiographic progression.

**Conclusions:**

We demonstrate at disease presentation, prior to pathology modulation by therapy, the presence of specific cellular/molecular synovial signatures that delineate disease severity/progression and therapeutic response and may pave the way to more precise definition of RA taxonomy, therapeutic targeting and improved outcomes.

**Key messages**

**What is already known about this subject?**

- Disease tissue heterogeneity is postulated to play a major role in incomplete drug response related to diverse target expression levels and drug pharmacology. In established/long-standing RA distinct histological and molecular subtypes have been described.
- However, their relationship with clinical phenotypes (e.g. disease activity/progression) and treatment response remains controversial.

**What does this study add?**

- This study provides: first-time evidence in early, treatment-naive RA patients of the existence in the synovium of three specific pathology subgroups (Lympho-myeloid, Diffuse-myeloid and Pauci-immune-fibroid), prior to the potential modification of disease pathology by immune-modulatory therapies.
- In addition, it demonstrates that specific histopathotypes and associated transcriptional endotypes define distinct RA subtypes that are linked to diverse clinical phenotypes, disease activity/severity and treatment response and that gene expression signatures associated with cellular infiltration in synovium strongly correlate with imaging modalities including ultrasonographic measures of synovial thickness and power Doppler and erosive load on x-ray.
- Moreover, longitudinal analysis demonstrated that specific synovial signatures are associated with response to DMARD therapy, clinical outcome at 6 months and radiographic joint damage at 12 months.

**Key messages**

**How might this impact on clinical practice?**

- This study demonstrates that the cellular and molecular signatures define pathobiological endotypes early in the disease process, prior to treatment modification, that have a significant impact on disease prognosis and treatment outcome.
- This offers the potential for a more accurate patient stratification of this severe disabling disease where early disease modification is crucial to life course outcome and quality of life.

## Introduction

Better understanding of rheumatoid arthritis (RA) disease pathogenesis has led to the development of highly effective therapeutics inhibiting structural damage and improving prognosis^1^; however, a sizeable proportion of patients (~40%) do not respond to these therapies and the mechanisms for response/nonresponse remain unknown. Additionally, as currently it is not possible to predict which patients would benefit from individual therapeutic modalities, this leaves a huge unmet clinical need.

Disease tissue heterogeneity is postulated to play a major role in incomplete drug response related to diverse target expression levels and drug pharmacology. In established/long-standing RA, distinct histological and molecular subtypes have been described.^2 3^ However, their relationship with clinical phenotypes (eg, disease activity/progression) and treatment response remains controversial. The reasons for such discrepancies (reviewed^3^) include multiple confounding factors ranging from (i) small sample size (mostly 20–40 patient cohorts), (ii) reliance on cross-sectional rather than prospective data and (iii) inclusion of patients at different disease stages/severity and treated with multiple therapies potentially influencing synovial pathobiology.^3^

In addition, many studies have been performed in established/late-stage disease and with a sampling bias due to prevalent representation of large joints (arthroscopy/joint replacement) while only a handful of studies have included small joints.^3^ Furthermore, though molecular characterisation of synovial tissue has been recently published in established/long-standing RA,^4 5^ here we report the first systematic cellular (immunohistology) and molecular (NanoString) characterisation in early disease, treatment-naïve patients.

We demonstrate at disease presentation, prior to potential modification by therapy, the presence of three distinct pathology groups: (*1) lympho-myeloid* dominated by lymphoid lineage infiltration (T cells, B cells, plasma cells) in addition to myeloid cells; (*2) diffuse-myeloid* with myeloid lineage predominance but poor in B cells/plasma cells and (*3*) *Pauci-immune* characterised by scanty immune cells and prevalent stromal cells. Longitudinal correlation of molecular signatures demonstrated that elevation of myeloid and lymphoid-associated gene expression strongly correlated with disease activity, acute phase reactants and conventional synthetics disease-modifying antirheumatic drugs (DMARDs) response at 6 months. Furthermore, elevation of synovial lymphoid-associated genes correlated with autoantibody positivity and the elevation of osteoclast-targeting genes and predicted radiographic joint damage progression at 12 months. Patients with predominant pauci-immune pathology showed less severe disease activity and radiographic progression but, importantly, lower therapeutic response.

## Methods

### Pathobiology of Early Arthritis Cohort

In all, 144 RA patients fulfilling 2010 American College of Rheumatology/European League Against Rheumatism (EULAR) Classification Criteria, with clinically defined synovitis and symptom duration less than 12 months, were enrolled as part of the ‘Pathobiology of Early Arthritis Cohort’ (PEAC, http://www.peac-mrc.mds.qmul.ac.uk) at three UK Academic Centres: Queen Mary University of London/Barts Health NHS trust, University of Glasgow and University of Birmingham. All patients were naïve to steroid and DMARD therapy.

**Figure 1:**
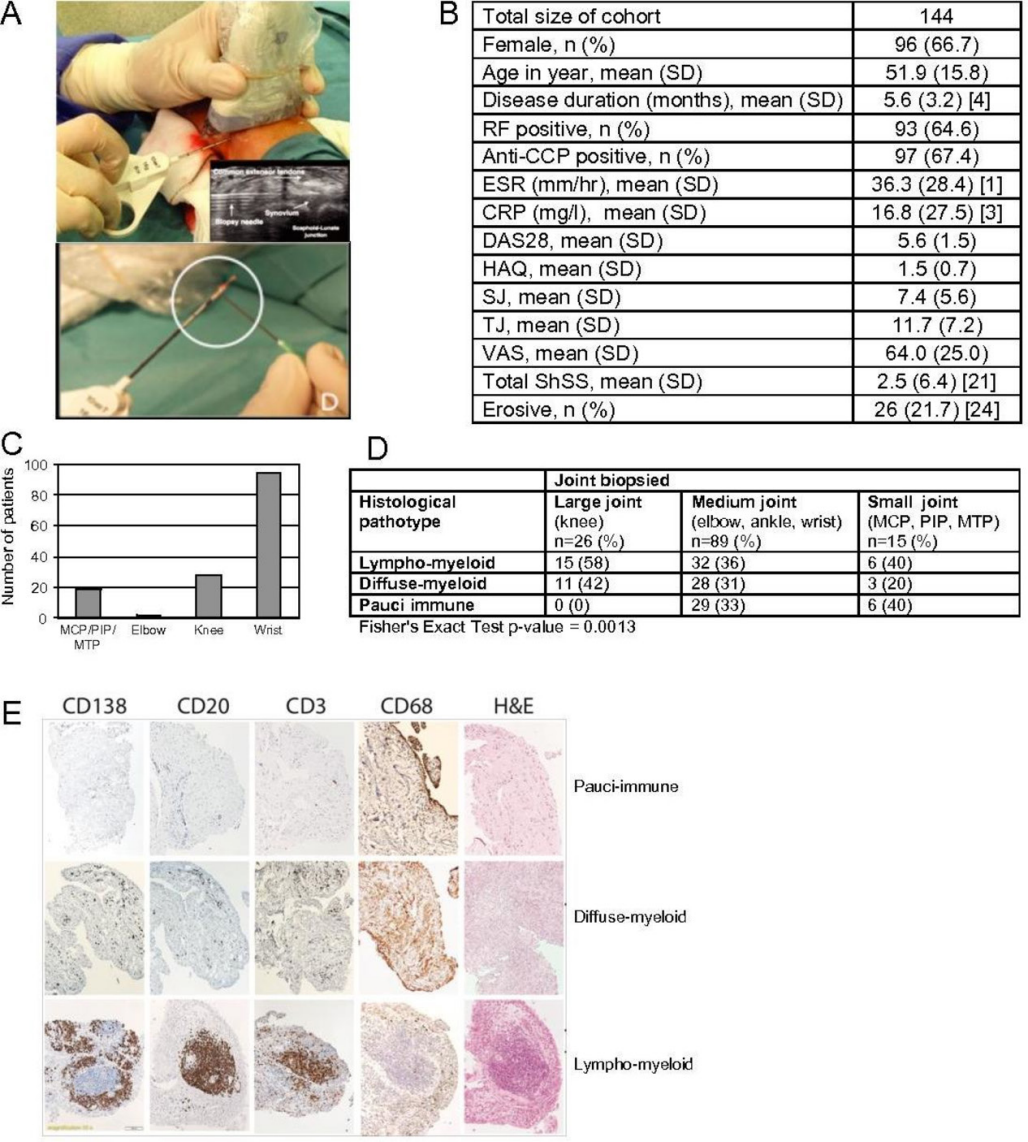
(A) Representative image of an US-guided wrist biopsy. Inset: greyscale transverse US image of wrist joint demonstrating biopsy needle entering joint space under extensor tendon complex. Synovial tissue fragment (encircled) in biopsy needle. (B) Patient characteristics of cohort. (C) Number and type of joints biopsied. (D) Synovial pathotype according to joint biopsied. (E) H&E staining and immunohistochemistry of synovial biopsies for CD20^+^ B cells, CD3^+^ T cells, CD68^+^ macrophages in synovial lining/sublining layers and CD138^+^ plasma cells from treatment-naïve individuals with early rheumatoid arthritis. Synovial biopsies were categorised as lympho-myeloid, diffuse myeloid or pauci-immune fibroid. CCP, cyclic citrullinated peptide; CRP, C-reactive protein; DAS28, disease activity score-28; ESR, erythrocyte sedimentation rate; MTP, metatarsophalangeal; MCP, metacarpophalangeal; PIP, proximal interphalangeal; RF, rheumatoid factor; SHSS, van der Heijde modified Sharp score; SJ, swollen joints; TJ, tender joints; US, ultrasound; VAS, visual analogue scale.

Patients underwent a ultrasound (US)-guided synovial biopsy (figure 1A) procedure we pioneered^6^ of a clinically active joint selected according to a previously defined algorithm to ensure maximal synovial tissue retrieval^6^; a minimum of 12 synovial biopsies were stored for subsequent analysis at the William Harvey research Institute (six for histological analysis and six for RNA extraction), and patients then commenced on standard DMARD therapy and/or low-dose corticosteroid. A treat-to-target approach to therapy escalation was followed aiming for low disease activity score-28 (DAS28) <3.2. Patients failing DMARD therapy were commenced on biological therapy according to the UK National Institute for Clinical Excellence prescribing algorithm for RA patients if they continued to have a DAS28 >5.1 at 6 months.

Ultrasonographic images were collected at the time of biopsy for both the individual biopsied joint and the global joint score: first to fifth metacarpophalangeal (MCP) joints and midline, radial and ulnar views of both wrist joints. Images subsequently underwent semiquantitative assessment by a single blinded (to clinical/histological data) assessor for both synovial thickening (ST) and power Doppler (PD) activity according to standard EULAR-OMERACT (Outcome Measures in Rheumatology) US synovitis scores (grade 0–3).^7^ The mean global ST and PD scores including the maximal score for the wrist joint were then determined.

Plain radiographs of the hands and feet performed at baseline and 12-month follow-up were scored in time sequential order according to the van der Heijde modified Sharp score (SHSS) by a single reader blinded to all clinical/histological data. The study received local ethical approval (REC-05/Q0703/198) and all participants gave written informed consent.

### Histology

Paraffin-embedded H&E stained sections were graded as suitable for further histopathological assessment if intact lining layer was identified. Following immunohistochemical staining for B cells (CD20), T cells (CD3), macrophages (CD68) and plasma cells (CD138) as previously reported,^8^ the degree of immune cell infiltration was determined semiquantitatively (0–4). Biopsies were then stratified into one of the three synovial groups according to the following criteria: (i) lympho-myeloid presence of grades 2–3—CD20+ aggregates, (CD20 ≥2) and/or CD138 ≥2; (ii) diffuse-myeloid—CD68 SL ≥2, CD20 ≤1 and/or CD3 ≥1, CD138 ≤2 and (iii) pauci-immune-fibroid—CD68 SL <2 and CD3, CD20, CD138 <1.

### NanoString gene expression analysis

*NanoString Panels Construction*. Pathotype-specific NanoString panels were developed based on a previous microarray study^2^ (GEO accession number GSE48780). Differential expression analysis was performed using the limma package.^9^ We performed all pairwise comparisons between samples in the pauci-immune, diffuse-myeloid and lympho-myeloid groups. Genes were selected for a pathotype if they showed differential expression with each of the other two pathotypes at a Benjamini-Hochberg adjusted p value <0.01 (online supplementary figure 1A–C and online supplementary tables 1–3). These genes were validated in the baseline RNA-seq data (deposited in ArrayExpress accession code E-MTAB-6141) from the PEAC cohort. For each set of pathotype-specific genes, we identified the 50 genes that best correlated with the first principal component of the z-score transformed expression data for that gene set and included an additional set of 87 genes implicated in RA pathobiology (online supplementary table 3). We confirmed that expression levels determined by NanoString were concordant with those measured by RNA sequencing, in samples where both measurements were available (online supplementary figure 2).

10.1136/annrheumdis-2018-214539.supp2 Supplementary data

10.1136/annrheumdis-2018-214539.supp1 Supplementary data

*NanoString Analysis*. Total RNA was extracted from synovial tissue using TRIzol Reagent (ThermoFisher Scientific, Life Technologies, Invitrogen Division, UK) as previously described.^6^ A custom CodeSet of capture and reporter probes was designed to target regions of 100 nucleotides of 242 genes. Hybridisation of 50–100 ng of synovial RNA was carried out according to the manufacturer’s instructions. Raw expression data were obtained using the NanoString nCounter MAX analysis system (NanoString Technologies, Seattle, WA, USA) according to the manufacturer’s instructions. Analyses of NanoString expression data were performed using R (V.3.3.2). For differential expression analyses, we used the limma Bioconductor package^9^ using default settings. We used the Benjamini-Hochberg method^10^ to adjust for multiple testing, and considered genes to be differentially expressed if they had an adjusted p value <0.01. Eigengene scores were calculated as previously described,^11^ with statistical adjustment for biopsy joint position. The osteoclast gene set utilised was described in the Harmonizome database (http://amp.pharm.mssm.edu/Harmonizome/).

### Serum biomarker assessments

Serum samples from 111 patients at baseline were assessed for levels of soluble intercellular adhesion molecule 1 (sICAM1), C-X-C motif-chemokine-13 (CXCL13), interleukin-8 (IL-8) and matrix metalloproteinase-3 (MMP-3) using customised electrochemiluminescence assays incorporating sample diluent blocking reagents to minimise interference from heterophilic antibodies.

### Statistical methods

Statistical analyses of histological and clinical parameters and predictive modelling of progression were run using R.3.0.2. P values relating discrete variables to each other were calculated with the use of Fisher’s exact test. P values for correlation were derived using Spearman’s correlation. To account for multiple testing, we used the method of Benjamini and Hochberg to adjust p values. For correlations between clinical characteristics and eigengene values with adjustment for biopsy joint position, all p values from all comparisons were adjusted together. Likewise, for correlations between serum biomarkers and clinical characteristics and histological parameters, p values were adjusted altogether. P values for comparison of means between three groups were calculated using one-way analysis of variance with Bonferroni post hoc test. All p values reported are two sided.

Selection of gene predictors: Backward stepwise selection method using logistic regression was performed using glm function in R, with 16 baseline clinical covariates considered as candidates in the regression model. A subset of predictors was selected by imposing a L1 (least absolute shrinkage and selection operator, LASSO) penalty on the regression parameters (46 genes and 8 clinical covariates) of a sparse logistic regression using R package glmnet. The penalty parameter (lambda) was optimised by 10-fold cross-validation. Lambda tuned to the smallest mean cross-validated error was retained as final penalty parameter in the model.

Predictive performance evaluation: The predictive performance of the models with and without the genes (respectively, LASSO and backward stepwise models) was assessed by computing the area under the receiver operating characteristic curve (AUC). Both apparent and internally validated AUC were assessed. To compensate for overfitting inherent to the apparent AUC, internal validation was performed to correct the measure of predictive performance for optimism using bootstrap (repeated 500 times) with the R-package boot V.1.3–18, as a valid method to generate unbiased optimism-adjusted estimates of the C statistic (AUC) with small absolute errors.

## Results

### Patient cohort

Patient characteristics are summarised in figure 1B. Briefly, most patients had high disease activity (mean DAS28 5.6±SD 1.5), mean disease duration 5.6 months (SD 3.2), approximately 65% were positive for rheumatoid factor (RF) and/or anti-citrullinated peptide antibodies (ACPA), and 20% had at least one radiographic erosion. The joint biopsied most frequently was the wrist (~65%), with good additional representation from MCP/proximal interphalangeal/metatarsophalangeal joints (~15%) and knee, elbow together ~20% (figure 1C).

### Identification of synovial pathotypes by histopathology

In total, 129 of the 144 recruited patients (89.6%) had synovial tissue suitable for subsequent histological analysis (failure rate 10.4%). Using the algorithm described in the Methods, we classified patients into three distinct synovial pathological groups (figure 1D): (1) *lympho-myeloid* (n=51, 39%) dominated by lymphoid lineage infiltration (T cells, B cells, plasma cells) in addition to myeloid cells; (2) *diffuse-myeloid* (n=44, 34%) with myeloid lineage predominance but poor in B cells/plasma-cells and (3) *pauci-immune* (n=34, 27%) characterised by fibroblasts expansion but scanty immune cells. When evaluating prevalence of pathotype according to joint biopsied, we noted a higher proportion of pauci-immune pathotype in small- and medium-sized joints (figure 1D).

### Identification of pathotype-specific gene expression markers

**Figure 2:**
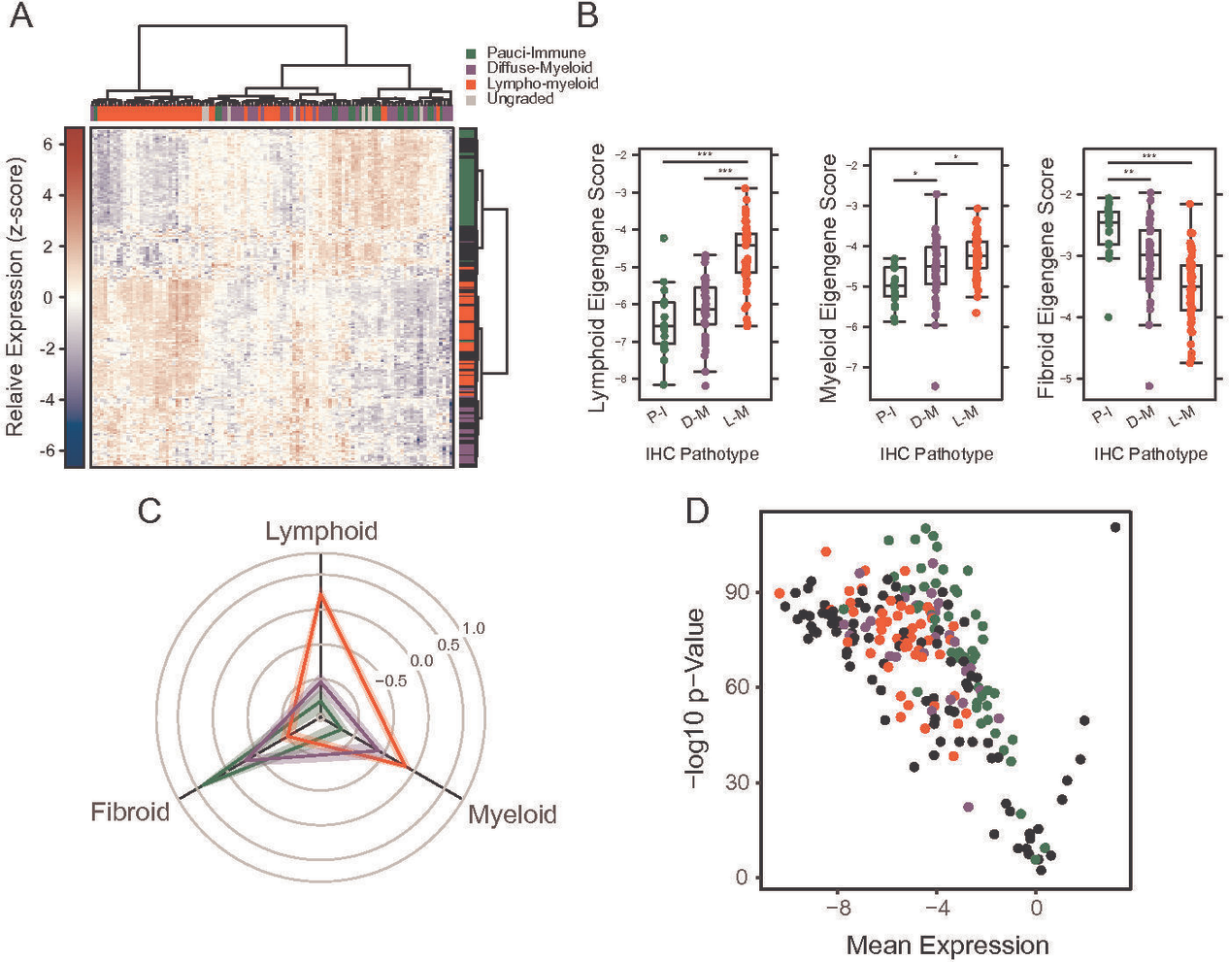
(A) Heatmap of NanoString gene expression data. Raw log_2_ NanoString counts for 212 genes and 111 patient samples were normalised per probe to give a mean of 0 and SD of 1. Normalised data were clustered by row and column using Euclidean distance and Ward’s linkage. Samples are coloured according to IHC-determined pathotype, with ungraded samples coloured grey. Rows are coloured according to the pathotype with which the gene was originally associated, with RA biology-associated genes coloured black. (B) Eigengene scores versus IHC-determined pathotypes. Individual eigengene scores are plotted for each sample, grouped and coloured by the pathotype as determined by IHC. Stars represent statistical significance as determined by linear regression across groups: *p<0.05, **p<0.01, ***p<0.001. (C) Radar plot of standardised eigengene scores. Eigengene values were normalised to give a mean 0 and SD of 1. Samples were grouped by pathotype, and the mean (solid lines) and SE of the mean (shaded region) were calculated for the normalised eigengenes. Spokes of the radar plot represent the distance along each normalised eigengene for each sample group. (D) Volcano plot of gene expression differences across pathotypes. For each gene, one-way ANOVA was performed comparing expression across the three pathotypes. The –log_10_ p value from the one-way ANOVA is plotted against the root mean square of the log_2_ fold changes between each pair of eigengenes. Genes are coloured according to the pathotype in which it was initially identified, with RA biology-associated genes coloured black. ANOVA, analysis of variance; IHC, immunohistochemistry; RA, rheumatoid arthritis.

To determine the relationship of clinical characteristics with molecular profiling in pretreatment and post-treatment biopsies, we profiled gene expression of the 111 baseline and 68 post-treatment samples using a custom NanoString panel. Unsupervised clustering of NanoString expression data showed strong grouping of pathotype-defined genes in concordance with their initial pathotype assigned by histology (figure 2A). Samples classified as either lympho-myeloid or pauci-immune showed highest expression of the lymphoid or fibroid eigengene score, respectively, while samples classified as diffuse-myeloid had myeloid eigengene scores similar to the lympho-myeloid samples (figure 2B,C: Radar Plot). Furthermore, most genes measured significantly differed across the three groups; all but 1 of 212 genes had an adjusted p value <0.01 (figure 2D). Eigengene scores were then examined against synovial aggregational scores demonstrating strong correlations with the level of inflammatory infiltrate organisation (online supplementary figure 3A,B). Comparing each pathotype to the others, we found that pauci-immune-fibroid eigengene scores were negatively correlated with lymphoid and myeloid eigengene scores, whereas lymphoid and myeloid scores were positively correlated (online supplementary figure 4A). Comparing myeloid pathotype samples with the other two yielded fewer differentially expressed genes, with lower expression of lymphoid-specific genes in these samples relative to the combined lympho-myeloid/pauci-immune-fibroid group (online supplementary figure 4B). This is consistent with the scoring algorithm used to determine the pathotypes: diffuse-myeloid samples mainly differ from lympho-myeloid samples by a relative lack of B- and T-cell infiltrate. Thus, the eigengene-specific gene sets show strong association with the expected pathotypes.

### Synovial pathotypes and clinical phenotypes

**Figure 3:**
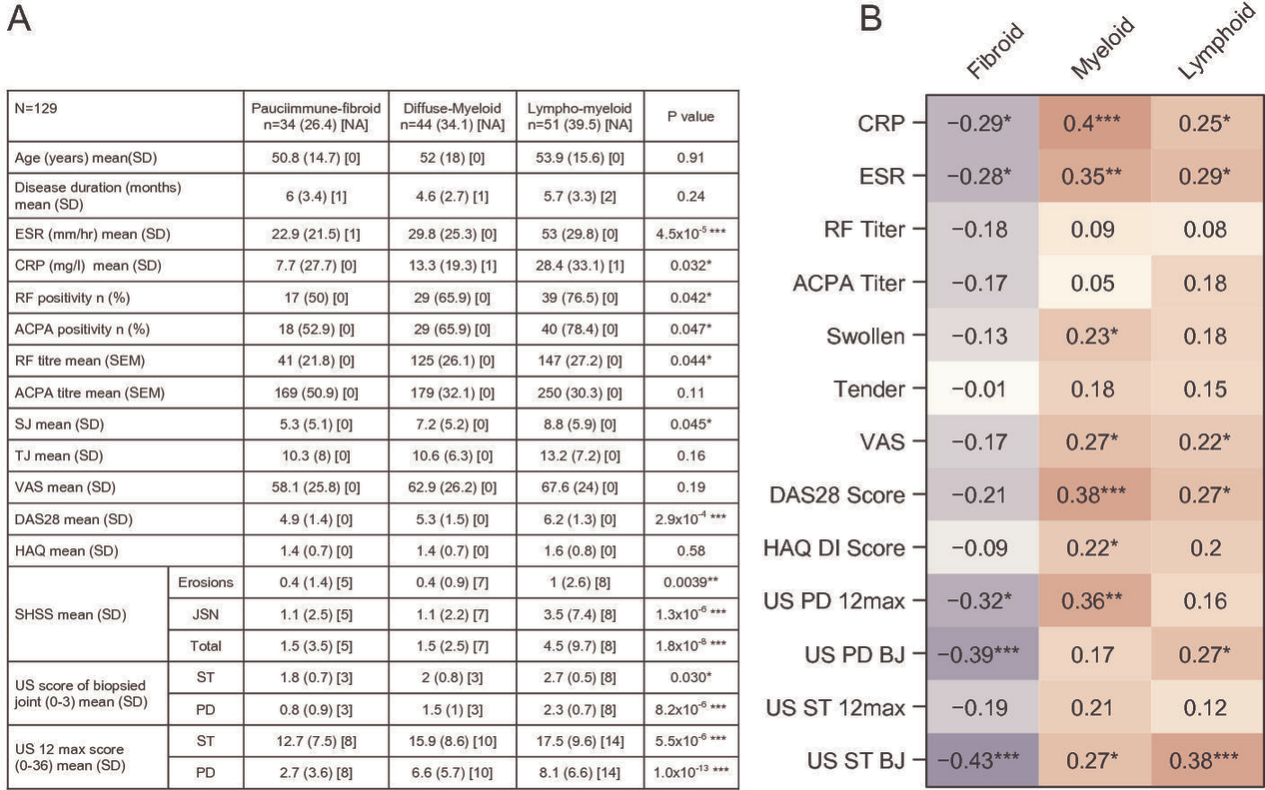
(A) Baseline clinical and histological parameters stratified according to three pathological subtypes, adjusted for joint type (n=129), *significant differences. (B) Correlation analysis of each Eigengene score with metrics of clinical disease activity, autoantibodies, acute phase reactants and ultrasonography. Values represent Spearman correlation coefficients between the clinical variables and the individual eigengene scores adjusted for joint type. Stars represent the significance of the correlation coefficient: *p<0.05, **p<0.01, ***p<0.001. ACPA, anti-citrullinated peptide antibodies; BJ, biopsied joint; CRP, C-reactive protein; DAS28, disease activity score-28; DI, Disability Index; ESR, erythrocyte sedimentation rate; HAQ, Health Assessment Questionnaire; RF, rheumatoid factor; SHSS, van der Heijde modified Sharp score; SJ, swollen joints; ST, synovial thickening; TJ, tender joints; US, ultrasound, VAS, visual analogue scale.

To establish the relationship between synovial pathotypes and clinical phenotypes, we compared a number of disease characteristics with the three synovial pathotypes defined by immunohistochemistry (IHC), with statistical adjustment for biopsy joint position. The lympho-myeloid pathotype had the highest levels of erythrocyte sedimentation rate (ESR), C-reactive protein, ACPA titre, swollen-joint-counts and DAS28-ESR scores (figure 3A). Assessment of joint damage by SHSS indicated an association of the lympho-myeloid group with worse erosive load and joint space narrowing (figure 3A). Furthermore, ultrasonographic assessment indicated that the lympho-myeloid group had significantly higher levels of ST and PD scores both within the biopsied joint and overall scores. The pauci-immune group had the lowest levels of acute phase reactants, RF and ACPA positivity, and US scores despite the presence of active disease, documented by high DAS28-ESR (mean 4.9), swollen joint counts, Health Assessment Questionnaire (HAQ) and patient global health visual analogue scale (VAS) scores as well as synovitis determined by ultrasonography (figure 3A). The diffuse-myeloid group showed intermediate metrics.

### Lymphoid/myeloid gene expression signatures correlate with RA disease activity

Comparison of eigengene scores to features of disease activity (figure 3B) showed that the myeloid eigengene was highly associated with disease activity, including acute phase reactant, DAS28-ESR, HAQ-Disability Index (DI) and overall PD ultrasonography score. The lymphoid eigengene was also correlated with disease activity, but at a lower level, and was more associated with ultrasonographic scores. In keeping with the histopathological correlations, the fibroid eigengene was negatively associated with many aspects of disease activity (figure 3B).

### Serum biomarkers reflect synovial pathophysiology

**Figure 4:**
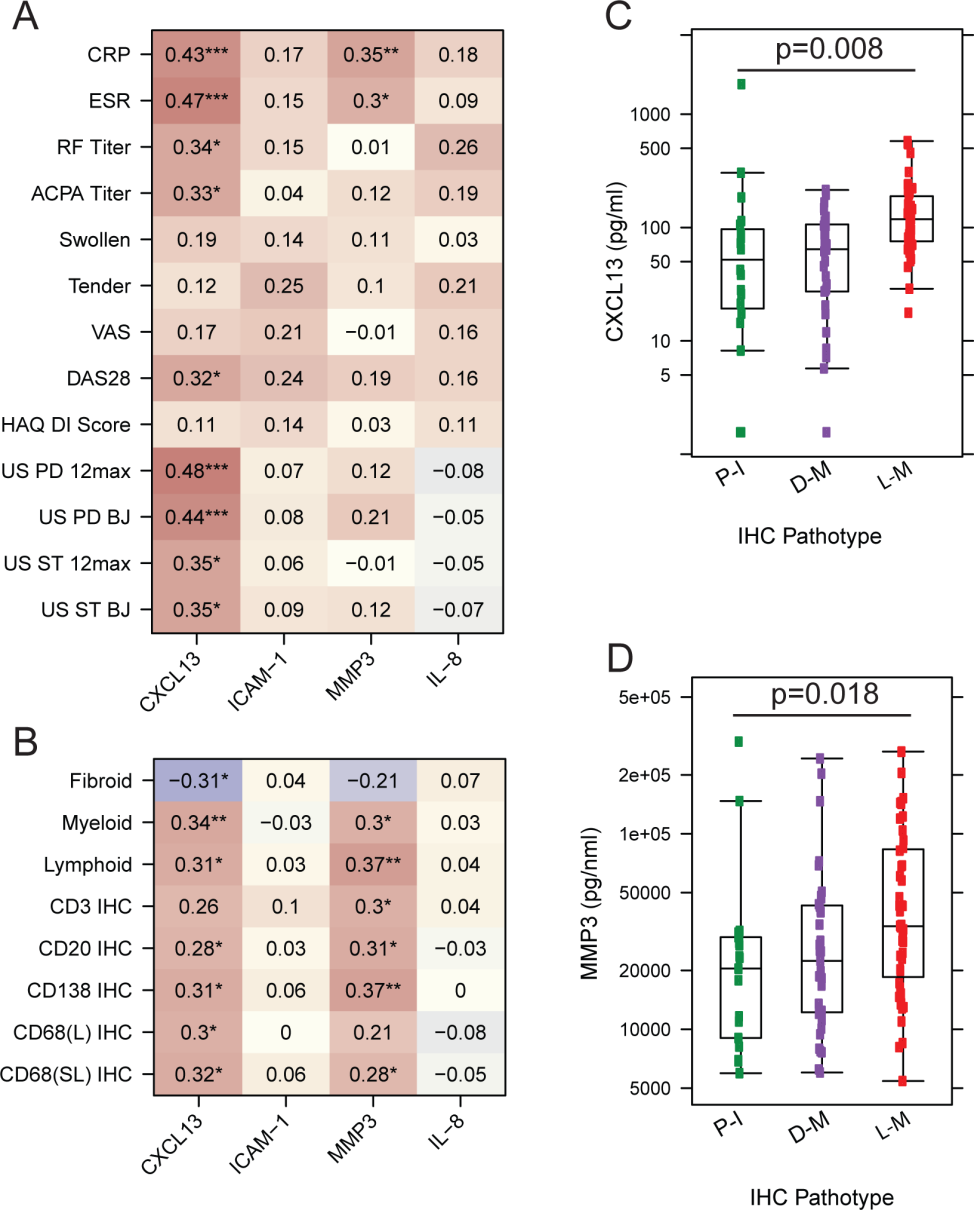
(A) Correlation of pretreatment serum CXCL13, sICAM1, MMP3 and IL-8 with clinical disease metrics and ultrasonography scores. Values represent Spearman correlation coefficients between serum biomarkers and clinical variables. Stars represent the significance of the correlation coefficient: *p<0.05, **p<0.01, ***p<0.001. P values were corrected for multiple testing using Benjamini-Hochberg method. (B) Correlation of pretreatment serum CXCL13, sICAM1, MMP3 and IL-8 with synovial histology scores. Values represent Spearman correlation with individual eigengene scores adjusted for biopsy joint size, or histology semiquantitative scores. (C) Concentration of serum CXCL13 versus synovial pathotype status. (D) Concentration of serum MMP3 versus synovial pathotype status. P values were calculated using student t-test, with correction for multiple testing. ACPA, anti-citrullinated protein antibodies; BJ, biopsied joint; CRP, C-reactive protein; CXCL13, C-X-C motif-chemokine-13; DAS28, disease activity score-28; DI, Disability Index; ESR, erythrocyte sedimentation rate; HAQ, Health Assessment Questionnaire; IHC, immunohistochemistry; IL-8, interleukin-8; MMP-3, matrix metalloproteinase-3; PD, power Doppler; RF, rheumatoid factor; sICAM1, soluble intercellular adhesion molecule 1; ST, synovial thickening; US, ultrasound; VAS, visual analogue scale.

We next assessed if reported circulating serum biomarkers could function as surrogates of synovial tissue pathology^2^ and reliable measures of disease activity. We selected CXCL13, sICAM-1, MMP3 and IL-8 due to their important inflammatory roles in RA pathophysiology. CXCL13 and sICAM1 have been previously shown to be elevated in different synovial phenotypes,^2^ whereas MMP3 is also elevated in synovitis and associated with radiographic progression.^12^ IL-8 drives tissue recruitment of neutrophils (polymorphonuclear neutrophils), which could play a role in synovial autoimmunity through the exposure of citrullinated proteins by neutrophil extracellular traps.^13^ Consistent with previous reports,^14 15^ we found that serum CXCL13 correlated with global disease metrics, including DAS28, serological and ultrasonographic measures of disease activity and synovial histology (figure 4A,B). Serum MMP-3 also showed modest yet significant correlation with acute phase reactants, DAS28 score and synovial histology (figure 4A,B). Intriguingly, CXCL13 and MMP3 were both elevated in patients with a lymphoid–myeloid pathotype, compared with the other two pathotypes (figure 4C,D). In contrast, despite previous reports of elevated levels in RA patients,^2 16^ ICAM-1 and IL-8 exhibited modest and variable correlations with clinical indices such as tender joint scores, acute phase reactants and autoantibody titres (figure 4A,B). Therefore, elevation of some but not all inflammatory proteins in the serum of RA patients tracks with synovitis and clinical disease activity.

### Pretreatment synovial pathotypes gene expression signatures associate with response to DMARD treatment

**Figure 5:**
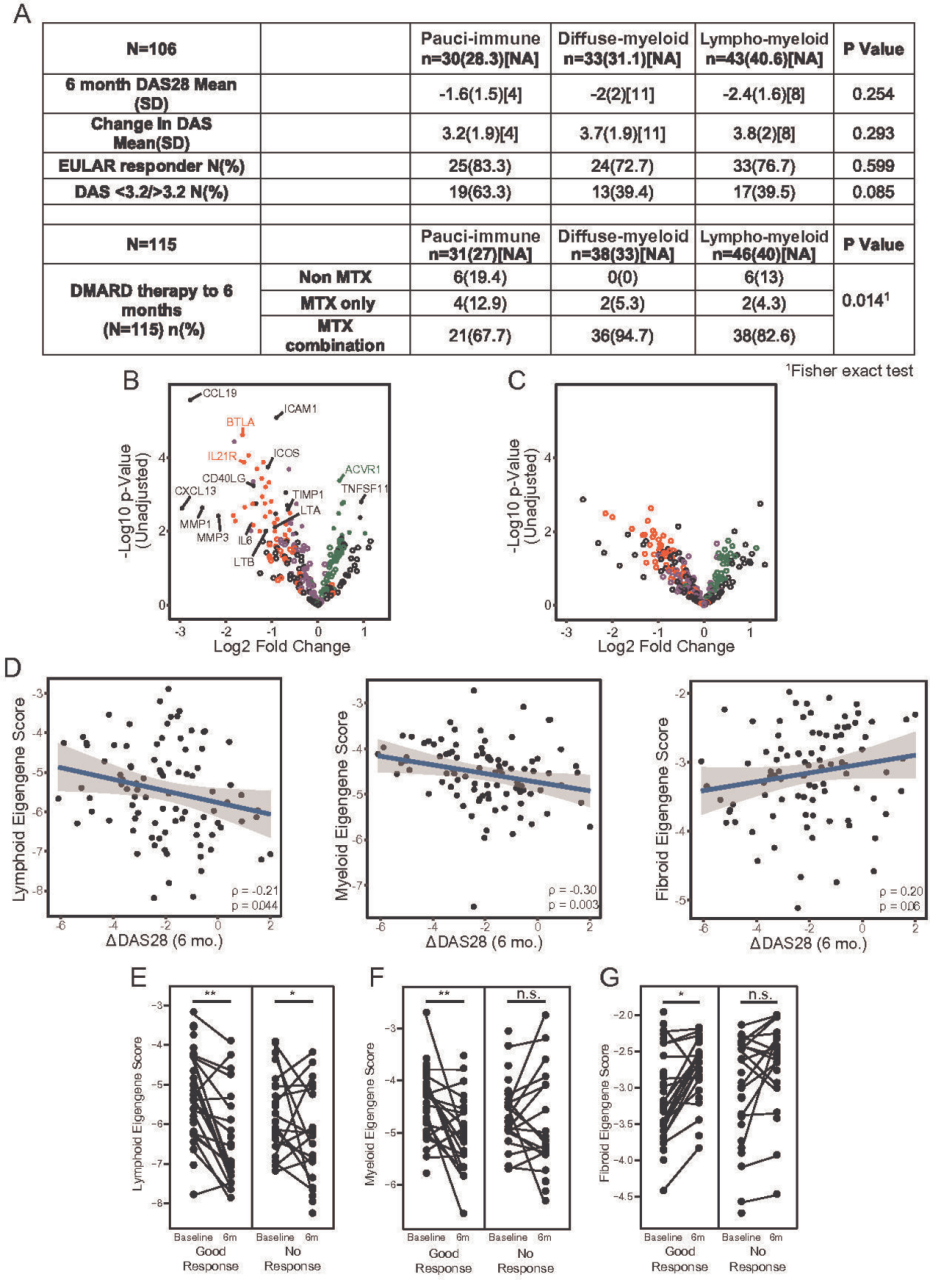
(A) Clinical changes in disease activity and treatment regimens stratified according to pathotype. (B) Volcano plot showing changes in gene expression between baseline and 6 months in patients with a EULAR response. Individual points are coloured by the pathotype in which the gene was originally identified, with RA biology-associated genes coloured black. (C) Volcano plot showing changes in gene expression between baseline and 6 months in patients with EULAR nonresponse. Genes are coloured as above. (D) Correlation of pretreatment lymphoid, myeloid and fibroid eigengene scores with change in DAS28-ESR after 6 months of DMARD treatment. Spearman’s correlation coefficient is shown, along with the significance of this value. (E–G) Paired plots for baseline and 6 month lymphoid (E), myeloid (F) and fibroid (G) eigengene scores in patients who achieved good or poor clinical responses to DMARD treatment at 6 months by the EULAR response criteria. Patients who achieved a good response, or failed to achieve a moderate response, according to EULAR criteria are shown. For each patient, the pretreatment eigengene scores are connected to the post-treatment eigengene score, for each of the three eigengenes. Stars represent significance of the difference between pretreatment and post-treatment samples using a linear mixed effects model with sample date as a fixed effect and patient as a random effect: *p<0.05, **p<0.01, ***p<0.001. DAS28, disease activity score-28; DMARD, disease-modifying antirheumatic drug; ESR, erythrocyte sedimentation rate; EULAR, European League Against Rheumatism; MTX, methotrexate.

Histologically defined pathotypes and gene expression signatures were examined for their response to DMARD therapy as determined by change in DAS28-ESR at 6 months and EULAR response criteria (figure 5). Overall, 90% of patients were treated with methotrexate either alone or in combination (figure 5A).

Though histologically defined pathotypes did not associated with therapeutic outcome (figure 5A), differential gene expression analysis in EULAR good-responder patients showed that multiple inflammatory pathways were statistically significantly reduced including genes associated with lymphoid aggregates (*CCL19*, *BTLA*, *IL21R*, *CXCL13*, *LTA*, *LTB*) and inflammatory cytokines (*IL6*) (figure 5B). In contrast, nonresponder patients showed decrease in nonsignificant inflammatory gene expression (figure 5C).

We next assessed whether eigengene scores at baseline correlated with therapeutic response. Higher myeloid and lymphoid eigengene expression (but not fibroid) was associated with larger decreases in DAS28-ESR scores post-treatment (myeloid, p=0.003; lymphoid, p=0.044; figure 5D). Specific eigengene scores were then compared separately in good and nonresponders (figure 5E–G) determining that good DMARD response showed more dynamic gene expression, with significant decreases in both lymphoid and myeloid eigengenes, but a concomitant increase in fibroid eigengene expression. Nonresponders exhibited a more muted change in gene expression, with significant decrease in lymphoid eigengenes, but highly variable changes in myeloid and fibroid eigengene expression indicating ongoing presence of myeloid gene expression in patients with continuing disease activity despite DMARD therapy, but strong downregulation in responder patients.

### Synovial pathotypes and gene expression signatures predict radiographic progression

**Figure 6:**
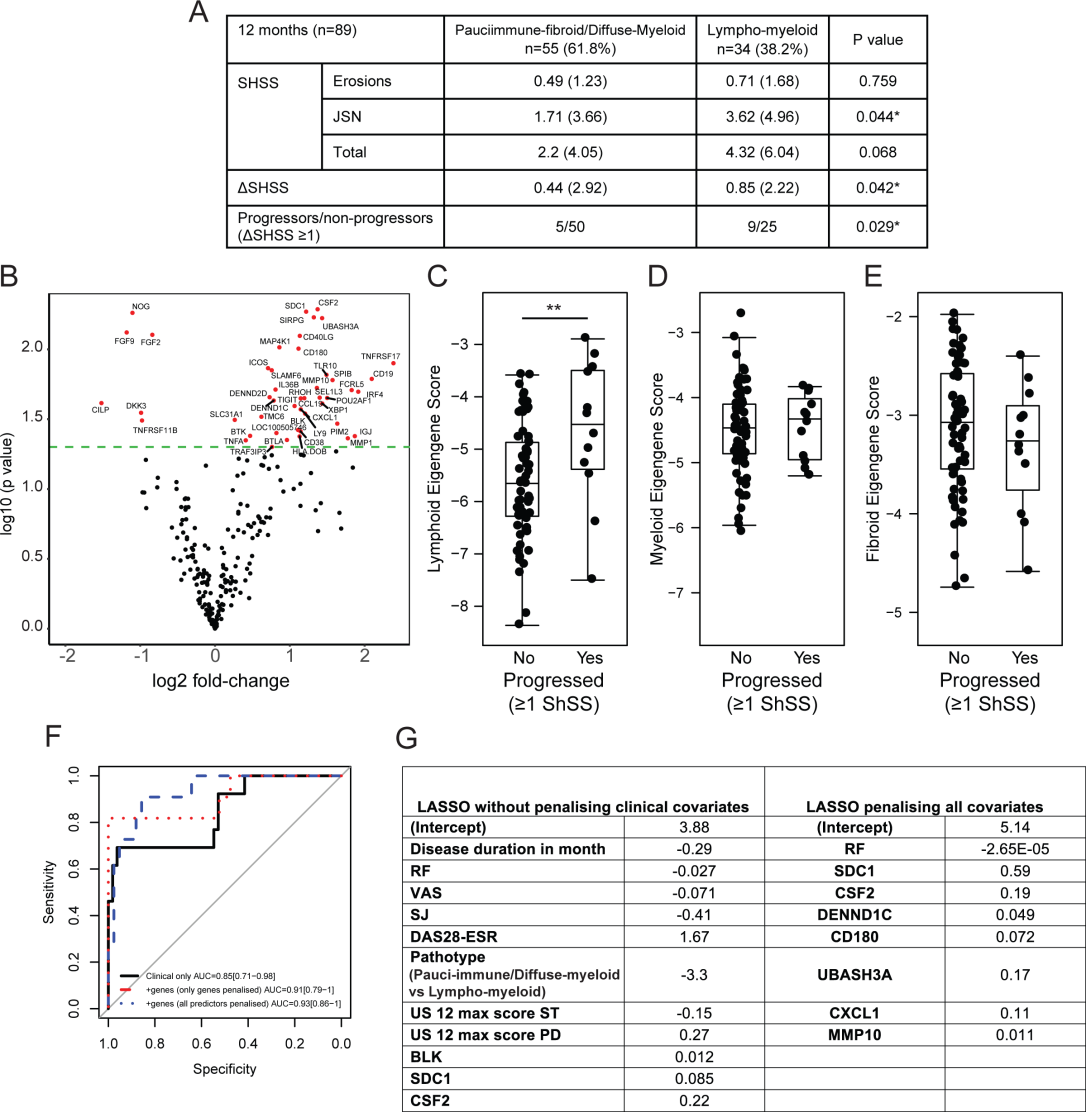
(A) 12-month radiographic outcome of patients stratified according to pauci-immune-fibroid/diffuse-myeloid versus lympho-myeloid pathotypes. (B) Volcano plot of pretreatment genes differentially expressed between patients who progress radiographically after 1 year (ΔSHSS≥1); P values were from the two-sample t-test comparing the progressors and nonprogressors without adjustment. In all, 46 genes with p value <0.05 are highlighted in red. (C–E) Baseline eigengene values versus radiographic progression. Lymphoid (C), myeloid (D) and pauci-immune-fibroid (E) baseline scores are plotted against progression status (ΔSHSS≥1) at 1 year. **p<0.01 by t-test. (F–G) Identification of clinical and gene expression features predictive of radiographic progression at 1 year. Logistic regression, coupled with backward stepwise model selection, was applied to baseline clinical parameters against a dependent variable of radiographic progression or not at 12 months to select which clinical covariate contributed the most to the prediction; 16 baseline clinical covariates were considered as candidates in the regression model. Baseline variables included gender, age, disease duration, ESR, CRP, RF titre, ACPA titre (as continuous variables), VAS, tender and swollen joint number, baseline DAS28-ESR, EULAR response at 6 months (categorical), baseline HAQ, 12 max US ST and US PD scores and baseline pathotype (two categories: lympho-myeloid versus pauci-immune-fibroid/diffuse-myeloid). Stepwise variable selection yielded a model with eight clinical variables: baseline RF titre, disease duration, VAS, swollen joint number, DAS28-ESR, baseline pathotype, 12 max US ST and US PD scores. Selected covariates (46 genes plus 8 clinical covariates) were entered simultaneously into a logistic model with an L1 regularisation penalty (LASSO) in order to determine the optimal sparse prediction model. We have a better predictive performance of the model where clinical variables were penalised (F, blue-dashed line) than when they were not penalised (F, red-dotted line). (G) Nonzero weights associated with the final variables selected by the LASSO regression. ACPA, anti-citrullinated peptide antibodies; CRP, C-reactive protein; CXCL13, C-X-C motif-chemokine-13; DAS28-ESR, disease activity score-28-erythrocyte sedimentation rate; EULAR, European League Against Rheumatism; HAQ, Health Assessment Questionnaire; JSN, joint space narrowing; LASSO, least absolute shrinkage and selection operator; MMP-10, matrix metalloproteinase-10; RF, rheumatoid factor; SHSS, van der Heijde modified Sharp score; SJ, swollen joints; US ST, ultrasound synovial thickening; US PD, ultrasound power Doppler; VAS, visual analogue scale.

Next, we assessed whether baseline pathotypes or gene expression were associated with ongoing radiographic damage at 12 months. Lympho-myeloid pathotype patients had a greater change in SHSS compared with diffuse-myeloid and pauci-immune-fibroid patients (figure 6A). Importantly, of the 14 patients subsequently commenced on biological therapy between 6 and 12 months of follow-up, a higher proportion with radiographic progression at 12 months fell within the lympho-myeloid pathotype (26.5%, 9 progressors vs 25 nonprogressors), as compared with the diffuse-myeloid/pauci-immune-fibroid pathotypes (9.1%, 5 progressors vs 50 nonprogressors, Fishers exact test, p=0.029). Thus, despite more intensive treatment regimens (including higher rates of biologic use), patients with a lympho-myeloid pathotype were significantly more likely to develop joint damage progression.

Next, we compared pretreatment/baseline gene expression with SHSS progression scores (figure 6B); 46 genes with p value <0.05 were identified and these included B-cell-associated genes such as CD19, FCRL5 and BCMA. In agreement with these findings, we further noted that the pretreatment lymphoid eigengene score was significantly elevated in patients who had 12-month SHSS scores increase versus those who did not (figure 6C), whereas in contrast the myeloid and pauci-immune-fibroid eigengene scores were not significantly different between progressors versus non-progressors (figure 6D,E).

In order to explore mechanistically the link between the lympho-myeloid pathotype-associated gene signatures and structural damage, we investigated the expression of a predefined osteoclast gene set derived from Harmonizome database (http://amp.pharm.mssm.edu/Harmonizome/).

As expected, the osteoclast eigengene score strongly correlated with histology scores for both B cells, T cells, plasma cells and macrophages (online supplementary figure 5). However, when patients were grouped according to pathotype, the demonstration that osteoclast eigengene scores were most significantly elevated in the lympho-myeloid pathotype patients (p<0.01 lympho-myeloid vs diffuse-myeloid and p<0.001 vs pauci-immune) strongly suggests that the lymphoid/B-cell component of the immune cell infiltrate is orchestrating osteoclast activation and therefore driving joint erosion.

Finally, to determine whether baseline clinical and gene expression data could be combined into a model for predicting radiographic progression, we used two complementary approaches: (i) a logistic regression coupled with backward model selection to identify a minimal set of clinical predictors and (ii) a penalised method based on logistic regression with an L1 regularisation penalty (LASSO) to identify genes that improve the clinical model.

First, we used backward model selection on baseline clinical parameters to identify predictors of radiographic progression. The final clinical covariates included baseline RF titre, disease duration, VAS, swollen joint number, DAS28-ESR, baseline pathotype, 12 max US ST and US PD scores. The predictive performance of the model evaluated by the optimism-corrected AUC was 0.75 (online supplementary figure 6A).

Next, a logistic regression with an L1 regularisation penalty (LASSO) was applied on the eight clinical covariates and 46 genes (figure 6B) identified as being significantly differentially expressed between progressors and nonprogressors. We found that a model incorporating RF titre, and the expression of seven genes (*SDC1*, *CSF2*, *DENND1C*, *CD180*, *UBASH3A*, *CXCL1*, *MMP10*, figure 6F), yielded the optimal predictor of progression (lambda=0.0631, online supplementary figure 6B). After adjustment for optimism, our final prediction model was able to discriminate patients with and without radiographic progression with an optimism-corrected AUC of 0.88 (figure 6G). The predictive performance of the model was corrected for potential overfitting by computing the optimism-corrected AUC using a bootstrap method.^17 18^ These results suggest that including both clinical covariates and genes in the model resulted in a superior predictive value compared with clinical covariates alone, with improved discrimination between patients with and without radiographic progression.

## Discussion

It is recognised that RA pathology is highly heterogeneous^1–3 19 20^; however, how such heterogeneity relates to disease activity, prognosis and therapeutic response/outcome has been unclear. In addition, the reproducibility of defined histopathological categories, as well as their stability over time and/or relationship to different disease stages also remains uncertain. The work described here tackles these problems investigating longitudinally a large, treatment-naïve, early RA cohort, with serial synovial sampling pre-DMARD and post-DMARD therapy and stringent clinical and imaging assessments.

To address the issue of whether synovial heterogeneity is present in early versus established RA, we designed gene panels linked to lymphoid, macrophage-myeloid and fibroblast cell-lineages, trained on synovial subsets we reported in late-stage RA joint replacement synovial tissue,^2^ and tested them in the PEAC cohort. The data revealed that prevalent lympho-myeloid, diffuse-myeloid and pauci-immune pathotypes are indeed present in early RA tissue prior to potentially modifiable therapeutic-sensitive events.

Lympho-myeloid patients had the highest levels of disease activity and RF and ACPA seropositivity. Although previous definitions of synovial pathotype have referred to lymphoid, myeloid and fibroid subgroups importantly, the data analysed herein demonstrate that lymphoid-rich patients also showed high expression of myeloid genes hence the definition of ‘lympho-myeloid pathotype’ while another group of patients had a high expression of myeloid genes but low expression of B-cell genes. This latter group also showed a diffuse inflammatory infiltrate lacking cellular aggregates and such was defined as ‘diffuse myeloid’ while the ‘pauci-immune’ group showed a prevalence of stromal cells but almost complete absence of immune cells.

Of note, the myeloid gene score was most strongly positively correlated at baseline with clinical signs and symptoms including acute phase reactant, joint counts, DAS28-ESR, VAS, HAQ-DI. Importantly, subsequent treatment of these patients with DMARDs indicated that the pretreatment levels of myeloid genes were most strongly correlated with treatment outcome at 6 months and, further, that significant reduction of myeloid gene scores was only observed in patients who achieved a robust clinical response. This suggests that while higher myeloid and lymphoid gene expression is associated with increased disease activity, these are the patients more likely to respond to broad DMARD immune suppression, as pretreatment levels correlated with therapeutic outcome. These findings are consistent with previous reports indicating that the mean change in sublining macrophages is a strong biomarker of treatment response in RA.^21^ They also indicate that the myeloid lineage is a key driver in RA pathogenesis and specific therapeutic targeting improves signs and symptoms, although not specifically joint damage progression as previously suggested in a small cohort of RA patients with long-standing disease.^22^ It is worth reinforcing that the broad anti-inflammatory activities of methotrexate via mechanisms including activation of the adenosine pathway and down-modulation of adhesion molecules and proteases^23^ are supported by our data indicating that multiple immune pathways are suppressed by methotrexate treatment.

Importantly, patients with high expression of fibroid genes but low expression of lymphoid and myeloid genes were found in 27% of the cohort, suggesting that this fibroid/pauci-immune phenotype previously reported in joint replacement tissue appears to be a defined disease endotype and not an end-stage (burned out) disease characteristic. Furthermore, the demonstration of a significant difference in pathotype frequency between joints highlights the importance of techniques such as US-guided synovial biopsy to enable unbiased recruitment of patients, through capacity to sample a wide variety of joints, and thus ensure representative sampling. While in our study we did not perform simultaneous biopsies of different joints in the same patient, so cannot address per se whether synovial pathotype/molecular signatures are stable between joints of the same patient, it important to note that previous studies have documented stable cellular infiltrates^24^ and specific T-cell oligoclonal expansions^25^ between joints within the same individuals, supporting the notion of a uniform pathology at individual patient level across multiple joints.

Regarding the relationship of lineage-specific modules and clinical/therapeutic outcomes, it is noteworthy that while fibroid gene scores correlated with less severe disease, these patients still had active disease (mean DAS28: 4.9). Moreover, these patients showed the poorest subsequent response to DMARD treatment while successful drug treatment of the patients with lympho-myeloid and diffuse-myeloid pathotypes resulted in elevation of repair-response genes. These results indicate that DMARD treatment primarily reduces inflammatory cell infiltrate, thus changing the cellular composition of the synovium. Furthermore, as the fibroid genes themselves are not reduced with current immune modulatory therapies, our data support the notion that new therapies are required to target this specific cell-lineage known to play a critical role in pathogenesis/joint damage through epigenetic modifications leading to an aggressive phenotype even in the absence of immune/inflammatory cells.^26^

The strong correlation between radiographic damage (baseline and 12 months), lympho-myeloid pathotype and associated osteoclast-targeting genes, suggests that active immunological processes drive inflammation and structural damage, accelerated by high B-cell infiltration levels. This is concordant with previous reports showing that B cells and plasma cells produce RANKL and TWEAK^27–29^ while their number was elevated in patients with MRI-determined bone oedema and increased RANKL expression.^30^ In contrast, nonprogressors had elevated baseline levels of osteoprotegerin (a decoy receptor/negative regulator for RANKL-mediated osteoclastogenesis) and fibroid-associated genes including fibroblast growth factor family members, Noggin and cartilage intermediate layer protein (*CILP*).

In summary, the results presented herein indicate that diverse synovial pathotypes are present at an early disease stage prior to potential drug-sensitive modification of disease pathology. Furthermore, we demonstrated that specific synovial pathotypes and related molecular signatures are associated with clinical phenotypes, disease outcome/prognosis, that is, radiographic progression and moreover that specific molecular signatures predict response to therapy. These data support the evaluation of such biomarkers within randomised clinical trials with the aim of enriching response to current biological therapies by targeting the specific cognate pathways expressed in some patients but not others, while offering the opportunity, similar to cancer medicine, of developing pathway-driven/stratified approaches to patients with RA.
