# Supplementary material for: Synovial cellular and molecular signatures stratify clinical response to csDMARD therapy and predict radiographic progression in early rheumatoid arthritis patients
Source: Ann Rheum Dis. 2019 Mar 16;78(6):761–72. doi: 10.1136/annrheumdis-2018-214539 (PMC6579551; doi:10.1136/annrheumdis-2018-214539)
Supplement: Supplementary data [file annrheumdis-2018-214539supp002.pdf]

Cellular and Molecular Signatures in the Disease Tissue of Early Rheumatoid Arthritis Stratify Clinical Response to csDMARD-Therapy and Predict Radiographic Progression

Frances Humby[1],* Myles Lewis[1],* Nandhini Ramamoorthi[2], Jason Hackney[3], Michael Barnes[1], Michele Bombardieri[1], Francesca Setiadi[2], Stephen Kelly[1], Fabiola Bene[1], Maria di Cicco[1], Sudeh Riahi[1], Vidalba Rocher-Ros[1], Nora Ng[1], Ilias Lazorou[1], Rebecca E. Hands[1], Desiree van der Heijde[4], Robert Landewé[5], Annette van der Helm-van Mil[4], Alberto Cauli[6], Iain B. McInnes[7], Christopher D. Buckley[8], Ernest Choy[9], Peter Taylor[10], Michael J. Townsend[2] & Costantino Pitzalis[1]

[1]Centre for Experimental Medicine and Rheumatology, William Harvey Research Institute, Barts and The London School of Medicine and Dentistry, Queen Mary University of London, Charterhouse Square, London EC1M 6BQ, UK.
Departments of [2]Biomarker Discovery OMNI, [3]Bioinformatics and Computational Biology, Genentech Research and Early Development, South San Francisco, California 94080 USA
[4]Department of Rheumatology, Leiden University Medical Center, The Netherlands
[5]Department of Clinical Immunology & Rheumatology, Amsterdam Rheumatology & Immunology Center, Amsterdam, The Netherlands
[6]Rheumatology Unit, Department of Medical Sciences, Policlinico of the University of Cagliari, Cagliari, Italy
[7]Institute of Infection, Immunity and Inflammation, University of Glasgow, Glasgow G12 8TA, UK
[8]Rheumatology Research Group, Institute of Inflammation and Ageing (IIA), University of Birmingham, Birmingham B15 2WB, UK
[9]Institute of Infection and Immunity, Cardiff University School of Medicine, Cardiff CF14 4XN, UK
[10]Nuffield Department of Orthopaedics, Rheumatology and Musculoskeletal Sciences and the Kennedy Institute of Rheumatology, University of Oxford, Oxford, UK

FH, ML, NR and JH contributed equally to the study

Correspondence: Professor Costantino Pitzalis – c.pitzalis@qmul.ac.uk
Dr. Michael J. Townsend – townsem1@gene.com

Supplementary Tables 1-3

Pathotype-related Genes and microarray probes as defined in the Michigan RA cohort

Table 1: Lymphoid Genes and Probes

ProbeId	EntrezId	Symbol	GeneName
243968_x_at	115350	FCRL1	Fc receptor-like 1
230551_at	283455	KSR2	kinase suppressor of ras 2
1561411_at	102800316	LINC01222	long intergenic non-protein coding RNA 1222
1561335_at	440602	LOC440602	uncharacterized LOC440602
1559861_at	101928099	PCAT7	prostate cancer associated transcript 7 (non-protein coding)
214735_at	26034	IPCEF1	interaction protein for cytohesin exchange factors 1
207579_at	4114	MAGEB3	melanoma antigen family B, 3
233052_at	1769	DNAH8	dynein, axonemal, heavy chain 8
219424_at	10148	EBI3	Epstein-Barr virus induced 3
232170_at	338324	S100A7A	S100 calcium binding protein A7A
1562888_at	112937	GLB1L3	galactosidase, beta 1-like 3
229971_at	221188	GPR114	G protein-coupled receptor 114
206983_at	1235	CCR6	chemokine (C-C motif) receptor 6
1556924_at	65072	CFLAR-AS1	CFLAR antisense RNA 1
235139_at	2793	GNGT2	guanine nucleotide binding protein (G protein), gamma transducing activity polypeptide 2
221459_at	9038	TAAR5	trace amine associated receptor 5
207035_at	7781	SLC30A3	solute carrier family 30 (zinc transporter), member 3
1552497_a_at	114836	SLAMF6	SLAM family member 6
1552892_at	115650	TNFRSF13C	tumor necrosis factor receptor superfamily, member 13C
214551_s_at	924	CD7	CD7 molecule
223751_x_at	81793	TLR10	toll-like receptor 10
1564263_at	144817	LINC00330	long intergenic non-protein coding RNA 330
1562728_at	100996665	LINC01533	long intergenic non-protein coding RNA 1533
1554716_s_at	414926	LINC00593	long intergenic non-protein coding RNA 593
220727_at	54207	KCNK10	potassium channel, subfamily K, member 10
1568791_s_at	100505518	EDNRB-AS1	EDNRB antisense RNA 1
210439_at	29851	ICOS	inducible T-cell co-stimulator
221239_s_at	79368	FCRL2	Fc receptor-like 2
205813_s_at	4143	MAT1A	methionine adenosyltransferase I, alpha
214519_s_at	6019	RLN2	relaxin 2
219014_at	51316	PLAC8	placenta-specific 8
240842_at	101927020	LOC101927020	uncharacterized LOC101927020
218600_at	80774	LIMD2	LIM domain containing 2
220485_s_at	55423	SIRPG	signal-regulatory protein gamma
222782_s_at	51291	GMIP	GEM interacting protein
1562786_at	101928886	LOC101928886	uncharacterized LOC101928886
1570266_x_at	100616446	ERVH-1	endogenous retrovirus group H, member 1
1559097_at	388011	C14orf64	chromosome 14 open reading frame 64
1553647_at	124359	CDYL2	chromodomain protein, Y-like 2
210724_at	84658	EMR3	egf-like module containing, mucin-like, hormone receptor-like 3
230978_at	100505659	LINC00967	long intergenic non-protein coding RNA 967
239020_at	84750	FUT10	fucosyltransferase 10 (alpha (1,3) fucosyltransferase)
1562841_at	339666	LOC339666	uncharacterized LOC339666
236855_at	404216	C10orf85	chromosome 10 open reading frame 85
236854_at	284749	LINC00494	long intergenic non-protein coding RNA 494
220714_at	63978	PRDM14	PR domain containing 14
243406_at	100652857	TMCO5B	transmembrane and coiled-coil domains 5B, pseudogene
1558630_at	101926987	DLGAP4-AS1	DLGAP4 antisense RNA 1
1559591_s_at	55349	CHDH	choline dehydrogenase
1560725_at	101928376	IL12A-AS1	IL12A antisense RNA 1
230863_at	4036	LRP2	low density lipoprotein receptor-related protein 2
205291_at	3560	IL2RB	interleukin 2 receptor, beta
233135_at	100506527	LINC01007	long intergenic non-protein coding RNA 1007

211687_x_at	3811	KIR3DL1	killer cell immunoglobulin-like receptor, three domains, long cytoplasmic tail, 1
224401_s_at	83417	FCRL4	Fc receptor-like 4
1562939_at	55604	LRRC16A	leucine rich repeat containing 16A
215331_at	22989	MYH15	myosin, heavy chain 15
1569805_at	101927274	LOC101927274	uncharacterized LOC101927274
236024_at	2823	GPM6A	glycoprotein M6A
220068_at	29802	VPREB3	pre-B lymphocyte 3
1553272_at	206358	SLC36A1	solute carrier family 36 (proton/amino acid symporter), member 1
1560631_at	10241	CALCOCO2	calcium binding and coiled-coil domain 2
1552806_a_at	89790	SIGLEC10	sialic acid binding Ig-like lectin 10
232739_at	6689	SPIB	Spi-B transcription factor (Spi-1/PU.1 related)
237493_at	116379	IL22RA2	interleukin 22 receptor, alpha 2
1553519_at	246705	LINC00314	long intergenic non-protein coding RNA 314
1556739_at	283796	GOLGA8I	golgin A8 family, member I
224257_s_at	59351	PBOV1	prostate and breast cancer overexpressed 1
1556929_at	64895	PAPOLG	poly(A) polymerase gamma
228903_at	283848	CES4A	carboxylesterase 4A
235977_at	164832	LONRF2	LON peptidase N-terminal domain and ring finger 2
1560812_at	101928708	LOC101928708	uncharacterized LOC101928708
209995_s_at	8115	TCL1A	T-cell leukemia/lymphoma 1A
1563007_at	101929241	LOC101929241	uncharacterized LOC101929241
1554583_a_at	254013	METTL20	methyltransferase like 20
1558832_at	440584	SLC2A1-AS1	SLC2A1 antisense RNA 1
228592_at	931	MS4A1	membrane-spanning 4-domains, subfamily A, member 1
233990_at	56006	SMG9	SMG9 nonsense mediated mRNA decay factor
236852_at	286151	FBXO43	F-box protein 43
230021_at	90381	TICRR	TOPBP1-interacting checkpoint and replication regulator
219775_s_at	594855	CPLX3	complexin 3
221407_at	57369	GJD2	gap junction protein, delta 2, 36kDa
215594_at	100616497	ERV9-1	endogenous retrovirus group 9, member 1
204674_at	4033	LRMP	lymphoid-restricted membrane protein
224006_at	100507377	LOC100507377	uncharacterized LOC100507377
1553335_x_at	285696	LOC285696	uncharacterized LOC285696
210279_at	2841	GPR18	G protein-coupled receptor 18
216666_at	93432	LOC93432	maltase-glucoamylase (alpha-glucosidase)
1561050_a_at	100874305	NAV2-IT1	NAV2 intronic transcript 1 (non-protein coding)
228787_s_at	55653	BCAS4	breast carcinoma amplified sequence 4
220375_s_at	100996485	C5orf66	chromosome 5 open reading frame 66
231236_at	346171	ZFP57	ZFP57 zinc finger protein
37793_r_at	5892	RAD51D	RAD51 paralog D
1569996_at	100101938	ANKRD26P3	ankyrin repeat domain 26 pseudogene 3
242810_x_at	100132874	LOC100132874	uncharacterized LOC100132874
219812_at	79037	PVRIG	poliovirus receptor related immunoglobulin domain containing
231595_at	100129827	MRVI1-AS1	MRVI1 antisense RNA 1
210020_x_at	810	CALML3	calmodulin-like 3
1569696_at	402779	CSMD2-AS1	CSMD2 antisense RNA 1
1553558_at	259287	TAS2R41	taste receptor, type 2, member 41
1561319_at	100309464	OTX2-AS1	OTX2 antisense RNA 1 (head to head)
1553561_at	259296	TAS2R50	taste receptor, type 2, member 50
1562664_at	286009	LOC286009	uncharacterized LOC286009
229592_at	100507501	LOC100507501	uncharacterized LOC100507501
211583_x_at	259197	NCR3	natural cytotoxicity triggering receptor 3
1569767_at	101927131	LOC101927131	uncharacterized LOC101927131
207666_x_at	10214	SSX3	synovial sarcoma, X breakpoint 3
232377_at	30010	NXPH1	neurexophilin 1
1570020_at	729522	AACSP1	acetoacetyl-CoA synthetase pseudogene 1
215274_at	6559	SLC12A3	solute carrier family 12 (sodium/chloride transporter), member 3
1562601_at	400952	LINC01121	long intergenic non-protein coding RNA 1121
208267_at	56302	TRPV5	transient receptor potential cation channel, subfamily V, member 5
224274_at	84075	FSCB	fibrous sheath CABYR binding protein
1564125_at	285857	LOC285857	uncharacterized LOC285857

1569468_at	642280	ZNF876P	zinc finger protein 876, pseudogene
1564921_at	140258	KRTAP13-1	keratin associated protein 13-1
233143_at	140873	C20orf173	chromosome 20 open reading frame 173
242683_at	400643	LINC00668	long intergenic non-protein coding RNA 668
208406_s_at	9402	GRAP2	GRB2-related adaptor protein 2
236986_at	100287814	LINC00582	long intergenic non-protein coding RNA 582
235372_at	84824	FCRLA	Fc receptor-like A
231796_at	2046	EPHA8	EPH receptor A8
219870_at	80063	ATF7IP2	activating transcription factor 7 interacting protein 2
206226_at	3273	HRG	histidine-rich glycoprotein
228258_at	374403	TBC1D10C	TBC1 domain family, member 10C
244543_s_at	100286844	BCDIN3D-AS1	BCDIN3D antisense RNA 1
1552596_at	246176	GAS2L2	growth arrest-specific 2 like 2
205456_at	916	CD3E	CD3e molecule, epsilon (CD3-TCR complex)
1563800_at	283140	LOC283140	uncharacterized LOC283140
205268_s_at	119	ADD2	adducin 2 (beta)
234637_at	85289	KRTAP4-5	keratin associated protein 4-5
210450_at	28387	IGHV5-78	immunoglobulin heavy variable 5-78 (pseudogene)
206900_x_at	56242	ZNF253	zinc finger protein 253
232936_at	3743	KCNA7	potassium voltage-gated channel, shaker-related subfamily, member 7
210031_at	919	CD247	CD247 molecule
1555313_a_at	4168	MCF2	MCF.2 cell line derived transforming sequence
244029_at	100131043	LOC100131043	uncharacterized LOC100131043
1561288_at	100859921	LINC00536	long intergenic non-protein coding RNA 536
1564039_at	222696	ZSCAN23	zinc finger and SCAN domain containing 23
230792_at	158584	FAAH2	fatty acid amide hydrolase 2
240565_at	140612	ZFP28	ZFP28 zinc finger protein
1561450_at	101928618	LOC101928618	uncharacterized LOC101928618
1554840_at	280665	LOC280665	anti-CNG alpha 1 cation channel translation product-like
210606_x_at	3824	KLRD1	killer cell lectin-like receptor subfamily D, member 1
234820_at	116511	MAS1L	MAS1 proto-oncogene like, G protein-coupled receptor
1553524_at	1607	DGKB	diacylglycerol kinase, beta 90kDa
234845_at	100130370	LOC100130370	uncharacterized LOC100130370
1559429_a_at	255798	SMCO1	single-pass membrane protein with coiled-coil domains 1
1559689_a_at	400581	GRAPL	GRB2-related adaptor protein-like
1555792_a_at	164592	CCDC116	coiled-coil domain containing 116
396_f_at	2057	EPOR	erythropoietin receptor
1552579_a_at	8747	ADAM21	ADAM metallopeptidase domain 21
206337_at	1236	CCR7	chemokine (C-C motif) receptor 7
1552816_at	158046	NXNL2	nucleoredoxin-like 2
1569009_s_at	100132249	LOC100132249	uncharacterized LOC100132249
206964_at	51471	NAT8B	N-acetyltransferase 8B (GCN5-related, putative, gene/pseudogene)
1553373_at	128025	WDR64	WD repeat domain 64
233551_at	642776	LOC642776	uncharacterized LOC642776
1564749_at	100289045	LOC100289045	uncharacterized LOC100289045
1559877_at	100289094	LOC100289094	uncharacterized LOC100289094
1564435_a_at	140807	KRT72	keratin 72
232664_at	400946	LINC00954	long intergenic non-protein coding RNA 954
1562590_at	283659	PRTG	protogenin
233075_at	100132101	HERC2P7	hect domain and RLD 2 pseudogene 7
206180_x_at	65988	ZNF747	zinc finger protein 747
237230_at	170589	GPHA2	glycoprotein hormone alpha 2
208059_at	1237	CCR8	chemokine (C-C motif) receptor 8
1553078_at	120066	OR5P3	olfactory receptor, family 5, subfamily P, member 3
214762_at	534	ATP6V1G2	ATPase, H+ transporting, lysosomal 13kDa, V1 subunit G2
211226_at	8811	GALR2	galanin receptor 2
234341_x_at	91548	LOC91548	uncharacterized LOC91548
1567036_at	100128998	C20orf181	chromosome 20 open reading frame 181
206626_x_at	6756	SSX1	synovial sarcoma, X breakpoint 1
233661_at	100861544	LINC00557	long intergenic non-protein coding RNA 557
205929_at	10223	GPA33	glycoprotein A33 (transmembrane)
1553917_at	200576	PIKFYVE	phosphoinositide kinase, FYVE finger containing

224520_s_at	144453	BEST3	bestrophin 3
1563528_at	91149	RAPGEF4-AS1	RAPGEF4 antisense RNA 1
1562749_at	644090	LOC644090	uncharacterized LOC644090
224285_at	84636	GPR174	G protein-coupled receptor 174
1552839_at	161142	FAM71D	family with sequence similarity 71, member D
236073_at	284656	EPHA10	EPH receptor A10
1569006_at	284379	LOC284379	solute carrier family 7 (cationic amino acid transporter, y+ system), member 3 pseudogene
237335_at	22917	ZP1	zona pellucida glycoprotein 1 (sperm receptor)
1554203_at	642976	GRIK1-AS1	GRIK1 antisense RNA 1
224367_at	84707	BEX2	brain expressed X-linked 2
1556464_a_at	257407	C2orf72	chromosome 2 open reading frame 72
205484_at	27240	SIT1	signaling threshold regulating transmembrane adaptor 1
221125_s_at	27094	KCNMB3	potassium large conductance calcium-activated channel, subfamily M beta member 3
1556829_at	100287227	TIPARP-AS1	TIPARP antisense RNA 1
1564287_at	144776	LINC00410	long intergenic non-protein coding RNA 410
206801_at	4879	NPPB	natriuretic peptide B
211814_s_at	9134	CCNE2	cyclin E2
230896_at	389206	BEND4	BEN domain containing 4
1566145_s_at	644450	LOC644450	uncharacterized LOC644450
1566513_a_at	2786	GNG4	guanine nucleotide binding protein (G protein), gamma 4
1563586_at	401324	LOC401324	uncharacterized LOC401324
1566147_a_at	100874392	ANKRD20A12P	ankyrin repeat domain 20 family, member A12, pseudogene
232738_at	401145	CCSER1	coiled-coil serine-rich protein 1
1566956_at	81137	OR7E104P	olfactory receptor, family 7, subfamily E, member 104 pseudogene
1561127_at	642394	ADARB2-AS1	ADARB2 antisense RNA 1
1556987_s_at	26716	OR2H1	olfactory receptor, family 2, subfamily H, member 1
220828_s_at	55338	FLJ11292	uncharacterized protein FLJ11292
1567069_at	26689	OR4D1	olfactory receptor, family 4, subfamily D, member 1
1561492_at	647107	LINC01192	long intergenic non-protein coding RNA 1192
230400_s_at	5586	PKN2	protein kinase N2
234016_at	90499	FAM95A	family with sequence similarity 95, member A
216661_x_at	1559	CYP2C9	cytochrome P450, family 2, subfamily C, polypeptide 9
208135_at	6928	HNF1B	HNF1 homeobox B
219753_at	10734	STAG3	stromal antigen 3
1562261_at	155185	AMZ1	archaelysin family metallopeptidase 1
242510_at	101928324	LOC101928324	uncharacterized LOC101928324
1559624_at	202374	STK32A	serine/threonine kinase 32A
237120_at	374454	KRT77	keratin 77
206398_s_at	930	CD19	CD19 molecule
1567055_at	26188	OR1C1	olfactory receptor, family 1, subfamily C, member 1
231583_at	121391	KRT74	keratin 74
1562256_at	22861	NLRP1	NLR family, pyrin domain containing 1
215758_x_at	81931	ZNF93	zinc finger protein 93
235802_at	122618	PLD4	phospholipase D family, member 4
207385_at	51270	TFDP3	transcription factor Dp family, member 3
220267_at	192666	KRT24	keratin 24
231767_at	3214	HOXB4	homeobox B4
221402_at	4992	OR1F1	olfactory receptor, family 1, subfamily F, member 1
1558603_at	5342	PLGLB2	plasminogen-like B2
1552576_at	200420	ALMS1P	Alstrom syndrome 1 pseudogene
1553642_at	158055	C9orf163	chromosome 9 open reading frame 163
1556507_at	100507274	LINC01210	long intergenic non-protein coding RNA 1210
1562934_at	101927460	LOC101927460	uncharacterized LOC101927460
237288_at	116179	TGM7	transglutaminase 7
232173_at	154790	CLEC2L	C-type lectin domain family 2, member L
1562857_at	101928417	LOC101928417	uncharacterized LOC101928417
227053_at	29993	PACSIN1	protein kinase C and casein kinase substrate in neurons 1
1562801_at	101927379	LOC101927379	uncharacterized LOC101927379
1564281_at	285708	LINC00491	long intergenic non-protein coding RNA 491
210060_at	5148	PDE6G	phosphodiesterase 6G, cGMP-specific, rod, gamma

207325_x_at	4100	MAGEA1	melanoma antigen family A, 1 (directs expression of antigen MZ2-E)
221271_at	59067	IL21	interleukin 21
1566776_at	25981	DNAH1	dynein, axonemal, heavy chain 1
1553574_at	338376	IFNE	interferon, epsilon
201998_at	6480	ST6GAL1	ST6 beta-galactosamide alpha-2,6-sialyltranferase 1
1555368_x_at	90827	ZNF479	zinc finger protein 479
1569775_at	114804	RNF157	ring finger protein 157
218677_at	57402	S100A14	S100 calcium binding protein A14
234231_at	197350	CASP16	caspase 16, apoptosis-related cysteine peptidase (putative)
206998_x_at	5544	PRB3	proline-rich protein BstNI subfamily 3
221209_s_at	56914	OTOR	otoraplin
1570015_at	149620	CHIAP2	chitinase, acidic pseudogene 2
227560_at	118980	SFXN2	sideroflexin 2
220282_at	79608	RIC3	RIC3 acetylcholine receptor chaperone
1569780_at	101928700	LOC101928700	uncharacterized LOC101928700
1562909_at	554279	LINC00862	long intergenic non-protein coding RNA 862
221601_s_at	9214	FAIM3	Fas apoptotic inhibitory molecule 3
244692_at	126410	CYP4F22	cytochrome P450, family 4, subfamily F, polypeptide 22
211771_s_at	5452	POU2F2	POU class 2 homeobox 2
232972_at	92340	PRR29	proline rich 29
1560830_a_at	147646	C19orf84	chromosome 19 open reading frame 84
1561512_at	101928861	LOC101928861	uncharacterized LOC101928861
234475_x_at	887	CCKBR	cholecystokinin B receptor
1557693_at	100507299	SMC5-AS1	SMC5 antisense RNA 1 (head to head)
207074_s_at	6570	SLC18A1	solute carrier family 18 (vesicular monoamine transporter), member 1
206658_at	80761	UPK3B	uroplakin 3B
219786_at	9633	MTL5	metallothionein-like 5, testis-specific (tesmin)
1552843_at	10861	SLC26A1	solute carrier family 26 (anion exchanger), member 1
227353_at	147138	TMC8	transmembrane channel-like 8
1557386_at	100507600	LOC100507600	uncharacterized LOC100507600
234469_at	79339	OR51B4	olfactory receptor, family 51, subfamily B, member 4
1553081_at	128488	WFDC12	WAP four-disulfide core domain 12
1558920_at	100128590	SLC8A1-AS1	SLC8A1 antisense RNA 1
207404_s_at	3354	HTR1E	5-hydroxytryptamine (serotonin) receptor 1E, G protein-coupled
216030_s_at	6407	SEMG2	semenogelin II
223820_at	83758	RBP5	retinol binding protein 5, cellular
1569855_at	101928306	LOC101928306	uncharacterized LOC101928306
1561232_at	100270680	CASC11	cancer susceptibility candidate 11 (non-protein coding)
234766_at	283160	OR8D2	olfactory receptor, family 8, subfamily D, member 2
237236_x_at	731789	LINC00202-2	long intergenic non-protein coding RNA 202-2
1562878_at	102723704	LOC102723704	uncharacterized LOC102723704
214222_at	56171	DNAH7	dynein, axonemal, heavy chain 7
1565771_at	283218	DKFZp434E1119	uncharacterized DKFZp434E1119
1567682_x_at	26821	SNORA74A	small nucleolar RNA, H/ACA box 74A
1556727_at	768206	PRCD	progressive rod-cone degeneration
224159_x_at	89122	TRIM4	tripartite motif containing 4
240719_at	100505536	ISM1-AS1	ISM1 antisense RNA 1
208247_at	711	ERC2-IT1	ERC2 intronic transcript 1 (non-protein coding)
223876_at	83893	SPATA16	spermatogenesis associated 16
230378_at	92304	SCGB3A1	secretoglobin, family 3A, member 1
1569807_at	101928778	LOC101928778	uncharacterized LOC101928778
230824_at	162333	MARCH10	membrane-associated ring finger (C3HC4) 10, E3 ubiquitin protein ligase
228979_at	253970	SFTA3	surfactant associated 3
235140_at	134549	SHROOM1	shroom family member 1
224124_at	84083	ZRANB3	zinc finger, RAN-binding domain containing 3
219651_at	55211	DPPA4	developmental pluripotency associated 4
216018_at	6048	RNF5	ring finger protein 5, E3 ubiquitin protein ligase
214652_at	1812	DRD1	dopamine receptor D1
1569677_a_at	157777	MCMDC2	minichromosome maintenance domain containing 2
1564639_at	100291323	LOC100291323	uncharacterized LOC100291323
1566526_at	283688	LINC00927	long intergenic non-protein coding RNA 927

1560119_at	389634	LINC00937	long intergenic non-protein coding RNA 937
233913_at	140832	WFDC10A	WAP four-disulfide core domain 10A
222965_at	55387	PRO2214	uncharacterized protein PRO2214
1553229_at	137209	ZNF572	zinc finger protein 572
204267_x_at	9088	PKMYT1	protein kinase, membrane associated tyrosine/threonine 1
1559226_x_at	353135	LCE1E	late cornified envelope 1E
221172_at	80099	C7orf69	chromosome 7 open reading frame 69
1553202_at	219736	STOX1	storkhead box 1
206485_at	921	CD5	CD5 molecule
216051_x_at	100169750	PRINS	psoriasis associated non-protein coding RNA induced by stress
1559754_at	4050	LTB	lymphotoxin beta (TNF superfamily, member 3)
221112_at	26280	IL1RAPL2	interleukin 1 receptor accessory protein-like 2
216299_s_at	7517	XRCC3	X-ray repair complementing defective repair in Chinese hamster cells 3
244565_at	3167	HMX2	H6 family homeobox 2
1555834_at	7345	UCHL1	ubiquitin carboxyl-terminal esterase L1 (ubiquitin thiolesterase)
222377_at	347853	TBX10	T-box 10
1560833_at	643648	LINC01189	long intergenic non-protein coding RNA 1189
1564338_at	101927257	LOC101927257	uncharacterized LOC101927257
204951_at	399	RHOH	ras homolog family member H
1552311_a_at	84839	RAX2	retina and anterior neural fold homeobox 2
1561402_at	339894	LINC00880	long intergenic non-protein coding RNA 880
210448_s_at	5026	P2RX5	purinergic receptor P2X, ligand-gated ion channel, 5
206589_at	2672	GFI1	growth factor independent 1 transcription repressor
216153_x_at	8434	RECK	reversion-inducing-cysteine-rich protein with kazal motifs
221397_at	50839	TAS2R10	taste receptor, type 2, member 10
233935_at	8740	TNFSF14	tumor necrosis factor (ligand) superfamily, member 14
1553258_at	146512	FLJ30679	uncharacterized protein FLJ30679
223748_at	83959	SLC4A11	solute carrier family 4, sodium borate transporter, member 11
220423_at	26279	PLA2G2D	phospholipase A2, group IID
1562604_at	101927043	LOC101927043	uncharacterized LOC101927043
218907_s_at	65999	LRRC61	leucine rich repeat containing 61
1553323_a_at	117155	CATSPER2	cation channel, sperm associated 2
208602_x_at	923	CD6	CD6 molecule
206760_s_at	2208	FCER2	Fc fragment of IgE, low affinity II, receptor for (CD23)
1553681_a_at	5551	PRF1	perforin 1 (pore forming protein)
1562831_a_at	283089	WDR11-AS1	WDR11 antisense RNA 1
223670_s_at	55363	HEMGN	hemogen
1554129_a_at	149685	ADIG	adipogenin
1565846_at	493754	LOC493754	RAB guanine nucleotide exchange factor (GEF) 1 pseudogene
216935_at	388699	LINC00302	long intergenic non-protein coding RNA 302
234133_s_at	89894	TMEM116	transmembrane protein 116
210934_at	640	BLK	BLK proto-oncogene, Src family tyrosine kinase
215356_at	91646	TDRD12	tudor domain containing 12
231196_x_at	387644	LINC00202-1	long intergenic non-protein coding RNA 202-1
1569674_at	100287765	LINC00630	long intergenic non-protein coding RNA 630
1556249_a_at	440952	EIF1B-AS1	EIF1B antisense RNA 1
220626_at	51156	SERPINA10	serpin peptidase inhibitor, clade A (alpha-1 antiproteinase, antitrypsin), member 10
241694_at	5314	PKHD1	polycystic kidney and hepatic disease 1 (autosomal recessive)
210858_x_at	472	ATM	ATM serine/threonine kinase
215015_at	92558	CCDC64	coiled-coil domain containing 64
1557498_a_at	101928190	LINC01487	long intergenic non-protein coding RNA 1487
222307_at	282997	PDCD4-AS1	PDCD4 antisense RNA 1
208217_at	2570	GABRR2	gamma-aminobutyric acid (GABA) A receptor, rho 2
1561688_at	101929662	LOC101929662	uncharacterized LOC101929662
233997_at	100287166	LOC100287166	uncharacterized LOC100287166
1553196_a_at	115352	FCRL3	Fc receptor-like 3
1556099_at	728081	LINC00290	long intergenic non-protein coding RNA 290
232195_at	57512	GPR158	G protein-coupled receptor 158
1554394_at	4745	NELL1	NEL-like 1 (chicken)
1560198_at	283601	LINC00523	long intergenic non-protein coding RNA 523
236739_at	150622	LINC01105	long intergenic non-protein coding RNA 1105

233960_s_at	115110	LOC115110	uncharacterized LOC115110
1563117_at	388456	LOC388456	uncharacterized LOC388456
208008_at	26083	TBC1D29	TBC1 domain family, member 29
211132_at	65123	INTS3	integrator complex subunit 3
230488_s_at	138948	DBH-AS1	DBH antisense RNA 1
1567257_at	26740	OR1J2	olfactory receptor, family 1, subfamily J, member 2
237320_at	346653	FAM71F2	family with sequence similarity 71, member F2
206713_at	22854	NTNG1	netrin G1
227218_at	146206	RLTPR	RGD motif, leucine rich repeats, tropomodulin domain and proline-rich containing
1560897_a_at	386678	KRTAP10-11	keratin associated protein 10-11
210827_s_at	1999	ELF3	E74-like factor 3 (ets domain transcription factor, epithelial-specific)
1556793_a_at	128876	FAM83C	family with sequence similarity 83, member C
220431_at	28983	TMPRSS11E	transmembrane protease, serine 11E
1554246_at	149466	C1orf210	chromosome 1 open reading frame 210
1563802_at	284551	LINC01226	long intergenic non-protein coding RNA 1226
1562691_at	101927075	FGF10-AS1	FGF10 antisense RNA 1
220169_at	80008	TMEM156	transmembrane protein 156
208259_x_at	3444	IFNA7	interferon, alpha 7
1561105_at	5010	CLDN11	claudin 11
210313_at	23547	LILRA4	leukocyte immunoglobulin-like receptor, subfamily A (with TM domain), member 4
215797_at	28683	TRAV8-3	T cell receptor alpha variable 8-3
203713_s_at	3993	LLGL2	lethal giant larvae homolog 2 (Drosophila)
204953_at	9892	SNAP91	synaptosomal-associated protein, 91kDa
222194_at	100132923	FAM66D	family with sequence similarity 66, member D
204138_s_at	7593	MZF1	myeloid zinc finger 1
1555265_at	150000	ABCC13	ATP-binding cassette, sub-family C (CFTR/MRP), member 13, pseudogene
1563086_at	101928894	LOC101928894	uncharacterized LOC101928894
234648_s_at	55998	NXF5	nuclear RNA export factor 5
207009_at	8929	PHOX2B	paired-like homeobox 2b
206520_x_at	946	SIGLEC6	sialic acid binding Ig-like lectin 6
206609_at	9947	MAGEC1	melanoma antigen family C, 1
231264_at	127391	TMCO2	transmembrane and coiled-coil domains 2
220045_at	63974	NEUROD6	neuronal differentiation 6
1561579_at	728445	LOC728445	uncharacterized LOC728445
221182_at	80133	MROH9	maestro heat-like repeat family member 9
217133_x_at	1555	CYP2B6	cytochrome P450, family 2, subfamily B, polypeptide 6
223514_at	84433	CARD11	caspase recruitment domain family, member 11
241990_at	171177	RHOV	ras homolog family member V
220634_at	9496	TBX4	T-box 4
219118_at	51303	FKBP11	FK506 binding protein 11, 19 kDa
206478_at	9834	KIAA0125	KIAA0125
226226_at	120224	TMEM45B	transmembrane protein 45B
1563488_at	114795	TMEM132B	transmembrane protein 132B
231998_at	9092	SART1	squamous cell carcinoma antigen recognized by T cells
211438_at	7201	TRHR	thyrotropin-releasing hormone receptor
233429_at	79925	SPEF2	sperm flagellar 2
236642_at	401207	C5orf63	chromosome 5 open reading frame 63
244518_at	100130452	LOC100130452	uncharacterized LOC100130452
1569614_s_at	138307	LCN8	lipocalin 8
221392_at	50832	TAS2R4	taste receptor, type 2, member 4
1561387_a_at	120400	NXPE1	neurexophilin and PC-esterase domain family, member 1
220384_at	51314	NME8	NME/NM23 family member 8
1561226_at	2829	XCR1	chemokine (C motif) receptor 1
220286_at	54893	MTMR10	myotubularin related protein 10
204563_at	6402	SELL	selectin L
1562038_at	101928694	LOC101928694	uncharacterized LOC101928694
207185_at	6554	SLC10A1	solute carrier family 10 (sodium/bile acid cotransporter), member 1
1563318_s_at	79917	MAGIX	MAGI family member, X-linked
1568593_a_at	152195	NUDT16P1	nudix (nucleoside diphosphate linked moiety X)-type motif 16 pseudogene 1
1564584_at	100506557	LOC100506557	uncharacterized LOC100506557

224053_s_at	83697	SLC4A9	solute carrier family 4, sodium bicarbonate cotransporter, member 9
1557450_s_at	440253	WHAMMP2	WAS protein homolog associated with actin, golgi membranes and microtubules pseudogene 2
1564220_a_at	100506465	LINC01234	long intergenic non-protein coding RNA 1234
204411_at	23046	KIF21B	kinesin family member 21B
1570206_at	101928797	LOC101928797	uncharacterized LOC101928797
1568897_at	100422212	LOC100422212	eukaryotic translation initiation factor 3, subunit J pseudogene
238721_at	130752	MDH1B	malate dehydrogenase 1B, NAD (soluble)
1552524_at	116969	ART5	ADP-ribosyltransferase 5
235998_at	114822	RHPN1	rhophilin, Rho GTPase binding protein 1
1557451_at	26222	DGCR10	DiGeorge syndrome critical region gene 10 (non-protein coding)
229229_at	64902	AGXT2	alanine--glyoxylate aminotransferase 2
206045_s_at	8715	NOL4	nucleolar protein 4
211427_s_at	3769	KCNJ13	potassium inwardly-rectifying channel, subfamily J, member 13
1556516_at	100506331	LOC100506331	uncharacterized LOC100506331
208376_at	1233	CCR4	chemokine (C-C motif) receptor 4
220506_at	2974	GUCY1B2	guanylate cyclase 1, soluble, beta 2 (pseudogene)
231036_at	100505774	LOC100505774	uncharacterized LOC100505774
233158_at	3888	KRT82	keratin 82
1562984_at	101928937	LOC101928937	uncharacterized LOC101928937
1567242_at	26247	OR2L1P	olfactory receptor, family 2, subfamily L, member 1 pseudogene
1564679_at	142685	ASB15	ankyrin repeat and SOCS box containing 15
1554662_at	284083	C17orf47	chromosome 17 open reading frame 47
234141_s_at	286059	LOC286059	tumor necrosis factor receptor superfamily, member 10d, decoy with truncated death domain pseudogene
214341_at	8906	AP1G2	adaptor-related protein complex 1, gamma 2 subunit
211223_at	5626	PROP1	PROP paired-like homeobox 1
1561559_at	101927948	LOC101927948	uncharacterized LOC101927948
207490_at	80086	TUBA4B	tubulin, alpha 4b (pseudogene)
1556914_at	100652911	LOC100652911	uncharacterized LOC100652911
232076_at	286075	ZNF707	zinc finger protein 707
207209_at	1068	CETN1	centrin, EF-hand protein, 1
211145_x_at	3452	IFNA21	interferon, alpha 21
207372_s_at	954	ENTPD2	ectonucleoside triphosphate diphosphohydrolase 2
235816_s_at	266747	RGL4	ral guanine nucleotide dissociation stimulator-like 4
1552386_at	202309	GAPT	GRB2-binding adaptor protein, transmembrane
206740_x_at	6847	SYCP1	synaptonemal complex protein 1
1563827_at	158434	LOC158434	uncharacterized LOC158434
1569974_x_at	641977	SEPT7P2	septin 7 pseudogene 2
239656_at	723809	LHFPL3-AS2	LHFPL3 antisense RNA 2
207466_at	51083	GAL	galanin/GMAP prepropeptide
1555662_s_at	267012	DAOA	D-amino acid oxidase activator
223536_at	84249	PSD2	pleckstrin and Sec7 domain containing 2
207919_at	417	ART1	ADP-ribosyltransferase 1
1559870_at	100129129	LOC100129129	uncharacterized LOC100129129
1560288_at	101927196	LOC101927196	uncharacterized LOC101927196
216276_s_at	1587	ADAM3A	ADAM metallopeptidase domain 3A (pseudogene)
1561423_at	642924	LINC00535	long intergenic non-protein coding RNA 535
208558_at	26539	OR10H1	olfactory receptor, family 10, subfamily H, member 1
1562320_at	100874012	NAV2-AS5	NAV2 antisense RNA 5
207670_at	3891	KRT85	keratin 85
207777_s_at	11262	SP140	SP140 nuclear body protein
1563484_at	101927766	LOC101927766	uncharacterized LOC101927766
1562391_at	148789	B3GALNT2	beta-1,3-N-acetylgalactosaminyltransferase 2
232359_at	51109	RDH11	retinol dehydrogenase 11 (all-trans/9-cis/11-cis)
1570241_at	374955	SPATA21	spermatogenesis associated 21
1564559_at	728073	LOC728073	uncharacterized LOC728073
229686_at	286530	P2RY8	purinergic receptor P2Y, G-protein coupled, 8
1552882_a_at	139285	AMER1	APC membrane recruitment protein 1
224137_at	59284	CACNG7	calcium channel, voltage-dependent, gamma subunit 7
1569270_at	100134368	LOC100134368	uncharacterized LOC100134368
1553901_x_at	90649	ZNF486	zinc finger protein 486

1560412_at	100507506	LOC100507506	uncharacterized LOC100507506
228298_at	91523	PCED1B	PC-esterase domain containing 1B
1557843_at	101927994	LINC01087	long intergenic non-protein coding RNA 1087
1552526_at	196472	FAM71C	family with sequence similarity 71, member C
1553061_at	89883	OR6W1P	olfactory receptor, family 6, subfamily W, member 1 pseudogene
232680_at	154150	HDGFL1	hepatoma derived growth factor-like 1
208019_at	7712	ZNF157	zinc finger protein 157
220421_at	79908	BTNL8	butyrophilin-like 8
221293_s_at	50619	DEF6	differentially expressed in FDCP 6 homolog (mouse)
1562908_at	339468	LOC339468	uncharacterized LOC339468
228844_at	284111	SLC13A5	solute carrier family 13 (sodium-dependent citrate transporter), member 5
205692_s_at	952	CD38	CD38 molecule
206997_s_at	9394	HS6ST1	heparan sulfate 6-O-sulfotransferase 1
206988_at	6370	CCL25	chemokine (C-C motif) ligand 25
1553857_at	283284	IGSF22	immunoglobulin superfamily, member 22
211225_at	2527	FUT5	fucosyltransferase 5 (alpha (1,3) fucosyltransferase)
207735_at	54941	RNF125	ring finger protein 125, E3 ubiquitin protein ligase
1562455_at	101929586	LOC101929586	uncharacterized LOC101929586
216981_x_at	6693	SPN	sialophorin
210884_s_at	653423	SPAG11A	sperm associated antigen 11A
243699_at	100507006	LOC100507006	uncharacterized LOC100507006
204988_at	2244	FGB	fibrinogen beta chain
1556267_at	196446	MYRFL	myelin regulatory factor-like
1553614_a_at	100128202	FLJ25694	uncharacterized protein FLJ25694
1564713_a_at	121643	FOXN4	forkhead box N4
214515_at	8387	OR1E1	olfactory receptor, family 1, subfamily E, member 1
1569828_at	101928107	LOC101928107	uncharacterized LOC101928107
1564402_at	146795	LOC146795	uncharacterized LOC146795
224586_x_at	10923	SUB1	SUB1 homolog (S. cerevisiae)
224125_at	84069	PLEKHN1	pleckstrin homology domain containing, family N member 1
1560380_at	497256	LOC497256	uncharacterized LOC497256
242364_x_at	100131096	TNRC6C-AS1	TNRC6C antisense RNA 1
240692_at	414927	MGC34796	sepiapterin reductase (7,8-dihydrobiopterin:NADP+ oxidoreductase) pseudogene
1557474_at	284578	LOC284578	uncharacterized LOC284578
227189_at	57699	CPNE5	copine V
1565786_x_at	645566	FLJ45482	uncharacterized LOC645566
210957_s_at	2334	AFF2	AF4/FMR2 family, member 2
220112_at	79722	ANKRD55	ankyrin repeat domain 55
1554616_at	5271	SERPINB8	serpin peptidase inhibitor, clade B (ovalbumin), member 8
1553165_at	11276	SYNRG	synergin, gamma
240974_at	100506851	LOC100506851	uncharacterized LOC100506851
207725_at	5458	POU4F2	POU class 4 homeobox 2
1553125_x_at	284403	WDR62	WD repeat domain 62
210263_at	3754	KCNF1	potassium voltage-gated channel, subfamily F, member 1
237414_at	2155	F7	coagulation factor VII (serum prothrombin conversion accelerator)
229976_at	254956	MORN5	MORN repeat containing 5
215785_s_at	26999	CYFIP2	cytoplasmic FMR1 interacting protein 2
206323_x_at	4983	OPHN1	oligophrenin 1
1568634_a_at	339977	LRRC66	leucine rich repeat containing 66
211201_at	2492	FSHR	follicle stimulating hormone receptor
229252_at	285973	ATG9B	autophagy related 9B
1552761_at	162515	SLC16A11	solute carrier family 16, member 11
213669_at	23149	FCHO1	FCH domain only 1
1564314_at	219690	LOC219690	uncharacterized LOC219690
204960_at	5790	PTPRCAP	protein tyrosine phosphatase, receptor type, C-associated protein
206207_at	1178	CLC	Charcot-Leyden crystal galectin
1562972_at	503519	LINC00929	long intergenic non-protein coding RNA 929
1563107_at	101927843	LOC101927843	uncharacterized LOC101927843
206000_at	4224	MEP1A	meprin A, alpha (PABA peptide hydrolase)
206761_at	10225	CD96	CD96 molecule

221672_s_at	83696	TRAPPC9	trafficking protein particle complex 9
1564193_at	100652824	LOC100652824	uncharacterized protein KIAA2012
210036_s_at	3757	KCNH2	potassium voltage-gated channel, subfamily H (eag-related), member 2
216356_x_at	8938	BAIAP3	BAI1-associated protein 3
207392_x_at	7366	UGT2B15	UDP glucuronosyltransferase 2 family, polypeptide B15
1557359_at	285758	LINC01268	long intergenic non-protein coding RNA 1268
1560636_a_at	100286989	HSPC081	uncharacterized LOC100286989
207504_at	766	CA7	carbonic anhydrase VII
1563132_at	102723165	LINC01169	long intergenic non-protein coding RNA 1169
1560246_at	101929524	LOC101929524	uncharacterized LOC101929524
205255_x_at	6932	TCF7	transcription factor 7 (T-cell specific, HMG-box)
1559648_at	100128420	LINC00892	long intergenic non-protein coding RNA 892
1553805_at	132200	C3orf49	chromosome 3 open reading frame 49
206974_at	10663	CXCR6	chemokine (C-X-C motif) receptor 6
1553934_at	221241	LINC00305	long intergenic non-protein coding RNA 305
1569741_at	101927900	LINC01477	long intergenic non-protein coding RNA 1477
219702_at	10761	PLAC1	placenta-specific 1
233054_at	4848	CNOT2	CCR4-NOT transcription complex, subunit 2
221458_at	3355	HTR1F	5-hydroxytryptamine (serotonin) receptor 1F, G protein-coupled
1562930_at	100128788	SRRM2-AS1	SRRM2 antisense RNA 1
205831_at	914	CD2	CD2 molecule
1555580_at	101927774	LINC00824	long intergenic non-protein coding RNA 824
1562020_s_at	284958	NT5DC4	5'-nucleotidase domain containing 4
230115_at	374387	DKFZp779M0652	uncharacterized DKFZp779M0652
207333_at	4829	NMBR	neuromedin B receptor
232158_x_at	152519	NIPAL1	NIPA-like domain containing 1
1555645_at	100130322	GAFA2	FGF-2 activity-associated protein 2
1564855_at	101927079	LOC101927079	uncharacterized LOC101927079
208268_at	10863	ADAM28	ADAM metallopeptidase domain 28
1554953_a_at	114043	C21orf90	chromosome 21 open reading frame 90
204260_at	1114	CHGB	chromogranin B (secretogranin 1)
233657_at	221391	OPN5	opsin 5
1555188_at	100874107	MTUS2-AS1	MTUS2 antisense RNA 1
213534_s_at	23178	PASK	PAS domain containing serine/threonine kinase
231661_at	130120	REG3G	regenerating islet-derived 3 gamma
1558431_at	283948	NHLRC4	NHL repeat containing 4
207149_at	1010	CDH12	cadherin 12, type 2 (N-cadherin 2)
1564490_at	100128830	LINC01165	long intergenic non-protein coding RNA 1165
206290_s_at	6000	RGS7	regulator of G-protein signaling 7
1560928_at	151657	LOC151657	uncharacterized LOC151657
234272_at	100653005	LOC100653005	uncharacterized LOC100653005
229451_at	50614	GALNT9	polypeptide N-acetylgalactosaminyltransferase 9
220776_at	3770	KCNJ14	potassium inwardly-rectifying channel, subfamily J, member 14
243153_at	55755	CDK5RAP2	CDK5 regulatory subunit associated protein 2
223807_at	3547	IGSF1	immunoglobulin superfamily, member 1
1555000_at	100131077	OK/SW-CL.36	OK/SW-CL.36
1553464_at	286023	FLJ40288	uncharacterized FLJ40288
219277_s_at	55753	OGDHL	oxoglutarate dehydrogenase-like
1554831_x_at	151254	ALS2CR11	amyotrophic lateral sclerosis 2 (juvenile) chromosome region, candidate 11
235116_at	7185	TRAF1	TNF receptor-associated factor 1
237385_at	100506126	LINC00867	long intergenic non-protein coding RNA 867
207476_at	100507630	LOC100507630	uncharacterized LOC100507630
1559427_at	23263	MCF2L	MCF.2 cell line derived transforming sequence-like
1563188_at	102723448	LOC102723448	uncharacterized LOC102723448
211697_x_at	56902	PNO1	partner of NOB1 homolog (S. cerevisiae)
1563868_a_at	286234	SPATA31E1	SPATA31 subfamily E, member 1
1559303_at	100505835	LINC01532	long intergenic non-protein coding RNA 1532
1556459_at	100689073	ARHGAP22-IT1	ARHGAP22 intronic transcript 1 (non-protein coding)
1564595_at	101927360	LINC01103	long intergenic non-protein coding RNA 1103
210354_at	3458	IFNG	interferon, gamma
1569961_at	101928844	LOC101928844	uncharacterized LOC101928844

1561102_at	101929488	LOC101929488	uncharacterized LOC101929488
1556755_s_at	286149	LOC286149	uncharacterized LOC286149
219877_at	79698	ZMAT4	zinc finger, matrin-type 4
220418_at	53347	UBASH3A	ubiquitin associated and SH3 domain containing A
207248_at	3739	KCNA4	potassium voltage-gated channel, shaker-related subfamily, member 4
214536_at	57152	SLURP1	secreted LY6/PLAUR domain containing 1
1567060_at	26494	OR8G1	olfactory receptor, family 8, subfamily G, member 1
1569212_at	619207	SCART1	scavenger receptor protein family member
234861_at	93463	LOC93463	uncharacterized LOC93463
202524_s_at	9806	SPOCK2	sparc/osteonectin, cwcv and kazal-like domains proteoglycan (testican) 2
215765_at	10489	LRRC41	leucine rich repeat containing 41
206333_at	4440	MSI1	musashi RNA-binding protein 1
1560707_at	283856	LOC283856	uncharacterized LOC283856
1554396_at	55293	UEVLD	UEV and lactate/malate dehyrogenase domains
206828_at	7294	TXK	TXK tyrosine kinase
1556835_s_at	100652770	LOC100652770	uncharacterized LOC100652770
1563327_a_at	724087	CXorf31	chromosome X open reading frame 31
211338_at	3440	IFNA2	interferon, alpha 2
1564400_at	284274	SMIM21	small integral membrane protein 21
243592_at	51455	REV1	REV1, polymerase (DNA directed)
1564960_at	337878	KRTAP7-1	keratin associated protein 7-1 (gene/pseudogene)
211617_at	228	ALDOAP2	aldolase A, fructose-bisphosphate pseudogene 2
227711_at	121355	GTSF1	gametocyte specific factor 1
216993_s_at	1302	COL11A2	collagen, type XI, alpha 2
1562173_a_at	100861543	LINC00555	long intergenic non-protein coding RNA 555
229195_at	55897	MESP1	mesoderm posterior basic helix-loop-helix transcription factor 1
1560282_at	101928590	LOC101928590	uncharacterized LOC101928590
207019_s_at	8852	AKAP4	A kinase (PRKA) anchor protein 4
1570110_at	101928730	LOC101928730	uncharacterized LOC101928730
230753_at	197135	PATL2	protein associated with topoisomerase II homolog 2 (yeast)
210538_s_at	330	BIRC3	baculoviral IAP repeat containing 3
1552440_at	11318	GPR182	G protein-coupled receptor 182
57540_at	64080	RBKS	ribokinase
229561_at	90668	LRRC16B	leucine rich repeat containing 16B
205549_at	5121	PCP4	Purkinje cell protein 4
211597_s_at	84525	HOPX	HOP homeobox
1560531_at	353132	LCE1B	late cornified envelope 1B
227598_at	113763	ZBED6CL	ZBED6 C-terminal like
1561732_at	101929181	LOC101929181	uncharacterized LOC101929181
223783_s_at	100131454	DBIL5P	diazepam binding inhibitor-like 5, pseudogene
1565611_at	83658	DYNLRB1	dynein, light chain, roadblock-type 1
227224_at	55103	RALGPS2	Ral GEF with PH domain and SH3 binding motif 2
233837_at	2678	GGT1	gamma-glutamyltransferase 1
207420_at	10584	COLEC10	collectin sub-family member 10 (C-type lectin)
38149_at	9938	ARHGAP25	Rho GTPase activating protein 25
240316_at	138240	C9orf57	chromosome 9 open reading frame 57
1561651_s_at	6886	TAL1	T-cell acute lymphocytic leukemia 1
238095_at	140628	GATA5	GATA binding protein 5
1561619_at	101929478	LOC101929478	uncharacterized LOC101929478
244512_at	100874362	HOXB-AS1	HOXB cluster antisense RNA 1
1553544_at	83550	GPR101	G protein-coupled receptor 101
208359_s_at	3761	KCNJ4	potassium inwardly-rectifying channel, subfamily J, member 4
1554970_at	204474	PDILT	protein disulfide isomerase-like, testis expressed
213874_at	5267	SERPINA4	serpin peptidase inhibitor, clade A (alpha-1 antiproteinase, antitrypsin), member 4
211005_at	27040	LAT	linker for activation of T cells
1560472_at	338588	LINC00705	long intergenic non-protein coding RNA 705
220577_at	387751	GVINP1	GTPase, very large interferon inducible pseudogene 1
243961_at	100505622	LOC100505622	uncharacterized LOC100505622
219466_s_at	336	APOA2	apolipoprotein A-II

210604_at	2780	GNAT2	guanine nucleotide binding protein (G protein), alpha transducing activity polypeptide 2
207634_at	5133	PDCD1	programmed cell death 1
1569786_at	101929694	LOC101929694	uncharacterized LOC101929694
222895_s_at	64919	BCL11B	B-cell CLL/lymphoma 11B (zinc finger protein)
1568754_at	101927814	LOC101927814	uncharacterized LOC101927814
211585_at	4863	NPAT	nuclear protein, ataxia-telangiectasia locus
210272_at	1556	CYP2B7P	cytochrome P450, family 2, subfamily B, polypeptide 7, pseudogene
233077_at	399876	NAV2-AS4	NAV2 antisense RNA 4
1553484_at	144360	LINC00477	long intergenic non-protein coding RNA 477
207267_s_at	53820	RIPPLY3	ripply transcriptional repressor 3
236302_at	22843	PPM1E	protein phosphatase, Mg2+/Mn2+ dependent, 1E
232577_at	145945	LOC145945	uncharacterized LOC145945
237254_at	115584	SLC5A11	solute carrier family 5 (sodium/inositol cotransporter), member 11
214407_x_at	2994	GYPB	glycophorin B (MNS blood group)
1555401_at	54937	SOHLH2	spermatogenesis and oogenesis specific basic helix-loop-helix 2
235358_at	728485	LOC728485	uncharacterized LOC728485
215725_at	25786	DGCR11	DiGeorge syndrome critical region gene 11 (non-protein coding)
221150_at	56955	MEPE	matrix extracellular phosphoglycoprotein
236119_s_at	6706	SPRR2G	small proline-rich protein 2G
221630_s_at	54514	DDX4	DEAD (Asp-Glu-Ala-Asp) box polypeptide 4
1555273_at	442117	GALNTL6	polypeptide N-acetylgalactosaminyltransferase-like 6
233975_at	149830	PRNT	prion protein (testis specific)
207811_at	3859	KRT12	keratin 12
232368_at	100128327	TRAPPC3L	trafficking protein particle complex 3-like
1563572_at	152274	LOC152274	uncharacterized LOC152274
214973_x_at	3495	IGHD	immunoglobulin heavy constant delta
1570273_at	101927907	LOC101927907	uncharacterized LOC101927907
1561391_at	100128126	STAU2-AS1	STAU2 antisense RNA 1
241512_at	375686	SPATC1	spermatogenesis and centriole associated 1
234626_at	390063	OR51I1	olfactory receptor, family 51, subfamily I, member 1
207020_at	11077	HSF2BP	heat shock transcription factor 2 binding protein
219463_at	24141	LAMP5	lysosomal-associated membrane protein family, member 5
235650_at	222256	CDHR3	cadherin-related family member 3
1556454_a_at	100506274	LOC100506274	uncharacterized LOC100506274
1562870_at	101929517	LOC101929517	uncharacterized LOC101929517
228436_at	3749	KCNC4	potassium voltage-gated channel, Shaw-related subfamily, member 4
1555613_a_at	7535	ZAP70	zeta-chain (TCR) associated protein kinase 70kDa
1569935_at	144348	ZNF664	zinc finger protein 664
201004_at	6748	SSR4	signal sequence receptor, delta
221451_s_at	26692	OR2W1	olfactory receptor, family 2, subfamily W, member 1
1569086_at	100287177	C19orf83	chromosome 19 open reading frame 83
1563079_at	100289090	LOC100289090	uncharacterized LOC100289090
241367_at	400629	TEX19	testis expressed 19
219359_at	80162	ATHL1	ATH1, acid trehalase-like 1 (yeast)
1553013_at	129684	CNTNAP5	contactin associated protein-like 5
238205_at	139170	DCAF12L1	DDB1 and CUL4 associated factor 12-like 1
1555396_s_at	340602	CXorf67	chromosome X open reading frame 67
207694_at	5456	POU3F4	POU class 3 homeobox 4
232042_at	94015	TTYH2	tweety family member 2
221395_at	50838	TAS2R13	taste receptor, type 2, member 13
1561251_at	285577	LINC01019	long intergenic non-protein coding RNA 1019
237635_at	100128164	LOC100128164	four and a half LIM domains 1 pseudogene
234671_at	85291	KRTAP4-2	keratin associated protein 4-2
243439_at	147686	ZNF418	zinc finger protein 418
206406_at	4184	SMCP	sperm mitochondria-associated cysteine-rich protein
232593_at	93082	NEURL3	neuralized E3 ubiquitin protein ligase 3
62987_r_at	27092	CACNG4	calcium channel, voltage-dependent, gamma subunit 4
209992_at	5208	PFKFB2	6-phosphofructo-2-kinase/fructose-2,6-biphosphatase 2
206784_at	343	AQP8	aquaporin 8
200670_at	7494	XBP1	X-box binding protein 1
1558820_a_at	374864	CCDC178	coiled-coil domain containing 178

214529_at	7252	TSHB	thyroid stimulating hormone, beta
206280_at	1016	CDH18	cadherin 18, type 2
236658_at	100861523	RPS6KA2-AS1	RPS6KA2 antisense RNA 1
1561545_at	100874018	EPN2-AS1	EPN2 antisense RNA 1
229294_at	57338	JPH3	junctophilin 3
236421_at	339416	ANKRD45	ankyrin repeat domain 45
230819_at	126567	C2CD4C	C2 calcium-dependent domain containing 4C
205320_at	10297	APC2	adenomatosis polyposis coli 2
205852_at	8941	CDK5R2	cyclin-dependent kinase 5, regulatory subunit 2 (p39)
215970_at	100130331	LOC100130331	POTE ankyrin domain family, member F pseudogene
1553367_a_at	125965	COX6B2	cytochrome c oxidase subunit VIb polypeptide 2 (testis)
1564838_a_at	151760	LOC151760	uncharacterized LOC151760
205949_at	759	CA1	carbonic anhydrase I
1566656_a_at	100507654	LOC100507654	uncharacterized LOC100507654
1556014_at	145873	MESP2	mesoderm posterior basic helix-loop-helix transcription factor 2
1561290_at	339622	LOC339622	uncharacterized LOC339622
224134_at	84757	MGC10814	uncharacterized protein MGC10814
1562130_at	79781	IQCA1	IQ motif containing with AAA domain 1
1561671_at	286121	LOC286121	uncharacterized LOC286121
205747_at	869	CBLN1	cerebellin 1 precursor
237909_at	8755	ADAM6	ADAM metallopeptidase domain 6, pseudogene
236497_at	729683	LOC729683	uncharacterized LOC729683
222223_s_at	26525	IL36RN	interleukin 36 receptor antagonist
224273_at	84077	C3orf20	chromosome 3 open reading frame 20
221362_at	3361	HTR5A	5-hydroxytryptamine (serotonin) receptor 5A, G protein-coupled
1554371_at	114780	PKD1L2	polycystic kidney disease 1-like 2
223663_at	283234	CCDC88B	coiled-coil domain containing 88B
207615_s_at	750	C16orf3	chromosome 16 open reading frame 3
1558530_at	654429	LRTM2	leucine-rich repeats and transmembrane domains 2
221236_s_at	81551	STMN4	stathmin-like 4
229469_at	9502	XAGE2	X antigen family, member 2
207581_s_at	4115	MAGEB4	melanoma antigen family B, 4
207608_x_at	1544	CYP1A2	cytochrome P450, family 1, subfamily A, polypeptide 2
243265_at	2572	GAD2	glutamate decarboxylase 2 (pancreatic islets and brain, 65kDa)
214612_x_at	4105	MAGEA6	melanoma antigen family A, 6
239359_at	441061	MARCH11	membrane-associated ring finger (C3HC4) 11
211147_s_at	9127	P2RX6	purinergic receptor P2X, ligand-gated ion channel, 6
1570155_at	101926978	LINC01111	long intergenic non-protein coding RNA 1111
220481_at	10936	GPR75	G protein-coupled receptor 75
220307_at	51744	CD244	CD244 molecule, natural killer cell receptor 2B4
221198_at	6343	SCT	secretin
1553079_at	135644	TRIM40	tripartite motif containing 40
226564_at	57623	ZFAT	zinc finger and AT hook domain containing
1563005_at	101929441	LOC101929441	uncharacterized LOC101929441
232623_at	100128751	LOC100128751	INM04
228099_at	162972	ZNF550	zinc finger protein 550
1562319_at	283777	FAM169B	family with sequence similarity 169, member B
215420_at	3549	IHH	indian hedgehog
207651_at	29909	GPR171	G protein-coupled receptor 171
1558212_at	401491	VLDLR-AS1	VLDLR antisense RNA 1
1559170_at	440482	ANKRD20A5P	ankyrin repeat domain 20 family, member A5, pseudogene
205242_at	10563	CXCL13	chemokine (C-X-C motif) ligand 13
1562778_at	401565	FAM166A	family with sequence similarity 166, member A
220910_at	80144	FRAS1	Fraser extracellular matrix complex subunit 1
235445_at	100508046	LOC100508046	uncharacterized LOC100508046
205924_at	5865	RAB3B	RAB3B, member RAS oncogene family
215186_at	23329	TBC1D30	TBC1 domain family, member 30
1560713_a_at	114794	ELFN2	extracellular leucine-rich repeat and fibronectin type III domain containing 2
237765_at	283600	SLC25A47	solute carrier family 25, member 47
208274_at	10896	OCLM	oculomedin
211768_at	7462	LAT2	linker for activation of T cells family, member 2
220883_at	55478	PRO2012	uncharacterized protein PRO2012

244716_x_at	126259	TMIGD2	transmembrane and immunoglobulin domain containing 2
234687_x_at	8994	LIMD1	LIM domains containing 1
220015_at	54897	CASZ1	castor zinc finger 1
227393_at	338440	ANO9	anoctamin 9
1553062_at	116255	MOGAT1	monoacylglycerol O-acyltransferase 1
202015_x_at	10988	METAP2	methionyl aminopeptidase 2
237995_at	23504	RIMBP2	RIMS binding protein 2
208484_at	3024	HIST1H1A	histone cluster 1, H1a
215480_at	57242	KIAA0509	uncharacterized LOC57242
220830_at	50939	IMPG2	interphotoreceptor matrix proteoglycan 2
201094_at	6235	RPS29	ribosomal protein S29
1554187_at	554206	LOC554206	leucine carboxyl methyltransferase 1 pseudogene
1553560_at	317703	VN1R4	vomeronasal 1 receptor 4
230717_at	286256	LCN12	lipocalin 12
206336_at	6372	CXCL6	chemokine (C-X-C motif) ligand 6
242020_s_at	81030	ZBP1	Z-DNA binding protein 1
1563854_s_at	283045	LOC283045	uncharacterized LOC283045
242473_at	9618	TRAF4	TNF receptor-associated factor 4
1556232_at	221458	KIF6	kinesin family member 6
1561197_at	442028	LOC442028	uncharacterized LOC442028
1570121_at	22891	ZNF365	zinc finger protein 365
1557591_at	283038	LOC283038	uncharacterized LOC283038
206751_s_at	9468	PCYT1B	phosphate cytidylyltransferase 1, choline, beta
1569954_at	101928834	LOC101928834	uncharacterized LOC101928834
203332_s_at	3635	INPP5D	inositol polyphosphate-5-phosphatase, 145kDa
1557008_at	340107	LOC340107	uncharacterized LOC340107
205939_at	1551	CYP3A7	cytochrome P450, family 3, subfamily A, polypeptide 7
207354_at	6360	CCL16	chemokine (C-C motif) ligand 16
1562030_at	284898	LOC284898	uncharacterized LOC284898
215672_s_at	23382	AHCYL2	adenosylhomocysteinase-like 2
207861_at	6367	CCL22	chemokine (C-C motif) ligand 22
1568812_at	100507140	LOC100507140	uncharacterized LOC100507140
206641_at	608	TNFRSF17	tumor necrosis factor receptor superfamily, member 17
226132_s_at	149175	MANEAL	mannosidase, endo-alpha-like
221054_s_at	27004	TCL6	T-cell leukemia/lymphoma 6 (non-protein coding)
209942_x_at	4102	MAGEA3	melanoma antigen family A, 3
219954_s_at	57733	GBA3	glucosidase, beta, acid 3
227845_s_at	56961	SHD	Src homology 2 domain containing transforming protein D
207228_at	5568	PRKACG	protein kinase, cAMP-dependent, catalytic, gamma
1553555_at	5726	TAS2R38	taste receptor, type 2, member 38
229725_at	23305	ACSL6	acyl-CoA synthetase long-chain family member 6
228675_at	100131733	USP30-AS1	USP30 antisense RNA 1
1554367_at	255022	CALHM1	calcium homeostasis modulator 1
1556662_at	100506142	LOC100506142	uncharacterized LOC100506142
207560_at	9154	SLC28A1	solute carrier family 28 (concentrative nucleoside transporter), member 1
1569515_a_at	100996681	LOC100996681	uncharacterized LOC100996681
236977_at	646588	LOC646588	uncharacterized LOC646588
210419_at	8538	BARX2	BARX homeobox 2
1562103_at	101927084	LINC01359	long intergenic non-protein coding RNA 1359
220972_s_at	81870	KRTAP9-9	keratin associated protein 9-9
239430_at	374918	IGFL1	IGF-like family member 1
228715_at	170261	ZCCHC12	zinc finger, CCHC domain containing 12
208366_at	27328	PCDH11X	protocadherin 11 X-linked
206160_at	10930	APOBEC2	apolipoprotein B mRNA editing enzyme, catalytic polypeptide-like 2
230565_at	127124	ATP6V1G3	ATPase, H+ transporting, lysosomal 13kDa, V1 subunit G3
211349_at	6564	SLC15A1	solute carrier family 15 (oligopeptide transporter), member 1
234109_x_at	390874	ONECUT3	one cut homeobox 3
240801_at	100505929	C21orf37	chromosome 21 open reading frame 37
1559333_at	101927416	LOC101927416	uncharacterized LOC101927416
1570373_at	155061	ZNF746	zinc finger protein 746
207389_at	2811	GP1BA	glycoprotein Ib (platelet), alpha polypeptide

215267_s_at	6543	SLC8A2	solute carrier family 8 (sodium/calcium exchanger), member 2
220232_at	79966	SCD5	stearoyl-CoA desaturase 5
1564229_at	729173	LOC729173	uncharacterized LOC729173
1559789_a_at	163115	ZNF781	zinc finger protein 781
216986_s_at	3662	IRF4	interferon regulatory factor 4
208138_at	2520	GAST	gastrin
208378_x_at	2250	FGF5	fibroblast growth factor 5
233634_at	91862	MARVELD3	MARVEL domain containing 3
1561978_at	284798	LOC284798	uncharacterized LOC284798
240268_at	440117	LOC440117	uncharacterized LOC440117
220491_at	57817	HAMP	hepcidin antimicrobial peptide
236226_at	151888	BTLA	B and T lymphocyte associated
1552510_at	142680	SLC34A3	solute carrier family 34 (type II sodium/phosphate contransporter), member 3
1570230_at	101927884	LOC101927884	uncharacterized LOC101927884
1556740_at	100506475	EGFLAM-AS2	EGFLAM antisense RNA 2
1557068_at	100505782	LOC100505782	uncharacterized LOC100505782
209769_s_at	100526833	SEPT5-GP1BB	SEPT5-GP1BB readthrough
206216_at	26576	SRPK3	SRSF protein kinase 3
202525_at	5652	PRSS8	protease, serine, 8
211693_at	3492	IGH	immunoglobulin heavy locus
204816_s_at	9704	DHX34	DEAH (Asp-Glu-Ala-His) box polypeptide 34
207309_at	4842	NOS1	nitric oxide synthase 1 (neuronal)
207964_x_at	3441	IFNA4	interferon, alpha 4
210383_at	6323	SCN1A	sodium channel, voltage-gated, type I, alpha subunit
1563776_at	100507283	LOC100507283	uncharacterized LOC100507283
1563062_at	728192	LINC00460	long intergenic non-protein coding RNA 460
232674_at	90226	UCN2	urocortin 2
225051_at	2035	EPB41	erythrocyte membrane protein band 4.1
221394_at	9287	TAAR2	trace amine associated receptor 2
1559670_at	100506489	LOC100506489	uncharacterized LOC100506489
234764_x_at	28823	IGLV1-44	immunoglobulin lambda variable 1-44
1554887_at	101927746	LOC101927746	uncharacterized LOC101927746
207393_at	3062	HCRTR2	hypocretin (orexin) receptor 2
206521_s_at	2957	GTF2A1	general transcription factor IIA, 1, 19/37kDa
241290_at	101927342	LOC101927342	uncharacterized LOC101927342
1564691_at	100128594	LL0XNC01-116E7.2	uncharacterized LOC100128594
216676_x_at	115653	KIR3DL3	killer cell immunoglobulin-like receptor, three domains, long cytoplasmic tail, 3
232332_at	57481	KIAA1210	KIAA1210
1554988_at	284525	SLC9C2	solute carrier family 9, member C2 (putative)
1555752_at	246744	STH	saitohin
244649_at	646484	LOC646484	uncharacterized LOC646484
205866_at	8547	FCN3	ficolin (collagen/fibrinogen domain containing) 3
1554960_at	339512	C1orf110	chromosome 1 open reading frame 110
216106_at	145678	LOC145678	uncharacterized LOC145678
232456_at	118461	C10orf71	chromosome 10 open reading frame 71
1561085_at	153910	LOC153910	uncharacterized LOC153910
224157_at	353219	KAAG1	kidney associated antigen 1
230965_at	9099	USP2	ubiquitin specific peptidase 2
1555656_at	146894	CD300LG	CD300 molecule-like family member g
211232_x_at	2740	GLP1R	glucagon-like peptide 1 receptor
1555238_at	113091	PTH2	parathyroid hormone 2
210686_x_at	8034	SLC25A16	solute carrier family 25 (mitochondrial carrier), member 16
1557389_at	100505839	SH3PXD2A-AS1	SH3PXD2A antisense RNA 1
217613_at	55314	TMEM144	transmembrane protein 144
1554949_at	554174	LOC554174	uncharacterized LOC554174
236598_at	100996579	LOC100996579	uncharacterized LOC100996579
31861_at	3508	IGHMBP2	immunoglobulin mu binding protein 2
220236_at	55066	PDPR	pyruvate dehydrogenase phosphatase regulatory subunit
233234_at	57528	KCTD16	potassium channel tetramerization domain containing 16
216959_x_at	4897	NRCAM	neuronal cell adhesion molecule

241432_at	100505893	SLIT2-IT1	SLIT2 intronic transcript 1 (non-protein coding)
207598_x_at	7516	XRCC2	X-ray repair complementing defective repair in Chinese hamster cells 2
1570219_at	84171	LOXL4	lysyl oxidase-like 4
207840_at	11126	CD160	CD160 molecule
230830_at	123264	SLC51B	solute carrier family 51, beta subunit
234685_x_at	100132386	KRTAP4-9	keratin associated protein 4-9
233594_at	100506025	ISPD-AS1	ISPD antisense RNA 1
1552421_a_at	125972	CALR3	calreticulin 3
215759_at	79998	ANKRD53	ankyrin repeat domain 53
235239_at	169714	QSOX2	quiescin Q6 sulfhydryl oxidase 2
230949_at	151295	SLC23A3	solute carrier family 23, member 3
221226_s_at	55515	ASIC4	acid-sensing (proton-gated) ion channel family member 4
1557776_at	101928303	LOC101928303	uncharacterized LOC101928303
220008_at	79834	PEAK1	pseudopodium-enriched atypical kinase 1
1560854_s_at	51427	ZNF107	zinc finger protein 107
1569436_at	400128	TUSC8	tumor suppressor candidate 8 (non-protein coding)
239641_at	100507392	SENCR	smooth muscle and endothelial cell enriched migration/differentiation-associated long non-coding RNA
1560130_at	574407	C1orf145	chromosome 1 open reading frame 145
1556771_a_at	415056	CNTFR-AS1	CNTFR antisense RNA 1
229789_at	220359	TIGD3	tigger transposable element derived 3
1553434_at	163720	CYP4Z2P	cytochrome P450, family 4, subfamily Z, polypeptide 2, pseudogene
1557215_at	100506433	LINC00648	long intergenic non-protein coding RNA 648
1560891_a_at	100505875	LINC01088	long intergenic non-protein coding RNA 1088
1553041_at	170572	HTR3C	5-hydroxytryptamine (serotonin) receptor 3C, ionotropic
1569987_at	100874074	DLEU7-AS1	DLEU7 antisense RNA 1
204891_s_at	3932	LCK	LCK proto-oncogene, Src family tyrosine kinase
1561305_at	101927849	LOC101927849	uncharacterized LOC101927849
212430_at	55544	RBM38	RNA binding motif protein 38
216184_s_at	22999	RIMS1	regulating synaptic membrane exocytosis 1
1556842_at	286087	LOC286087	uncharacterized LOC286087
1560448_at	340037	PRR7-AS1	PRR7 antisense RNA 1
220428_at	50489	CD207	CD207 molecule, langerin
1553023_a_at	79400	NOX5	NADPH oxidase, EF-hand calcium binding domain 5
1564012_at	100132354	LINC01512	long intergenic non-protein coding RNA 1512
223299_at	90701	SEC11C	SEC11 homolog C (S. cerevisiae)
1556999_at	100271832	LOC100271832	uncharacterized LOC100271832
1553401_at	259249	MRGPRX1	MAS-related GPR, member X1
1553891_at	128209	KLF17	Kruppel-like factor 17
1563849_at	387694	SH2D4B	SH2 domain containing 4B
210929_s_at	197	AHSG	alpha-2-HS-glycoprotein
224323_s_at	83876	MRO	maestro
1554099_a_at	169981	SPIN3	spindlin family, member 3
1559606_at	163351	GBP6	guanylate binding protein family, member 6
211798_x_at	28831	IGLJ3	immunoglobulin lambda joining 3
1563978_at	728690	LOC728690	uncharacterized LOC728690
232318_s_at	121838	LINC00284	long intergenic non-protein coding RNA 284
221404_at	27179	IL36A	interleukin 36, alpha
1561386_at	102723701	LOC102723701	uncharacterized LOC102723701
220069_at	51807	TUBA8	tubulin, alpha 8
206524_at	6862	T	T, brachyury homolog (mouse)
1553702_at	90874	ZNF697	zinc finger protein 697
220826_at	140290	TCP10L	t-complex 10-like
206981_at	6329	SCN4A	sodium channel, voltage-gated, type IV, alpha subunit
1558653_at	339751	MLK7-AS1	MLK7 antisense RNA 1
227806_at	404550	C16orf74	chromosome 16 open reading frame 74
220844_at	51224	TCEB3B	transcription elongation factor B polypeptide 3B (elongin A2)
205671_s_at	3112	HLA-DOB	major histocompatibility complex, class II, DO beta
1569040_s_at	645784	ANKRD36BP2	ankyrin repeat domain 36B pseudogene 2
237365_at	100506409	ELOVL2-AS1	ELOVL2 antisense RNA 1
1561266_at	100507033	LOC100507033	uncharacterized LOC100507033
204830_x_at	5673	PSG5	pregnancy specific beta-1-glycoprotein 5

231096_at	118425	PCAT4	prostate cancer associated transcript 4 (non-protein coding)
219301_s_at	26047	CNTNAP2	contactin associated protein-like 2
204856_at	10331	B3GNT3	UDP-GlcNAc:betaGal beta-1,3-N-acetylglucosaminyltransferase 3
1562372_at	22987	SV2C	synaptic vesicle glycoprotein 2C
216408_at	81697	OR2B2	olfactory receptor, family 2, subfamily B, member 2
238943_at	84929	FIBCD1	fibrinogen C domain containing 1
1553226_at	145978	LINC00052	long intergenic non-protein coding RNA 52
211339_s_at	3702	ITK	IL2-inducible T-cell kinase
213888_s_at	80342	TRAF3IP3	TRAF3 interacting protein 3
238885_at	57670	KIAA1549	KIAA1549
240887_at	100506470	LOC100506470	uncharacterized LOC100506470
238708_at	386758	ZNF582-AS1	ZNF582 antisense RNA 1 (head to head)
208550_x_at	26251	KCNG2	potassium voltage-gated channel, subfamily G, member 2
1559372_at	101928043	LOC101928043	uncharacterized LOC101928043
1560548_at	101927195	LINC00382	long intergenic non-protein coding RNA 382
241380_at	389337	ARHGEF37	Rho guanine nucleotide exchange factor (GEF) 37
1556859_a_at	285740	LOC285740	uncharacterized LOC285740
229547_s_at	65268	WNK2	WNK lysine deficient protein kinase 2
1564333_a_at	768239	PSAPL1	prosaposin-like 1 (gene/pseudogene)
229244_at	4045	LSAMP	limbic system-associated membrane protein
214219_x_at	11184	MAP4K1	mitogen-activated protein kinase kinase kinase kinase 1
1560712_at	132724	TMPRSS11B	transmembrane protease, serine 11B
1566289_at	26245	OR2M4	olfactory receptor, family 2, subfamily M, member 4
221444_at	50833	TAS2R16	taste receptor, type 2, member 16
1569983_at	100861547	LINC00566	long intergenic non-protein coding RNA 566
206299_at	27112	FAM155B	family with sequence similarity 155, member B
208037_s_at	8174	MADCAM1	mucosal vascular addressin cell adhesion molecule 1
202612_s_at	9282	MED14	mediator complex subunit 14
1553020_at	140771	SMCR5	Smith-Magenis syndrome chromosome region, candidate 5 (non-protein coding)
208058_s_at	4248	MGAT3	mannosyl (beta-1,4-)-glycoprotein beta-1,4-N-acetylglucosaminyltransferase
201427_s_at	6414	SEPP1	selenoprotein P, plasma, 1
220270_at	56163	RNF17	ring finger protein 17
225912_at	94241	TP53INP1	tumor protein p53 inducible nuclear protein 1
1562633_at	196475	RMST	rhabdomyosarcoma 2 associated transcript (non-protein coding)
1561243_at	284186	TMEM105	transmembrane protein 105
234678_at	85290	KRTAP4-3	keratin associated protein 4-3
1557330_at	103581031	CASC23	cancer susceptibility candidate 23 (non-protein coding)
214340_at	245	ALOX12P2	arachidonate 12-lipoxygenase pseudogene 2
1553912_at	285492	LINC00955	long intergenic non-protein coding RNA 955
240070_at	201633	TIGIT	T cell immunoreceptor with Ig and ITIM domains
224404_s_at	83416	FCRL5	Fc receptor-like 5
1561587_at	284260	LINC00907	long intergenic non-protein coding RNA 907
1555586_at	153745	FAM71B	family with sequence similarity 71, member B
1555196_at	100287114	LINC00421	long intergenic non-protein coding RNA 421
231549_at	93190	C1orf158	chromosome 1 open reading frame 158
231012_at	159371	SLC35G1	solute carrier family 35, member G1
214699_x_at	26100	WIPI2	WD repeat domain, phosphoinositide interacting 2
214470_at	3820	KLRB1	killer cell lectin-like receptor subfamily B, member 1
1556292_s_at	285877	POM121L12	POM121 transmembrane nucleoporin-like 12
1565454_at	139629	XAGE-4	XAGE-4 protein
231363_at	149018	LELP1	late cornified envelope-like proline-rich 1
206339_at	9607	CARTPT	CART prepropeptide
1558324_a_at	643236	TMEM72	transmembrane protein 72
201547_at	10765	KDM5B	lysine (K)-specific demethylase 5B
1564426_x_at	729732	LOC729732	uncharacterized LOC729732
236341_at	1493	CTLA4	cytotoxic T-lymphocyte-associated protein 4
206660_at	3543	IGLL1	immunoglobulin lambda-like polypeptide 1
209604_s_at	2625	GATA3	GATA binding protein 3
1560131_at	100506497	LOC100506497	uncharacterized LOC100506497
1569465_at	729830	FAM160A1	family with sequence similarity 160, member A1
1567288_at	26339	OR5K1	olfactory receptor, family 5, subfamily K, member 1
221682_s_at	56100	PCDHGB6	protocadherin gamma subfamily B, 6

222925_at	51473	DCDC2	doublecortin domain containing 2
221681_s_at	1834	DSPP	dentin sialophosphoprotein
1566544_at	100506012	PPP5D1	PPP5 tetratricopeptide repeat domain containing 1
238820_at	161145	TMEM229B	transmembrane protein 229B
1552973_at	7484	WNT9B	wingless-type MMTV integration site family, member 9B
203864_s_at	88	ACTN2	actinin, alpha 2
224258_at	100169989	DBIL5P2	diazepam binding inhibitor-like 5 pseudogene 2
206438_x_at	79867	TCTN2	tectonic family member 2
233640_x_at	85280	KRTAP9-4	keratin associated protein 9-4
231517_at	440590	ZYG11A	zyg-11 family member A, cell cycle regulator
1556639_at	100996455	LOC100996455	uncharacterized LOC100996455
208283_at	2543	GAGE1	G antigen 1
206816_s_at	26206	SPAG8	sperm associated antigen 8
1564446_at	284930	LOC284930	uncharacterized LOC284930
241596_at	170685	NUDT10	nudix (nucleoside diphosphate linked moiety X)-type motif 10
213539_at	915	CD3D	CD3d molecule, delta (CD3-TCR complex)
222009_at	752014	CEMP1	cementum protein 1
224425_x_at	440888	ACTR3BP2	ACTR3B pseudogene 2
204118_at	962	CD48	CD48 molecule
1563082_at	285045	LINC00486	long intergenic non-protein coding RNA 486
1560413_at	339788	LINC00298	long intergenic non-protein coding RNA 298
1553898_a_at	222029	DKFZp434L192	uncharacterized protein DKFZp434L192
206181_at	6504	SLAMF1	signaling lymphocytic activation molecule family member 1
231081_at	129852	C2orf73	chromosome 2 open reading frame 73
233812_at	140875	LINC00028	long intergenic non-protein coding RNA 28
1569660_at	100128046	PP13439	uncharacterized LOC100128046
1559812_at	101927944	FAM53B-AS1	FAM53B antisense RNA 1
1559528_at	100129917	LOC100129917	uncharacterized LOC100129917
1569887_a_at	286135	FAM183CP	family with sequence similarity 183, member C, pseudogene
214372_x_at	10595	ERN2	endoplasmic reticulum to nucleus signaling 2
1552623_at	84941	HSH2D	hematopoietic SH2 domain containing
1553238_a_at	147746	HIPK4	homeodomain interacting protein kinase 4
1564138_at	285231	FBXW12	F-box and WD repeat domain containing 12
228621_at	148738	HFE2	hemochromatosis type 2 (juvenile)
232388_at	85445	CNTNAP4	contactin associated protein-like 4
239028_at	130574	LYPD6	LY6/PLAUR domain containing 6
1562634_at	101927869	LOC101927869	uncharacterized LOC101927869
220149_at	79919	C2orf54	chromosome 2 open reading frame 54
231469_at	283738	NTRK3-AS1	NTRK3 antisense RNA 1
1553506_at	130749	CPO	carboxypeptidase O
1557493_x_at	100240728	LOC100240728	uncharacterized LOC100240728
206769_at	9087	TMSB4Y	thymosin beta 4, Y-linked
1561361_at	285349	ZNF660	zinc finger protein 660
239417_x_at	347744	C6orf52	chromosome 6 open reading frame 52
1555400_at	645261	LOC645261	PP565
232822_x_at	100506757	LINC00629	long intergenic non-protein coding RNA 629
1557758_at	101928535	LOC101928535	uncharacterized LOC101928535
210614_at	7274	TTPA	tocopherol (alpha) transfer protein
234461_at	200312	RNF215	ring finger protein 215
205267_at	5450	POU2AF1	POU class 2 associating factor 1
210039_s_at	5588	PRKCQ	protein kinase C, theta
218614_at	55196	KIAA1551	KIAA1551
243349_at	57535	KIAA1324	KIAA1324
236811_at	63946	DMRTC2	DMRT-like family C2
1561257_at	286083	LOC286083	uncharacterized LOC286083
223781_x_at	127	ADH4	alcohol dehydrogenase 4 (class II), pi polypeptide
204973_at	2705	GJB1	gap junction protein, beta 1, 32kDa
1559645_at	100302691	LINC00184	long intergenic non-protein coding RNA 184
1557873_at	102546294	LOC102546294	uncharacterized LOC102546294
231571_at	100507362	LINC01015	long intergenic non-protein coding RNA 1015
1560911_at	100133461	LOC100133461	uncharacterized LOC100133461
231277_x_at	285605	DTWD2	DTW domain containing 2

1558623_at	729121	RGPD4-AS1	RGPD4 antisense RNA 1 (head to head)
1560754_at	112616	CMTM7	CKLF-like MARVEL transmembrane domain containing 7
1558493_at	164380	CST13P	cystatin 13, pseudogene
220306_at	54855	FAM46C	family with sequence similarity 46, member C
243263_at	284185	LINC00482	long intergenic non-protein coding RNA 482
1558790_s_at	286103	ZNF252P-AS1	ZNF252P antisense RNA 1
206872_at	6568	SLC17A1	solute carrier family 17 (organic anion transporter), member 1
1562826_at	101927049	PLCE1-AS2	PLCE1 antisense RNA 2
208004_at	58503	PROL1	proline rich, lacrimal 1
236933_at	645027	EVPLL	envoplakin-like
235245_at	162461	TMEM92	transmembrane protein 92
221355_at	1146	CHRNG	cholinergic receptor, nicotinic, gamma (muscle)
235297_at	10658	CELF1	CUGBP, Elav-like family member 1
1557879_at	100129175	LOC100129175	uncharacterized LOC100129175
231491_at	54088	LINC00113	long intergenic non-protein coding RNA 113
1553064_at	132243	H1FOO	H1 histone family, member O, oocyte-specific
1557866_at	286207	C9orf117	chromosome 9 open reading frame 117
1556206_at	100652856	LINC00408	long intergenic non-protein coding RNA 408
244710_at	136332	LRGUK	leucine-rich repeats and guanylate kinase domain containing
207084_at	5454	POU3F2	POU class 3 homeobox 2
1554842_at	6557	SLC12A1	solute carrier family 12 (sodium/potassium/chloride transporter), member 1
1563316_at	100852409	NEGR1-IT1	NEGR1 intronic transcript 1 (non-protein coding)
1569172_a_at	402160	CFAP99	cilia and flagella associated protein 99
1554115_at	133874	C5orf58	chromosome 5 open reading frame 58
206410_at	8431	NR0B2	nuclear receptor subfamily 0, group B, member 2
243614_s_at	58510	PRODH2	proline dehydrogenase (oxidase) 2
207663_x_at	2575	GAGE3	G antigen 3
237453_at	57711	ZNF529	zinc finger protein 529
1554325_at	1794	DOCK2	dedicator of cytokinesis 2
210545_at	50618	ITSN2	intersectin 2
215946_x_at	91353	IGLL3P	immunoglobulin lambda-like polypeptide 3, pseudogene
230981_at	347732	CATSPER3	cation channel, sperm associated 3
223905_at	84229	DRC7	dynein regulatory complex subunit 7
211053_at	3755	KCNG1	potassium voltage-gated channel, subfamily G, member 1
210085_s_at	8416	ANXA9	annexin A9
215323_at	338645	LUZP2	leucine zipper protein 2
224097_s_at	50848	F11R	F11 receptor
210991_s_at	9783	RIMS3	regulating synaptic membrane exocytosis 3
244667_at	100996425	LOC100996425	uncharacterized LOC100996425
207051_at	10050	SLC17A4	solute carrier family 17, member 4
213947_s_at	23225	NUP210	nucleoporin 210kDa
239289_x_at	22909	FAN1	FANCD2/FANCI-associated nuclease 1
231776_at	8320	EOMES	eomesodermin
1553880_at	170393	C10orf91	chromosome 10 open reading frame 91
213502_x_at	91316	GUSBP11	glucuronidase, beta pseudogene 11
220062_s_at	51438	MAGEC2	melanoma antigen family C, 2
1564383_s_at	400579	FLJ35934	FLJ35934
207199_at	7015	TERT	telomerase reverse transcriptase
1561098_at	641365	LINC00616	long intergenic non-protein coding RNA 616
1552955_at	83655	LINC00208	long intergenic non-protein coding RNA 208
209670_at	28755	TRAC	T cell receptor alpha constant
206674_at	2322	FLT3	fms-related tyrosine kinase 3
206663_at	6671	SP4	Sp4 transcription factor
230882_at	285987	DLX6-AS1	DLX6 antisense RNA 1
214303_x_at	4586	MUC5AC	mucin 5AC, oligomeric mucus/gel-forming
1555550_at	353174	ZACN	zinc activated ligand-gated ion channel
234017_at	91948	LINC00923	long intergenic non-protein coding RNA 923
217422_s_at	933	CD22	CD22 molecule
205595_at	1830	DSG3	desmoglein 3
1557550_at	148145	LINC00906	long intergenic non-protein coding RNA 906
1557633_at	29797	POM121L8P	POM121 transmembrane nucleoporin-like 8 pseudogene

1561471_at	441009	CPEB2-AS1	CPEB2 antisense RNA 1 (head to head)
207366_at	3787	KCNS1	potassium voltage-gated channel, delayed-rectifier, subfamily S, member 1
233949_s_at	57644	MYH7B	myosin, heavy chain 7B, cardiac muscle, beta
1554800_at	54734	RAB39A	RAB39A, member RAS oncogene family
1560047_s_at	642826	BMS1P6	BMS1 pseudogene 6
224493_x_at	85019	TMEM241	transmembrane protein 241
206798_x_at	9940	DLEC1	deleted in lung and esophageal cancer 1
220562_at	54905	CYP2W1	cytochrome P450, family 2, subfamily W, polypeptide 1
207910_at	10648	SCGB1D1	secretoglobin, family 1D, member 1
1554983_at	378828	LINC00317	long intergenic non-protein coding RNA 317
205015_s_at	7039	TGFA	transforming growth factor, alpha
231306_at	131375	LYZL4	lysozyme-like 4
244136_at	100507261	MIR670HG	MIR670 host gene (non-protein coding)
1569631_at	64802	NMNAT1	nicotinamide nucleotide adenylyltransferase 1
224168_at	84203	TXNDC2	thioredoxin domain containing 2 (spermatozoa)
207013_s_at	4325	MMP16	matrix metallopeptidase 16 (membrane-inserted)
231748_at	79465	ULBP3	UL16 binding protein 3
227450_at	121506	ERP27	endoplasmic reticulum protein 27
1557588_at	100128946	LINC01310	long intergenic non-protein coding RNA 1310
220747_at	29075	LINC00652	long intergenic non-protein coding RNA 652
1555757_at	135927	C7orf34	chromosome 7 open reading frame 34
81737_at	100505915	LOC100505915	uncharacterized LOC100505915
223906_s_at	83639	TEX101	testis expressed 101
1554911_at	85320	ABCC11	ATP-binding cassette, sub-family C (CFTR/MRP), member 11
1564083_at	101926975	LOC101926975	uncharacterized LOC101926975
233321_x_at	90834	LOC90834	uncharacterized protein BC001742
206642_at	1828	DSG1	desmoglein 1
205049_s_at	973	CD79A	CD79a molecule, immunoglobulin-associated alpha
1553812_at	79816	TLE6	transducin-like enhancer of split 6
1559340_at	401550	TTLL11-IT1	TTLL11 intronic transcript 1 (non-protein coding)
1555180_at	100132686	LOC100132686	uncharacterized LOC100132686
207446_at	10333	TLR6	toll-like receptor 6
1557466_at	441072	FLJ31104	uncharacterized LOC441072
220867_s_at	25769	SLC24A2	solute carrier family 24 (sodium/potassium/calcium exchanger), member 2
1556505_at	100131366	LINC00605	long intergenic non-protein coding RNA 605
207203_s_at	8856	NR1I2	nuclear receptor subfamily 1, group I, member 2
207351_s_at	9047	SH2D2A	SH2 domain containing 2A
211557_x_at	11309	SLCO2B1	solute carrier organic anion transporter family, member 2B1
1569783_at	401387	LRRD1	leucine-rich repeats and death domain containing 1
1554989_at	9870	AREL1	apoptosis resistant E3 ubiquitin protein ligase 1
216969_s_at	3835	KIF22	kinesin family member 22
203413_at	4753	NELL2	NEL-like 2 (chicken)
236597_at	133688	UGT3A1	UDP glycosyltransferase 3 family, polypeptide A1
1553429_at	158263	FLJ31713	uncharacterized protein FLJ31713
1553131_a_at	2626	GATA4	GATA binding protein 4
221401_at	27091	CACNG5	calcium channel, voltage-dependent, gamma subunit 5
1560757_at	116447	TOP1MT	topoisomerase (DNA) I, mitochondrial
230940_at	100288123	LOC100288123	uncharacterized LOC100288123
233301_at	64064	OXCT2	3-oxoacid CoA transferase 2
1554642_at	140545	RNF32	ring finger protein 32
1561602_at	100129476	LOC100129476	uncharacterized LOC100129476
205815_at	5068	REG3A	regenerating islet-derived 3 alpha
210452_x_at	8529	CYP4F2	cytochrome P450, family 4, subfamily F, polypeptide 2
1562294_x_at	374860	ANKRD30B	ankyrin repeat domain 30B
217115_at	7686	MKRN7P	makorin ring finger protein 7, pseudogene
221334_s_at	50943	FOXP3	forkhead box P3
232343_at	84516	DCTN5	dynactin 5 (p25)
207846_at	5449	POU1F1	POU class 1 homeobox 1
1562643_at	101929279	LOC101929279	uncharacterized LOC101929279
231448_at	132612	ADAD1	adenosine deaminase domain containing 1 (testis-specific)
208285_at	26659	OR7A5	olfactory receptor, family 7, subfamily A, member 5

237211_x_at	283385	MORN3	MORN repeat containing 3
1556406_at	255025	LINC00879	long intergenic non-protein coding RNA 879
233403_x_at	53345	TM6SF2	transmembrane 6 superfamily member 2
220982_s_at	81833	SPACA1	sperm acrosome associated 1
1569755_at	100499171	LINC00276	long intergenic non-protein coding RNA 276
208574_at	8403	SOX14	SRY (sex determining region Y)-box 14
1564233_at	285150	FLJ33534	uncharacterized LOC285150
208261_x_at	3446	IFNA10	interferon, alpha 10
207569_at	6098	ROS1	ROS proto-oncogene 1 , receptor tyrosine kinase
205602_x_at	5676	PSG7	pregnancy specific beta-1-glycoprotein 7 (gene/pseudogene)
230562_at	100507530	LOC100507530	uncharacterized LOC100507530
223856_at	83592	AKR1E2	aldo-keto reductase family 1, member E2
206824_at	51716	CES1P1	carboxylesterase 1 pseudogene 1
238054_at	113622	ADPRHL1	ADP-ribosylhydrolase like 1
239620_at	153657	TTC23L	tetratricopeptide repeat domain 23-like
217016_x_at	389177	TMEM212	transmembrane protein 212
210288_at	10219	KLRG1	killer cell lectin-like receptor subfamily G, member 1
1555634_a_at	353514	LILRA5	leukocyte immunoglobulin-like receptor, subfamily A (with TM domain), member 5
206837_at	8092	ALX1	ALX homeobox 1
227885_at	400236	FOXN3-AS1	FOXN3 antisense RNA 1
1562073_at	101927815	LOC101927815	uncharacterized LOC101927815
204914_s_at	6664	SOX11	SRY (sex determining region Y)-box 11
215972_at	25859	PART1	prostate androgen-regulated transcript 1 (non-protein coding)
1562052_at	101927159	LOC101927159	uncharacterized LOC101927159
1570250_at	54886	LPPR1	lipid phosphate phosphatase-related protein type 1
1552473_at	2593	GAMT	guanidinoacetate N-methyltransferase
219513_s_at	10045	SH2D3A	SH2 domain containing 3A
1557021_s_at	100507250	LOC100507250	uncharacterized LOC100507250
213193_x_at	28639	TRBC1	T cell receptor beta constant 1
1559344_at	101929064	LOC101929064	uncharacterized LOC101929064
1559658_at	79768	KATNBL1	katanin p80 subunit B-like 1
239141_at	100126784	LOC100126784	uncharacterized LOC100126784
235863_at	126306	JSRP1	junctional sarcoplasmic reticulum protein 1
223570_at	55388	MCM10	minichromosome maintenance complex component 10
211633_x_at	3500	IGHG1	immunoglobulin heavy constant gamma 1 (G1m marker)
211837_s_at	171558	PTCRA	pre T-cell antigen receptor alpha
1562895_at	101927502	LOC101927502	uncharacterized LOC101927502
242804_at	353497	POLN	polymerase (DNA directed) nu
219472_at	79172	CENPO	centromere protein O
207938_at	51050	PI15	peptidase inhibitor 15
237507_at	319101	KRT73	keratin 73
1557666_s_at	158067	AK8	adenylate kinase 8
238567_at	130367	SGPP2	sphingosine-1-phosphate phosphatase 2
1560147_at	100131176	WDR86-AS1	WDR86 antisense RNA 1
206123_at	3996	LLGL1	lethal giant larvae homolog 1 (Drosophila)
1553752_at	128497	SPATA25	spermatogenesis associated 25
1559714_at	5995	RGR	retinal G protein coupled receptor
207593_at	64137	ABCG4	ATP-binding cassette, sub-family G (WHITE), member 4
222781_s_at	55071	C9orf40	chromosome 9 open reading frame 40
1569372_at	100507194	LOC100507194	uncharacterized LOC100507194
1559390_a_at	149643	SPATA45	spermatogenesis associated 45
1557826_at	338817	LINC01252	long intergenic non-protein coding RNA 1252
235083_at	151009	LINC01106	long intergenic non-protein coding RNA 1106
1569709_at	23334	SZT2	seizure threshold 2 homolog (mouse)
215584_at	23072	HECW1	HECT, C2 and WW domain containing E3 ubiquitin protein ligase 1
1560963_a_at	100506379	LOC100506379	uncharacterized LOC100506379
207734_at	54900	LAX1	lymphocyte transmembrane adaptor 1
1552865_a_at	160492	LMNTD1	lamin tail domain containing 1
220067_at	51332	SPTBN5	spectrin, beta, non-erythrocytic 5
204475_at	4312	MMP1	matrix metallopeptidase 1 (interstitial collagenase)
229721_x_at	91319	DERL3	derlin 3

1559540_at	100506526	LOC100506526	uncharacterized LOC100506526
204664_at	250	ALPP	alkaline phosphatase, placental
1554318_at	541473	LOC541473	FK506 binding protein 6, 36kDa pseudogene
1569138_a_at	10847	SRCAP	Snf2-related CREBBP activator protein
215590_x_at	100128640	ACVR2B-AS1	ACVR2B antisense RNA 1
1560932_at	403150	FLJ31356	uncharacterized protein FLJ31356
206711_at	9142	TMEM257	transmembrane protein 257
232248_at	100652768	LOC100652768	uncharacterized LOC100652768
220831_at	51301	GCNT4	glucosaminyl (N-acetyl) transferase 4, core 2
1562523_at	101927641	LOC101927641	uncharacterized LOC101927641
207021_at	11055	ZPBP	zona pellucida binding protein
1555085_at	101928751	LOC101928751	uncharacterized LOC101928751
1568639_a_at	101927167	GATA2-AS1	GATA2 antisense RNA 1
220451_s_at	79444	BIRC7	baculoviral IAP repeat containing 7
220472_at	29063	ZCCHC4	zinc finger, CCHC domain containing 4
204613_at	5336	PLCG2	phospholipase C, gamma 2 (phosphatidylinositol-specific)
1557821_at	283547	LINC00639	long intergenic non-protein coding RNA 639
216734_s_at	643	CXCR5	chemokine (C-X-C motif) receptor 5
207218_at	2158	F9	coagulation factor IX
1557608_a_at	284080	LOC284080	uncharacterized LOC284080
1558546_at	1773	DNASE1	deoxyribonuclease I
228252_at	80119	PIF1	PIF1 5'-to-3' DNA helicase
205590_at	10125	RASGRP1	RAS guanyl releasing protein 1 (calcium and DAG-regulated)
228658_at	440823	MIAT	myocardial infarction associated transcript (non-protein coding)
1568617_a_at	57662	CAMSAP3	calmodulin regulated spectrin-associated protein family, member 3
231958_at	132001	TAMM41	TAM41, mitochondrial translocator assembly and maintenance protein, homolog (S. cerevisiae)
1552917_at	282618	IFNL1	interferon, lambda 1
1552578_a_at	140469	MYO3B	myosin IIIB
1568723_at	374868	ATP9B	ATPase, class II, type 9B
1553185_at	158158	RASEF	RAS and EF-hand domain containing
230441_at	153478	PLEKHG4B	pleckstrin homology domain containing, family G (with RhoGef domain) member 4B
220645_at	54827	NXPE4	neurexophilin and PC-esterase domain family, member 4
231323_at	5690	PSMB2	proteasome (prosome, macropain) subunit, beta type, 2
1564460_at	286442	LINC01281	long intergenic non-protein coding RNA 1281
205319_at	8000	PSCA	prostate stem cell antigen
207664_at	2515	ADAM2	ADAM metallopeptidase domain 2
1559522_at	100505918	LOC100505918	uncharacterized LOC100505918
221338_at	79136	LY6G6E	lymphocyte antigen 6 complex, locus G6E (pseudogene)
1565583_at	79149	ZSCAN5A	zinc finger and SCAN domain containing 5A
1568796_at	550631	CCDC157	coiled-coil domain containing 157
1555189_a_at	6898	TAT	tyrosine aminotransferase
239593_at	155006	TMEM213	transmembrane protein 213
212311_at	23231	SEL1L3	sel-1 suppressor of lin-12-like 3 (C. elegans)
205028_at	7216	TRO	trophinin
1553060_at	85481	PSKH2	protein serine kinase H2
207450_s_at	11281	POU6F2	POU class 6 homeobox 2
230789_at	140883	ZNF280B	zinc finger protein 280B
1556289_at	100506016	LOC100506016	uncharacterized LOC100506016
1559787_at	101927438	LOC101927438	uncharacterized LOC101927438
206150_at	939	CD27	CD27 molecule
229977_at	27156	RSPH14	radial spoke head 14 homolog (Chlamydomonas)
211301_at	3752	KCND3	potassium voltage-gated channel, Shal-related subfamily, member 3
1559715_at	100507391	LOC100507391	uncharacterized LOC100507391
242348_at	151647	FAM19A4	family with sequence similarity 19 (chemokine (C-C motif)-like), member A4
1557669_at	100132005	LOC100132005	uncharacterized LOC100132005
241233_x_at	391267	ANKRD20A11P	ankyrin repeat domain 20 family, member A11, pseudogene
205885_s_at	3676	ITGA4	integrin, alpha 4 (antigen CD49D, alpha 4 subunit of VLA-4 receptor)
233463_at	166824	RASSF6	Ras association (RalGDS/AF-6) domain family member 6
219425_at	25830	SULT4A1	sulfotransferase family 4A, member 1

1561141_at	101929504	LOC101929504	uncharacterized LOC101929504
235269_at	113828	FAM83F	family with sequence similarity 83, member F
1563878_a_at	338963	LOC338963	epididymal protein pseudogene
1558662_s_at	55024	BANK1	B-cell scaffold protein with ankyrin repeats 1
230469_at	219790	RTKN2	rhotekin 2
233514_x_at	56159	TEX11	testis expressed 11
1558523_at	79632	FAM184A	family with sequence similarity 184, member A
211828_s_at	23043	TNIK	TRAF2 and NCK interacting kinase
237808_at	93654	ST7-AS2	ST7 antisense RNA 2
1561342_at	150005	LOC150005	uncharacterized LOC150005
207823_s_at	199	AIF1	allograft inflammatory factor 1
230402_at	128853	DUSP15	dual specificity phosphatase 15
1564709_at	286238	LOC286238	uncharacterized LOC286238
211350_s_at	100505879	KIF25-AS1	KIF25 antisense RNA 1
1552915_at	282616	IFNL2	interferon, lambda 2
224123_at	84214	LOC84214	uncharacterized LOC84214
238600_at	152789	JAKMIP1	janus kinase and microtubule interacting protein 1
1562619_at	347736	NME9	NME/NM23 family member 9
224039_at	84070	FAM186B	family with sequence similarity 186, member B
1563589_at	340184	LOC340184	uncharacterized LOC340184
1553352_x_at	30816	ERVW-1	endogenous retrovirus group W, member 1
223565_at	51237	MZB1	marginal zone B and B1 cell-specific protein
207227_x_at	10739	RFPL2	ret finger protein-like 2
214994_at	200316	APOBEC3F	apolipoprotein B mRNA editing enzyme, catalytic polypeptide-like 3F
232239_at	643529	LINC00865	long intergenic non-protein coding RNA 865
228836_at	399512	SLC25A35	solute carrier family 25, member 35
243062_at	201163	FLCN	folliculin
1552608_at	259239	WFDC11	WAP four-disulfide core domain 11
214018_at	23426	GRIP1	glutamate receptor interacting protein 1
229475_at	84944	MAEL	maelstrom spermatogenic transposon silencer
214768_x_at	3514	IGKC	immunoglobulin kappa constant
239791_at	404266	HOXB-AS3	HOXB cluster antisense RNA 3
1552814_a_at	136259	KLF14	Kruppel-like factor 14
1561472_at	728805	LOC728805	uncharacterized LOC728805
1556405_s_at	374890	LOC374890	uncharacterized LOC374890
211908_x_at	50802	IGK	immunoglobulin kappa locus
230075_at	116442	RAB39B	RAB39B, member RAS oncogene family
220852_at	29018	FOXN3-AS2	FOXN3 antisense RNA 2
1554707_at	55064	SPATA6L	spermatogenesis associated 6-like
1569777_a_at	131368	ZPLD1	zona pellucida-like domain containing 1
236769_at	158402	LOC158402	uncharacterized LOC158402
224284_x_at	400949	FKSG49	FKSG49
206454_s_at	6010	RHO	rhodopsin
223903_at	54106	TLR9	toll-like receptor 9
203806_s_at	2175	FANCA	Fanconi anemia, complementation group A
215509_s_at	699	BUB1	BUB1 mitotic checkpoint serine/threonine kinase
1552772_at	338339	CLEC4D	C-type lectin domain family 4, member D
206628_at	6523	SLC5A1	solute carrier family 5 (sodium/glucose cotransporter), member 1
1561527_at	101927396	LINC01514	long intergenic non-protein coding RNA 1514
234799_at	104	ADARB1	adenosine deaminase, RNA-specific, B1
227919_at	652995	UCA1	urothelial cancer associated 1 (non-protein coding)
233171_at	116443	GRIN3A	glutamate receptor, ionotropic, N-methyl-D-aspartate 3A
220420_at	79748	LMAN1L	lectin, mannose-binding, 1 like
1556510_at	100996570	NFIA-AS2	NFIA antisense RNA 2
207139_at	495	ATP4A	ATPase, H+/K+ exchanging, alpha polypeptide
1562589_at	101928460	LOC101928460	uncharacterized LOC101928460
1552985_at	169026	SLC30A8	solute carrier family 30 (zinc transporter), member 8
1553622_a_at	161835	FSIP1	fibrous sheath interacting protein 1
208520_at	26532	OR10H3	olfactory receptor, family 10, subfamily H, member 3
1570262_at	101928851	LOC101928851	uncharacterized LOC101928851
1553541_at	4009	LMX1A	LIM homeobox transcription factor 1, alpha
204375_at	9746	CLSTN3	calsyntenin 3

208338_at	5024	P2RX3	purinergic receptor P2X, ligand-gated ion channel, 3
207955_at	10850	CCL27	chemokine (C-C motif) ligand 27
48030_i_at	10826	FAXDC2	fatty acid hydroxylase domain containing 2
222891_s_at	53335	BCL11A	B-cell CLL/lymphoma 11A (zinc finger protein)
201974_s_at	51622	CCZ1	CCZ1 vacuolar protein trafficking and biogenesis associated homolog (S. cerevisiae)
207183_at	2842	GPR19	G protein-coupled receptor 19
1561378_at	374470	C12orf42	chromosome 12 open reading frame 42
221122_at	54979	HRASLS2	HRAS-like suppressor 2
208209_s_at	725	C4BPB	complement component 4 binding protein, beta
208220_x_at	266	AMELY	amelogenin, Y-linked
204539_s_at	9620	CELSR1	cadherin, EGF LAG seven-pass G-type receptor 1
1557848_at	101929084	LOC101929084	uncharacterized LOC101929084
236771_at	134701	RIPPLY2	ripply transcriptional repressor 2
1564000_at	256006	ANKRD31	ankyrin repeat domain 31
222797_at	56896	DPYSL5	dihydropyrimidinase-like 5
1558971_at	387357	THEMIS	thymocyte selection associated
232777_s_at	168090	C6orf118	chromosome 6 open reading frame 118
227736_at	387695	C10orf99	chromosome 10 open reading frame 99
218965_s_at	64852	TUT1	terminal uridylyl transferase 1, U6 snRNA-specific
1553550_at	317705	VN1R5	vomeronasal 1 receptor 5 (gene/pseudogene)
207460_at	3004	GZMM	granzyme M (lymphocyte met-ase 1)
1556887_at	348808	NPHP3-AS1	NPHP3 antisense RNA 1
232765_x_at	146429	SLC22A31	solute carrier family 22, member 31
215705_at	5536	PPP5C	protein phosphatase 5, catalytic subunit
229104_s_at	2863	GPR39	G protein-coupled receptor 39
1561403_at	402381	SOHLH1	spermatogenesis and oogenesis specific basic helix-loop-helix 1
204740_at	10256	CNKSR1	connector enhancer of kinase suppressor of Ras 1
238853_at	117177	RAB3IP	RAB3A interacting protein
1563742_at	643650	LINC00842	long intergenic non-protein coding RNA 842
1563165_at	101928978	LOC101928978	uncharacterized LOC101928978
1554857_at	63916	ELMO2	engulfment and cell motility 2
210116_at	4068	SH2D1A	SH2 domain containing 1A
221169_s_at	59340	HRH4	histamine receptor H4
1555542_at	134265	AFAP1L1	actin filament associated protein 1-like 1
210883_x_at	1949	EFNB3	ephrin-B3
220152_at	79946	C10orf95	chromosome 10 open reading frame 95
229901_at	118738	ZNF488	zinc finger protein 488
1559145_at	386597	RNF144A-AS1	RNF144A antisense RNA 1
229286_at	57692	MAGEE1	melanoma antigen family E, 1
231043_at	200373	CFAP221	cilia and flagella associated protein 221
1561516_at	101928995	LOC101928995	uncharacterized LOC101928995
220965_s_at	81492	RSPH6A	radial spoke head 6 homolog A (Chlamydomonas)
207619_at	3061	HCRTR1	hypocretin (orexin) receptor 1
1567519_at	55558	PLXNA3	plexin A3
236767_at	389123	IQCF2	IQ motif containing F2
233541_at	644714	LIMD1-AS1	LIMD1 antisense RNA 1
231458_at	100507629	LINC00658	long intergenic non-protein coding RNA 658
208410_x_at	265	AMELX	amelogenin, X-linked
219836_at	79413	ZBED2	zinc finger, BED-type containing 2
231683_at	10249	GLYAT	glycine-N-acyltransferase
236965_at	143630	UBQLNL	ubiquilin-like
219139_s_at	114819	CROCCP3	ciliary rootlet coiled-coil, rootletin pseudogene 3
224088_at	56923	NMUR2	neuromedin U receptor 2
235799_at	25936	NSL1	NSL1, MIS12 kinetochore complex component
219969_at	55787	TXLNG	taxilin gamma
214524_at	2691	GHRH	growth hormone releasing hormone
1557787_at	101929153	LOC101929153	uncharacterized LOC101929153
1553211_at	162282	ANKFN1	ankyrin-repeat and fibronectin type III domain containing 1
1563770_at	728116	ZBTB8B	zinc finger and BTB domain containing 8B
208165_s_at	10279	PRSS16	protease, serine, 16 (thymus)
243710_at	100506175	LOC100506175	uncharacterized LOC100506175

238148_s_at	390963	ZNF818P	zinc finger protein 818, pseudogene
1562081_a_at	100874182	LINC00424	long intergenic non-protein coding RNA 424
1570474_s_at	338579	ANKRD30BP3	ankyrin repeat domain 30B pseudogene 3
220048_at	10913	EDAR	ectodysplasin A receptor
1560513_at	400568	LOC400568	uncharacterized LOC400568
242378_at	255275	MYADML2	myeloid-associated differentiation marker-like 2
1553130_at	652276	LOC652276	potassium channel tetramerization domain containing 5 pseudogene
244615_x_at	123283	TARSL2	threonyl-tRNA synthetase-like 2
1561233_at	283387	LOC283387	uncharacterized LOC283387
231441_at	219557	C7orf62	chromosome 7 open reading frame 62
231447_at	440757	LINC00851	long intergenic non-protein coding RNA 851
230995_at	134147	CMBL	carboxymethylenebutenolidase homolog (Pseudomonas)
221330_at	1129	CHRM2	cholinergic receptor, muscarinic 2
1552396_at	140870	WFDC6	WAP four-disulfide core domain 6
241118_at	100129597	LINC00462	long intergenic non-protein coding RNA 462
235468_at	146713	RBFOX3	RNA binding protein, fox-1 homolog (C. elegans) 3
207452_s_at	53942	CNTN5	contactin 5
1570182_at	101927539	LOC101927539	uncharacterized LOC101927539
221520_s_at	55143	CDCA8	cell division cycle associated 8
208137_x_at	81856	ZNF611	zinc finger protein 611
1555133_at	171482	FAM9A	family with sequence similarity 9, member A
204269_at	11040	PIM2	Pim-2 proto-oncogene, serine/threonine kinase
1559083_x_at	284600	LOC284600	uncharacterized LOC284600
236351_at	389023	DPP10-AS1	DPP10 antisense RNA 1
233092_s_at	101060157	LOC101060157	uncharacterized LOC101060157
230011_at	150365	MEI1	meiosis inhibitor 1
1569990_at	11165	NUDT3	nudix (nucleoside diphosphate linked moiety X)-type motif 3
207485_x_at	11119	BTN3A1	butyrophilin, subfamily 3, member A1
230510_at	94086	HSPB9	heat shock protein, alpha-crystallin-related, B9
1563571_at	285463	CTBP1-AS	CTBP1 antisense RNA
209957_s_at	4878	NPPA	natriuretic peptide A
1552745_at	133482	SLCO6A1	solute carrier organic anion transporter family, member 6A1
219936_s_at	53836	GPR87	G protein-coupled receptor 87
1564392_at	387486	LINC00320	long intergenic non-protein coding RNA 320
1557651_x_at	2582	GALE	UDP-galactose-4-epimerase
216298_at	445347	TARP	TCR gamma alternate reading frame protein
224408_at	84539	MCHR2	melanin-concentrating hormone receptor 2
214615_at	27334	P2RY10	purinergic receptor P2Y, G-protein coupled, 10
213990_s_at	57144	PAK7	p21 protein (Cdc42/Rac)-activated kinase 7
240729_at	131831	ERICH6	glutamate-rich 6
207210_at	2556	GABRA3	gamma-aminobutyric acid (GABA) A receptor, alpha 3
233250_x_at	80020	FOXRED2	FAD-dependent oxidoreductase domain containing 2
221329_at	23538	OR52A1	olfactory receptor, family 52, subfamily A, member 1
236295_s_at	197358	NLRC3	NLR family, CARD domain containing 3
217589_at	142684	RAB40A	RAB40A, member RAS oncogene family
207201_s_at	6580	SLC22A1	solute carrier family 22 (organic cation transporter), member 1
1553423_a_at	146857	SLFN13	schlafen family member 13
210477_x_at	5599	MAPK8	mitogen-activated protein kinase 8
231734_at	5948	RBP2	retinol binding protein 2, cellular
217305_s_at	55811	ADCY10	adenylate cyclase 10 (soluble)
1562226_at	221806	VWDE	von Willebrand factor D and EGF domains
1569879_a_at	84465	MEGF11	multiple EGF-like-domains 11
220446_s_at	10164	CHST4	carbohydrate (N-acetylglucosamine 6-O) sulfotransferase 4
1561850_at	100133669	LOC100133669	uncharacterized LOC100133669
233498_at	2066	ERBB4	v-erb-b2 avian erythroblastic leukemia viral oncogene homolog 4
205317_s_at	6565	SLC15A2	solute carrier family 15 (oligopeptide transporter), member 2
1562337_at	162998	OR7D2	olfactory receptor, family 7, subfamily D, member 2
238222_at	200504	GKN2	gastrokine 2
220252_x_at	80231	CXorf21	chromosome X open reading frame 21
1553136_at	130535	KCTD18	potassium channel tetramerization domain containing 18
1557611_at	9654	TTLL4	tubulin tyrosine ligase-like family, member 4
214822_at	57795	BRINP2	bone morphogenetic protein/retinoic acid inducible neural-specific 2

207237_at	3738	KCNA3	potassium voltage-gated channel, shaker-related subfamily, member 3
1558846_at	119548	PNLIPRP3	pancreatic lipase-related protein 3
214088_s_at	2525	FUT3	fucosyltransferase 3 (galactoside 3(4)-L-fucosyltransferase, Lewis blood group)
207718_x_at	1549	CYP2A7	cytochrome P450, family 2, subfamily A, polypeptide 7
219562_at	25837	RAB26	RAB26, member RAS oncogene family
215129_at	5288	PIK3C2G	phosphatidylinositol-4-phosphate 3-kinase, catalytic subunit type 2 gamma
1567058_at	26492	OR8G2	olfactory receptor, family 8, subfamily G, member 2
1566931_at	64216	TFB2M	transcription factor B2, mitochondrial
210496_at	57051	NAG18	NAG18 mRNA
1552456_a_at	125997	MBD3L2	methyl-CpG binding domain protein 3-like 2
210730_s_at	4887	NPY2R	neuropeptide Y receptor Y2
1557886_at	201134	CEP112	centrosomal protein 112kDa
1560522_at	201477	DLGAP1-AS3	DLGAP1 antisense RNA 3
219851_at	79898	ZNF613	zinc finger protein 613
1563793_at	100130278	LOC100130278	uncharacterized LOC100130278
1562595_at	101928314	LOC101928314	uncharacterized LOC101928314
1561061_at	729506	LOC729506	uncharacterized LOC729506
220390_at	79841	AGBL2	ATP/GTP binding protein-like 2
206666_at	3003	GZMK	granzyme K (granzyme 3; tryptase II)
234410_at	200959	GABRR3	gamma-aminobutyric acid (GABA) A receptor, rho 3
237428_at	389320	TEX43	testis expressed 43
1555056_at	901	CCNG2	cyclin G2
1553138_a_at	126549	ANKLE1	ankyrin repeat and LEM domain containing 1
220242_x_at	55762	ZNF701	zinc finger protein 701
214947_at	54491	FAM105A	family with sequence similarity 105, member A
220796_x_at	79939	SLC35E1	solute carrier family 35, member E1
1561044_at	101928973	LOC101928973	uncharacterized LOC101928973
1570285_at	101927851	LOC101927851	uncharacterized LOC101927851
223812_at	51252	FAM178B	family with sequence similarity 178, member B
205901_at	5368	PNOC	prepronociceptin
220536_at	55237	VRTN	vertebrae development associated
1553905_at	170063	CXorf22	chromosome X open reading frame 22
1561928_s_at	389161	ANKUB1	ankyrin repeat and ubiquitin domain containing 1
220520_s_at	54830	NUP62CL	nucleoporin 62kDa C-terminal like
220813_at	57105	CYSLTR2	cysteinyl leukotriene receptor 2
205367_at	10603	SH2B2	SH2B adaptor protein 2
244694_at	402665	IGLON5	IgLON family member 5
218865_at	64757	MARC1	mitochondrial amidoxime reducing component 1
239929_at	148811	PM20D1	peptidase M20 domain containing 1
220590_at	55846	ITFG2	integrin alpha FG-GAP repeat containing 2
217711_at	7010	TEK	TEK tyrosine kinase, endothelial
232521_at	9159	PCSK7	proprotein convertase subtilisin/kexin type 7
237266_at	100289509	KCNIP2-AS1	KCNIP2 antisense RNA 1
230973_at	400745	SH2D5	SH2 domain containing 5
215341_at	1768	DNAH6	dynein, axonemal, heavy chain 6
1555457_at	1620	BRINP1	bone morphogenetic protein/retinoic acid inducible neural-specific 1
240373_at	58509	CACTIN	cactin, spliceosome C complex subunit
221910_at	2115	ETV1	ets variant 1
226070_at	286257	C9orf142	chromosome 9 open reading frame 142
1569637_at	728927	ZNF736	zinc finger protein 736
243954_at	285286	LINC00877	long intergenic non-protein coding RNA 877
210431_at	251	ALPPL2	alkaline phosphatase, placental-like 2
217192_s_at	639	PRDM1	PR domain containing 1, with ZNF domain
1555478_at	388407	C17orf82	chromosome 17 open reading frame 82
213248_at	730101	LOC730101	uncharacterized LOC730101
231404_at	100506540	SPTY2D1-AS1	SPTY2D1 antisense RNA 1
207533_at	6346	CCL1	chemokine (C-C motif) ligand 1
1554349_at	91419	XRCC6BP1	XRCC6 binding protein 1
1558770_a_at	146850	PIK3R6	phosphoinositide-3-kinase, regulatory subunit 6
231333_at	100505515	LOC100505515	uncharacterized LOC100505515
229777_at	119467	CLRN3	clarin 3

1552948_at	148870	CCDC27	coiled-coil domain containing 27
236214_at	84691	FAM71F1	family with sequence similarity 71, member F1
233850_s_at	57593	EBF4	early B-cell factor 4
1570085_at	100506679	LOC100506679	uncharacterized LOC100506679
237598_at	653677	SEC1P	secretory blood group 1, pseudogene
230572_at	152641	WWC2-AS2	WWC2 antisense RNA 2
1559785_at	122664	TPPP2	tubulin polymerization-promoting protein family member 2
1559593_a_at	9441	MED26	mediator complex subunit 26
1553499_s_at	327657	SERPINA9	serpin peptidase inhibitor, clade A (alpha-1 antiproteinase, antitrypsin), member 9
209829_at	9750	FAM65B	family with sequence similarity 65, member B
1566455_at	100507494	LOC100507494	uncharacterized LOC100507494
1556768_at	100144604	LINC00930	long intergenic non-protein coding RNA 930
207509_s_at	3904	LAIR2	leukocyte-associated immunoglobulin-like receptor 2
1558661_at	100506207	LOC100506207	uncharacterized LOC100506207
1563171_at	101927422	GNA14-AS1	GNA14 antisense RNA 1
1560790_at	283685	GOLGA6L2	golgin A6 family-like 2
1552389_at	203111	ERICH5	glutamate-rich 5
214858_at	100130449	PP14571	uncharacterized LOC100130449
233835_at	90246	LOC90246	uncharacterized LOC90246
227134_at	84958	SYTL1	synaptotagmin-like 1
1570127_at	101929144	LOC101929144	uncharacterized LOC101929144
91580_at	57408	LRTM1	leucine-rich repeats and transmembrane domains 1
240784_at	375607	NAT16	N-acetyltransferase 16 (GCN5-related, putative)
208035_at	2916	GRM6	glutamate receptor, metabotropic 6
224099_at	90134	KCNH7	potassium voltage-gated channel, subfamily H (eag-related), member 7
203901_at	10454	TAB1	TGF-beta activated kinase 1/MAP3K7 binding protein 1
204950_at	22900	CARD8	caspase recruitment domain family, member 8
242567_at	80325	ABTB1	ankyrin repeat and BTB (POZ) domain containing 1
206926_s_at	3589	IL11	interleukin 11
225107_at	3181	HNRNPA2B1	heterogeneous nuclear ribonucleoprotein A2/B1
216828_at	140678	MLLT10P1	myeloid/lymphoid or mixed-lineage leukemia (trithorax homolog, Drosophila); translocated to, 10 pseudogene 1
217148_x_at	3537	IGLC1	immunoglobulin lambda constant 1 (Mcg marker)
233194_at	100874241	STARD13-AS	STARD13 antisense RNA
216875_x_at	55547	HAB1	B1 for mucin
1553310_at	151056	PLB1	phospholipase B1
209720_s_at	6317	SERPINB3	serpin peptidase inhibitor, clade B (ovalbumin), member 3
227002_at	286336	FAM78A	family with sequence similarity 78, member A
235817_at	202915	TMEM184A	transmembrane protein 184A
217308_at	26184	OR1F2P	olfactory receptor, family 1, subfamily F, member 2
220090_at	49860	CRNN	cornulin
231792_at	85366	MYLK2	myosin light chain kinase 2
220498_at	10880	ACTL7B	actin-like 7B
236493_at	158801	NKAPP1	NFKB activating protein pseudogene 1
244789_at	387718	TEX36	testis expressed 36
207892_at	959	CD40LG	CD40 ligand
217594_at	23318	ZCCHC11	zinc finger, CCHC domain containing 11
226147_s_at	5284	PIGR	polymeric immunoglobulin receptor
1557682_a_at	284688	LINC01142	long intergenic non-protein coding RNA 1142
205544_s_at	1380	CR2	complement component (3d/Epstein Barr virus) receptor 2
235583_at	286676	ILDR1	immunoglobulin-like domain containing receptor 1
1553478_at	283165	KIRREL3-AS3	KIRREL3 antisense RNA 3
1564160_at	401124	DTHD1	death domain containing 1
236152_at	90737	PAGE5	P antigen family, member 5 (prostate associated)
1562587_at	116449	CLNK	cytokine-dependent hematopoietic cell linker
231814_at	10071	MUC12	mucin 12, cell surface associated
235455_at	348487	FAM131C	family with sequence similarity 131, member C
206802_at	5079	PAX5	paired box 5
236496_at	123099	DEGS2	delta(4)-desaturase, sphingolipid 2
1564439_a_at	283303	MRGPRG-AS1	MRGPRG antisense RNA 1
223874_at	653857	ACTR3C	ARP3 actin-related protein 3 homolog C (yeast)

1561271_at	348254	CCDC144CP	coiled-coil domain containing 144C, pseudogene
1569841_x_at	101927798	LOC101927798	uncharacterized LOC101927798
228358_at	6666	SOX12	SRY (sex determining region Y)-box 12
215465_at	26154	ABCA12	ATP-binding cassette, sub-family A (ABC1), member 12
234049_at	286042	FAM86B3P	family with sequence similarity 86, member A pseudogene
1567286_at	26338	OR5L2	olfactory receptor, family 5, subfamily L, member 2
1561125_at	25902	MTHFD1L	methylenetetrahydrofolate dehydrogenase (NADP+ dependent) 1-like
208548_at	3443	IFNA6	interferon, alpha 6
1554890_a_at	7072	TIA1	TIA1 cytotoxic granule-associated RNA binding protein
209848_s_at	6490	PMEL	premelanosome protein
207097_s_at	10246	SLC17A2	solute carrier family 17, member 2
1556255_a_at	400756	LOC400756	uncharacterized LOC400756
240320_at	100507650	RNF212B	ring finger protein 212B
234071_at	64798	DEPTOR	DEP domain containing MTOR-interacting protein
226097_at	252995	FNDC5	fibronectin type III domain containing 5
211419_s_at	1124	CHN2	chimerin 2
223517_at	93611	FBXO44	F-box protein 44
1561531_at	100874035	PLCH1-AS1	PLCH1 antisense RNA 1
226530_at	90427	BMF	Bcl2 modifying factor
233730_at	57579	FAM135A	family with sequence similarity 135, member A
1562943_at	100379174	MACROD2-AS1	MACROD2 antisense RNA 1
232443_at	441052	LOC441052	uncharacterized LOC441052
228705_at	147968	CAPN12	calpain 12
1566844_at	168741	PER4	period circadian clock 3 pseudogene
1555348_at	339488	TFAP2E	transcription factor AP-2 epsilon (activating enhancer binding protein 2 epsilon)
1561544_at	101927058	LOC101927058	uncharacterized LOC101927058
232192_at	153811	LOC153811	uncharacterized LOC153811
1552493_s_at	1584	CYP11B1	cytochrome P450, family 11, subfamily B, polypeptide 1
214493_s_at	10207	INADL	InaD-like (Drosophila)
243836_at	285175	UNC80	unc-80 homolog (C. elegans)
1554803_s_at	493829	TRIM72	tripartite motif containing 72, E3 ubiquitin protein ligase
240162_at	100506791	LINC01511	long intergenic non-protein coding RNA 1511
223891_at	56479	KCNQ5	potassium voltage-gated channel, KQT-like subfamily, member 5
221413_at	9196	KCNAB3	potassium voltage-gated channel, shaker-related subfamily, beta member 3
236695_at	100505826	STK4-AS1	STK4 antisense RNA 1 (head to head)
235504_at	64388	GREM2	gremlin 2, DAN family BMP antagonist
232111_at	100507043	TUNAR	TCL1 upstream neural differentiation-associated RNA
227182_at	203328	SUSD3	sushi domain containing 3
1555627_s_at	84536	C21orf67	chromosome 21 open reading frame 67
1553927_at	202865	C7orf33	chromosome 7 open reading frame 33
223327_x_at	80154	GOLGA2P10	golgin A2 pseudogene 10
1553472_at	150596	FLJ32955	uncharacterized protein FLJ32955
219741_x_at	79818	ZNF552	zinc finger protein 552
205733_at	641	BLM	Bloom syndrome, RecQ helicase-like
234102_at	65997	RASL11B	RAS-like, family 11, member B
230367_at	219537	SMTNL1	smoothelin-like 1
220720_x_at	80097	MZT2B	mitotic spindle organizing protein 2B
214382_at	54346	UNC93A	unc-93 homolog A (C. elegans)
232891_at	128646	SIRPD	signal-regulatory protein delta
220855_at	100506863	CLTC-IT1	CLTC intronic transcript 1 (non-protein coding)
217160_at	7258	TSPY1	testis specific protein, Y-linked 1
204885_s_at	10232	MSLN	mesothelin
1559790_at	646241	LOC646241	uncharacterized LOC646241
1558711_at	285512	FAM13A-AS1	FAM13A antisense RNA 1
1552950_at	161502	C15orf26	chromosome 15 open reading frame 26
231162_at	125875	CLDND2	claudin domain containing 2
1565866_a_at	100381270	ZBED6	zinc finger, BED-type containing 6
1559405_a_at	55503	TRPV6	transient receptor potential cation channel, subfamily V, member 6
1562383_at	100423035	MIR4313	microRNA 4313
221110_x_at	50940	PDE11A	phosphodiesterase 11A
211778_s_at	58495	OVOL2	ovo-like zinc finger 2

229927_at	93273	LEMD1	LEM domain containing 1
244577_at	102724009	LOC102724009	uncharacterized LOC102724009
1555746_at	974	CD79B	CD79b molecule, immunoglobulin-associated beta
233298_at	728591	CCDC169	coiled-coil domain containing 169
1560810_at	101927735	LOC101927735	uncharacterized LOC101927735
230985_at	138724	C9orf131	chromosome 9 open reading frame 131
215527_at	202559	KHDRBS2	KH domain containing, RNA binding, signal transduction associated 2
240967_at	337970	KRTAP19-3	keratin associated protein 19-3
231434_at	100505841	LOC100505841	zinc finger protein 474-like
205402_x_at	5645	PRSS2	protease, serine, 2 (trypsin 2)
208188_at	3857	KRT9	keratin 9
209851_at	23091	ZC3H13	zinc finger CCCH-type containing 13
230191_at	84630	TTBK1	tau tubulin kinase 1
1562017_at	100130654	LOC100130654	uncharacterized LOC100130654
215336_at	11215	AKAP11	A kinase (PRKA) anchor protein 11
1557060_at	148756	OVAAL	ovarian adenocarcinoma amplified long non-coding RNA
1553235_at	56135	PCDHAC1	protocadherin alpha subfamily C, 1
1560673_at	101929591	KIZ-AS1	KIZ antisense RNA 1
1560035_at	285093	RTP5	receptor (chemosensory) transporter protein 5 (putative)
1552991_at	120065	OR5P2	olfactory receptor, family 5, subfamily P, member 2
236863_at	339210	C17orf67	chromosome 17 open reading frame 67
1567270_at	26242	OR4C1P	olfactory receptor, family 4, subfamily C, member 1 pseudogene
1554875_at	79823	CAMKMT	calmodulin-lysine N-methyltransferase
232965_at	400684	LOC400684	uncharacterized LOC400684
1563658_a_at	143425	SYT9	synaptotagmin IX
220805_at	3274	HRH2	histamine receptor H2
1559167_x_at	255027	MPV17L	MPV17 mitochondrial membrane protein-like
1568884_at	27000	DNAJC2	DnaJ (Hsp40) homolog, subfamily C, member 2
204949_at	3385	ICAM3	intercellular adhesion molecule 3
1555978_s_at	10627	MYL12A	myosin, light chain 12A, regulatory, non-sarcomeric
213261_at	9881	TRANK1	tetratricopeptide repeat and ankyrin repeat containing 1
220659_s_at	55262	C7orf43	chromosome 7 open reading frame 43
1561940_at	100128843	LOC100128843	uncharacterized LOC100128843
235048_at	26049	FAM169A	family with sequence similarity 169, member A
226912_at	254887	ZDHHC23	zinc finger, DHHC-type containing 23
217101_at	22996	TTC39A	tetratricopeptide repeat domain 39A
211222_s_at	9001	HAP1	huntingtin-associated protein 1
238383_at	352999	C6orf58	chromosome 6 open reading frame 58
1559298_a_at	388182	SPATA41	spermatogenesis associated 41 (non-protein coding)
207885_at	795	S100G	S100 calcium binding protein G
1561029_at	101927821	LINC01425	long intergenic non-protein coding RNA 1425
224262_at	84639	IL1F10	interleukin 1 family, member 10 (theta)
204196_x_at	5316	PKNOX1	PBX/knotted 1 homeobox 1
205828_at	4314	MMP3	matrix metallopeptidase 3 (stromelysin 1, progelatinase)
1553708_at	84847	LINC00525	long intergenic non-protein coding RNA 525
220561_at	51214	IGF2-AS	IGF2 antisense RNA
233157_x_at	93233	CCDC114	coiled-coil domain containing 114
220197_at	50617	ATP6V0A4	ATPase, H+ transporting, lysosomal V0 subunit a4
1554744_at	114769	CARD16	caspase recruitment domain family, member 16
208000_at	2765	GML	glycosylphosphatidylinositol anchored molecule like
240228_at	114788	CSMD3	CUB and Sushi multiple domains 3
1555044_a_at	131377	KLHL40	kelch-like family member 40
1559514_at	100132077	LOC100132077	uncharacterized LOC100132077
232105_at	101669762	BLACAT1	bladder cancer associated transcript 1 (non-protein coding)
214461_at	3929	LBP	lipopolysaccharide binding protein
1563265_at	101927219	HNF4A-AS1	HNF4A antisense RNA 1
233534_at	83897	KRTAP3-2	keratin associated protein 3-2
214357_at	92346	C1orf105	chromosome 1 open reading frame 105
1556533_at	283994	LINC00868	long intergenic non-protein coding RNA 868
244344_at	65266	WNK4	WNK lysine deficient protein kinase 4
214523_at	1053	CEBPE	CCAAT/enhancer binding protein (C/EBP), epsilon
216916_s_at	9228	DLGAP2	discs, large (Drosophila) homolog-associated protein 2

1564242_at	101928607	TRPM2-AS	TRPM2 antisense RNA
227819_at	59352	LGR6	leucine-rich repeat containing G protein-coupled receptor 6
1559311_at	643210	EHMT1-IT1	EHMT1 intronic transcript 1
1554356_at	84296	GINS4	GINS complex subunit 4 (Sld5 homolog)
36865_at	23357	ANGEL1	angel homolog 1 (Drosophila)
232462_s_at	503538	A1BG-AS1	A1BG antisense RNA 1
209680_s_at	3833	KIFC1	kinesin family member C1
228984_at	57571	CARNS1	carnosine synthase 1
220394_at	26281	FGF20	fibroblast growth factor 20
1553523_at	338323	NLRP14	NLR family, pyrin domain containing 14
212699_at	192683	SCAMP5	secretory carrier membrane protein 5
203879_at	5293	PIK3CD	phosphatidylinositol-4,5-bisphosphate 3-kinase, catalytic subunit delta
236666_s_at	390205	LRRC10B	leucine rich repeat containing 10B
218960_at	56649	TMPRSS4	transmembrane protease, serine 4
224119_at	101928604	ZBTB46-AS1	ZBTB46 antisense RNA 1
1554895_a_at	54933	RHBDL2	rhomboid, veinlet-like 2 (Drosophila)
244374_at	257000	TINCR	tissue differentiation-inducing non-protein coding RNA
233590_at	100506457	LOC100506457	uncharacterized LOC100506457
1564149_at	102723927	LOC102723927	uncharacterized LOC102723927
221081_s_at	79961	DENND2D	DENN/MADD domain containing 2D
1557677_a_at	339976	TRIML1	tripartite motif family-like 1
1553482_at	145858	C15orf32	chromosome 15 open reading frame 32
1560565_at	101929082	LINC01333	long intergenic non-protein coding RNA 1333
1554476_x_at	388558	ZNF808	zinc finger protein 808
208262_x_at	4210	MEFV	Mediterranean fever
211184_s_at	10083	USH1C	Usher syndrome 1C (autosomal recessive, severe)
204684_at	4884	NPTX1	neuronal pentraxin I
237529_at	100507143	LINC00708	long intergenic non-protein coding RNA 708
1567238_at	26246	OR2L2	olfactory receptor, family 2, subfamily L, member 2
212592_at	3512	IGJ	immunoglobulin J polypeptide, linker protein for immunoglobulin alpha and mu polypeptides
210521_s_at	26998	FETUB	fetuin B
219272_at	55223	TRIM62	tripartite motif containing 62
221391_at	50840	TAS2R14	taste receptor, type 2, member 14
234486_at	79345	OR51B2	olfactory receptor, family 51, subfamily B, member 2
215685_s_at	1746	DLX2	distal-less homeobox 2
1553471_at	146861	SLC35G3	solute carrier family 35, member G3
236036_at	100506343	FAM212B-AS1	FAM212B antisense RNA 1
1552592_at	118856	MMP21	matrix metallopeptidase 21
1559884_at	100048912	CDKN2B-AS1	CDKN2B antisense RNA 1
207462_at	2742	GLRA2	glycine receptor, alpha 2
1552675_at	150353	DNAJB7	DnaJ (Hsp40) homolog, subfamily B, member 7
1561481_at	101928435	LOC101928435	uncharacterized LOC101928435
205693_at	7140	TNNT3	troponin T type 3 (skeletal, fast)
244681_at	255330	NUP210P1	nucleoporin 210kDa pseudogene 1
219442_at	100132341	CLUHP3	clustered mitochondria (cluA/CLU1) homolog pseudogene 3
242633_x_at	7627	ZNF75A	zinc finger protein 75a
1561459_at	101929526	LOC101929526	uncharacterized LOC101929526
1563787_a_at	285782	CAGE1	cancer antigen 1
239691_at	196415	C12orf77	chromosome 12 open reading frame 77
237905_at	147183	KRT25	keratin 25
231982_at	284422	SMIM24	small integral membrane protein 24
1569537_at	101928922	LINC00463	long intergenic non-protein coding RNA 463
231492_at	101060004	LINC01523	long intergenic non-protein coding RNA 1523
1565666_s_at	4588	MUC6	mucin 6, oligomeric mucus/gel-forming
217147_s_at	50852	TRAT1	T cell receptor associated transmembrane adaptor 1
232766_at	140836	BANF2	barrier to autointegration factor 2
230236_at	732253	TDRG1	testis development related 1 (non-protein coding)
1557777_at	5158	PDE6B	phosphodiesterase 6B, cGMP-specific, rod, beta
215821_x_at	5671	PSG3	pregnancy specific beta-1-glycoprotein 3
213680_at	3854	KRT6B	keratin 6B
226670_s_at	80336	PABPC1L	poly(A) binding protein, cytoplasmic 1-like

1562783_at	100128840	LOC100128840	uncharacterized LOC100128840
228004_at	140828	LINC00261	long intergenic non-protein coding RNA 261
226549_at	388228	SBK1	SH3 domain binding kinase 1
208377_s_at	778	CACNA1F	calcium channel, voltage-dependent, L type, alpha 1F subunit
220478_at	80831	APOL5	apolipoprotein L, 5
221289_at	1750	DLX6	distal-less homeobox 6
229039_at	6854	SYN2	synapsin II
1564209_at	282980	LINC00700	long intergenic non-protein coding RNA 700
1552615_at	32	ACACB	acetyl-CoA carboxylase beta
217979_at	27075	TSPAN13	tetraspanin 13
207251_at	4225	MEP1B	meprin A, beta
207197_at	7547	ZIC3	Zic family member 3
210393_at	8549	LGR5	leucine-rich repeat containing G protein-coupled receptor 5
1554173_at	124599	CD300LB	CD300 molecule-like family member b
223840_s_at	83890	SPATA9	spermatogenesis associated 9
1555557_a_at	10188	TNK2	tyrosine kinase, non-receptor, 2
1563610_at	157273	LOC157273	uncharacterized LOC157273
1557613_at	285352	KIF9-AS1	KIF9 antisense RNA 1
214816_x_at	91442	C19orf40	chromosome 19 open reading frame 40
1554941_at	57565	KLHL14	kelch-like family member 14
210182_at	1325	CORT	cortistatin
216407_at	55697	VAC14	Vac14 homolog (S. cerevisiae)
1553087_at	84322	C18orf12	chromosome 18 open reading frame 12
216998_s_at	255926	ADAM5	ADAM metallopeptidase domain 5, pseudogene
1554749_s_at	1188	CLCNKB	chloride channel, voltage-sensitive Kb
1570065_at	348645	C22orf34	chromosome 22 open reading frame 34
206256_at	1369	CPN1	carboxypeptidase N, polypeptide 1
234529_at	64002	PCGEM1	PCGEM1, prostate-specific transcript (non-protein coding)
215208_x_at	6165	RPL35A	ribosomal protein L35a
205832_at	51200	CPA4	carboxypeptidase A4
211353_at	26103	LRIT1	leucine-rich repeat, immunoglobulin-like and transmembrane domains 1
213475_s_at	3683	ITGAL	integrin, alpha L (antigen CD11A (p180), lymphocyte function-associated antigen 1; alpha polypeptide)
230398_at	84951	TNS4	tensin 4
233522_at	101929423	MEF2C-AS1	MEF2C antisense RNA 1
1561682_at	100874314	TTLL7-IT1	TTLL7 intronic transcript 1 (non-protein coding)
232740_at	114044	MCM3AP-AS1	MCM3AP antisense RNA 1
1561454_at	101929123	LOC101929123	uncharacterized LOC101929123
206692_at	3766	KCNJ10	potassium inwardly-rectifying channel, subfamily J, member 10
220944_at	57115	PGLYRP4	peptidoglycan recognition protein 4
238173_at	6919	TCEA2	transcription elongation factor A (SII), 2
219739_at	54546	RNF186	ring finger protein 186
221080_s_at	79958	DENND1C	DENN/MADD domain containing 1C
204733_at	5653	KLK6	kallikrein-related peptidase 6
203147_s_at	9830	TRIM14	tripartite motif containing 14
229196_at	63901	FAM111A	family with sequence similarity 111, member A
1562632_at	285191	LOC285191	uncharacterized LOC285191
238136_at	729956	SHISA7	shisa family member 7
1570447_at	643365	LINC00452	long intergenic non-protein coding RNA 452
207906_at	3562	IL3	interleukin 3
234370_at	340547	VSIG1	V-set and immunoglobulin domain containing 1
208212_s_at	238	ALK	anaplastic lymphoma receptor tyrosine kinase
1562829_at	339568	LOC339568	uncharacterized LOC339568
219243_at	55303	GIMAP4	GTPase, IMAP family member 4
1553357_at	158696	LINC00889	long intergenic non-protein coding RNA 889
1559828_at	170370	FAM170B	family with sequence similarity 170, member B
1562048_at	152225	LOC152225	uncharacterized LOC152225
211841_s_at	8718	TNFRSF25	tumor necrosis factor receptor superfamily, member 25
206557_at	79986	ZNF702P	zinc finger protein 702, pseudogene
233343_at	140850	DEFB127	defensin, beta 127
220051_at	10942	PRSS21	protease, serine, 21 (testisin)
1554125_a_at	22829	NLGN4Y	neuroligin 4, Y-linked

1552568_at	83597	RTP3	receptor (chemosensory) transporter protein 3
220435_at	55532	SLC30A10	solute carrier family 30, member 10
233942_at	613126	CYP51A1-AS1	CYP51A1 antisense RNA 1 (head to head)
1554847_at	525	ATP6V1B1	ATPase, H+ transporting, lysosomal 56/58kDa, V1 subunit B1
211405_x_at	3451	IFNA17	interferon, alpha 17
1560081_at	100505648	RAD51-AS1	RAD51 antisense RNA 1 (head to head)
1553157_at	89884	LHX4	LIM homeobox 4
1557827_at	414245	DNAJC9-AS1	DNAJC9 antisense RNA 1
207425_s_at	10801	SEPT9	septin 9
1552775_at	222545	GPRC6A	G protein-coupled receptor, class C, group 6, member A
215151_at	55619	DOCK10	dedicator of cytokinesis 10
233252_s_at	55342	STRBP	spermatid perinuclear RNA binding protein
1561249_a_at	100128285	DNM1P35	DNM1 pseudogene 35
220336_s_at	51206	GP6	glycoprotein VI (platelet)
1560862_at	101929684	LINC01520	long intergenic non-protein coding RNA 1520
1556178_x_at	129685	TAF8	TAF8 RNA polymerase II, TATA box binding protein (TBP)-associated factor, 43kDa
220114_s_at	55576	STAB2	stabilin 2
205639_at	313	AOAH	acyloxyacyl hydrolase (neutrophil)
1569453_a_at	692247	LOC692247	uncharacterized LOC692247
236674_at	388780	LOC388780	uncharacterized LOC388780
1554629_at	2045	EPHA7	EPH receptor A7
237282_s_at	158798	AKAP14	A kinase (PRKA) anchor protein 14
1555224_at	554201	CCDC148-AS1	CCDC148 antisense RNA 1
208360_s_at	51359	ERVH-4	endogenous retrovirus group H, member 4
213017_at	171586	ABHD3	abhydrolase domain containing 3
220838_at	54932	EXD3	exonuclease 3'-5' domain containing 3
1552522_at	201798	TIGD4	tigger transposable element derived 4
217495_x_at	796	CALCA	calcitonin-related polypeptide alpha
209760_at	23240	KIAA0922	KIAA0922
1562802_at	100885798	LINC00210	long intergenic non-protein coding RNA 210
234284_at	94235	GNG8	guanine nucleotide binding protein (G protein), gamma 8
205983_at	1800	DPEP1	dipeptidase 1 (renal)
205948_at	11122	PTPRT	protein tyrosine phosphatase, receptor type, T
224554_at	2693	GHSR	growth hormone secretagogue receptor
206842_at	3750	KCND1	potassium voltage-gated channel, Shal-related subfamily, member 1
244795_at	55245	UQCC1	ubiquinol-cytochrome c reductase complex assembly factor 1
233941_at	145497	LRRC74A	leucine rich repeat containing 74A
240167_at	152742	LINC01085	long intergenic non-protein coding RNA 1085
1561455_at	284294	LOC284294	uncharacterized LOC284294
219934_s_at	6783	SULT1E1	sulfotransferase family 1E, estrogen-preferring, member 1
210560_at	2637	GBX2	gastrulation brain homeobox 2
220611_at	1600	DAB1	Dab, reelin signal transducer, homolog 1 (Drosophila)
207303_at	5137	PDE1C	phosphodiesterase 1C, calmodulin-dependent 70kDa
1556558_s_at	285266	ENTPD3-AS1	ENTPD3 antisense RNA 1
1553586_at	283932	FBXL19-AS1	FBXL19 antisense RNA 1 (head to head)
205531_s_at	27165	GLS2	glutaminase 2 (liver, mitochondrial)
207444_at	9390	SLC22A13	solute carrier family 22 (organic anion/urate transporter), member 13
205645_at	9185	REPS2	RALBP1 associated Eps domain containing 2
240301_at	151871	DPPA2	developmental pluripotency associated 2
230924_at	284076	TTLL6	tubulin tyrosine ligase-like family, member 6
234918_at	29997	GLTSCR2	glioma tumor suppressor candidate region gene 2
231928_at	54626	HES2	hes family bHLH transcription factor 2
211166_at	285596	FAM153A	family with sequence similarity 153, member A
242970_at	57609	DIP2B	DIP2 disco-interacting protein 2 homolog B (Drosophila)
1553181_at	64794	DDX31	DEAD (Asp-Glu-Ala-Asp) box polypeptide 31
238245_at	339221	ENPP7	ectonucleotide pyrophosphatase/phosphodiesterase 7
1552540_s_at	115811	IQCD	IQ motif containing D
229566_at	645638	WFDC21P	WAP four-disulfide core domain 21, pseudogene
223556_at	3070	HELLS	helicase, lymphoid-specific
237425_at	100505890	SORCS3-AS1	SORCS3 antisense RNA 1
1559180_at	100131860	LOC100131860	uncharacterized LOC100131860

1556377_s_at	4008	LMO7	LIM domain 7
1562492_at	340090	LOC340090	uncharacterized LOC340090
1552415_a_at	259240	WFDC9	WAP four-disulfide core domain 9
204116_at	3561	IL2RG	interleukin 2 receptor, gamma
219724_s_at	9840	TESPA1	thymocyte expressed, positive selection associated 1
216690_at	26664	OR7C1	olfactory receptor, family 7, subfamily C, member 1
1567065_at	158131	OR1Q1	olfactory receptor, family 1, subfamily Q, member 1
241224_x_at	84677	DSCR8	Down syndrome critical region gene 8
1553844_a_at	256815	C10orf67	chromosome 10 open reading frame 67
223963_s_at	10644	IGF2BP2	insulin-like growth factor 2 mRNA binding protein 2
231483_at	100505985	LOC100505985	uncharacterized LOC100505985
207059_at	5083	PAX9	paired box 9
1553426_at	285668	C5orf64	chromosome 5 open reading frame 64
1561469_at	101928865	LOC101928865	uncharacterized LOC101928865
239367_at	627	BDNF	brain-derived neurotrophic factor
1560382_at	2549	GAB1	GRB2-associated binding protein 1
219105_x_at	23594	ORC6	origin recognition complex, subunit 6
237234_at	9266	CYTH2	cytohesin 2
1555159_at	157753	TMEM74	transmembrane protein 74
1561270_at	353088	ZNF429	zinc finger protein 429
1569847_at	84952	CGNL1	cingulin-like 1
1568689_at	100631378	LOC100631378	uncharacterized 100631378
210677_at	8435	SOAT2	sterol O-acyltransferase 2
205777_at	1852	DUSP9	dual specificity phosphatase 9
220441_at	79962	DNAJC22	DnaJ (Hsp40) homolog, subfamily C, member 22
1568974_at	100294720	NHEG1	neuroblastoma highly expressed 1
237047_at	26080	FAM230C	family with sequence similarity 230, member C
202507_s_at	6616	SNAP25	synaptosomal-associated protein, 25kDa
1563913_at	101929717	LOC101929717	uncharacterized LOC101929717
204582_s_at	354	KLK3	kallikrein-related peptidase 3
1553690_at	151648	SGOL1	shugoshin-like 1 (S. pombe)
235499_at	728743	LOC728743	zinc finger protein pseudogene
240411_at	133558	MROH2B	maestro heat-like repeat family member 2B
1561030_at	79905	TMC7	transmembrane channel-like 7
1553039_a_at	136371	ASB10	ankyrin repeat and SOCS box containing 10
225488_at	79139	DERL1	derlin 1
234924_s_at	57592	ZNF687	zinc finger protein 687
1563907_at	84253	GARNL3	GTPase activating Rap/RanGAP domain-like 3
210576_at	11283	CYP4F8	cytochrome P450, family 4, subfamily F, polypeptide 8
234835_at	100506667	LOC100506667	uncharacterized LOC100506667
1561253_at	101927901	LOC101927901	uncharacterized LOC101927901
1564403_at	101362076	GVQW1	GVQW motif containing 1
231783_at	1128	CHRM1	cholinergic receptor, muscarinic 1
1560291_at	92129	RIPPLY1	ripply transcriptional repressor 1
211295_x_at	1548	CYP2A6	cytochrome P450, family 2, subfamily A, polypeptide 6
1559079_at	283486	LINC00567	long intergenic non-protein coding RNA 567
212974_at	22898	DENND3	DENN/MADD domain containing 3
1569555_at	9615	GDA	guanine deaminase
214518_at	5161	PDHA2	pyruvate dehydrogenase (lipoamide) alpha 2
232380_at	55277	FGGY	FGGY carbohydrate kinase domain containing
232642_at	90113	VWA5B2	von Willebrand factor A domain containing 5B2
217552_x_at	1378	CR1	complement component (3b/4b) receptor 1 (Knops blood group)
1564310_a_at	165631	PARP15	poly (ADP-ribose) polymerase family, member 15
233775_x_at	100289333	LOC100289333	uncharacterized LOC100289333
220957_at	64693	CTAGE1	cutaneous T-cell lymphoma-associated antigen 1
206939_at	1630	DCC	DCC netrin 1 receptor
220701_at	55451	LINC00216	long intergenic non-protein coding RNA 216
205488_at	3001	GZMA	granzyme A (granzyme 1, cytotoxic T-lymphocyte-associated serine esterase 3)
226205_at	338692	ANKRD13D	ankyrin repeat domain 13 family, member D
1554545_at	403314	APOBEC4	apolipoprotein B mRNA editing enzyme, catalytic polypeptide-like 4 (putative)
1561446_at	101928443	LOC101928443	uncharacterized LOC101928443

207179_at	3195	TLX1	T-cell leukemia homeobox 1
1552932_at	171389	NLRP6	NLR family, pyrin domain containing 6
215334_at	22979	EFR3B	EFR3 homolog B (S. cerevisiae)
230780_at	730091	LINC00886	long intergenic non-protein coding RNA 886
1557465_at	283521	LINC00282	long intergenic non-protein coding RNA 282
206643_at	3034	HAL	histidine ammonia-lyase
215680_at	85368	KIAA1654	KIAA1654 protein
206975_at	4049	LTA	lymphotoxin alpha
1553011_at	138474	TAF1L	TAF1 RNA polymerase II, TATA box binding protein (TBP)-associated factor, 210kDa-like
1561572_at	101929284	LOC101929284	uncharacterized LOC101929284
1556514_at	338809	C12orf74	chromosome 12 open reading frame 74
231433_at	100506929	LOC100506929	uncharacterized LOC100506929
219823_at	79727	LIN28A	lin-28 homolog A (C. elegans)
232061_at	54549	SDK2	sidekick cell adhesion molecule 2
228071_at	168537	GIMAP7	GTPase, IMAP family member 7
1553583_a_at	7069	THRSP	thyroid hormone responsive
207951_at	1447	CSN2	casein beta
1558685_a_at	158960	LOC158960	uncharacterized protein BC009467
1558195_at	283404	LINC00592	long intergenic non-protein coding RNA 592
1556661_at	150384	GTSE1-AS1	GTSE1 antisense RNA 1 (head to head)
1559650_at	100128081	JAZF1-AS1	JAZF1 antisense RNA 1
241828_x_at	339240	KRT17P5	keratin 17 pseudogene 5
1552673_at	222546	RFX6	regulatory factor X, 6
1556954_at	283854	CASC22	cancer susceptibility candidate 22 (non-protein coding)
206152_at	116986	AGAP2	ArfGAP with GTPase domain, ankyrin repeat and PH domain 2
205143_at	1463	NCAN	neurocan
236914_at	100506790	LOC100506790	uncharacterized LOC100506790
1561247_at	101927112	LOC101927112	uncharacterized LOC101927112
233172_at	100507073	LOC100507073	uncharacterized LOC100507073
1561518_at	283914	LOC283914	uncharacterized LOC283914
1560507_at	158833	AWAT1	acyl-CoA wax alcohol acyltransferase 1
227644_at	140730	RIMS4	regulating synaptic membrane exocytosis 4
1569811_at	729307	LOC729307	uncharacterized LOC729307
1560762_at	285972	LINC00996	long intergenic non-protein coding RNA 996
1562365_at	286177	LOC286177	uncharacterized LOC286177
213602_s_at	4320	MMP11	matrix metallopeptidase 11 (stromelysin 3)
225763_at	92241	RCSD1	RCSD domain containing 1
203849_s_at	547	KIF1A	kinesin family member 1A
203929_s_at	4137	MAPT	microtubule-associated protein tau
231758_at	58531	PRM3	protamine 3
209006_s_at	57035	RSRP1	arginine/serine-rich protein 1
206384_at	10368	CACNG3	calcium channel, voltage-dependent, gamma subunit 3
205958_x_at	1444	CSHL1	chorionic somatomammotropin hormone-like 1
221363_x_at	2848	GPR25	G protein-coupled receptor 25
220845_at	55289	ACOXL	acyl-CoA oxidase-like
205790_at	8631	SKAP1	src kinase associated phosphoprotein 1
207151_at	117	ADCYAP1R1	adenylate cyclase activating polypeptide 1 (pituitary) receptor type I
1569681_at	101929378	LOC101929378	uncharacterized LOC101929378
1563897_at	1381	CRABP1	cellular retinoic acid binding protein 1
220508_at	150160	CCT8L2	chaperonin containing TCP1, subunit 8 (theta)-like 2
242284_at	199899	LINC00466	long intergenic non-protein coding RNA 466
1570080_at	101928846	HIPK1-AS1	HIPK1 antisense RNA 1
206771_at	7380	UPK3A	uroplakin 3A
237712_at	100507562	LOC100507562	uncharacterized LOC100507562
1557328_at	283665	LOC283665	uncharacterized LOC283665
1562754_at	339260	LOC339260	uncharacterized LOC339260
220623_s_at	80705	TSGA10	testis specific, 10
1555475_x_at	26140	TTLL3	tubulin tyrosine ligase-like family, member 3
234771_at	84237	DKFZp547J222	uncharacterized LOC84237
205654_at	722	C4BPA	complement component 4 binding protein, alpha
208182_x_at	3448	IFNA14	interferon, alpha 14

219270_at	79094	CHAC1	ChaC, cation transport regulator homolog 1 (E. coli)
1555310_a_at	56924	PAK6	p21 protein (Cdc42/Rac)-activated kinase 6
244709_at	205147	AMER3	APC membrane recruitment protein 3
224362_at	100128922	LOC100128922	connexin
208692_at	6188	RPS3	ribosomal protein S3
1568638_a_at	169355	IDO2	indoleamine 2,3-dioxygenase 2
222829_s_at	53832	IL20RA	interleukin 20 receptor, alpha
1561539_at	100506368	LOC100506368	uncharacterized LOC100506368
1570289_at	646736	LOC646736	uncharacterized LOC646736
1562645_at	401176	LOC401176	uncharacterized LOC401176
1560506_at	101928161	LOC101928161	uncharacterized LOC101928161
236745_at	124093	CCDC78	coiled-coil domain containing 78
217269_s_at	5651	TMPRSS15	transmembrane protease, serine 15
221469_at	2854	GPR32	G protein-coupled receptor 32
201031_s_at	3187	HNRNPH1	heterogeneous nuclear ribonucleoprotein H1 (H)
204743_at	29114	TAGLN3	transgelin 3
1559268_at	8745	ADAM23	ADAM metallopeptidase domain 23
214555_at	6755	SSTR5	somatostatin receptor 5
1553006_at	139378	GPR112	G protein-coupled receptor 112
234639_x_at	83901	KRTAP9-8	keratin associated protein 9-8
243534_at	387707	CC2D2B	coiled-coil and C2 domain containing 2B
215736_at	27012	KCNV1	potassium channel, subfamily V, member 1
1554938_a_at	26027	ACOT11	acyl-CoA thioesterase 11
1552836_at	285267	ZNF619	zinc finger protein 619
1554742_at	5378	PMS1	PMS1 postmeiotic segregation increased 1 (S. cerevisiae)
1552491_at	91734	IDI2	isopentenyl-diphosphate delta isomerase 2
239061_at	348825	TPRXL	tetra-peptide repeat homeobox-like
203256_at	1001	CDH3	cadherin 3, type 1, P-cadherin (placental)
205942_s_at	6296	ACSM3	acyl-CoA synthetase medium-chain family member 3
1561607_at	101929384	LOC101929384	uncharacterized LOC101929384
207834_at	2192	FBLN1	fibulin 1
210800_at	1678	TIMM8A	translocase of inner mitochondrial membrane 8 homolog A (yeast)
220946_s_at	29072	SETD2	SET domain containing 2
1561847_at	200035	NUDT17	nudix (nucleoside diphosphate linked moiety X)-type motif 17
229707_at	80095	ZNF606	zinc finger protein 606
224289_s_at	387316	VN1R10P	vomeronasal 1 receptor 10 pseudogene
1569332_at	677779	LINC00488	long intergenic non-protein coding RNA 488
209846_s_at	11118	BTN3A2	butyrophilin, subfamily 3, member A2
206294_at	3284	HSD3B2	hydroxy-delta-5-steroid dehydrogenase, 3 beta- and steroid delta-isomerase 2
1559837_at	101928420	LINC01490	long intergenic non-protein coding RNA 1490
218953_s_at	78991	PCYOX1L	prenylcysteine oxidase 1 like
222067_x_at	3017	HIST1H2BD	histone cluster 1, H2bd
223529_at	6860	SYT4	synaptotagmin IV
204923_at	54440	SASH3	SAM and SH3 domain containing 3
210945_at	1288	COL4A6	collagen, type IV, alpha 6
218870_at	55843	ARHGAP15	Rho GTPase activating protein 15
207374_at	57047	PLSCR2	phospholipid scramblase 2
232942_at	84795	PYROXD2	pyridine nucleotide-disulphide oxidoreductase domain 2
223927_at	56127	PCDHB9	protocadherin beta 9
218835_at	729238	SFTPA2	surfactant protein A2
233111_at	100302522	PTCSC1	papillary thyroid carcinoma susceptibility candidate 1 (non-protein coding)
232881_at	149775	GNAS-AS1	GNAS antisense RNA 1
1557606_at	101928002	LOC101928002	uncharacterized LOC101928002
216617_s_at	4099	MAG	myelin associated glycoprotein
214324_at	2813	GP2	glycoprotein 2 (zymogen granule membrane)
224423_x_at	5370	PMCHL2	pro-melanin-concentrating hormone-like 2, pseudogene
230826_at	221938	MMD2	monocyte to macrophage differentiation-associated 2
1552964_at	54777	CFAP46	cilia and flagella associated protein 46
1564377_at	22941	SHANK2	SH3 and multiple ankyrin repeat domains 2
1557731_at	400620	LOC400620	uncharacterized LOC400620
1552819_at	257103	LINC00205	long intergenic non-protein coding RNA 205

234524_at	63977	PRDM15	PR domain containing 15
242782_x_at	130612	TMEM198	transmembrane protein 198
1557680_at	161394	SAMD15	sterile alpha motif domain containing 15
242271_at	115019	SLC26A9	solute carrier family 26 (anion exchanger), member 9
207314_x_at	3812	KIR3DL2	killer cell immunoglobulin-like receptor, three domains, long cytoplasmic tail, 2
1553153_at	245972	ATP6V0D2	ATPase, H+ transporting, lysosomal 38kDa, V0 subunit d2
1560958_s_at	101928516	LOC101928516	uncharacterized LOC101928516
1563174_at	101929620	LINC01243	long intergenic non-protein coding RNA 1243
219591_at	51286	CEND1	cell cycle exit and neuronal differentiation 1
207017_at	5874	RAB27B	RAB27B, member RAS oncogene family
1562510_at	339442	LINC01343	long intergenic non-protein coding RNA 1343
208299_at	8911	CACNA1I	calcium channel, voltage-dependent, T type, alpha 1I subunit
1561442_at	283585	LOC283585	uncharacterized LOC283585
1562326_at	400955	LINC01122	long intergenic non-protein coding RNA 1122
1562336_at	255082	CASC2	cancer susceptibility candidate 2 (non-protein coding)
1563771_a_at	101929073	LOC101929073	uncharacterized LOC101929073
230890_at	57536	KIAA1328	KIAA1328
1558007_s_at	100506655	LCMT1-AS2	LCMT1 antisense RNA 2
220194_at	79730	NSUN7	NOP2/Sun domain family, member 7
1562527_at	441666	LOC441666	zinc finger protein 91 pseudogene
1556941_a_at	283484	LOC283484	uncharacterized LOC283484
224529_s_at	84618	NT5C1A	5'-nucleotidase, cytosolic IA
232605_s_at	100507135	SPG20OS	SPG20 opposite strand
215861_at	101927770	LOC101927770	uncharacterized LOC101927770
230616_at	22973	LAMB2P1	laminin, beta 2 pseudogene 1
1554233_at	338872	C1QTNF9	C1q and tumor necrosis factor related protein 9
231253_at	162540	SPPL2C	signal peptide peptidase like 2C
224925_at	57580	PREX1	phosphatidylinositol-3,4,5-trisphosphate-dependent Rac exchange factor 1
1553991_s_at	54621	VSIG10	V-set and immunoglobulin domain containing 10
223508_at	4851	NOTCH1	notch 1
204002_s_at	3382	ICA1	islet cell autoantigen 1, 69kDa
206843_at	1413	CRYBA4	crystallin, beta A4
221332_at	9210	BMP15	bone morphogenetic protein 15
1563821_at	170425	LINC00858	long intergenic non-protein coding RNA 858
1552391_at	164127	CCDC185	coiled-coil domain containing 185
226218_at	3575	IL7R	interleukin 7 receptor
203509_at	6653	SORL1	sortilin-related receptor, L(DLR class) A repeats containing
1570156_s_at	342184	FMN1	formin 1
1562424_at	285889	LOC285889	uncharacterized LOC285889
1552747_a_at	151649	PP2D1	protein phosphatase 2C-like domain containing 1
220962_s_at	29943	PADI1	peptidyl arginine deiminase, type I
220465_at	80054	CEBPA-AS1	CEBPA antisense RNA 1 (head to head)
200943_at	3150	HMGN1	high mobility group nucleosome binding domain 1
221072_at	57000	GSN-AS1	GSN antisense RNA 1
235101_at	23360	FNBP4	formin binding protein 4
221164_x_at	23563	CHST5	carbohydrate (N-acetylglucosamine 6-O) sulfotransferase 5
1559138_a_at	219938	SPATA19	spermatogenesis associated 19
224540_at	90070	LACRT	lacritin
203455_s_at	6303	SAT1	spermidine/spermine N1-acetyltransferase 1
1553033_at	94122	SYTL5	synaptotagmin-like 5
207349_s_at	7352	UCP3	uncoupling protein 3 (mitochondrial, proton carrier)
1561414_at	401497	LINC01242	long intergenic non-protein coding RNA 1242
207936_x_at	10738	RFPL3	ret finger protein-like 3
201891_s_at	567	B2M	beta-2-microglobulin
215637_at	95681	CEP41	centrosomal protein 41kDa
208172_s_at	9312	KCNB2	potassium voltage-gated channel, Shab-related subfamily, member 2
231389_at	139212	PIH1D3	PIH1 domain containing 3
204328_at	11322	TMC6	transmembrane channel-like 6
207526_s_at	9173	IL1RL1	interleukin 1 receptor-like 1
1558565_at	80709	AKNA	AT-hook transcription factor
238004_at	267002	PGBD2	piggyBac transposable element derived 2

205363_at	8424	BBOX1	butyrobetaine (gamma), 2-oxoglutarate dioxygenase (gamma-butyrobetaine hydroxylase) 1
220940_at	57730	ANKRD36B	ankyrin repeat domain 36B
213953_at	54474	KRT20	keratin 20
1563872_at	284395	LOC284395	uncharacterized LOC284395
1555332_at	121278	TPH2	tryptophan hydroxylase 2
234946_at	955	ENTPD6	ectonucleoside triphosphate diphosphohydrolase 6 (putative)
229023_at	84826	SFT2D3	SFT2 domain containing 3
1569782_at	101929116	LOC101929116	uncharacterized LOC101929116
1570445_a_at	643201	LOC643201	centrosomal protein 192kDa pseudogene
207367_at	479	ATP12A	ATPase, H+/K+ transporting, nongastric, alpha polypeptide
1564203_at	147004	LOC147004	uncharacterized LOC147004
217168_s_at	9709	HERPUD1	homocysteine-inducible, endoplasmic reticulum stress-inducible, ubiquitin-like domain member 1
1559003_a_at	126661	CCDC163P	coiled-coil domain containing 163, pseudogene
215925_s_at	971	CD72	CD72 molecule
222005_s_at	2785	GNG3	guanine nucleotide binding protein (G protein), gamma 3
205454_at	3208	HPCA	hippocalcin
1560207_at	644660	RAD21-AS1	RAD21 antisense RNA 1
221373_x_at	5623	PSPN	persephin
1562821_a_at	100506492	DSCAM-AS1	DSCAM antisense RNA 1
1556832_at	101927314	LOC101927314	uncharacterized LOC101927314
220604_x_at	10841	FTCD	formimidoyltransferase cyclodeaminase
234903_at	442184	OR2B3	olfactory receptor, family 2, subfamily B, member 3
1563639_a_at	114827	FHAD1	forkhead-associated (FHA) phosphopeptide binding domain 1
233238_s_at	101927115	CTB-12O2.1	uncharacterized LOC101927115
1568986_x_at	51604	PIGT	phosphatidylinositol glycan anchor biosynthesis, class T
221684_s_at	60506	NYX	nyctalopin
1555405_at	9690	UBE3C	ubiquitin protein ligase E3C
236833_at	158248	TTC16	tetratricopeptide repeat domain 16
214478_at	6694	SPP2	secreted phosphoprotein 2, 24kDa
1559479_at	285540	SEPSECS-AS1	SEPSECS antisense RNA 1 (head to head)
1560785_at	8444	DYRK3	dual-specificity tyrosine-(Y)-phosphorylation regulated kinase 3
1555942_a_at	642587	MIR205HG	MIR205 host gene (non-protein coding)
224198_at	1990	CELA1	chymotrypsin-like elastase family, member 1
238171_at	253512	SLC25A30	solute carrier family 25, member 30
223878_at	8821	INPP4B	inositol polyphosphate-4-phosphatase, type II, 105kDa
1562621_at	101927040	LOC101927040	uncharacterized LOC101927040
222234_s_at	79007	DBNDD1	dysbindin (dystrobrevin binding protein 1) domain containing 1
236033_at	142689	ASB12	ankyrin repeat and SOCS box containing 12
1562724_at	286114	LOC286114	uncharacterized LOC286114
201709_s_at	8508	NIPSNAP1	nipsnap homolog 1 (C. elegans)
238373_at	341567	H1FNT	H1 histone family, member N, testis-specific
236534_at	149428	BNIPL	BCL2/adenovirus E1B 19kD interacting protein like
220059_at	26228	STAP1	signal transducing adaptor family member 1
235988_at	266977	GPR110	G protein-coupled receptor 110
207583_at	225	ABCD2	ATP-binding cassette, sub-family D (ALD), member 2
1563993_at	101929268	LOC101929268	uncharacterized LOC101929268
219551_at	55840	EAF2	ELL associated factor 2
231825_x_at	55729	ATF7IP	activating transcription factor 7 interacting protein
244372_at	101060019	LOC101060019	uncharacterized LOC101060019
215430_at	2712	GK2	glycerol kinase 2
228892_at	153769	SH3RF2	SH3 domain containing ring finger 2
240876_x_at	145645	C15orf43	chromosome 15 open reading frame 43
1553204_at	253868	C20orf166-AS1	C20orf166 antisense RNA 1
1570328_s_at	140834	C20orf62	chromosome 20 open reading frame 62
1562901_at	400456	LINC01197	long intergenic non-protein coding RNA 1197
237699_at	100507040	LINC00427	long intergenic non-protein coding RNA 427
218726_at	55355	HJURP	Holliday junction recognition protein
1562433_at	379034	LINC01181	long intergenic non-protein coding RNA 1181
208013_s_at	56	ACRV1	acrosomal vesicle protein 1
232821_at	149699	GTSF1L	gametocyte specific factor 1-like

218237_s_at	81539	SLC38A1	solute carrier family 38, member 1
1568898_at	101928140	LOC101928140	uncharacterized LOC101928140
1565554_at	127841	LINC00628	long intergenic non-protein coding RNA 628
237163_x_at	390705	LOC390705	protein phosphatase 2, regulatory subunit B'', beta pseudogene
216779_at	1538	CYLC1	cylicin, basic protein of sperm head cytoskeleton 1
229599_at	440335	SMIM22	small integral membrane protein 22
206164_at	9635	CLCA2	chloride channel accessory 2
230419_at	400618	SOX9-AS1	SOX9 antisense RNA 1
234757_at	100129884	LOC100129884	uncharacterized LOC100129884
220816_at	23566	LPAR3	lysophosphatidic acid receptor 3
1562982_at	101928795	LINC01448	long intergenic non-protein coding RNA 1448
233162_at	101926892	LOC101926892	uncharacterized LOC101926892
1559252_a_at	101929125	LOC101929125	uncharacterized LOC101929125
220923_s_at	29944	PNMA3	paraneoplastic Ma antigen 3
236400_at	100507475	IDH1-AS1	IDH1 antisense RNA 1
230506_at	63914	C6orf164	chromosome 6 open reading frame 164
223642_at	7546	ZIC2	Zic family member 2
216601_at	90586	AOC4P	amine oxidase, copper containing 4, pseudogene
207166_at	2792	GNGT1	guanine nucleotide binding protein (G protein), gamma transducing activity polypeptide 1
1558794_at	728190	NUTM2A-AS1	NUTM2A antisense RNA 1
206134_at	27299	ADAMDEC1	ADAM-like, decysin 1
1553341_at	131669	UROC1	urocanate hydratase 1
208203_x_at	3810	KIR2DS5	killer cell immunoglobulin-like receptor, two domains, short cytoplasmic tail, 5
228509_at	80309	SPHKAP	SPHK1 interactor, AKAP domain containing
1562549_at	101927586	LINC01271	long intergenic non-protein coding RNA 1271
208384_s_at	11043	MID2	midline 2
211331_x_at	3077	HFE	hemochromatosis
1555051_at	282966	C10orf53	chromosome 10 open reading frame 53
232211_at	89801	PPP1R3F	protein phosphatase 1, regulatory subunit 3F
214468_at	4624	MYH6	myosin, heavy chain 6, cardiac muscle, alpha
1570224_at	101928773	LINC01449	long intergenic non-protein coding RNA 1449
1553809_a_at	169693	TMEM252	transmembrane protein 252
217030_at	101929550	LOC101929550	uncharacterized LOC101929550
1561322_at	101927450	LOC101927450	uncharacterized LOC101927450
238655_at	80724	ACAD10	acyl-CoA dehydrogenase family, member 10
224388_s_at	84570	COL25A1	collagen, type XXV, alpha 1
231554_at	89882	TPD52L3	tumor protein D52-like 3
220660_at	58483	LINC00474	long intergenic non-protein coding RNA 474
1561294_a_at	100128554	LOC100128554	uncharacterized LOC100128554
1562121_at	101927193	CHL1-AS1	CHL1 antisense RNA 1
1564785_at	196913	C14orf183	chromosome 14 open reading frame 183
214825_at	728215	FAM155A	family with sequence similarity 155, member A
242350_s_at	100128098	ST8SIA6-AS1	ST8SIA6 antisense RNA 1
227952_at	152687	ZNF595	zinc finger protein 595
232814_x_at	84334	APOPT1	apoptogenic 1, mitochondrial
1552620_at	163778	SPRR4	small proline-rich protein 4
223581_at	84765	ZNF577	zinc finger protein 577
1561262_at	101929025	LINC01516	long intergenic non-protein coding RNA 1516
224293_at	246119	TTTY10	testis-specific transcript, Y-linked 10 (non-protein coding)
235049_at	107	ADCY1	adenylate cyclase 1 (brain)
1568871_at	100874038	CADM2-AS1	CADM2 antisense RNA 1
237980_at	338864	LINC00347	long intergenic non-protein coding RNA 347
1563655_at	7139	TNNT2	troponin T type 2 (cardiac)
205569_at	27074	LAMP3	lysosomal-associated membrane protein 3
1558402_at	101928857	CTD-3080P12.3	uncharacterized LOC101928857
1557047_at	55689	YEATS2	YEATS domain containing 2
236563_at	343035	RD3	retinal degeneration 3
1563035_x_at	2819	GPD1	glycerol-3-phosphate dehydrogenase 1 (soluble)
217574_at	1006	CDH8	cadherin 8, type 2
241538_at	645090	CXorf30	chromosome X open reading frame 30

229275_at	91156	IGFN1	immunoglobulin-like and fibronectin type III domain containing 1
1553895_at	160298	C11orf42	chromosome 11 open reading frame 42
235900_at	201305	SPNS3	spinster homolog 3 (Drosophila)
210109_at	27099	SND1-IT1	SND1 intronic transcript 1 (non-protein coding)
227563_at	100131997	FAM27E3	family with sequence similarity 27, member E3
1562447_a_at	100506599	PPP1R26-AS1	PPP1R26 antisense RNA 1
217158_at	442421	PTGER4P2-CDK2AP2P2	PTGER4P2-CDK2AP2P2 readthrough transcribed pseudogene
229152_at	260436	FDCSP	follicular dendritic cell secreted protein
1558844_at	100506127	LOC100506127	putative uncharacterized protein FLJ37770-like
231126_at	339778	C2orf70	chromosome 2 open reading frame 70
1555533_at	84109	QRFPR	pyroglutamylated RFamide peptide receptor
219832_s_at	3229	HOXC13	homeobox C13
1556826_s_at	374946	DRAXIN	dorsal inhibitory axon guidance protein
233530_at	100507495	SDCBP2-AS1	SDCBP2 antisense RNA 1
1557486_at	101927547	ANKRD44-IT1	ANKRD44 intronic transcript 1 (non-protein coding)
1553879_a_at	137362	GOT1L1	glutamic-oxaloacetic transaminase 1-like 1
226264_at	64420	SUSD1	sushi domain containing 1
211520_s_at	2890	GRIA1	glutamate receptor, ionotropic, AMPA 1
1562378_s_at	150696	PROM2	prominin 2
1557098_s_at	768096	HAR1A	highly accelerated region 1A (non-protein coding)
206630_at	7299	TYR	tyrosinase
1562272_at	101927914	LOC101927914	uncharacterized LOC101927914
1552993_at	143241	DYDC1	DPY30 domain containing 1
232697_at	57497	LRFN2	leucine rich repeat and fibronectin type III domain containing 2
1562264_at	339685	LOC339685	uncharacterized LOC339685
1561091_at	101928684	LINC01209	long intergenic non-protein coding RNA 1209
227676_at	131177	FAM3D	family with sequence similarity 3, member D
1554195_a_at	389336	C5orf46	chromosome 5 open reading frame 46
231366_at	619190	FDPSP2	farnesyl diphosphate synthase pseudogene 2
1559544_s_at	100862704	LINC00441	long intergenic non-protein coding RNA 441
210912_x_at	2948	GSTM4	glutathione S-transferase mu 4
1553077_at	121214	SDR9C7	short chain dehydrogenase/reductase family 9C, member 7
237701_at	121273	C12orf54	chromosome 12 open reading frame 54
1561853_a_at	149233	IL23R	interleukin 23 receptor
220445_s_at	389903	CSAG3	CSAG family, member 3
230057_at	285178	LOC285178	uncharacterized LOC285178
207827_x_at	6622	SNCA	synuclein, alpha (non A4 component of amyloid precursor)
1569268_at	2905	GRIN2C	glutamate receptor, ionotropic, N-methyl D-aspartate 2C
1554810_at	8605	PLA2G4C	phospholipase A2, group IVC (cytosolic, calcium-independent)
203673_at	7038	TG	thyroglobulin
1557702_at	101927604	LOC101927604	uncharacterized LOC101927604
209083_at	11151	CORO1A	coronin, actin binding protein, 1A
215588_x_at	8780	RIOK3	RIO kinase 3
1567284_at	26219	OR1J4	olfactory receptor, family 1, subfamily J, member 4
242226_at	100288079	GS1-279B7.1	microtubule-associated protein 1 light chain 3 beta pseudogene
241475_at	286076	BREA2	breast cancer estrogen-induced apoptosis 2
1556529_a_at	285326	LINC00692	long intergenic non-protein coding RNA 692
1563744_a_at	27185	DISC1	disrupted in schizophrenia 1
230881_at	146849	CCDC42	coiled-coil domain containing 42
1555231_a_at	114041	C21orf88	chromosome 21 open reading frame 88
207324_s_at	1823	DSC1	desmocollin 1
1557783_at	100133991	MAP3K14-AS1	MAP3K14 antisense RNA 1
1561252_at	648691	LOC648691	uncharacterized LOC648691
217273_at	343071	PRAMEF10	PRAME family member 10
1559263_s_at	340152	ZC3H12D	zinc finger CCCH-type containing 12D
240830_at	677769	SCARNA17	small Cajal body-specific RNA 17
1553600_at	259236	TMIE	transmembrane inner ear
1569353_at	9738	CCP110	centriolar coiled coil protein 110kDa
204092_s_at	6790	AURKA	aurora kinase A
1563063_at	101927224	LINC01049	long intergenic non-protein coding RNA 1049

220791_x_at	11280	SCN11A	sodium channel, voltage-gated, type XI, alpha subunit
1561216_at	101928961	LOC101928961	uncharacterized LOC101928961
238188_at	574432	IBA57-AS1	IBA57 antisense RNA 1 (head to head)
213636_at	23349	KIAA1045	KIAA1045
1559621_at	101927237	LOC101927237	uncharacterized LOC101927237
1570270_at	101926942	LOC101926942	uncharacterized LOC101926942
221396_at	50837	TAS2R7	taste receptor, type 2, member 7
215289_at	388567	ZNF749	zinc finger protein 749
234250_at	100506405	LOC100506405	uncharacterized LOC100506405
1561677_at	140733	MACROD2	MACRO domain containing 2
1559050_at	253018	HCG27	HLA complex group 27 (non-protein coding)
216493_s_at	10643	IGF2BP3	insulin-like growth factor 2 mRNA binding protein 3
1555040_at	253128	LINC00612	long intergenic non-protein coding RNA 612
241587_at	100862679	NAALADL2-AS3	NAALADL2 antisense RNA 3
223688_s_at	54742	LY6K	lymphocyte antigen 6 complex, locus K
207054_at	3617	IMPG1	interphotoreceptor matrix proteoglycan 1
233962_at	140846	FAM83C-AS1	FAM83C antisense RNA 1
230287_at	129049	SGSM1	small G protein signaling modulator 1
1560879_a_at	83849	SYT15	synaptotagmin XV
211274_at	6899	TBX1	T-box 1
1556171_a_at	101927516	LOC101927516	uncharacterized LOC101927516
209374_s_at	3507	IGHM	immunoglobulin heavy constant mu
1558782_a_at	100130557	NFYC-AS1	NFYC antisense RNA 1
227781_x_at	83723	FAM57B	family with sequence similarity 57, member B
1556820_a_at	8847	DLEU2	deleted in lymphocytic leukemia 2 (non-protein coding)
1559002_at	340544	MORF4L2-AS1	MORF4L2 antisense RNA 1
1553804_a_at	124783	SPATA32	spermatogenesis associated 32
235001_at	134218	DNAJC21	DnaJ (Hsp40) homolog, subfamily C, member 21
1560954_at	101928167	LOC101928167	uncharacterized LOC101928167
1563529_at	100288805	HYDIN2	HYDIN2, axonemal central pair apparatus protein (pseudogene)
211001_at	23650	TRIM29	tripartite motif containing 29
210459_at	5710	PSMD4	proteasome (prosome, macropain) 26S subunit, non-ATPase, 4
210650_s_at	27445	PCLO	piccolo presynaptic cytomatrix protein
243313_at	79933	SYNPO2L	synaptopodin 2-like
234466_at	56975	FAM20C	family with sequence similarity 20, member C
207049_at	6334	SCN8A	sodium channel, voltage gated, type VIII, alpha subunit
1563743_at	439927	C1orf180	chromosome 1 open reading frame 180
1570023_at	641515	MGAT4EP	MGAT4 family, member E, pseudogene
210339_s_at	3817	KLK2	kallikrein-related peptidase 2
1561213_at	101927295	LINC01360	long intergenic non-protein coding RNA 1360
220781_at	50514	DEC1	deleted in esophageal cancer 1
1561245_at	102724612	LOC102724612	uncharacterized LOC102724612
234050_at	117289	TAGAP	T-cell activation RhoGTPase activating protein
1568804_at	9451	EIF2AK3	eukaryotic translation initiation factor 2-alpha kinase 3
1564122_at	283875	LINC00514	long intergenic non-protein coding RNA 514
206680_at	922	CD5L	CD5 molecule-like
208552_at	2900	GRIK4	glutamate receptor, ionotropic, kainate 4
234679_at	83900	KRTAP9-3	keratin associated protein 9-3
237340_at	116369	SLC26A8	solute carrier family 26 (anion exchanger), member 8
240760_at	146822	CDRT15	CMT1A duplicated region transcript 15
1552713_a_at	6521	SLC4A1	solute carrier family 4 (anion exchanger), member 1 (Diego blood group)
1569544_at	101927152	LINC01212	long intergenic non-protein coding RNA 1212
1555345_at	55089	SLC38A4	solute carrier family 38, member 4
1560457_x_at	440503	PLIN5	perilipin 5
1553057_at	89777	SERPINB12	serpin peptidase inhibitor, clade B (ovalbumin), member 12
212960_at	23158	TBC1D9	TBC1 domain family, member 9 (with GRAM domain)
1553378_a_at	256957	HEATR9	HEAT repeat containing 9
1555708_a_at	266553	OFCC1	orofacial cleft 1 candidate 1
211817_s_at	3762	KCNJ5	potassium inwardly-rectifying channel, subfamily J, member 5
214707_x_at	7840	ALMS1	Alstrom syndrome 1
237613_at	283150	FOXR1	forkhead box R1
1552889_a_at	90332	EXOC3L2	exocyst complex component 3-like 2

234617_at	390066	OR52D1	olfactory receptor, family 52, subfamily D, member 1
220071_x_at	55142	HAUS2	HAUS augmin-like complex, subunit 2
243708_at	124842	TMEM132E	transmembrane protein 132E
235997_at	7625	ZNF74	zinc finger protein 74
211040_x_at	51512	GTSE1	G-2 and S-phase expressed 1
1554711_at	119395	CALHM3	calcium homeostasis modulator 3
1555034_at	7401	CLRN1	clarin 1
205662_at	27077	B9D1	B9 protein domain 1
234117_at	404744	NPSR1-AS1	NPSR1 antisense RNA 1
244056_at	389376	SFTA2	surfactant associated 2
219630_at	10158	PDZK1IP1	PDZK1 interacting protein 1
241066_at	203523	ZNF449	zinc finger protein 449
220179_at	64180	DPEP3	dipeptidase 3
1566665_at	643677	CCDC168	coiled-coil domain containing 168
232609_at	92359	CRB3	crumbs family member 3
237621_at	101928869	LOC101928869	uncharacterized LOC101928869
1554559_at	118442	GPR62	G protein-coupled receptor 62
208183_at	6870	TACR3	tachykinin receptor 3
242332_at	400550	FENDRR	FOXF1 adjacent non-coding developmental regulatory RNA
1561101_at	153469	JAKMIP2-AS1	JAKMIP2 antisense RNA 1
228849_at	4916	NTRK3	neurotrophic tyrosine kinase, receptor, type 3
229888_at	144608	C12orf60	chromosome 12 open reading frame 60
208521_at	10798	OR5I1	olfactory receptor, family 5, subfamily I, member 1
1555171_at	6487	ST3GAL3	ST3 beta-galactoside alpha-2,3-sialyltransferase 3
220811_at	10394	PRG3	proteoglycan 3
221558_s_at	51176	LEF1	lymphoid enhancer-binding factor 1
243195_s_at	90233	ZNF551	zinc finger protein 551
223325_at	51061	TXNDC11	thioredoxin domain containing 11
240809_at	150142	ZNF295-AS1	ZNF295 antisense RNA 1
1552944_a_at	56666	PANX2	pannexin 2
220369_at	55671	SMEK1	SMEK homolog 1, suppressor of mek1 (Dictyostelium)
1563119_at	100652929	HP09025	uncharacterized LOC100652929
229041_s_at	100505746	ITGB2-AS1	ITGB2 antisense RNA 1
1557875_at	101928165	CASC17	cancer susceptibility candidate 17 (non-protein coding)
229522_at	93517	SDR42E1	short chain dehydrogenase/reductase family 42E, member 1
232785_at	57529	RGAG1	retrotransposon gag domain containing 1
1559443_s_at	283888	IL21R-AS1	IL21R antisense RNA 1
244071_at	345630	FBLL1	fibrillarin-like 1
231124_x_at	4063	LY9	lymphocyte antigen 9
206208_at	762	CA4	carbonic anhydrase IV
229332_at	84842	HPDL	4-hydroxyphenylpyruvate dioxygenase-like
220710_at	80035	ANP32A-IT1	ANP32A intronic transcript 1 (non-protein coding)
240091_at	143471	PSMA8	proteasome (prosome, macropain) subunit, alpha type, 8
206897_at	8712	PAGE1	P antigen family, member 1 (prostate associated)
216328_at	27181	SIGLEC8	sialic acid binding Ig-like lectin 8
1559627_at	285941	C7orf71	chromosome 7 open reading frame 71
200803_s_at	7009	TMBIM6	transmembrane BAX inhibitor motif containing 6
207908_at	3849	KRT2	keratin 2
232250_at	57501	KIAA1257	KIAA1257
226062_x_at	55793	FAM63A	family with sequence similarity 63, member A
240786_at	4855	NOTCH4	notch 4
235604_x_at	284443	ZNF493	zinc finger protein 493
1567251_at	26496	OR10A3	olfactory receptor, family 10, subfamily A, member 3
207337_at	30848	CTAG2	cancer/testis antigen 2
238177_at	340024	SLC6A19	solute carrier family 6 (neutral amino acid transporter), member 19
1566219_at	338651	KRTAP5-AS1	KRTAP5-1/KRTAP5-2 antisense RNA 1
221111_at	55801	IL26	interleukin 26
206762_at	3741	KCNA5	potassium voltage-gated channel, shaker-related subfamily, member 5
201288_at	397	ARHGDIB	Rho GDP dissociation inhibitor (GDI) beta
204921_at	2622	GAS8	growth arrest-specific 8
229346_at	10763	NES	nestin
232554_at	115399	LRRC56	leucine rich repeat containing 56

207930_at	3933	LCN1	lipocalin 1
231485_at	100505540	SLIT1-AS1	SLIT1 antisense RNA 1
1570013_at	101927411	LOC101927411	uncharacterized LOC101927411
1557862_at	654841	LOC654841	uncharacterized LOC654841
229276_at	57549	IGSF9	immunoglobulin superfamily, member 9
1568719_s_at	63027	SLC22A23	solute carrier family 22, member 23
1562623_at	146513	LOC146513	uncharacterized LOC146513
214539_at	5273	SERPINB10	serpin peptidase inhibitor, clade B (ovalbumin), member 10
206206_at	4064	CD180	CD180 molecule
233679_at	100506472	LOC100506472	uncharacterized LOC100506472
234048_s_at	57724	EPG5	ectopic P-granules autophagy protein 5 homolog (C. elegans)
1560934_at	284669	LOC284669	uncharacterized LOC284669
236838_at	80725	SRCIN1	SRC kinase signaling inhibitor 1
211748_x_at	5730	PTGDS	prostaglandin D2 synthase 21kDa (brain)
1557570_a_at	285084	LINC01305	long intergenic non-protein coding RNA 1305
243468_at	160065	PATE1	prostate and testis expressed 1
208530_s_at	5915	RARB	retinoic acid receptor, beta
220722_s_at	60482	SLC5A7	solute carrier family 5 (sodium/choline cotransporter), member 7
235794_at	4336	MOBP	myelin-associated oligodendrocyte basic protein
219683_at	7976	FZD3	frizzled class receptor 3
231422_x_at	219743	TYSND1	trypsin domain containing 1
1553422_s_at	54715	RBFOX1	RNA binding protein, fox-1 homolog (C. elegans) 1
210237_at	9048	ARTN	artemin
216659_at	1720	LOC1720	dihydrofolate reductase pseudogene
207182_at	2559	GABRA6	gamma-aminobutyric acid (GABA) A receptor, alpha 6
1559616_x_at	199777	ZNF626	zinc finger protein 626
215434_x_at	55672	NBPF1	neuroblastoma breakpoint family, member 1
1563013_at	646522	LOC646522	uncharacterized LOC646522
1557026_at	101927787	LOC101927787	uncharacterized LOC101927787
229124_at	84432	PROK1	prokineticin 1
207208_at	27288	RBMXL2	RNA binding motif protein, X-linked-like 2
222729_at	55294	FBXW7	F-box and WD repeat domain containing 7, E3 ubiquitin protein ligase
220782_x_at	43849	KLK12	kallikrein-related peptidase 12
224294_at	100128185	LOC100128185	PNAS-19
220309_at	55001	TTC22	tetratricopeptide repeat domain 22
1555687_a_at	170482	CLEC4C	C-type lectin domain family 4, member C
239290_at	9758	FRMPD4	FERM and PDZ domain containing 4
210015_s_at	4133	MAP2	microtubule-associated protein 2
233100_at	80820	EEPD1	endonuclease/exonuclease/phosphatase family domain containing 1
1563373_at	731223	LINC01220	long intergenic non-protein coding RNA 1220
1554675_a_at	93426	SYCE1	synaptonemal complex central element protein 1
216755_at	114884	OSBPL10	oxysterol binding protein-like 10
239748_x_at	54940	OCIAD1	OCIA domain containing 1
223935_at	29850	TRPM5	transient receptor potential cation channel, subfamily M, member 5
221093_at	23629	BRD7P3	bromodomain containing 7 pseudogene 3
1566865_at	221786	FAM200A	family with sequence similarity 200, member A
221658_s_at	50615	IL21R	interleukin 21 receptor
202488_s_at	5349	FXYD3	FXYD domain containing ion transport regulator 3
205237_at	2219	FCN1	ficolin (collagen/fibrinogen domain containing) 1
1557761_s_at	400794	LOC400794	uncharacterized LOC400794
1567249_at	26495	OR9A1P	olfactory receptor, family 9, subfamily A, member 1 pseudogene
215532_x_at	57615	ZNF492	zinc finger protein 492
243231_at	151258	SLC38A11	solute carrier family 38, member 11
1564352_at	27442	CECR3	cat eye syndrome chromosome region, candidate 3 (non-protein coding)
204734_at	3866	KRT15	keratin 15
227074_at	100131564	LOC100131564	uncharacterized LOC100131564
229481_at	85407	NKD1	naked cuticle homolog 1 (Drosophila)
207221_at	9002	F2RL3	coagulation factor II (thrombin) receptor-like 3
204399_s_at	24139	EML2	echinoderm microtubule associated protein like 2
237033_at	348378	FAM159A	family with sequence similarity 159, member A
234511_at	140731	ANKRD60	ankyrin repeat domain 60
224017_at	50945	TBX22	T-box 22

1557296_at	440101	FLJ12825	uncharacterized LOC440101
207911_s_at	9333	TGM5	transglutaminase 5
237760_at	100505768	LINC01364	long intergenic non-protein coding RNA 1364
230833_at	84519	ACRBP	acrosin binding protein
239975_at	3116	HLA-DPB2	major histocompatibility complex, class II, DP beta 2 (pseudogene)
1564697_a_at	400752	LINC01144	long intergenic non-protein coding RNA 1144
234349_at	23145	SSPO	SCO-spondin
1556883_a_at	440896	LOC440896	uncharacterized LOC440896
1570186_at	692159	GRASPOS	GRP1-associated scaffold protein opposite strand
210260_s_at	25816	TNFAIP8	tumor necrosis factor, alpha-induced protein 8
223620_at	2857	GPR34	G protein-coupled receptor 34
223550_s_at	56934	CA10	carbonic anhydrase X
219121_s_at	54845	ESRP1	epithelial splicing regulatory protein 1
1559144_x_at	100130581	LINC00910	long intergenic non-protein coding RNA 910
201401_s_at	156	ADRBK1	adrenergic, beta, receptor kinase 1
220388_at	80307	FER1L4	fer-1-like family member 4, pseudogene (functional)
220868_s_at	56301	SLC7A10	solute carrier family 7 (neutral amino acid transporter light chain, asc system), member 10
1557038_s_at	100289373	UBAC2-AS1	UBAC2 antisense RNA 1
209560_s_at	8788	DLK1	delta-like 1 homolog (Drosophila)
234054_at	143188	LOC143188	uncharacterized LOC143188
207256_at	4153	MBL2	mannose-binding lectin (protein C) 2, soluble
1567361_at	497258	BDNF-AS	BDNF antisense RNA
207538_at	3565	IL4	interleukin 4
216786_at	401630	FAM224A	family with sequence similarity 224, member A (non-protein coding)
1564485_at	100131551	LINC00887	long intergenic non-protein coding RNA 887
1560276_at	283403	C12orf80	chromosome 12 open reading frame 80
232034_at	203274	LINC00537	long intergenic non-protein coding RNA 537
220195_at	55777	MBD5	methyl-CpG binding domain protein 5
1558858_at	100506885	LOC100506885	uncharacterized LOC100506885
221398_at	50836	TAS2R8	taste receptor, type 2, member 8
207800_at	9495	AKAP5	A kinase (PRKA) anchor protein 5
224667_x_at	119504	ANAPC16	anaphase promoting complex subunit 16
1553132_a_at	123036	TC2N	tandem C2 domains, nuclear
242103_at	144110	TMEM86A	transmembrane protein 86A
209913_x_at	9907	AP5Z1	adaptor-related protein complex 5, zeta 1 subunit
1554934_at	55213	RCBTB1	regulator of chromosome condensation (RCC1) and BTB (POZ) domain containing protein 1
203528_at	10507	SEMA4D	sema domain, immunoglobulin domain (Ig), transmembrane domain (TM) and short cytoplasmic domain, (semaphorin) 4D
1565685_at	400940	LOC400940	uncharacterized LOC400940
206386_at	6906	SERPINA7	serpin peptidase inhibitor, clade A (alpha-1 antiproteinase, antitrypsin), member 7
210826_x_at	5884	RAD17	RAD17 homolog (S. pombe)
1555124_at	100129726	LINC01126	long intergenic non-protein coding RNA 1126
1566647_s_at	149086	LINC01225	long intergenic non-protein coding RNA 1225
242334_at	147945	NLRP4	NLR family, pyrin domain containing 4
208257_x_at	5669	PSG1	pregnancy specific beta-1-glycoprotein 1
205708_s_at	7226	TRPM2	transient receptor potential cation channel, subfamily M, member 2
1559406_at	253650	ANKRD18A	ankyrin repeat domain 18A
238795_at	54906	FAM208B	family with sequence similarity 208, member B
1561890_at	170371	C10orf128	chromosome 10 open reading frame 128
1555235_s_at	401067	IQCF3	IQ motif containing F3
33304_at	3669	ISG20	interferon stimulated exonuclease gene 20kDa
1554601_at	154215	NKAIN2	Na+/K+ transporting ATPase interacting 2
237475_x_at	100129792	CCDC152	coiled-coil domain containing 152
227318_at	100507421	TMEM178B	transmembrane protein 178B
208302_at	57824	HMHB1	histocompatibility (minor) HB-1
217319_x_at	284541	CYP4A22	cytochrome P450, family 4, subfamily A, polypeptide 22
216220_s_at	134	ADORA1	adenosine A1 receptor
218662_s_at	64151	NCAPG	non-SMC condensin I complex, subunit G

1552834_at	192134	B3GNT6	UDP-GlcNAc:betaGal beta-1,3-N-acetylglucosaminyltransferase 6 (core 3 synthase)
209795_at	969	CD69	CD69 molecule
243529_at	92935	MARS2	methionyl-tRNA synthetase 2, mitochondrial
206778_at	1415	CRYBB2	crystallin, beta B2
1554117_at	160777	CCDC60	coiled-coil domain containing 60
208346_at	10895	PPBPP2	pro-platelet basic protein pseudogene 2
231229_at	373861	HILS1	histone linker H1 domain, spermatid-specific 1, pseudogene
234831_at	101929164	LOC101929164	uncharacterized LOC101929164
223605_at	83733	SLC25A18	solute carrier family 25 (glutamate carrier), member 18
1562973_at	101928201	LOC101928201	uncharacterized LOC101928201
1569852_at	286006	LSMEM1	leucine-rich single-pass membrane protein 1
1554781_at	153163	MGC32805	uncharacterized LOC153163
239139_at	151835	CPNE9	copine family member IX
220082_at	54866	PPP1R14D	protein phosphatase 1, regulatory (inhibitor) subunit 14D
206804_at	917	CD3G	CD3g molecule, gamma (CD3-TCR complex)
1554027_a_at	8671	SLC4A4	solute carrier family 4 (sodium bicarbonate cotransporter), member 4
1565000_a_at	255394	TCP11L2	t-complex 11, testis-specific-like 2
224242_at	85569	GALP	galanin-like peptide
224532_at	83844	USP26	ubiquitin specific peptidase 26
1555417_a_at	80835	TAS1R1	taste receptor, type 1, member 1
1569470_a_at	84978	FRMD5	FERM domain containing 5
221586_s_at	1875	E2F5	E2F transcription factor 5, p130-binding
210499_s_at	10084	PQBP1	polyglutamine binding protein 1
239400_at	729220	FLJ45513	uncharacterized LOC729220
213820_s_at	80765	STARD5	StAR-related lipid transfer (START) domain containing 5
211446_at	353299	RGSL1	regulator of G-protein signaling like 1
236320_at	149483	CCDC17	coiled-coil domain containing 17
1552742_at	131096	KCNH8	potassium voltage-gated channel, subfamily H (eag-related), member 8
1562916_at	100359394	LINC00102	long intergenic non-protein coding RNA 102
1564339_a_at	1131	CHRM3	cholinergic receptor, muscarinic 3
220449_at	79015	LINC01260	long intergenic non-protein coding RNA 1260
214186_s_at	352961	HCG26	HLA complex group 26 (non-protein coding)
213607_x_at	65220	NADK	NAD kinase
206835_at	6779	STATH	statherin
1554491_a_at	462	SERPINC1	serpin peptidase inhibitor, clade C (antithrombin), member 1
215699_x_at	9814	SFI1	Sfi1 homolog, spindle assembly associated (yeast)
1559654_s_at	100874068	GRTP1-AS1	GRTP1 antisense RNA 1
232234_at	84174	SLA2	Src-like-adaptor 2
206623_at	5145	PDE6A	phosphodiesterase 6A, cGMP-specific, rod, alpha
240219_at	100506697	LINC00327	long intergenic non-protein coding RNA 327
213670_x_at	155400	NSUN5P1	NOP2/Sun domain family, member 5 pseudogene 1
208134_x_at	5670	PSG2	pregnancy specific beta-1-glycoprotein 2
223938_at	84066	TEX35	testis expressed 35
206937_at	6708	SPTA1	spectrin, alpha, erythrocytic 1
1566927_at	54748	LINC00527	long intergenic non-protein coding RNA 527
204070_at	5920	RARRES3	retinoic acid receptor responder (tazarotene induced) 3
206082_at	10866	HCP5	HLA complex P5 (non-protein coding)
222838_at	57823	SLAMF7	SLAM family member 7
239564_at	100128198	LOC100128198	uncharacterized LOC100128198
1554116_s_at	57097	PARP11	poly (ADP-ribose) polymerase family, member 11
206118_at	6775	STAT4	signal transducer and activator of transcription 4
241601_at	644150	WIPF3	WAS/WASL interacting protein family, member 3
211565_at	6457	SH3GL3	SH3-domain GRB2-like 3
1556900_at	149773	APCDD1L-AS1	APCDD1L antisense RNA 1 (head to head)
208529_at	690	BTF3P11	basic transcription factor 3 pseudogene 11
205697_at	10590	SCGN	secretagogin, EF-hand calcium binding protein
212443_at	23218	NBEAL2	neurobeachin-like 2
242660_at	340895	MALRD1	MAM and LDL receptor class A domain containing 1
1554307_at	644852	LOC644852	uncharacterized LOC644852
221378_at	9350	CER1	cerberus 1, DAN family BMP antagonist
1560832_at	101927715	LINC01029	long intergenic non-protein coding RNA 1029

1557162_at	146378	C16orf92	chromosome 16 open reading frame 92
207093_s_at	4974	OMG	oligodendrocyte myelin glycoprotein
1564044_at	148930	KNCN	kinocilin
216402_at	284904	SEC14L4	SEC14-like 4 (S. cerevisiae)
231977_at	2894	GRID1	glutamate receptor, ionotropic, delta 1
1554145_a_at	129285	PPP1R21	protein phosphatase 1, regulatory subunit 21
228598_at	57628	DPP10	dipeptidyl-peptidase 10 (non-functional)
231514_at	84970	C1orf94	chromosome 1 open reading frame 94
224357_s_at	51338	MS4A4A	membrane-spanning 4-domains, subfamily A, member 4A
208448_x_at	3449	IFNA16	interferon, alpha 16
230432_at	100422737	LOC100422737	uncharacterized LOC100422737
236613_at	58517	RBM25	RNA binding motif protein 25
1569617_at	23762	OSBP2	oxysterol binding protein 2
215855_s_at	7110	TMF1	TATA element modulatory factor 1
228410_at	139716	GAB3	GRB2-associated binding protein 3
230325_at	100133985	LOC100133985	uncharacterized LOC100133985
215504_x_at	100505494	ANKRD10-IT1	ANKRD10 intronic transcript 1 (non-protein coding)
220091_at	11182	SLC2A6	solute carrier family 2 (facilitated glucose transporter), member 6
229729_at	51754	TMEM8B	transmembrane protein 8B
230839_at	56341	PRMT8	protein arginine methyltransferase 8
206435_at	2583	B4GALNT1	beta-1,4-N-acetyl-galactosaminyl transferase 1
206650_at	55721	IQCC	IQ motif containing C
1556687_a_at	9071	CLDN10	claudin 10
219734_at	54847	SIDT1	SID1 transmembrane family, member 1
207868_at	1135	CHRNA2	cholinergic receptor, nicotinic, alpha 2 (neuronal)
225931_s_at	57674	RNF213	ring finger protein 213
1553209_at	84900	RNFT2	ring finger protein, transmembrane 2
241550_at	340168	DPPA5	developmental pluripotency associated 5
203387_s_at	9882	TBC1D4	TBC1 domain family, member 4
1560449_at	101927543	LOC101927543	uncharacterized LOC101927543
1569426_at	100996342	LOC100996342	uncharacterized LOC100996342
214636_at	797	CALCB	calcitonin-related polypeptide beta
215243_s_at	2707	GJB3	gap junction protein, beta 3, 31kDa
238241_at	100144602	EPHA5-AS1	EPHA5 antisense RNA 1
1555291_at	162514	TRPV3	transient receptor potential cation channel, subfamily V, member 3
1564449_at	100499227	USP2-AS1	USP2 antisense RNA 1 (head to head)
243803_at	643037	C11orf97	chromosome 11 open reading frame 97
231195_at	346689	KLRG2	killer cell lectin-like receptor subfamily G, member 2
232121_at	1787	TRDMT1	tRNA aspartic acid methyltransferase 1
1552811_at	117166	WFIKKN1	WAP, follistatin/kazal, immunoglobulin, kunitz and netrin domain containing 1
204887_s_at	10733	PLK4	polo-like kinase 4
213676_at	441151	TMEM151B	transmembrane protein 151B
1565775_at	101927410	LOC101927410	uncharacterized LOC101927410
213721_at	6657	SOX2	SRY (sex determining region Y)-box 2
206504_at	1591	CYP24A1	cytochrome P450, family 24, subfamily A, polypeptide 1
1562697_at	339988	LOC339988	uncharacterized LOC339988
202957_at	3059	HCLS1	hematopoietic cell-specific Lyn substrate 1
213441_x_at	25803	SPDEF	SAM pointed domain containing ETS transcription factor
210107_at	1179	CLCA1	chloride channel accessory 1
215826_x_at	90485	ZNF835	zinc finger protein 835
1552903_at	124872	B4GALNT2	beta-1,4-N-acetyl-galactosaminyl transferase 2
219191_s_at	51411	BIN2	bridging integrator 2
234583_at	10752	CHL1	cell adhesion molecule L1-like
220653_at	23619	ZIM2	zinc finger, imprinted 2
207238_s_at	5788	PTPRC	protein tyrosine phosphatase, receptor type, C
232037_at	9543	IGDCC3	immunoglobulin superfamily, DCC subclass, member 3
1560089_at	100289019	SLC25A25-AS1	SLC25A25 antisense RNA 1
205305_at	2267	FGL1	fibrinogen-like 1
221323_at	80329	ULBP1	UL16 binding protein 1
35150_at	958	CD40	CD40 molecule, TNF receptor superfamily member 5
1555071_at	7092	TLL1	tolloid-like 1

207184_at	6540	SLC6A13	solute carrier family 6 (neurotransmitter transporter), member 13
1562573_at	1586	CYP17A1	cytochrome P450, family 17, subfamily A, polypeptide 1
234116_at	219770	GJD4	gap junction protein, delta 4, 40.1kDa
203418_at	890	CCNA2	cyclin A2
232215_x_at	55771	PRR11	proline rich 11
228212_at	145501	ISM2	isthmin 2
220790_s_at	64232	MS4A5	membrane-spanning 4-domains, subfamily A, member 5
1560316_s_at	113263	GLCCI1	glucocorticoid induced transcript 1
231946_at	85446	ZFHX2	zinc finger homeobox 2
230473_s_at	127540	HMGB4	high mobility group box 4
205576_at	3053	SERPIND1	serpin peptidase inhibitor, clade D (heparin cofactor), member 1
203892_at	10406	WFDC2	WAP four-disulfide core domain 2
223562_at	64098	PARVG	parvin, gamma
209800_at	3868	KRT16	keratin 16
206588_at	1618	DAZL	deleted in azoospermia-like
1570207_at	391059	FRRS1	ferric-chelate reductase 1
239293_at	140767	NRSN1	neurensin 1
1564504_at	51802	ASIC5	acid-sensing (proton-gated) ion channel family member 5
229456_s_at	23576	DDAH1	dimethylarginine dimethylaminohydrolase 1

Table 2: Myeloid Genes and Probes

ProbeId	EntrezId	Symbol	GeneName
235022_at	125228	FAM210A	family with sequence similarity 210, member A
210845_s_at	5329	PLAUR	plasminogen activator, urokinase receptor
216598_s_at	6347	CCL2	chemokine (C-C motif) ligand 2
202030_at	10295	BCKDK	branched chain ketoacid dehydrogenase kinase
55081_at	85377	MICALL1	MICAL-like 1
1568574_x_at	6696	SPP1	secreted phosphoprotein 1
208075_s_at	6354	CCL7	chemokine (C-C motif) ligand 7
214974_x_at	6374	CXCL5	chemokine (C-X-C motif) ligand 5
202679_at	4864	NPC1	Niemann-Pick disease, type C1
219622_at	55647	RAB20	RAB20, member RAS oncogene family
208308_s_at	2821	GPI	glucose-6-phosphate isomerase
212112_s_at	23673	STX12	syntaxin 12
205220_at	8843	HCAR3	hydroxycarboxylic acid receptor 3
212657_s_at	3557	IL1RN	interleukin 1 receptor antagonist
1554452_a_at	29923	HILPDA	hypoxia inducible lipid droplet-associated
214038_at	6355	CCL8	chemokine (C-C motif) ligand 8
224880_at	5898	RALA	v-ral simian leukemia viral oncogene homolog A (ras related)
226152_at	145567	TTC7B	tetratricopeptide repeat domain 7B
218240_at	28511	NKIRAS2	NFKB inhibitor interacting Ras-like 2
213338_at	25907	TMEM158	transmembrane protein 158 (gene/pseudogene)
203775_at	10165	SLC25A13	solute carrier family 25 (aspartate/glutamate carrier), member 13
221539_at	1978	EIF4EBP1	eukaryotic translation initiation factor 4E binding protein 1
218374_s_at	57102	C12orf4	chromosome 12 open reading frame 4
218763_at	53407	STX18	syntaxin 18
205633_s_at	211	ALAS1	aminolevulinate, delta-, synthase 1
205681_at	597	BCL2A1	BCL2-related protein A1
202859_x_at	3576	CXCL8	chemokine (C-X-C motif) ligand 8
203234_at	7378	UPP1	uridine phosphorylase 1
224815_at	149951	COMMD7	COMM domain containing 7
202934_at	3099	HK2	hexokinase 2
228152_s_at	91351	DDX60L	DEAD (Asp-Glu-Ala-Asp) box polypeptide 60-like
227463_at	1636	ACE	angiotensin I converting enzyme
219026_s_at	9462	RASAL2	RAS protein activator like 2
210002_at	2627	GATA6	GATA binding protein 6
212501_at	1051	CEBPB	CCAAT/enhancer binding protein (C/EBP), beta
204999_s_at	22809	ATF5	activating transcription factor 5
215966_x_at	2713	GK3P	glycerol kinase 3 pseudogene
1553715_s_at	84331	FAM195A	family with sequence similarity 195, member A
222601_at	55236	UBA6	ubiquitin-like modifier activating enzyme 6
223767_at	53831	GPR84	G protein-coupled receptor 84
226275_at	4084	MXD1	MAX dimerization protein 1
235463_s_at	253782	CERS6	ceramide synthase 6
203045_at	4814	NINJ1	ninjurin 1
214681_at	2710	GK	glycerol kinase
219706_at	55317	AP5S1	adaptor-related protein complex 5, sigma 1 subunit
201201_at	1476	CSTB	cystatin B (stefin B)
218909_at	26750	RPS6KC1	ribosomal protein S6 kinase, 52kDa, polypeptide 1
207543_s_at	5033	P4HA1	prolyl 4-hydroxylase, alpha polypeptide I
219208_at	80204	FBXO11	F-box protein 11
227143_s_at	637	BID	BH3 interacting domain death agonist
219890_at	23601	CLEC5A	C-type lectin domain family 5, member A
222646_s_at	30001	ERO1L	ERO1-like (S. cerevisiae)
201751_at	9929	JOSD1	Josephin domain containing 1
220358_at	55509	BATF3	basic leucine zipper transcription factor, ATF-like 3
210512_s_at	7422	VEGFA	vascular endothelial growth factor A
212048_s_at	8565	YARS	tyrosyl-tRNA synthetase
221050_s_at	54676	GTPBP2	GTP binding protein 2
207850_at	2921	CXCL3	chemokine (C-X-C motif) ligand 3
205479_s_at	5328	PLAU	plasminogen activator, urokinase

218447_at	56942	CMC2	C-x(9)-C motif containing 2
221504_s_at	51606	ATP6V1H	ATPase, H+ transporting, lysosomal 50/57kDa, V1 subunit H
226416_at	90459	ERI1	exoribonuclease 1
214269_at	84179	MFSD7	major facilitator superfamily domain containing 7
223065_s_at	83930	STARD3NL	STARD3 N-terminal like
201642_at	3460	IFNGR2	interferon gamma receptor 2 (interferon gamma transducer 1)
218627_at	55332	DRAM1	DNA-damage regulated autophagy modulator 1
219874_at	84561	SLC12A8	solute carrier family 12, member 8
219256_s_at	54436	SH3TC1	SH3 domain and tetratricopeptide repeats 1
219631_at	29967	LRP12	low density lipoprotein receptor-related protein 12
207075_at	114548	NLRP3	NLR family, pyrin domain containing 3
225612_s_at	84002	B3GNT5	UDP-GlcNAc:betaGal beta-1,3-N-acetylglucosaminyltransferase 5
205126_at	7444	VRK2	vaccinia related kinase 2
212723_at	23210	JMJD6	jumonji domain containing 6
204146_at	10635	RAD51AP1	RAD51 associated protein 1
217962_at	55505	NOP10	NOP10 ribonucleoprotein
202043_s_at	6611	SMS	spermine synthase
205349_at	2769	GNA15	guanine nucleotide binding protein (G protein), alpha 15 (Gq class)
225252_at	140809	SRXN1	sulfiredoxin 1
214830_at	145389	SLC38A6	solute carrier family 38, member 6
226140_s_at	220213	OTUD1	OTU deubiquitinase 1
202637_s_at	3383	ICAM1	intercellular adhesion molecule 1
217078_s_at	11314	CD300A	CD300a molecule
202790_at	1366	CLDN7	claudin 7
204027_s_at	4234	METTL1	methyltransferase like 1
225870_s_at	126003	TRAPPC5	trafficking protein particle complex 5
204565_at	55856	ACOT13	acyl-CoA thioesterase 13
223682_s_at	84285	EIF1AD	eukaryotic translation initiation factor 1A domain containing
219956_at	11226	GALNT6	polypeptide N-acetylgalactosaminyltransferase 6
1569095_at	731424	LOC731424	uncharacterized LOC731424
218815_s_at	55092	TMEM51	transmembrane protein 51
203695_s_at	1687	DFNA5	deafness, autosomal dominant 5
224392_s_at	23596	OPN3	opsin 3
213201_s_at	7138	TNNT1	troponin T type 1 (skeletal, slow)
214430_at	2717	GLA	galactosidase, alpha
225824_at	8812	CCNK	cyclin K
228578_at	129831	RBM45	RNA binding motif protein 45
39402_at	3553	IL1B	interleukin 1, beta
222608_s_at	54443	ANLN	anillin, actin binding protein
218996_at	29844	TFPT	TCF3 (E2A) fusion partner (in childhood Leukemia)
202499_s_at	6515	SLC2A3	solute carrier family 2 (facilitated glucose transporter), member 3
209615_s_at	5058	PAK1	p21 protein (Cdc42/Rac)-activated kinase 1
220066_at	64127	NOD2	nucleotide-binding oligomerization domain containing 2
236077_at	2595	GANC	glucosidase, alpha; neutral C
221805_at	4747	NEFL	neurofilament, light polypeptide
201761_at	10797	MTHFD2	methylenetetrahydrofolate dehydrogenase (NADP+ dependent) 2, methenyltetrahydrofolate cyclohydrolase
218072_at	29099	COMMD9	COMM domain containing 9
223944_at	91662	NLRP12	NLR family, pyrin domain containing 12
210184_at	3687	ITGAX	integrin, alpha X (complement component 3 receptor 4 subunit)
209696_at	2203	FBP1	fructose-1,6-bisphosphatase 1
208315_x_at	7187	TRAF3	TNF receptor-associated factor 3
224374_s_at	84034	EMILIN2	elastin microfibril interfacer 2
227940_at	339803	LOC339803	uncharacterized LOC339803
222173_s_at	55357	TBC1D2	TBC1 domain family, member 2
230966_at	259307	IL4I1	interleukin 4 induced 1
1553769_at	89886	SLAMF9	SLAM family member 9
202120_x_at	1175	AP2S1	adaptor-related protein complex 2, sigma 1 subunit
220088_at	728	C5AR1	complement component 5a receptor 1
208932_at	5531	PPP4C	protein phosphatase 4, catalytic subunit
201454_s_at	9520	NPEPPS	aminopeptidase puromycin sensitive
218387_s_at	25796	PGLS	6-phosphogluconolactonase

209949_at	4688	NCF2	neutrophil cytosolic factor 2
225609_at	2936	GSR	glutathione reductase
243894_at	84102	SLC41A2	solute carrier family 41 (magnesium transporter), member 2
235013_at	1317	SLC31A1	solute carrier family 31 (copper transporter), member 1
226679_at	284129	SLC26A11	solute carrier family 26 (anion exchanger), member 11
223375_at	55633	TBC1D22B	TBC1 domain family, member 22B
205686_s_at	942	CD86	CD86 molecule
201463_s_at	6888	TALDO1	transaldolase 1
223241_at	29886	SNX8	sorting nexin 8
209124_at	4615	MYD88	myeloid differentiation primary response 88
212527_at	27351	DESI1	desumoylating isopeptidase 1
233176_at	100507642	LOC100507642	uncharacterized LOC100507642
226952_at	85403	EAF1	ELL associated factor 1
203282_at	2632	GBE1	glucan (1,4-alpha-), branching enzyme 1
211982_x_at	23214	XPO6	exportin 6
227185_at	643988	C1orf233	chromosome 1 open reading frame 233
231423_s_at	54522	ANKRD16	ankyrin repeat domain 16
212832_s_at	9793	CKAP5	cytoskeleton associated protein 5
37966_at	29780	PARVB	parvin, beta
220865_s_at	23590	PDSS1	prenyl (decaprenyl) diphosphate synthase, subunit 1
206173_x_at	2553	GABPB1	GA binding protein transcription factor, beta subunit 1
204580_at	4321	MMP12	matrix metallopeptidase 12 (macrophage elastase)
203978_at	4682	NUBP1	nucleotide binding protein 1
218945_at	79091	METTL22	methyltransferase like 22
204958_at	1263	PLK3	polo-like kinase 3
237731_at	154092	LINC01010	long intergenic non-protein coding RNA 1010
36566_at	1497	CTNS	cystinosin, lysosomal cystine transporter
218799_at	54707	GPN2	GPN-loop GTPase 2
209193_at	5292	PIM1	Pim-1 proto-oncogene, serine/threonine kinase
202205_at	7408	VASP	vasodilator-stimulated phosphoprotein
225201_s_at	64928	MRPL14	mitochondrial ribosomal protein L14
209053_s_at	7468	WHSC1	Wolf-Hirschhorn syndrome candidate 1
229845_at	79109	MAPKAP1	mitogen-activated protein kinase associated protein 1
207945_s_at	1453	CSNK1D	casein kinase 1, delta
218394_at	79641	ROGDI	rogdi homolog (Drosophila)
205476_at	6364	CCL20	chemokine (C-C motif) ligand 20
212552_at	3241	HPCAL1	hippocalcin-like 1
207610_s_at	30817	EMR2	egf-like module containing, mucin-like, hormone receptor-like 2
209928_s_at	9242	MSC	musculin
202153_s_at	23636	NUP62	nucleoporin 62kDa
200733_s_at	7803	PTP4A1	protein tyrosine phosphatase type IVA, member 1
203746_s_at	3052	HCCS	holocytochrome c synthase
209045_at	7511	XPNPEP1	X-prolyl aminopeptidase (aminopeptidase P) 1, soluble
212576_at	23295	MGRN1	mahogunin ring finger 1, E3 ubiquitin protein ligase
223064_at	51255	RNF181	ring finger protein 181
205099_s_at	1230	CCR1	chemokine (C-C motif) receptor 1
210273_at	5099	PCDH7	protocadherin 7
219842_at	54622	ARL15	ADP-ribosylation factor-like 15
214226_at	339105	PRSS53	protease, serine, 53
227046_at	201266	SLC39A11	solute carrier family 39, member 11
229860_x_at	401115	C4orf48	chromosome 4 open reading frame 48
1554503_a_at	126014	OSCAR	osteoclast associated, immunoglobulin-like receptor
204194_at	571	BACH1	BTB and CNC homology 1, basic leucine zipper transcription factor 1
201379_s_at	7165	TPD52L2	tumor protein D52-like 2
204300_at	5188	GATB	glutamyl-tRNA(Gln) amidotransferase, subunit B
205131_x_at	6320	CLEC11A	C-type lectin domain family 11, member A
237252_at	7056	THBD	thrombomodulin
222767_s_at	79794	C12orf49	chromosome 12 open reading frame 49
228707_at	137075	CLDN23	claudin 23
223491_at	51122	COMMD2	COMM domain containing 2
206632_s_at	9582	APOBEC3B	apolipoprotein B mRNA editing enzyme, catalytic polypeptide-like 3B
204715_at	24145	PANX1	pannexin 1

202345_s_at	2171	FABP5	fatty acid binding protein 5 (psoriasis-associated)
1560060_s_at	55048	VPS37C	vacuolar protein sorting 37 homolog C (S. cerevisiae)
205745_x_at	6868	ADAM17	ADAM metallopeptidase domain 17
203530_s_at	6810	STX4	syntaxin 4
223097_at	54936	ADPRHL2	ADP-ribosylhydrolase like 2
221345_at	2867	FFAR2	free fatty acid receptor 2
223454_at	58191	CXCL16	chemokine (C-X-C motif) ligand 16
225168_at	55691	FRMD4A	FERM domain containing 4A
222216_s_at	63875	MRPL17	mitochondrial ribosomal protein L17
204247_s_at	1020	CDK5	cyclin-dependent kinase 5
219788_at	29992	PILRA	paired immunoglobin-like type 2 receptor alpha
205485_at	6261	RYR1	ryanodine receptor 1 (skeletal)
1568592_at	140691	TRIM69	tripartite motif containing 69
225195_at	285381	DPH3	diphthamide biosynthesis 3
201390_s_at	1460	CSNK2B	casein kinase 2, beta polypeptide
203714_s_at	6905	TBCE	tubulin folding cofactor E
217968_at	7260	TSSC1	tumor suppressing subtransferable candidate 1
206067_s_at	7490	WT1	Wilms tumor 1
234950_s_at	64326	RFWD2	ring finger and WD repeat domain 2, E3 ubiquitin protein ligase
208478_s_at	581	BAX	BCL2-associated X protein

Table 3: Fibroid Genes and Probes

ProbeId	EntrezId	Symbol	GeneName
219525_at	55244	SLC47A1	solute carrier family 47 (multidrug and toxin extrusion), member 1
231798_at	9241	NOG	noggin
217367_s_at	23051	ZHX3	zinc fingers and homeoboxes 3
228255_at	65062	TMEM237	transmembrane protein 237
209018_s_at	65018	PINK1	PTEN induced putative kinase 1
230783_at	283713	LOC283713	uncharacterized LOC283713
209883_at	23127	COLGALT2	collagen beta(1-O)galactosyltransferase 2
236325_at	57562	KIAA1377	KIAA1377
203420_at	51439	FAM8A1	family with sequence similarity 8, member A1
217795_s_at	79188	TMEM43	transmembrane protein 43
212850_s_at	4038	LRP4	low density lipoprotein receptor-related protein 4
212345_s_at	64764	CREB3L2	cAMP responsive element binding protein 3-like 2
213800_at	3075	CFH	complement factor H
225546_at	29904	EEF2K	eukaryotic elongation factor-2 kinase
226402_at	113612	CYP2U1	cytochrome P450, family 2, subfamily U, polypeptide 1
200642_at	6647	SOD1	superoxide dismutase 1, soluble
213298_at	4782	NFIC	nuclear factor I/C (CCAAT-binding transcription factor)
227108_at	57519	STARD9	StAR-related lipid transfer (START) domain containing 9
212136_at	493	ATP2B4	ATPase, Ca++ transporting, plasma membrane 4
226018_at	222166	MTURN	maturin, neural progenitor differentiation regulator homolog (Xenopus)
201561_s_at	22883	CLSTN1	calsyntenin 1
206176_at	654	BMP6	bone morphogenetic protein 6
230121_at	574036	SERTAD4-AS1	SERTAD4 antisense RNA 1
222453_at	79901	CYBRD1	cytochrome b reductase 1
64900_at	79583	TMEM231	transmembrane protein 231
1557382_x_at	119385	AGAP11	ankyrin repeat and GTPase domain Arf GTPase activating protein 11
224755_at	56889	TM9SF3	transmembrane 9 superfamily member 3
222890_at	29070	CCDC113	coiled-coil domain containing 113
209355_s_at	8613	PPAP2B	phosphatidic acid phosphatase type 2B
212205_at	94239	H2AFV	H2A histone family, member V
204469_at	5803	PTPRZ1	protein tyrosine phosphatase, receptor-type, Z polypeptide 1
213176_s_at	8425	LTBP4	latent transforming growth factor beta binding protein 4
228890_at	84913	ATOH8	atonal homolog 8 (Drosophila)
212437_at	1059	CENPB	centromere protein B, 80kDa
228760_at	10929	SRSF8	serine/arginine-rich splicing factor 8
231118_at	148741	ANKRD35	ankyrin repeat domain 35
219038_at	79710	MORC4	MORC family CW-type zinc finger 4
218692_at	55638	SYBU	syntabulin (syntaxin-interacting)
227401_at	53342	IL17D	interleukin 17D
207788_s_at	10174	SORBS3	sorbin and SH3 domain containing 3
224804_s_at	57184	FAM219B	family with sequence similarity 219, member B
230454_at	130026	ICA1L	islet cell autoantigen 1,69kDa-like
204731_at	7049	TGFBR3	transforming growth factor, beta receptor III
228615_at	286161	LOC286161	uncharacterized LOC286161
206159_at	2662	GDF10	growth differentiation factor 10
218307_at	55316	RSAD1	radical S-adenosyl methionine domain containing 1
239461_at	117248	GALNT15	polypeptide N-acetylgalactosaminyltransferase 15
223693_s_at	55698	RADIL	Ras association and DIL domains
201022_s_at	11034	DSTN	destrin (actin depolymerizing factor)
221519_at	6468	FBXW4	F-box and WD repeat domain containing 4
204547_at	10966	RAB40B	RAB40B, member RAS oncogene family
230660_at	56256	SERTAD4	SERTA domain containing 4
205878_at	5463	POU6F1	POU class 6 homeobox 1
217844_at	58190	CTDSP1	CTD (carboxy-terminal domain, RNA polymerase II, polypeptide A) small phosphatase 1
200758_s_at	4779	NFE2L1	nuclear factor, erythroid 2-like 1
204284_at	5507	PPP1R3C	protein phosphatase 1, regulatory subunit 3C
226030_at	36	ACADSB	acyl-CoA dehydrogenase, short/branched chain
212726_at	5253	PHF2	PHD finger protein 2

1554574_a_at	1727	CYB5R3	cytochrome b5 reductase 3
231906_at	3234	HOXD8	homeobox D8
229310_at	114818	KLHL29	kelch-like family member 29
201412_at	26020	LRP10	low density lipoprotein receptor-related protein 10
201557_at	6844	VAMP2	vesicle-associated membrane protein 2 (synaptobrevin 2)
210155_at	4653	MYOC	myocilin, trabecular meshwork inducible glucocorticoid response
1554079_at	374378	GALNT18	polypeptide N-acetylgalactosaminyltransferase 18
229032_at	9671	WSCD2	WSC domain containing 2
204310_s_at	4882	NPR2	natriuretic peptide receptor 2
219440_at	10742	RAI2	retinoic acid induced 2
207797_s_at	55805	LRP2BP	LRP2 binding protein
219563_at	79686	LINC00341	long intergenic non-protein coding RNA 341
205158_at	6038	RNASE4	ribonuclease, RNase A family, 4
203151_at	4130	MAP1A	microtubule-associated protein 1A
200911_s_at	6867	TACC1	transforming, acidic coiled-coil containing protein 1
212229_s_at	23014	FBXO21	F-box protein 21
207842_s_at	22794	CASC3	cancer susceptibility candidate 3
219488_at	53947	A4GALT	alpha 1,4-galactosyltransferase
227198_at	3899	AFF3	AF4/FMR2 family, member 3
225391_at	93622	LOC93622	Morf4 family associated protein 1-like 1 pseudogene
218651_s_at	55323	LARP6	La ribonucleoprotein domain family, member 6
205412_at	38	ACAT1	acetyl-CoA acetyltransferase 1
213675_at	55742	PARVA	parvin, alpha
228131_at	2067	ERCC1	excision repair cross-complementation group 1
227758_at	85004	RERG	RAS-like, estrogen-regulated, growth inhibitor
210299_s_at	2273	FHL1	four and a half LIM domains 1
204154_at	1036	CDO1	cysteine dioxygenase type 1
230480_at	143689	PIWIL4	piwi-like RNA-mediated gene silencing 4
225587_at	92305	TMEM129	transmembrane protein 129, E3 ubiquitin protein ligase
207606_s_at	94134	ARHGAP12	Rho GTPase activating protein 12
204270_at	6497	SKI	SKI proto-oncogene
230351_at	283481	FGF14-AS2	FGF14 antisense RNA 2
223063_at	84886	C1orf198	chromosome 1 open reading frame 198
205854_at	7289	TULP3	tubby like protein 3
228857_at	2794	GNL1	guanine nucleotide binding protein-like 1
225134_at	84926	SPRYD3	SPRY domain containing 3
212128_s_at	1605	DAG1	dystroglycan 1 (dystrophin-associated glycoprotein 1)
218686_s_at	64285	RHBDF1	rhomboid 5 homolog 1 (Drosophila)
218180_s_at	64787	EPS8L2	EPS8-like 2
212239_at	5295	PIK3R1	phosphoinositide-3-kinase, regulatory subunit 1 (alpha)
228637_at	29800	ZDHHC1	zinc finger, DHHC-type containing 1
212848_s_at	84909	C9orf3	chromosome 9 open reading frame 3
204343_at	21	ABCA3	ATP-binding cassette, sub-family A (ABC1), member 3
206227_at	8483	CILP	cartilage intermediate layer protein, nucleotide pyrophosphohydrolase
213497_at	25841	ABTB2	ankyrin repeat and BTB (POZ) domain containing 2
221216_s_at	22955	SCMH1	sex comb on midleg homolog 1 (Drosophila)
204933_s_at	4982	TNFRSF11B	tumor necrosis factor receptor superfamily, member 11b
213405_at	57403	RAB22A	RAB22A, member RAS oncogene family
212504_at	22982	DIP2C	DIP2 disco-interacting protein 2 homolog C (Drosophila)
204223_at	5549	PRELP	proline/arginine-rich end leucine-rich repeat protein
201802_at	2030	SLC29A1	solute carrier family 29 (equilibrative nucleoside transporter), member 1
210879_s_at	26056	RAB11FIP5	RAB11 family interacting protein 5 (class I)
218656_s_at	10186	LHFP	lipoma HMGIC fusion partner
1552789_at	7095	SEC62	SEC62 homolog (S. cerevisiae)
244569_at	157657	C8orf37	chromosome 8 open reading frame 37
228977_at	729680	LOC729680	uncharacterized LOC729680
225914_s_at	81617	CAB39L	calcium binding protein 39-like
217891_at	64755	C16orf58	chromosome 16 open reading frame 58
231817_at	54532	USP53	ubiquitin specific peptidase 53
227713_at	84056	KATNAL1	katanin p60 subunit A-like 1
222423_at	80762	NDFIP1	Nedd4 family interacting protein 1
224719_s_at	113246	C12orf57	chromosome 12 open reading frame 57

224871_at	127262	TPRG1L	tumor protein p63 regulated 1-like
203883_s_at	22841	RAB11FIP2	RAB11 family interacting protein 2 (class I)
219295_s_at	26577	PCOLCE2	procollagen C-endopeptidase enhancer 2
209283_at	1410	CRYAB	crystallin, alpha B
214954_at	26032	SUSD5	sushi domain containing 5
209675_s_at	11100	HNRNPUL1	heterogeneous nuclear ribonucleoprotein U-like 1
222450_at	56937	PMEPA1	prostate transmembrane protein, androgen induced 1
206414_s_at	8853	ASAP2	ArfGAP with SH3 domain, ankyrin repeat and PH domain 2
201215_at	5358	PLS3	plastin 3
233825_s_at	83692	CD99L2	CD99 molecule-like 2
203688_at	5311	PKD2	polycystic kidney disease 2 (autosomal dominant)
228107_at	100127983	C8orf88	chromosome 8 open reading frame 88
203571_s_at	10974	ADIRF	adipogenesis regulatory factor
200762_at	1808	DPYSL2	dihydropyrimidinase-like 2
226360_at	84133	ZNRF3	zinc and ring finger 3
227400_at	4784	NFIX	nuclear factor I/X (CCAAT-binding transcription factor)
240806_at	6138	RPL15	ribosomal protein L15
208790_s_at	284119	PTRF	polymerase I and transcript release factor
200696_s_at	2934	GSN	gelsolin
212730_at	23336	SYNM	synemin, intermediate filament protein
201906_s_at	10217	CTDSPL	CTD (carboxy-terminal domain, RNA polymerase II, polypeptide A) small phosphatase-like
216264_s_at	3913	LAMB2	laminin, beta 2 (laminin S)
202291_s_at	4256	MGP	matrix Gla protein
226763_at	91404	SESTD1	SEC14 and spectrin domains 1
225132_at	26224	FBXL3	F-box and leucine-rich repeat protein 3
218418_s_at	25959	KANK2	KN motif and ankyrin repeat domains 2
210461_s_at	3983	ABLIM1	actin binding LIM protein 1
226426_at	23394	ADNP	activity-dependent neuroprotector homeobox
227195_at	84858	ZNF503	zinc finger protein 503
226282_at	5784	PTPN14	protein tyrosine phosphatase, non-receptor type 14
205236_x_at	6649	SOD3	superoxide dismutase 3, extracellular
235968_at	116987	AGAP1	ArfGAP with GTPase domain, ankyrin repeat and PH domain 1
235725_at	4089	SMAD4	SMAD family member 4
202920_at	287	ANK2	ankyrin 2, neuronal
218432_at	26273	FBXO3	F-box protein 3
218471_s_at	582	BBS1	Bardet-Biedl syndrome 1
1554176_a_at	285315	C3orf33	chromosome 3 open reading frame 33
209335_at	1634	DCN	decorin
202915_s_at	9917	FAM20B	family with sequence similarity 20, member B
229488_at	56957	OTUD7B	OTU deubiquitinase 7B
213224_s_at	92482	BBIP1	BBSome interacting protein 1
208033_s_at	463	ZFHX3	zinc finger homeobox 3
226157_at	7029	TFDP2	transcription factor Dp-2 (E2F dimerization partner 2)
235173_at	401093	MBNL1-AS1	MBNL1 antisense RNA 1
236336_at	100507171	BOLA3-AS1	BOLA3 antisense RNA 1 (head to head)
225298_at	25953	PNKD	paroxysmal nonkinesigenic dyskinesia
207312_at	5260	PHKG1	phosphorylase kinase, gamma 1 (muscle)
203230_at	1855	DVL1	dishevelled segment polarity protein 1
231773_at	9068	ANGPTL1	angiopoietin-like 1
217816_s_at	57092	PCNP	PEST proteolytic signal containing nuclear protein
203006_at	3632	INPP5A	inositol polyphosphate-5-phosphatase, 40kDa
227029_at	283635	FAM177A1	family with sequence similarity 177, member A1
203793_x_at	7703	PCGF2	polycomb group ring finger 2
242137_at	27303	RBMS3	RNA binding motif, single stranded interacting protein 3
212936_at	83989	FAM172A	family with sequence similarity 172, member A
204462_s_at	6567	SLC16A2	solute carrier family 16, member 2 (thyroid hormone transporter)
200601_at	81	ACTN4	actinin, alpha 4
229657_at	7068	THRB	thyroid hormone receptor, beta
227526_at	50937	CDON	cell adhesion associated, oncogene regulated
232224_at	5648	MASP1	mannan-binding lectin serine peptidase 1 (C4/C2 activating component of Ra-reactive factor)

1557167_at	493812	HCG11	HLA complex group 11 (non-protein coding)
217964_at	54902	TTC19	tetratricopeptide repeat domain 19
1552455_at	158471	PRUNE2	prune homolog 2 (Drosophila)
224597_at	647979	LINC00657	long intergenic non-protein coding RNA 657
203245_s_at	266655	LINC00094	long intergenic non-protein coding RNA 94
205604_at	3235	HOXD9	homeobox D9
1552301_a_at	84940	CORO6	coronin 6
228900_at	92521	SPECC1	sperm antigen with calponin homology and coiled-coil domains 1
225534_at	114926	SMIM19	small integral membrane protein 19
200619_at	10992	SF3B2	splicing factor 3b, subunit 2, 145kDa
213208_at	23506	GLTSCR1L	GLTSCR1-like
227444_at	100131755	ARMCX4	armadillo repeat containing, X-linked 4
238076_at	57459	GATAD2B	GATA zinc finger domain containing 2B
219999_at	4122	MAN2A2	mannosidase, alpha, class 2A, member 2
217948_at	26071	FAM127B	family with sequence similarity 127, member B
201276_at	5869	RAB5B	RAB5B, member RAS oncogene family
214889_at	25854	FAM149A	family with sequence similarity 149, member A
230076_at	83394	PITPNM3	PITPNM family member 3
218820_at	56967	C14orf132	chromosome 14 open reading frame 132
36553_at	8623	ASMTL	acetylserotonin O-methyltransferase-like
200671_s_at	6711	SPTBN1	spectrin, beta, non-erythrocytic 1
202893_at	10497	UNC13B	unc-13 homolog B (C. elegans)
211941_s_at	5037	PEBP1	phosphatidylethanolamine binding protein 1
227816_at	9423	NTN1	netrin 1
202289_s_at	10579	TACC2	transforming, acidic coiled-coil containing protein 2
210619_s_at	3373	HYAL1	hyaluronoglucosaminidase 1
214682_at	339044	PKD1P1	polycystic kidney disease 1 (autosomal dominant) pseudogene 1
209071_s_at	8490	RGS5	regulator of G-protein signaling 5
207542_s_at	358	AQP1	aquaporin 1 (Colton blood group)
224560_at	7077	TIMP2	TIMP metallopeptidase inhibitor 2
224688_at	55069	TMEM248	transmembrane protein 248
225274_at	51449	PCYOX1	prenylcysteine oxidase 1
203335_at	5264	PHYH	phytanoyl-CoA 2-hydroxylase
212062_at	10079	ATP9A	ATPase, class II, type 9A
210605_s_at	4240	MFGE8	milk fat globule-EGF factor 8 protein
1559840_s_at	9096	TBX18	T-box 18
217906_at	23588	KLHDC2	kelch domain containing 2
218706_s_at	65983	GRAMD3	GRAM domain containing 3
208978_at	1397	CRIP2	cysteine-rich protein 2
222571_at	30815	ST6GALNAC6	ST6 (alpha-N-acetyl-neuraminyl-2,3-beta-galactosyl-1,3)-N-acetylgalactosaminide alpha-2,6-sialyltransferase 6
213578_at	657	BMPR1A	bone morphogenetic protein receptor, type IA
219416_at	51435	SCARA3	scavenger receptor class A, member 3
206737_at	7481	WNT11	wingless-type MMTV integration site family, member 11
202652_at	322	APBB1	amyloid beta (A4) precursor protein-binding, family B, member 1 (Fe65)
232027_at	23345	SYNE1	spectrin repeat containing, nuclear envelope 1
201874_at	9019	MPZL1	myelin protein zero-like 1
205182_s_at	25799	ZNF324	zinc finger protein 324
226091_s_at	93621	MRFAP1	Morf4 family associated protein 1
238479_at	84259	DCUN1D5	DCN1, defective in cullin neddylation 1, domain containing 5
201076_at	4809	NHP2L1	NHP2 non-histone chromosome protein 2-like 1 (S. cerevisiae)
207738_s_at	10787	NCKAP1	NCK-associated protein 1
212793_at	23500	DAAM2	dishevelled associated activator of morphogenesis 2
227976_at	644538	SMIM10	small integral membrane protein 10
201095_at	1611	DAP	death-associated protein
202709_at	2331	FMOD	fibromodulin
1554062_at	7499	XG	Xg blood group
202564_x_at	402	ARL2	ADP-ribosylation factor-like 2
207283_at	56969	RPL23AP32	ribosomal protein L23a pseudogene 32
223316_at	83643	CCDC3	coiled-coil domain containing 3
200000_s_at	10594	PRPF8	pre-mRNA processing factor 8
225376_at	54994	GID8	GID complex subunit 8

205933_at	26040	SETBP1	SET binding protein 1
239300_at	5289	PIK3C3	phosphatidylinositol 3-kinase, catalytic subunit type 3
209657_s_at	3298	HSF2	heat shock transcription factor 2
223836_at	83888	FGFBP2	fibroblast growth factor binding protein 2
235308_at	26137	ZBTB20	zinc finger and BTB domain containing 20
228307_at	90187	EMILIN3	elastin microfibril interfacer 3
212772_s_at	20	ABCA2	ATP-binding cassette, sub-family A (ABC1), member 2
202685_s_at	558	AXL	AXL receptor tyrosine kinase
212641_at	3097	HIVEP2	human immunodeficiency virus type I enhancer binding protein 2
202125_s_at	66008	TRAK2	trafficking protein, kinesin binding 2
213290_at	1292	COL6A2	collagen, type VI, alpha 2
236634_at	157773	C8orf48	chromosome 8 open reading frame 48
228630_at	7637	ZNF84	zinc finger protein 84
227351_at	730094	C16orf52	chromosome 16 open reading frame 52
202598_at	6284	S100A13	S100 calcium binding protein A13
228466_at	126626	GABPB2	GA binding protein transcription factor, beta subunit 2
224968_at	112942	CFAP36	cilia and flagella associated protein 36
235124_at	645212	EIF3J-AS1	EIF3J antisense RNA 1 (head to head)
218901_at	57088	PLSCR4	phospholipid scramblase 4
201335_s_at	23365	ARHGEF12	Rho guanine nucleotide exchange factor (GEF) 12
221667_s_at	26353	HSPB8	heat shock 22kDa protein 8
201115_at	5425	POLD2	polymerase (DNA directed), delta 2, accessory subunit
219427_at	79633	FAT4	FAT atypical cadherin 4
229586_at	80205	CHD9	chromodomain helicase DNA binding protein 9
201121_s_at	10857	PGRMC1	progesterone receptor membrane component 1
213325_at	25945	PVRL3	poliovirus receptor-related 3
230435_at	375190	FAM228B	family with sequence similarity 228, member B
212451_at	9728	SECISBP2L	SECIS binding protein 2-like
203068_at	9903	KLHL21	kelch-like family member 21
226338_at	55529	TMEM55A	transmembrane protein 55A
235360_at	389072	PLEKHM3	pleckstrin homology domain containing, family M, member 3
201618_x_at	8733	GPAA1	glycosylphosphatidylinositol anchor attachment 1
204151_x_at	1645	AKR1C1	aldo-keto reductase family 1, member C1
219626_at	79649	MAP7D3	MAP7 domain containing 3
228005_at	158586	ZXDB	zinc finger, X-linked, duplicated B
212599_at	26053	AUTS2	autism susceptibility candidate 2
209655_s_at	83604	TMEM47	transmembrane protein 47
213221_s_at	23235	SIK2	salt-inducible kinase 2
229734_at	283174	MIR4697HG	MIR4697 host gene (non-protein coding)
205573_s_at	51375	SNX7	sorting nexin 7
203895_at	5332	PLCB4	phospholipase C, beta 4
213216_at	23252	OTUD3	OTU deubiquitinase 3
213413_at	11037	STON1	stonin 1
225078_at	2013	EMP2	epithelial membrane protein 2
212838_at	23268	DNMBP	dynamin binding protein
211769_x_at	10955	SERINC3	serine incorporator 3
209361_s_at	57060	PCBP4	poly(rC) binding protein 4
1568696_at	441549	CDNF	cerebral dopamine neurotrophic factor
229459_at	25817	FAM19A5	family with sequence similarity 19 (chemokine (C-C motif)-like), member A5
227156_at	8573	CASK	calcium/calmodulin-dependent serine protein kinase (MAGUK family)
226299_at	29941	PKN3	protein kinase N3
1569256_a_at	163933	FAM43B	family with sequence similarity 43, member B
221795_at	4915	NTRK2	neurotrophic tyrosine kinase, receptor, type 2
203449_s_at	7013	TERF1	telomeric repeat binding factor (NIMA-interacting) 1
226674_at	149345	SHISA4	shisa family member 4
228334_x_at	80817	CEP44	centrosomal protein 44kDa
205824_at	3316	HSPB2	heat shock 27kDa protein 2
225589_at	57630	SH3RF1	SH3 domain containing ring finger 1
209443_at	5104	SERPINA5	serpin peptidase inhibitor, clade A (alpha-1 antiproteinase, antitrypsin), member 5
228011_at	137392	FAM92A1	family with sequence similarity 92, member A1

213058_at	23331	TTC28	tetratricopeptide repeat domain 28
219338_s_at	54839	LRRC49	leucine rich repeat containing 49
201860_s_at	5327	PLAT	plasminogen activator, tissue
231062_at	100861541	DOCK9-AS2	DOCK9 antisense RNA 2 (head to head)
209789_at	10391	CORO2B	coronin, actin binding protein, 2B
227847_at	9852	EPM2AIP1	EPM2A (laforin) interacting protein 1
201658_at	400	ARL1	ADP-ribosylation factor-like 1
204431_at	7089	TLE2	transducin-like enhancer of split 2
231018_at	342979	PALM3	paralemmin 3
202617_s_at	4204	MECP2	methyl CpG binding protein 2
227359_at	127700	OSCP1	organic solute carrier partner 1
224605_at	401152	C4orf3	chromosome 4 open reading frame 3
218641_at	65998	C11orf95	chromosome 11 open reading frame 95
226326_at	84333	PCGF5	polycomb group ring finger 5
223126_s_at	81563	C1orf21	chromosome 1 open reading frame 21
225882_at	84912	SLC35B4	solute carrier family 35 (UDP-xylose/UDP-N-acetylglucosamine transporter), member B4
212923_s_at	221749	PXDC1	PX domain containing 1
217969_at	738	VPS51	vacuolar protein sorting 51 homolog (S. cerevisiae)
211538_s_at	3306	HSPA2	heat shock 70kDa protein 2
230433_at	729970	LOC729970	hCG2028352-like
230569_at	57587	CFAP97	cilia and flagella associated protein 97
224281_s_at	51335	NGRN	neugrin, neurite outgrowth associated
226563_at	4087	SMAD2	SMAD family member 2
209343_at	80303	EFHD1	EF-hand domain family, member D1
218204_s_at	79443	FYCO1	FYVE and coiled-coil domain containing 1
209699_x_at	1646	AKR1C2	aldo-keto reductase family 1, member C2
201889_at	10447	FAM3C	family with sequence similarity 3, member C
230228_at	284297	SSC5D	scavenger receptor cysteine rich family, 5 domains
201125_s_at	3693	ITGB5	integrin, beta 5
235014_at	147727	ILF3-AS1	ILF3 antisense RNA 1 (head to head)
212202_s_at	25963	TMEM87A	transmembrane protein 87A
212690_at	23259	DDHD2	DDHD domain containing 2
213131_at	10439	OLFM1	olfactomedin 1
227151_at	257364	SNX33	sorting nexin 33
208740_at	10284	SAP18	Sin3A-associated protein, 18kDa
201749_at	1889	ECE1	endothelin converting enzyme 1
204428_s_at	3931	LCAT	lecithin-cholesterol acyltransferase
201701_s_at	10424	PGRMC2	progesterone receptor membrane component 2
224928_at	80854	SETD7	SET domain containing (lysine methyltransferase) 7
212695_at	1408	CRY2	cryptochrome circadian clock 2
221016_s_at	83439	TCF7L1	transcription factor 7-like 1 (T-cell specific, HMG-box)
218284_at	4088	SMAD3	SMAD family member 3
202047_s_at	23466	CBX6	chromobox homolog 6
226051_at	140606	SELM	selenoprotein M
203453_at	6337	SCNN1A	sodium channel, non-voltage-gated 1 alpha subunit
220945_x_at	54682	MANSC1	MANSC domain containing 1
219578_s_at	64506	CPEB1	cytoplasmic polyadenylation element binding protein 1
224754_at	6667	SP1	Sp1 transcription factor
218220_at	60314	C12orf10	chromosome 12 open reading frame 10
208873_s_at	7905	REEP5	receptor accessory protein 5
1554555_a_at	79918	SETD6	SET domain containing 6
238778_at	143098	MPP7	membrane protein, palmitoylated 7 (MAGUK p55 subfamily member 7)
222471_s_at	56888	KCMF1	potassium channel modulatory factor 1
203516_at	6640	SNTA1	syntrophin, alpha 1
203538_at	819	CAMLG	calcium modulating ligand
227727_at	116535	MRGPRF	MAS-related GPR, member F
205384_at	5348	FXYD1	FXYD domain containing ion transport regulator 1
224325_at	8325	FZD8	frizzled class receptor 8
218510_x_at	54463	FAM134B	family with sequence similarity 134, member B
200884_at	1152	CKB	creatine kinase, brain
209737_at	9863	MAGI2	membrane associated guanylate kinase, WW and PDZ domain containing 2

221823_at	90355	C5orf30	chromosome 5 open reading frame 30
244640_at	342892	ZNF850	zinc finger protein 850
227193_at	7782	SLC30A4	solute carrier family 30 (zinc transporter), member 4
202171_at	7716	VEZF1	vascular endothelial zinc finger 1
212049_at	147179	WIPF2	WAS/WASL interacting protein family, member 2
224689_at	63905	MANBAL	mannosidase, beta A, lysosomal-like
227169_at	202052	DNAJC18	DnaJ (Hsp40) homolog, subfamily C, member 18
204569_at	22858	ICK	intestinal cell (MAK-like) kinase
232208_at	57611	ISLR2	immunoglobulin superfamily containing leucine-rich repeat 2
221014_s_at	83452	RAB33B	RAB33B, member RAS oncogene family
204400_at	10278	EFS	embryonal Fyn-associated substrate
203188_at	11041	B3GNT1	UDP-GlcNAc:betaGal beta-1,3-N-acetylglucosaminyltransferase 1
202294_at	10274	STAG1	stromal antigen 1
212554_at	10486	CAP2	CAP, adenylate cyclase-associated protein, 2 (yeast)
226638_at	57636	ARHGAP23	Rho GTPase activating protein 23
223407_at	84080	ENKD1	enkurin domain containing 1
230864_at	167359	NIM1K	NIM1 serine/threonine protein kinase
239848_at	10480	EIF3M	eukaryotic translation initiation factor 3, subunit M
202985_s_at	9529	BAG5	BCL2-associated athanogene 5
225458_at	25845	PP7080	uncharacterized LOC25845
228452_at	79018	GID4	GID complex subunit 4
217866_at	79869	CPSF7	cleavage and polyadenylation specific factor 7, 59kDa
202935_s_at	6662	SOX9	SRY (sex determining region Y)-box 9
1570414_x_at	79667	KLF3-AS1	KLF3 antisense RNA 1
204570_at	1346	COX7A1	cytochrome c oxidase subunit VIIa polypeptide 1 (muscle)
202853_s_at	6259	RYK	receptor-like tyrosine kinase
226528_at	345778	MTX3	metaxin 3
214323_s_at	65110	UPF3A	UPF3 regulator of nonsense transcripts homolog A (yeast)
205880_at	5587	PRKD1	protein kinase D1
229929_at	92369	SPSB4	splA/ryanodine receptor domain and SOCS box containing 4
225389_at	90135	BTBD6	BTB (POZ) domain containing 6
200680_x_at	3146	HMGB1	high mobility group box 1
211026_s_at	11343	MGLL	monoglyceride lipase
210105_s_at	2534	FYN	FYN proto-oncogene, Src family tyrosine kinase
226676_at	25925	ZNF521	zinc finger protein 521
223441_at	26503	SLC17A5	solute carrier family 17 (acidic sugar transporter), member 5
227373_at	342371	ATXN1L	ataxin 1-like
204753_s_at	3131	HLF	hepatic leukemia factor
219998_at	29094	LGALSL	lectin, galactoside-binding-like
236124_at	153546	LOC153546	uncharacterized LOC153546
222921_s_at	23493	HEY2	hes-related family bHLH transcription factor with YRPW motif 2
204928_s_at	8273	SLC10A3	solute carrier family 10, member 3
205794_s_at	4857	NOVA1	neuro-oncological ventral antigen 1
221214_s_at	26012	NSMF	NMDA receptor synaptonuclear signaling and neuronal migration factor
227388_at	286319	TUSC1	tumor suppressor candidate 1
219929_s_at	79038	ZFYVE21	zinc finger, FYVE domain containing 21
220765_s_at	55679	LIMS2	LIM and senescent cell antigen-like domains 2
36612_at	23201	FAM168A	family with sequence similarity 168, member A
218045_x_at	5763	PTMS	parathymosin
232641_at	169270	ZNF596	zinc finger protein 596
208117_s_at	81887	LAS1L	LAS1-like (S. cerevisiae)
212928_at	23270	TSPYL4	TSPY-like 4
218466_at	79735	TBC1D17	TBC1 domain family, member 17
222916_s_at	3069	HDLBP	high density lipoprotein binding protein
202577_s_at	55308	DDX19A	DEAD (Asp-Glu-Ala-Asp) box polypeptide 19A
202609_at	2059	EPS8	epidermal growth factor receptor pathway substrate 8
226341_at	100506365	OTUD6B-AS1	OTUD6B antisense RNA 1 (head to head)
209274_s_at	81689	ISCA1	iron-sulfur cluster assembly 1
203762_s_at	51626	DYNC2LI1	dynein, cytoplasmic 2, light intermediate chain 1
235142_at	653121	ZBTB8A	zinc finger and BTB domain containing 8A
205097_at	1836	SLC26A2	solute carrier family 26 (anion exchanger), member 2

212426_s_at	10971	YWHAQ	tyrosine 3-monooxygenase/tryptophan 5-monooxygenase activation protein, theta
218823_s_at	54793	KCTD9	potassium channel tetramerization domain containing 9
226913_s_at	30812	SOX8	SRY (sex determining region Y)-box 8
205330_at	4330	MN1	meningioma (disrupted in balanced translocation) 1
212325_at	22998	LIMCH1	LIM and calponin homology domains 1
240115_at	100286925	LOC100286925	uncharacterized LOC100286925
219569_s_at	80723	SLC35G2	solute carrier family 35, member G2
206766_at	8515	ITGA10	integrin, alpha 10
221769_at	90864	SPSB3	splA/ryanodine receptor domain and SOCS box containing 3
225479_at	116064	LRRC58	leucine rich repeat containing 58
201578_at	5420	PODXL	podocalyxin-like
211320_s_at	10076	PTPRU	protein tyrosine phosphatase, receptor type, U
222392_x_at	64065	PERP	PERP, TP53 apoptosis effector
225406_at	57045	TWSG1	twisted gastrulation BMP signaling modulator 1
205451_at	4303	FOXO4	forkhead box O4
227240_at	25791	NGEF	neuronal guanine nucleotide exchange factor
227419_x_at	219348	PLAC9	placenta-specific 9
204042_at	10810	WASF3	WAS protein family, member 3
218262_at	64777	RMND5B	required for meiotic nuclear division 5 homolog B (S. cerevisiae)
223894_s_at	64400	AKTIP	AKT interacting protein
223283_s_at	10194	TSHZ1	teashirt zinc finger homeobox 1
227326_at	439921	MXRA7	matrix-remodelling associated 7
203035_s_at	10401	PIAS3	protein inhibitor of activated STAT, 3
218694_at	51309	ARMCX1	armadillo repeat containing, X-linked 1
227702_at	260293	CYP4X1	cytochrome P450, family 4, subfamily X, polypeptide 1
212338_at	4642	MYO1D	myosin ID
212458_at	200734	SPRED2	sprouty-related, EVH1 domain containing 2
209543_s_at	947	CD34	CD34 molecule
212866_at	203069	R3HCC1	R3H domain and coiled-coil containing 1
224619_at	113201	CASC4	cancer susceptibility candidate 4
203065_s_at	857	CAV1	caveolin 1, caveolae protein, 22kDa
207064_s_at	314	AOC2	amine oxidase, copper containing 2 (retina-specific)
225867_at	114990	VASN	vasorin
232235_at	92126	DSEL	dermatan sulfate epimerase-like
1564786_at	338667	LOC338667	uncharacterized LOC338667
224870_at	57291	DANCR	differentiation antagonizing non-protein coding RNA
212914_at	23492	CBX7	chromobox homolog 7
217988_at	57820	CCNB1IP1	cyclin B1 interacting protein 1, E3 ubiquitin protein ligase
203002_at	51421	AMOTL2	angiomotin like 2
1556121_at	4673	NAP1L1	nucleosome assembly protein 1-like 1
225150_s_at	6242	RTKN	rhotekin
202566_s_at	6840	SVIL	supervillin
226780_s_at	154791	C7orf55	chromosome 7 open reading frame 55
218025_s_at	10455	ECI2	enoyl-CoA delta isomerase 2
217894_at	51133	KCTD3	potassium channel tetramerization domain containing 3
203323_at	858	CAV2	caveolin 2
1553243_at	80760	ITIH5	inter-alpha-trypsin inhibitor heavy chain family, member 5
214279_s_at	57447	NDRG2	NDRG family member 2
213050_at	23242	COBL	cordon-bleu WH2 repeat protein
213977_s_at	25792	CIZ1	CDKN1A interacting zinc finger protein 1
239034_at	203414	CXorf24	chromosome X open reading frame 24
212134_at	23187	PHLDB1	pleckstrin homology-like domain, family B, member 1
227636_at	168451	THAP5	THAP domain containing 5
214464_at	8476	CDC42BPA	CDC42 binding protein kinase alpha (DMPK-like)
203525_s_at	324	APC	adenomatous polyposis coli
239657_x_at	100132074	FOXO6	forkhead box O6
219798_s_at	56257	MEPCE	methylphosphate capping enzyme
204307_at	9895	TECPR2	tectonin beta-propeller repeat containing 2
204797_s_at	2009	EML1	echinoderm microtubule associated protein like 1
242470_at	126272	EID2B	EP300 interacting inhibitor of differentiation 2B
213656_s_at	3831	KLC1	kinesin light chain 1

219379_x_at	140467	ZNF358	zinc finger protein 358
203356_at	23473	CAPN7	calpain 7
203851_at	3489	IGFBP6	insulin-like growth factor binding protein 6
212570_at	23052	ENDOD1	endonuclease domain containing 1
201361_at	79073	TMEM109	transmembrane protein 109
1555865_at	255512	TOLLIP-AS1	TOLLIP antisense RNA 1 (head to head)
225125_at	93380	MMGT1	membrane magnesium transporter 1
202419_at	2531	KDSR	3-ketodihydrosphingosine reductase
212494_at	23371	TENC1	tensin like C1 domain containing phosphatase (tensin 2)
208944_at	7048	TGFBR2	transforming growth factor, beta receptor II (70/80kDa)
212761_at	6934	TCF7L2	transcription factor 7-like 2 (T-cell specific, HMG-box)
213480_at	8674	VAMP4	vesicle-associated membrane protein 4
225820_at	79960	JADE1	jade family PHD finger 1
226985_at	152273	FGD5	FYVE, RhoGEF and PH domain containing 5
200932_s_at	10540	DCTN2	dynactin 2 (p50)
202456_s_at	10444	ZER1	zyg-11 related, cell cycle regulator
215116_s_at	1759	DNM1	dynamin 1
228604_at	199870	FAM76A	family with sequence similarity 76, member A
201431_s_at	1809	DPYSL3	dihydropyrimidinase-like 3
234491_s_at	60485	SAV1	salvador family WW domain containing protein 1
229742_at	145853	C15orf61	chromosome 15 open reading frame 61
232262_at	9487	PIGL	phosphatidylinositol glycan anchor biosynthesis, class L
221565_s_at	51063	CALHM2	calcium homeostasis modulator 2
235753_at	3204	HOXA7	homeobox A7
203733_at	28955	DEXI	Dexi homolog (mouse)
204682_at	4053	LTBP2	latent transforming growth factor beta binding protein 2
219353_at	374354	NHLRC2	NHL repeat containing 2
233496_s_at	1073	CFL2	cofilin 2 (muscle)
213372_at	152559	PAQR3	progestin and adipoQ receptor family member III
219024_at	59338	PLEKHA1	pleckstrin homology domain containing, family A (phosphoinositide binding specific) member 1
227192_at	112476	PRRT2	proline-rich transmembrane protein 2
225835_at	6558	SLC12A2	solute carrier family 12 (sodium/potassium/chloride transporter), member 2
205141_at	283	ANG	angiogenin, ribonuclease, RNase A family, 5
205978_at	9365	KL	klotho
204384_at	2801	GOLGA2	golgin A2
203683_s_at	7423	VEGFB	vascular endothelial growth factor B
201005_at	928	CD9	CD9 molecule
209759_s_at	1632	ECI1	enoyl-CoA delta isomerase 1
235980_at	5290	PIK3CA	phosphatidylinositol-4,5-bisphosphate 3-kinase, catalytic subunit alpha
229741_at	57506	MAVS	mitochondrial antiviral signaling protein
219103_at	55616	ASAP3	ArfGAP with SH3 domain, ankyrin repeat and PH domain 3
214198_s_at	9993	DGCR2	DiGeorge syndrome critical region gene 2
221857_s_at	93643	TJAP1	tight junction associated protein 1 (peripheral)
209333_at	8408	ULK1	unc-51 like autophagy activating kinase 1
201481_s_at	5834	PYGB	phosphorylase, glycogen; brain
219922_s_at	4054	LTBP3	latent transforming growth factor beta binding protein 3
223749_at	114898	C1QTNF2	C1q and tumor necrosis factor related protein 2
232051_at	92922	CCDC102A	coiled-coil domain containing 102A
225383_at	10838	ZNF275	zinc finger protein 275
233002_at	57718	PPP4R4	protein phosphatase 4, regulatory subunit 4
224715_at	89891	WDR34	WD repeat domain 34
1565638_at	5376	PMP22	peripheral myelin protein 22
207068_at	7539	ZFP37	ZFP37 zinc finger protein
203329_at	5797	PTPRM	protein tyrosine phosphatase, receptor type, M
232884_s_at	54753	ZNF853	zinc finger protein 853
229732_at	55552	ZNF823	zinc finger protein 823
224792_at	85456	TNKS1BP1	tankyrase 1 binding protein 1, 182kDa
205042_at	10020	GNE	glucosamine (UDP-N-acetyl)-2-epimerase/N-acetylmannosamine kinase
234107_s_at	92675	DTD1	D-tyrosyl-tRNA deacylase 1
202268_s_at	8883	NAE1	NEDD8 activating enzyme E1 subunit 1

226908_at	121227	LRIG3	leucine-rich repeats and immunoglobulin-like domains 3
205498_at	2690	GHR	growth hormone receptor
208714_at	4723	NDUFV1	NADH dehydrogenase (ubiquinone) flavoprotein 1, 51kDa
206315_at	9244	CRLF1	cytokine receptor-like factor 1
205807_s_at	7286	TUFT1	tuftelin 1
213280_at	23108	RAP1GAP2	RAP1 GTPase activating protein 2
212439_at	9807	IP6K1	inositol hexakisphosphate kinase 1
228214_at	55553	SOX6	SRY (sex determining region Y)-box 6
227115_at	100506870	LOC100506870	uncharacterized LOC100506870
225245_x_at	55766	H2AFJ	H2A histone family, member J
228338_at	120376	COLCA2	colorectal cancer associated 2
202371_at	79921	TCEAL4	transcription elongation factor A (SII)-like 4
201188_s_at	3710	ITPR3	inositol 1,4,5-trisphosphate receptor, type 3
218245_at	25987	TSKU	tsukushi, small leucine rich proteoglycan
201677_at	56941	HMCES	5-hydroxymethylcytosine (hmC) binding, ES cell-specific
219596_at	56906	THAP10	THAP domain containing 10
225044_at	115024	NT5C3B	5'-nucleotidase, cytosolic IIIB
235626_at	57118	CAMK1D	calcium/calmodulin-dependent protein kinase ID
212423_at	219654	ZCCHC24	zinc finger, CCHC domain containing 24
222125_s_at	54681	P4HTM	prolyl 4-hydroxylase, transmembrane (endoplasmic reticulum)
1560475_at	100129455	LOC100129455	uncharacterized LOC100129455
219801_at	80778	ZNF34	zinc finger protein 34
203641_s_at	22837	COBLL1	cordon-bleu WH2 repeat protein-like 1
225421_at	135293	PM20D2	peptidase M20 domain containing 2
213316_at	57608	KIAA1462	KIAA1462
230307_at	100129794	SLC25A21-AS1	SLC25A21 antisense RNA 1
212675_s_at	23177	CEP68	centrosomal protein 68kDa
212162_at	57498	KIDINS220	kinase D-interacting substrate, 220kDa
226771_at	57198	ATP8B2	ATPase, aminophospholipid transporter, class I, type 8B, member 2
204538_x_at	9284	NPIPA1	nuclear pore complex interacting protein family, member A1
202412_s_at	7398	USP1	ubiquitin specific peptidase 1
236359_at	6330	SCN4B	sodium channel, voltage-gated, type IV, beta subunit
205843_x_at	1384	CRAT	carnitine O-acetyltransferase
202365_at	84747	UNC119B	unc-119 homolog B (C. elegans)
219268_at	55224	ETNK2	ethanolamine kinase 2
229480_at	100505881	MAGI2-AS3	MAGI2 antisense RNA 3
209290_s_at	4781	NFIB	nuclear factor I/B
222834_s_at	55970	GNG12	guanine nucleotide binding protein (G protein), gamma 12
241710_at	728819	LOC728819	hCG1645220
218449_at	55325	UFSP2	UFM1-specific peptidase 2
212719_at	23239	PHLPP1	PH domain and leucine rich repeat protein phosphatase 1
204020_at	5813	PURA	purine-rich element binding protein A
226375_at	22853	LMTK2	lemur tyrosine kinase 2
203935_at	90	ACVR1	activin A receptor, type I
202119_s_at	8895	CPNE3	copine III
202734_at	9322	TRIP10	thyroid hormone receptor interactor 10
228538_at	389114	ZNF662	zinc finger protein 662
224975_at	4774	NFIA	nuclear factor I/A
227197_at	26084	ARHGEF26	Rho guanine nucleotide exchange factor (GEF) 26
233919_s_at	22927	HABP4	hyaluronan binding protein 4
206960_at	2846	LPAR4	lysophosphatidic acid receptor 4
225128_at	143888	KDELC2	KDEL (Lys-Asp-Glu-Leu) containing 2
1554242_a_at	1690	COCH	cochlin
222725_s_at	54873	PALMD	palmdelphin
201419_at	8314	BAP1	BRCA1 associated protein-1 (ubiquitin carboxy-terminal hydrolase)
203498_at	10231	RCAN2	regulator of calcineurin 2
226376_at	85451	UNK	unkempt family zinc finger
220176_at	80224	NUBPL	nucleotide binding protein-like
206949_s_at	23623	RUSC1	RUN and SH3 domain containing 1
225627_s_at	57685	CACHD1	cache domain containing 1
205779_at	10266	RAMP2	receptor (G protein-coupled) activity modifying protein 2
225338_at	79699	ZYG11B	zyg-11 family member B, cell cycle regulator

204235_s_at	51454	GULP1	GULP, engulfment adaptor PTB domain containing 1
212892_at	8427	ZNF282	zinc finger protein 282
209074_s_at	11170	FAM107A	family with sequence similarity 107, member A
226409_at	128637	TBC1D20	TBC1 domain family, member 20
218876_at	51673	TPPP3	tubulin polymerization-promoting protein family member 3
216321_s_at	2908	NR3C1	nuclear receptor subfamily 3, group C, member 1 (glucocorticoid receptor)
203650_at	10544	PROCR	protein C receptor, endothelial
213079_at	90121	TSR2	TSR2, 20S rRNA accumulation, homolog (S. cerevisiae)
203211_s_at	8898	MTMR2	myotubularin related protein 2
224690_at	116151	FAM210B	family with sequence similarity 210, member B
215807_s_at	5364	PLXNB1	plexin B1
218212_s_at	4338	MOCS2	molybdenum cofactor synthesis 2
227880_s_at	84548	TMEM185A	transmembrane protein 185A
225689_at	84892	POMGNT2	protein O-linked mannose N-acetylglucosaminyltransferase 2 (beta 1,4-)
214720_x_at	151011	SEPT10	septin 10
218665_at	8322	FZD4	frizzled class receptor 4
232014_at	90075	ZNF30	zinc finger protein 30
205968_at	3790	KCNS3	potassium voltage-gated channel, delayed-rectifier, subfamily S, member 3
235431_s_at	246330	PELI3	pellino E3 ubiquitin protein ligase family member 3
224059_s_at	9253	NUMBL	numb homolog (Drosophila)-like
219011_at	57664	PLEKHA4	pleckstrin homology domain containing, family A (phosphoinositide binding specific) member 4
208504_x_at	56125	PCDHB11	protocadherin beta 11
230277_at	100289187	GS1-259H13.2	transmembrane protein 225-like
226770_at	260425	MAGI3	membrane associated guanylate kinase, WW and PDZ domain containing 3
225543_at	9329	GTF3C4	general transcription factor IIIC, polypeptide 4, 90kDa
205497_at	7728	ZNF175	zinc finger protein 175
242358_at	100506451	RASSF8-AS1	RASSF8 atnisense RNA 1
221950_at	2018	EMX2	empty spiracles homeobox 2
225060_at	84918	LRP11	low density lipoprotein receptor-related protein 11
204504_s_at	8479	HIRIP3	HIRA interacting protein 3
225450_at	154810	AMOTL1	angiomotin like 1
213422_s_at	54587	MXRA8	matrix-remodelling associated 8
203943_at	9371	KIF3B	kinesin family member 3B
218031_s_at	1112	FOXN3	forkhead box N3
226022_at	23328	SASH1	SAM and SH3 domain containing 1
227784_s_at	9382	COG1	component of oligomeric golgi complex 1
212032_s_at	53635	PTOV1	prostate tumor overexpressed 1
234405_s_at	51808	PHAX	phosphorylated adaptor for RNA export
235496_at	646962	HRCT1	histidine rich carboxyl terminus 1
201426_s_at	7431	VIM	vimentin
239846_at	4522	MTHFD1	methylenetetrahydrofolate dehydrogenase (NADP+ dependent) 1, methenyltetrahydrofolate cyclohydrolase, formyltetrahydrofolate synthetase
214766_s_at	25909	AHCTF1	AT hook containing transcription factor 1
209402_s_at	6560	SLC12A4	solute carrier family 12 (potassium/chloride transporter), member 4
214077_x_at	4213	MEIS3P1	Meis homeobox 3 pseudogene 1
221507_at	30000	TNPO2	transportin 2
230063_at	9422	ZNF264	zinc finger protein 264
219688_at	55212	BBS7	Bardet-Biedl syndrome 7
201080_at	8396	PIP4K2B	phosphatidylinositol-5-phosphate 4-kinase, type II, beta
209485_s_at	114876	OSBPL1A	oxysterol binding protein-like 1A
219034_at	54956	PARP16	poly (ADP-ribose) polymerase family, member 16
218225_at	51295	ECSIT	ECSIT signalling integrator
221518_s_at	55031	USP47	ubiquitin specific peptidase 47
229603_at	166379	BBS12	Bardet-Biedl syndrome 12
231729_s_at	828	CAPS	calcyphosine
222138_s_at	64743	WDR13	WD repeat domain 13
224525_s_at	29789	OLA1	Obg-like ATPase 1
229430_at	254778	C8orf46	chromosome 8 open reading frame 46
201018_at	1964	EIF1AX	eukaryotic translation initiation factor 1A, X-linked
209501_at	1039	CDR2	cerebellar degeneration-related protein 2, 62kDa
224860_at	90871	TMEM261	transmembrane protein 261

227239_at	84668	FAM126A	family with sequence similarity 126, member A
229461_x_at	257194	NEGR1	neuronal growth regulator 1
203869_at	64854	USP46	ubiquitin specific peptidase 46
225868_at	91107	TRIM47	tripartite motif containing 47
202796_at	11346	SYNPO	synaptopodin
203812_at	6586	SLIT3	slit homolog 3 (Drosophila)
213339_at	57212	TP73-AS1	TP73 antisense RNA 1
1560371_at	401321	LINC00997	long intergenic non-protein coding RNA 997
203038_at	5796	PTPRK	protein tyrosine phosphatase, receptor type, K
204605_at	10668	CGRRF1	cell growth regulator with ring finger domain 1
227274_at	55333	SYNJ2BP	synaptojanin 2 binding protein
217982_s_at	10933	MORF4L1	mortality factor 4 like 1
206910_x_at	3080	CFHR2	complement factor H-related 2
214829_at	10157	AASS	aminoadipate-semialdehyde synthase
231969_at	56977	STOX2	storkhead box 2
204288_s_at	8470	SORBS2	sorbin and SH3 domain containing 2
206002_at	10149	GPR64	G protein-coupled receptor 64
239147_at	153642	ARSK	arylsulfatase family, member K
203818_s_at	10946	SF3A3	splicing factor 3a, subunit 3, 60kDa
201092_at	5931	RBBP7	retinoblastoma binding protein 7
216840_s_at	3908	LAMA2	laminin, alpha 2
223475_at	83690	CRISPLD1	cysteine-rich secretory protein LCCL domain containing 1
213547_at	23066	CAND2	cullin-associated and neddylation-dissociated 2 (putative)
207191_s_at	3671	ISLR	immunoglobulin superfamily containing leucine-rich repeat
1554703_at	9639	ARHGEF10	Rho guanine nucleotide exchange factor (GEF) 10
217957_at	29105	CFAP20	cilia and flagella associated protein 20
235224_s_at	55832	CAND1	cullin-associated and neddylation-dissociated 1
214764_at	51018	RRP15	ribosomal RNA processing 15 homolog (S. cerevisiae)
212747_at	23294	ANKS1A	ankyrin repeat and sterile alpha motif domain containing 1A
233047_at	90167	FRMD7	FERM domain containing 7
229656_s_at	400954	EML6	echinoderm microtubule associated protein like 6
223967_at	83854	ANGPTL6	angiopoietin-like 6
214620_x_at	5066	PAM	peptidylglycine alpha-amidating monooxygenase
228841_at	90624	LYRM7	LYR motif containing 7
211698_at	23741	EID1	EP300 interacting inhibitor of differentiation 1
219594_at	4815	NINJ2	ninjurin 2
203169_at	9827	RGP1	RGP1 retrograde golgi transport homolog (S. cerevisiae)
208671_at	57515	SERINC1	serine incorporator 1
213030_s_at	5362	PLXNA2	plexin A2
211478_s_at	1803	DPP4	dipeptidyl-peptidase 4
222988_s_at	252839	TMEM9	transmembrane protein 9
226364_at	3092	HIP1	huntingtin interacting protein 1
217902_s_at	8924	HERC2	HECT and RLD domain containing E3 ubiquitin protein ligase 2
223304_at	84255	SLC37A3	solute carrier family 37, member 3
204873_at	5189	PEX1	peroxisomal biogenesis factor 1
203339_at	8604	SLC25A12	solute carrier family 25 (aspartate/glutamate carrier), member 12
220233_at	115290	FBXO17	F-box protein 17
207002_s_at	5325	PLAGL1	pleiomorphic adenoma gene-like 1
218625_at	51299	NRN1	neuritin 1
208871_at	1822	ATN1	atrophin 1
207966_s_at	2734	GLG1	golgi glycoprotein 1
205086_s_at	29781	NCAPH2	non-SMC condensin II complex, subunit H2
241926_s_at	2078	ERG	v-ets avian erythroblastosis virus E26 oncogene homolog
201405_s_at	10980	COPS6	COP9 signalosome subunit 6
227093_at	57602	USP36	ubiquitin specific peptidase 36
217729_s_at	166	AES	amino-terminal enhancer of split
213066_at	9853	RUSC2	RUN and SH3 domain containing 2
206404_at	2254	FGF9	fibroblast growth factor 9
200795_at	8404	SPARCL1	SPARC-like 1 (hevin)
227679_at	79885	HDAC11	histone deacetylase 11
230520_at	51390	AIG1	androgen-induced 1
225698_at	114915	EPB41L4A-AS1	EPB41L4A antisense RNA 1

1568900_a_at	374900	ZNF568	zinc finger protein 568
222101_s_at	8642	DCHS1	dachsous cadherin-related 1
202623_at	55837	EAPP	E2F-associated phosphoprotein
212144_at	25777	SUN2	Sad1 and UNC84 domain containing 2
202261_at	6944	VPS72	vacuolar protein sorting 72 homolog (S. cerevisiae)
235505_s_at	4043	LRPAP1	low density lipoprotein receptor-related protein associated protein 1
204792_s_at	9742	IFT140	intraflagellar transport 140
207808_s_at	5627	PROS1	protein S (alpha)
225574_at	201965	RWDD4	RWD domain containing 4
201173_x_at	10726	NUDC	nudC nuclear distribution protein
230035_at	91653	BOC	BOC cell adhesion associated, oncogene regulated
228239_at	54065	SMIM11	small integral membrane protein 11
212904_at	57470	LRRC47	leucine rich repeat containing 47
214909_s_at	23564	DDAH2	dimethylarginine dimethylaminohydrolase 2
213934_s_at	7571	ZNF23	zinc finger protein 23
228096_at	440574	MINOS1	mitochondrial inner membrane organizing system 1
222462_s_at	23621	BACE1	beta-site APP-cleaving enzyme 1
218634_at	23612	PHLDA3	pleckstrin homology-like domain, family A, member 3
227917_at	100506990	LOC100506990	uncharacterized LOC100506990
1555958_at	55118	CRTAC1	cartilage acidic protein 1
202732_at	11142	PKIG	protein kinase (cAMP-dependent, catalytic) inhibitor gamma
226634_at	399818	METTL10	methyltransferase like 10
226886_at	2673	GFPT1	glutamine--fructose-6-phosphate transaminase 1
228121_at	7042	TGFB2	transforming growth factor, beta 2
203354_s_at	23362	PSD3	pleckstrin and Sec7 domain containing 3
225096_at	55352	COPRS	coordinator of PRMT5, differentiation stimulator
242323_at	81579	PLA2G12A	phospholipase A2, group XIIA
201027_s_at	9669	EIF5B	eukaryotic translation initiation factor 5B
208656_s_at	10983	CCNI	cyclin I
241701_at	57584	ARHGAP21	Rho GTPase activating protein 21
227341_at	222389	BEND7	BEN domain containing 7
228885_at	256691	MAMDC2	MAM domain containing 2
205713_s_at	1311	COMP	cartilage oligomeric matrix protein
203590_at	1783	DYNC1LI2	dynein, cytoplasmic 1, light intermediate chain 2
203319_s_at	7707	ZNF148	zinc finger protein 148
229982_at	79832	QSER1	glutamine and serine rich 1
205802_at	7220	TRPC1	transient receptor potential cation channel, subfamily C, member 1
217870_s_at	51727	CMPK1	cytidine monophosphate (UMP-CMP) kinase 1, cytosolic
205414_s_at	9912	ARHGAP44	Rho GTPase activating protein 44
211348_s_at	8555	CDC14B	cell division cycle 14B
212169_at	11328	FKBP9	FK506 binding protein 9, 63 kDa
223989_s_at	25996	REXO2	RNA exonuclease 2
218502_s_at	7227	TRPS1	trichorhinophalangeal syndrome I
218123_at	56683	C21orf59	chromosome 21 open reading frame 59
238081_at	404201	WDFY3-AS2	WDFY3 antisense RNA 2
1554609_at	100287896	LOC100287896	uncharacterized LOC100287896
218108_at	55148	UBR7	ubiquitin protein ligase E3 component n-recognin 7 (putative)
205259_at	4306	NR3C2	nuclear receptor subfamily 3, group C, member 2
203706_s_at	8324	FZD7	frizzled class receptor 7
219561_at	51226	COPZ2	coatomer protein complex, subunit zeta 2
201599_at	4942	OAT	ornithine aminotransferase
218128_at	4801	NFYB	nuclear transcription factor Y, beta
219152_at	50512	PODXL2	podocalyxin-like 2
223340_at	51062	ATL1	atlastin GTPase 1
203562_at	9638	FEZ1	fasciculation and elongation protein zeta 1 (zygin I)
214721_x_at	23580	CDC42EP4	CDC42 effector protein (Rho GTPase binding) 4
200675_at	975	CD81	CD81 molecule
204742_s_at	23047	PDS5B	PDS5, regulator of cohesion maintenance, homolog B (S. cerevisiae)
202762_at	9475	ROCK2	Rho-associated, coiled-coil containing protein kinase 2
227782_at	201501	ZBTB7C	zinc finger and BTB domain containing 7C
1553994_at	4907	NT5E	5'-nucleotidase, ecto (CD73)
231851_at	55225	RAVER2	ribonucleoprotein, PTB-binding 2

235587_at	202781	PAXIP1-AS1	PAXIP1 antisense RNA 1 (head to head)
223236_at	84081	NSRP1	nuclear speckle splicing regulatory protein 1
223249_at	9069	CLDN12	claudin 12
203950_s_at	1185	CLCN6	chloride channel, voltage-sensitive 6
206101_at	1842	ECM2	extracellular matrix protein 2, female organ and adipocyte specific
204012_s_at	9836	LCMT2	leucine carboxyl methyltransferase 2
225196_s_at	64949	MRPS26	mitochondrial ribosomal protein S26
220316_at	64067	NPAS3	neuronal PAS domain protein 3
224639_at	121665	SPPL3	signal peptide peptidase like 3
44702_at	85360	SYDE1	synapse defective 1, Rho GTPase, homolog 1 (C. elegans)
226907_at	81706	PPP1R14C	protein phosphatase 1, regulatory (inhibitor) subunit 14C
211727_s_at	1353	COX11	cytochrome c oxidase assembly homolog 11 (yeast)
218762_at	64763	ZNF574	zinc finger protein 574
202641_at	403	ARL3	ADP-ribosylation factor-like 3
224415_s_at	84681	HINT2	histidine triad nucleotide binding protein 2
219188_s_at	28992	MACROD1	MACRO domain containing 1
201260_s_at	6856	SYPL1	synaptophysin-like 1
208924_at	26994	RNF11	ring finger protein 11
227378_x_at	84326	C16orf13	chromosome 16 open reading frame 13
228739_at	192668	CYS1	cystin 1
204633_s_at	9252	RPS6KA5	ribosomal protein S6 kinase, 90kDa, polypeptide 5
200764_s_at	1495	CTNNA1	catenin (cadherin-associated protein), alpha 1, 102kDa
213883_s_at	83941	TM2D1	TM2 domain containing 1
204671_s_at	22881	ANKRD6	ankyrin repeat domain 6
227954_at	162073	ITPRIPL2	inositol 1,4,5-trisphosphate receptor interacting protein-like 2
206637_at	9934	P2RY14	purinergic receptor P2Y, G-protein coupled, 14
227701_at	55088	CCDC186	coiled-coil domain containing 186
217899_at	54867	TMEM214	transmembrane protein 214
201082_s_at	1639	DCTN1	dynactin 1
204421_s_at	2247	FGF2	fibroblast growth factor 2 (basic)
1558834_s_at	254268	AKNAD1	AKNA domain containing 1
227298_at	401264	TRAM2-AS1	TRAM2 antisense RNA 1 (head to head)
228535_at	5810	RAD1	RAD1 checkpoint DNA exonuclease
228783_at	11149	BVES	blood vessel epicardial substance
219059_s_at	10894	LYVE1	lymphatic vessel endothelial hyaluronan receptor 1
204457_s_at	2619	GAS1	growth arrest-specific 1
217991_x_at	23648	SSBP3	single stranded DNA binding protein 3
218518_at	51306	FAM13B	family with sequence similarity 13, member B
1568868_at	339761	CYP27C1	cytochrome P450, family 27, subfamily C, polypeptide 1
203071_at	7869	SEMA3B	sema domain, immunoglobulin domain (Ig), short basic domain, secreted, (semaphorin) 3B
202724_s_at	2308	FOXO1	forkhead box O1
236837_x_at	650794	MIPEPP3	mitochondrial intermediate peptidase pseudogene 3
209625_at	5283	PIGH	phosphatidylinositol glycan anchor biosynthesis, class H
203296_s_at	477	ATP1A2	ATPase, Na+/K+ transporting, alpha 2 polypeptide
227279_at	85012	TCEAL3	transcription elongation factor A (SII)-like 3
222519_s_at	55081	IFT57	intraflagellar transport 57
209108_at	7105	TSPAN6	tetraspanin 6
234971_x_at	113026	PLCD3	phospholipase C, delta 3
229127_at	58494	JAM2	junctional adhesion molecule 2
235164_at	219749	ZNF25	zinc finger protein 25
202744_at	6575	SLC20A2	solute carrier family 20 (phosphate transporter), member 2
218509_at	64748	LPPR2	lipid phosphate phosphatase-related protein type 2
209684_at	54453	RIN2	Ras and Rab interactor 2
227248_at	79990	PLEKHH3	pleckstrin homology domain containing, family H (with MyTH4 domain) member 3
219764_at	11211	FZD10	frizzled class receptor 10
203430_at	23593	HEBP2	heme binding protein 2
226829_at	84632	AFAP1L2	actin filament associated protein 1-like 2
203407_at	5493	PPL	periplakin
206243_at	7079	TIMP4	TIMP metallopeptidase inhibitor 4
202500_at	3300	DNAJB2	DnaJ (Hsp40) homolog, subfamily B, member 2

214297_at	1464	CSPG4	chondroitin sulfate proteoglycan 4
225900_at	23233	EXOC6B	exocyst complex component 6B
211325_x_at	171220	DSTNP2	destrin (actin depolymerizing factor) pseudogene 2
218573_at	28986	MAGEH1	melanoma antigen family H, 1
218816_at	55227	LRRC1	leucine rich repeat containing 1
230963_at	196047	EMX2OS	EMX2 opposite strand/antisense RNA
214783_s_at	311	ANXA11	annexin A11
213900_at	9413	FAM189A2	family with sequence similarity 189, member A2
201373_at	5339	PLEC	plectin
217849_s_at	9578	CDC42BPB	CDC42 binding protein kinase beta (DMPK-like)
229116_at	22866	CNKSR2	connector enhancer of kinase suppressor of Ras 2
209211_at	688	KLF5	Kruppel-like factor 5 (intestinal)
222605_at	55758	RCOR3	REST corepressor 3
229088_at	5167	ENPP1	ectonucleotide pyrophosphatase/phosphodiesterase 1
1557137_at	200728	TMEM17	transmembrane protein 17
213075_at	169611	OLFML2A	olfactomedin-like 2A
210874_s_at	24142	NAT6	N-acetyltransferase 6 (GCN5-related)
224830_at	11051	NUDT21	nudix (nucleoside diphosphate linked moiety X)-type motif 21
212061_at	23350	U2SURP	U2 snRNP-associated SURP domain containing
205006_s_at	9397	NMT2	N-myristoyltransferase 2
225953_at	55197	RPRD1A	regulation of nuclear pre-mRNA domain containing 1A
229829_at	147525	LINC00526	long intergenic non-protein coding RNA 526
213739_at	103344931	LOC103344931	uncharacterized LOC103344931
204324_s_at	27333	GOLIM4	golgi integral membrane protein 4
219496_at	65124	SOWAHC	sosondowah ankyrin repeat domain family member C
232874_at	23348	DOCK9	dedicator of cytokinesis 9
225790_at	253827	MSRB3	methionine sulfoxide reductase B3
200659_s_at	5245	PHB	prohibitin
1556427_s_at	221091	LRRN4CL	LRRN4 C-terminal like
211043_s_at	1212	CLTB	clathrin, light chain B
225278_at	5565	PRKAB2	protein kinase, AMP-activated, beta 2 non-catalytic subunit
218071_s_at	23609	MKRN2	makorin ring finger protein 2
219414_at	64084	CLSTN2	calsyntenin 2
227892_at	5563	PRKAA2	protein kinase, AMP-activated, alpha 2 catalytic subunit
224993_at	4298	MLLT1	myeloid/lymphoid or mixed-lineage leukemia (trithorax homolog, Drosophila); translocated to, 1
201161_s_at	8531	YBX3	Y box binding protein 3
201828_x_at	8933	FAM127A	family with sequence similarity 127, member A
224151_s_at	50808	AK3	adenylate kinase 3
202133_at	25937	WWTR1	WW domain containing transcription regulator 1
203515_s_at	10654	PMVK	phosphomevalonate kinase
225315_at	219927	MRPL21	mitochondrial ribosomal protein L21
236717_at	165186	FAM179A	family with sequence similarity 179, member A
201962_s_at	10193	RNF41	ring finger protein 41, E3 ubiquitin protein ligase
202429_s_at	5530	PPP3CA	protein phosphatase 3, catalytic subunit, alpha isozyme
227242_s_at	253738	EBF3	early B-cell factor 3
218970_s_at	51076	CUTC	cutC copper transporter
217721_at	989	SEPT7	septin 7
203776_at	27238	GPKOW	G patch domain and KOW motifs
238028_at	647024	C6orf132	chromosome 6 open reading frame 132
225462_at	85013	TMEM128	transmembrane protein 128
218934_s_at	27129	HSPB7	heat shock 27kDa protein family, member 7 (cardiovascular)
213924_at	2774	GNAL	guanine nucleotide binding protein (G protein), alpha activating activity polypeptide, olfactory type
204917_s_at	4300	MLLT3	myeloid/lymphoid or mixed-lineage leukemia (trithorax homolog, Drosophila); translocated to, 3
212289_at	23253	ANKRD12	ankyrin repeat domain 12
220744_s_at	55764	IFT122	intraflagellar transport 122
226589_at	201931	TMEM192	transmembrane protein 192
230807_at	115948	CCDC151	coiled-coil domain containing 151
221688_s_at	55272	IMP3	IMP3, U3 small nucleolar ribonucleoprotein
225170_at	11091	WDR5	WD repeat domain 5

225103_at	64978	MRPL38	mitochondrial ribosomal protein L38
213526_s_at	55957	LIN37	lin-37 DREAM MuvB core complex component
204045_at	9338	TCEAL1	transcription elongation factor A (SII)-like 1
221922_at	29899	GPSM2	G-protein signaling modulator 2
228676_at	220064	ORAOV1	oral cancer overexpressed 1
219566_at	79156	PLEKHF1	pleckstrin homology domain containing, family F (with FYVE domain) member 1
212713_at	4239	MFAP4	microfibrillar-associated protein 4
215247_at	100288570	LOC100288570	glycosylphosphatidylinositol anchor attachment protein 1 homolog (yeast) pseudogene
225991_at	90407	TMEM41A	transmembrane protein 41A
205883_at	7704	ZBTB16	zinc finger and BTB domain containing 16
209407_s_at	10522	DEAF1	DEAF1 transcription factor
202948_at	3554	IL1R1	interleukin 1 receptor, type I
226586_at	203286	ANKS6	ankyrin repeat and sterile alpha motif domain containing 6
1553228_at	220388	CCDC89	coiled-coil domain containing 89
222918_at	51209	RAB9B	RAB9B, member RAS oncogene family
213383_at	6305	SBF1	SET binding factor 1
225798_at	221895	JAZF1	JAZF zinc finger 1
222623_s_at	51193	ZNF639	zinc finger protein 639
223046_at	54583	EGLN1	egl-9 family hypoxia-inducible factor 1
201915_at	11231	SEC63	SEC63 homolog (S. cerevisiae)
206138_s_at	5298	PI4KB	phosphatidylinositol 4-kinase, catalytic, beta
221512_at	84065	TMEM222	transmembrane protein 222
207618_s_at	617	BCS1L	BC1 (ubiquinol-cytochrome c reductase) synthesis-like
200956_s_at	6749	SSRP1	structure specific recognition protein 1
224881_at	154807	VKORC1L1	vitamin K epoxide reductase complex, subunit 1-like 1
200673_at	9741	LAPTM4A	lysosomal protein transmembrane 4 alpha
225827_at	27161	AGO2	argonaute RISC catalytic component 2
212199_at	114932	MRFAP1L1	Morf4 family associated protein 1-like 1
233936_s_at	79893	GGNBP2	gametogenetin binding protein 2
216396_s_at	9538	EI24	etoposide induced 2.4
225181_at	57492	ARID1B	AT rich interactive domain 1B (SWI1-like)
214156_at	25924	MYRIP	myosin VIIA and Rab interacting protein
212992_at	113146	AHNAK2	AHNAK nucleoprotein 2
206875_s_at	9748	SLK	STE20-like kinase
215427_s_at	23174	ZCCHC14	zinc finger, CCHC domain containing 14
207722_s_at	55643	BTBD2	BTB (POZ) domain containing 2
218604_at	23592	LEMD3	LEM domain containing 3
218922_s_at	79603	CERS4	ceramide synthase 4
220346_at	441024	MTHFD2L	methylenetetrahydrofolate dehydrogenase (NADP+ dependent) 2-like
209046_s_at	11345	GABARAPL2	GABA(A) receptor-associated protein-like 2
226143_at	10743	RAI1	retinoic acid induced 1
226695_at	5396	PRRX1	paired related homeobox 1
201253_s_at	10423	CDIPT	CDP-diacylglycerol--inositol 3-phosphatidyltransferase
1553960_at	90203	SNX21	sorting nexin family member 21
238992_at	11201	POLI	polymerase (DNA directed) iota
226685_at	6645	SNTB2	syntrophin, beta 2 (dystrophin-associated protein A1, 59kDa, basic component 2)
218047_at	114883	OSBPL9	oxysterol binding protein-like 9
205076_s_at	10903	MTMR11	myotubularin related protein 11
203734_at	55810	FOXJ2	forkhead box J2
221902_at	387509	GPR153	G protein-coupled receptor 153
223028_s_at	51429	SNX9	sorting nexin 9
205352_at	5274	SERPINI1	serpin peptidase inhibitor, clade I (neuroserpin), member 1
218034_at	51024	FIS1	fission 1 (mitochondrial outer membrane) homolog (S. cerevisiae)
218546_at	79762	C1orf115	chromosome 1 open reading frame 115
210558_at	1109	AKR1C4	aldo-keto reductase family 1, member C4
222737_s_at	29117	BRD7	bromodomain containing 7
220948_s_at	476	ATP1A1	ATPase, Na+/K+ transporting, alpha 1 polypeptide
228972_at	100306951	PITPNA-AS1	PITPNA antisense RNA 1
235414_at	163087	ZNF383	zinc finger protein 383
206027_at	6274	S100A3	S100 calcium binding protein A3

225422_at	246184	CDC26	cell division cycle 26
218477_at	28978	TMEM14A	transmembrane protein 14A
225785_at	221035	REEP3	receptor accessory protein 3
32836_at	10554	AGPAT1	1-acylglycerol-3-phosphate O-acyltransferase 1
227188_at	59271	EVA1C	eva-1 homolog C (C. elegans)
221270_s_at	81890	QTRT1	queuine tRNA-ribosyltransferase 1
214736_s_at	118	ADD1	adducin 1 (alpha)
203089_s_at	27429	HTRA2	HtrA serine peptidase 2
241384_x_at	256356	GK5	glycerol kinase 5 (putative)
218903_s_at	79035	NABP2	nucleic acid binding protein 2
222243_s_at	10766	TOB2	transducer of ERBB2, 2
228065_at	283149	BCL9L	B-cell CLL/lymphoma 9-like
202176_at	2071	ERCC3	excision repair cross-complementation group 3
209311_at	599	BCL2L2	BCL2-like 2
205364_at	8309	ACOX2	acyl-CoA oxidase 2, branched chain
209216_at	11152	WDR45	WD repeat domain 45
210094_s_at	56288	PARD3	par-3 family cell polarity regulator
204752_x_at	10038	PARP2	poly (ADP-ribose) polymerase 2
239468_at	283078	MKX	mohawk homeobox
48106_at	55652	SLC48A1	solute carrier family 48 (heme transporter), member 1
218484_at	56901	NDUFA4L2	NADH dehydrogenase (ubiquinone) 1 alpha subcomplex, 4-like 2
202071_at	6385	SDC4	syndecan 4
202260_s_at	6812	STXBP1	syntaxin binding protein 1
222043_at	1191	CLU	clusterin
229909_at	283358	B4GALNT3	beta-1,4-N-acetyl-galactosaminyl transferase 3
213040_s_at	23467	NPTXR	neuronal pentraxin receptor
225576_at	116254	GINM1	glycoprotein integral membrane 1
226092_at	64398	MPP5	membrane protein, palmitoylated 5 (MAGUK p55 subfamily member 5)
217940_s_at	55739	CARKD	carbohydrate kinase domain containing
201580_s_at	56255	TMX4	thioredoxin-related transmembrane protein 4
212510_at	23171	GPD1L	glycerol-3-phosphate dehydrogenase 1-like
209387_s_at	4071	TM4SF1	transmembrane 4 L six family member 1
205579_at	3269	HRH1	histamine receptor H1
1552572_a_at	145282	MIPOL1	mirror-image polydactyly 1
207469_s_at	8544	PIR	pirin (iron-binding nuclear protein)
230250_at	5787	PTPRB	protein tyrosine phosphatase, receptor type, B
217897_at	53826	FXYD6	FXYD domain containing ion transport regulator 6
219132_at	57161	PELI2	pellino E3 ubiquitin protein ligase family member 2
225950_at	142891	SAMD8	sterile alpha motif domain containing 8
203845_at	8850	KAT2B	K(lysine) acetyltransferase 2B
213455_at	92689	FAM114A1	family with sequence similarity 114, member A1
212212_s_at	26173	INTS1	integrator complex subunit 1
227899_at	5212	VIT	vitrin
207855_s_at	23155	CLCC1	chloride channel CLIC-like 1
225138_at	54764	ZRANB1	zinc finger, RAN-binding domain containing 1
230472_at	79192	IRX1	iroquois homeobox 1
223616_at	65251	ZNF649	zinc finger protein 649
221583_s_at	3778	KCNMA1	potassium large conductance calcium-activated channel, subfamily M, alpha member 1
203410_at	10947	AP3M2	adaptor-related protein complex 3, mu 2 subunit
226932_at	8082	SSPN	sarcospan
216080_s_at	3995	FADS3	fatty acid desaturase 3
237833_s_at	9627	SNCAIP	synuclein, alpha interacting protein
202353_s_at	5718	PSMD12	proteasome (prosome, macropain) 26S subunit, non-ATPase, 12
202928_s_at	5252	PHF1	PHD finger protein 1
225016_at	147495	APCDD1	adenomatosis polyposis coli down-regulated 1
214258_x_at	10524	KAT5	K(lysine) acetyltransferase 5
226839_at	126382	NR2C2AP	nuclear receptor 2C2-associated protein
238458_at	286097	MICU3	mitochondrial calcium uptake family, member 3
213249_at	23194	FBXL7	F-box and leucine-rich repeat protein 7
201380_at	10491	CRTAP	cartilage associated protein
223129_x_at	29116	MYLIP	myosin regulatory light chain interacting protein

228429_x_at	64147	KIF9	kinesin family member 9
204071_s_at	10210	TOPORS	topoisomerase I binding, arginine/serine-rich, E3 ubiquitin protein ligase
224523_s_at	84319	CMSS1	cms1 ribosomal small subunit homolog (yeast)
221878_at	388969	C2orf68	chromosome 2 open reading frame 68
218966_at	55930	MYO5C	myosin VC
219354_at	55295	KLHL26	kelch-like family member 26
218253_s_at	1939	EIF2D	eukaryotic translation initiation factor 2D
208549_x_at	5757	PTMA	prothymosin, alpha
226571_s_at	5802	PTPRS	protein tyrosine phosphatase, receptor type, S
226716_at	57479	PRR12	proline rich 12
200986_at	710	SERPING1	serpin peptidase inhibitor, clade G (C1 inhibitor), member 1
235155_at	56898	BDH2	3-hydroxybutyrate dehydrogenase, type 2
200816_s_at	5048	PAFAH1B1	platelet-activating factor acetylhydrolase 1b, regulatory subunit 1 (45kDa)
226996_at	253558	LCLAT1	lysocardiolipin acyltransferase 1
209736_at	9580	SOX13	SRY (sex determining region Y)-box 13
236600_at	23111	SPG20	spastic paraplegia 20 (Troyer syndrome)
201928_at	8502	PKP4	plakophilin 4
228602_at	6444	SGCD	sarcoglycan, delta (35kDa dystrophin-associated glycoprotein)
226769_at	387758	FIBIN	fin bud initiation factor homolog (zebrafish)
232099_at	57717	PCDHB16	protocadherin beta 16
200621_at	1465	CSRP1	cysteine and glycine-rich protein 1
223095_at	83742	MARVELD1	MARVEL domain containing 1
200699_at	11014	KDELR2	KDEL (Lys-Asp-Glu-Leu) endoplasmic reticulum protein retention receptor 2
206756_at	56548	CHST7	carbohydrate (N-acetylglucosamine 6-O) sulfotransferase 7
202342_s_at	23321	TRIM2	tripartite motif containing 2
208920_at	6717	SRI	sorcin
201788_at	11325	DDX42	DEAD (Asp-Glu-Ala-Asp) box helicase 42
201375_s_at	5516	PPP2CB	protein phosphatase 2, catalytic subunit, beta isozyme
211110_s_at	367	AR	androgen receptor
224484_s_at	84312	BRMS1L	breast cancer metastasis-suppressor 1-like
214318_s_at	10129	FRY	furry homolog (Drosophila)
238477_at	10749	KIF1C	kinesin family member 1C
223250_at	55975	KLHL7	kelch-like family member 7
220617_s_at	55205	ZNF532	zinc finger protein 532
215460_x_at	23774	BRD1	bromodomain containing 1
225334_at	119032	C10orf32	chromosome 10 open reading frame 32
1558692_at	112770	C1orf85	chromosome 1 open reading frame 85
202930_s_at	8803	SUCLA2	succinate-CoA ligase, ADP-forming, beta subunit
213362_at	5789	PTPRD	protein tyrosine phosphatase, receptor type, D
238647_at	122525	C14orf28	chromosome 14 open reading frame 28
204271_s_at	1910	EDNRB	endothelin receptor type B
235377_at	90523	MLIP	muscular LMNA-interacting protein
204573_at	54677	CROT	carnitine O-octanoyltransferase
212237_at	171023	ASXL1	additional sex combs like transcriptional regulator 1
1552733_at	122773	KLHDC1	kelch domain containing 1
217814_at	57003	CCDC47	coiled-coil domain containing 47
200066_at	3550	IK	IK cytokine, down-regulator of HLA II
243650_at	130271	PLEKHH2	pleckstrin homology domain containing, family H (with MyTH4 domain) member 2
206188_at	9831	ZNF623	zinc finger protein 623
228584_at	6443	SGCB	sarcoglycan, beta (43kDa dystrophin-associated glycoprotein)
226479_at	89890	KBTBD6	kelch repeat and BTB (POZ) domain containing 6
223230_at	84950	PRPF38A	pre-mRNA processing factor 38A
226683_at	112574	SNX18	sorting nexin 18
228138_at	221785	ZSCAN25	zinc finger and SCAN domain containing 25
202561_at	8658	TNKS	tankyrase, TRF1-interacting ankyrin-related ADP-ribose polymerase
227731_at	7555	CNBP	CCHC-type zinc finger, nucleic acid binding protein
238077_at	200845	KCTD6	potassium channel tetramerization domain containing 6
201960_s_at	23077	MYCBP2	MYC binding protein 2, E3 ubiquitin protein ligase
212736_at	89927	C16orf45	chromosome 16 open reading frame 45
224943_at	55727	BTBD7	BTB (POZ) domain containing 7

205251_at	8864	PER2	period circadian clock 2
210625_s_at	8165	AKAP1	A kinase (PRKA) anchor protein 1
218147_s_at	55830	GLT8D1	glycosyltransferase 8 domain containing 1
204201_s_at	5783	PTPN13	protein tyrosine phosphatase, non-receptor type 13 (APO-1/CD95 (Fas)-associated phosphatase)
213103_at	90627	STARD13	StAR-related lipid transfer (START) domain containing 13
231899_at	85463	ZC3H12C	zinc finger CCCH-type containing 12C
226838_at	130502	TTC32	tetratricopeptide repeat domain 32
205606_at	4040	LRP6	low density lipoprotein receptor-related protein 6
228345_at	53344	CHIC1	cysteine-rich hydrophobic domain 1
44669_at	644096	SDHAF1	succinate dehydrogenase complex assembly factor 1
209022_at	10735	STAG2	stromal antigen 2
227582_at	126823	KLHDC9	kelch domain containing 9
226793_at	283267	LINC00294	long intergenic non-protein coding RNA 294
202962_at	23303	KIF13B	kinesin family member 13B
209503_s_at	5705	PSMC5	proteasome (prosome, macropain) 26S subunit, ATPase, 5
218492_s_at	80764	THAP7	THAP domain containing 7
202421_at	3321	IGSF3	immunoglobulin superfamily, member 3
209150_s_at	10548	TM9SF1	transmembrane 9 superfamily member 1
213181_s_at	4337	MOCS1	molybdenum cofactor synthesis 1
226297_at	10114	HIPK3	homeodomain interacting protein kinase 3
227081_at	7802	DNALI1	dynein, axonemal, light intermediate chain 1
32502_at	81544	GDPD5	glycerophosphodiester phosphodiesterase domain containing 5
221600_s_at	28971	AAMDC	adipogenesis associated, Mth938 domain containing
225127_at	57583	TMEM181	transmembrane protein 181
213794_s_at	25983	NGDN	neuroguidin, EIF4E binding protein
201534_s_at	5412	UBL3	ubiquitin-like 3
218110_at	56949	XAB2	XPA binding protein 2
209258_s_at	9126	SMC3	structural maintenance of chromosomes 3
225840_at	7008	TEF	thyrotrophic embryonic factor
201366_at	310	ANXA7	annexin A7
244293_at	7381	UQCRB	ubiquinol-cytochrome c reductase binding protein
222116_s_at	125058	TBC1D16	TBC1 domain family, member 16
218116_at	51759	C9orf78	chromosome 9 open reading frame 78
226314_at	113189	CHST14	carbohydrate (N-acetylgalactosamine 4-0) sulfotransferase 14
223412_at	84078	KBTBD7	kelch repeat and BTB (POZ) domain containing 7
214778_at	1954	MEGF8	multiple EGF-like-domains 8
220643_s_at	55179	FAIM	Fas apoptotic inhibitory molecule
223315_at	59277	NTN4	netrin 4
221534_at	83638	C11orf68	chromosome 11 open reading frame 68
203321_s_at	22850	ADNP2	ADNP homeobox 2
226181_at	51175	TUBE1	tubulin, epsilon 1
229889_at	388341	LRRC75A	leucine rich repeat containing 75A
212301_at	23168	RTF1	Rtf1, Paf1/RNA polymerase II complex component, homolog (S. cerevisiae)
204824_at	2021	ENDOG	endonuclease G
212666_at	57154	SMURF1	SMAD specific E3 ubiquitin protein ligase 1
203303_at	6990	DYNLT3	dynein, light chain, Tctex-type 3
225662_at	51776	ZAK	sterile alpha motif and leucine zipper containing kinase AZK
208955_at	1854	DUT	deoxyuridine triphosphatase
212068_s_at	84726	PRRC2B	proline-rich coiled-coil 2B
207727_s_at	4595	MUTYH	mutY homolog
228310_at	55740	ENAH	enabled homolog (Drosophila)
219729_at	51450	PRRX2	paired related homeobox 2
208651_x_at	100133941	CD24	CD24 molecule
214035_x_at	399491	LOC399491	polycystic kidney disease 1 (autosomal dominant) pseudogene
237291_at	344405	PRORSD1P	prolyl-tRNA synthetase associated domain containing 1, pseudogene
209350_s_at	2874	GPS2	G protein pathway suppressor 2
222650_s_at	56731	SLC2A4RG	SLC2A4 regulator
219939_s_at	7812	CSDE1	cold shock domain containing E1, RNA-binding
203009_at	4059	BCAM	basal cell adhesion molecule (Lutheran blood group)
201611_s_at	23463	ICMT	isoprenylcysteine carboxyl methyltransferase
231786_at	3209	HOXA13	homeobox A13

224772_at	89796	NAV1	neuron navigator 1
204498_s_at	115	ADCY9	adenylate cyclase 9
212586_at	831	CAST	calpastatin
218868_at	57180	ACTR3B	ARP3 actin-related protein 3 homolog B (yeast)
1564736_a_at	100506742	CASP12	caspase 12 (gene/pseudogene)
224280_s_at	56181	MTFR1L	mitochondrial fission regulator 1-like
225936_at	163126	EID2	EP300 interacting inhibitor of differentiation 2
203257_s_at	79096	C11orf49	chromosome 11 open reading frame 49
205187_at	4090	SMAD5	SMAD family member 5
227236_at	10100	TSPAN2	tetraspanin 2
213010_at	112464	PRKCDBP	protein kinase C, delta binding protein
213174_at	23508	TTC9	tetratricopeptide repeat domain 9
220939_s_at	54878	DPP8	dipeptidyl-peptidase 8
225529_at	116983	ACAP3	ArfGAP with coiled-coil, ankyrin repeat and PH domains 3
202513_s_at	5528	PPP2R5D	protein phosphatase 2, regulatory subunit B', delta
213394_at	23005	MAPKBP1	mitogen-activated protein kinase binding protein 1
203894_at	27175	TUBG2	tubulin, gamma 2
211564_s_at	8572	PDLIM4	PDZ and LIM domain 4
230782_at	6652	SORD	sorbitol dehydrogenase
201474_s_at	3675	ITGA3	integrin, alpha 3 (antigen CD49C, alpha 3 subunit of VLA-3 receptor)
222212_s_at	29956	CERS2	ceramide synthase 2
1554470_s_at	29068	ZBTB44	zinc finger and BTB domain containing 44
221958_s_at	79971	WLS	wntless Wnt ligand secretion mediator
226800_at	84455	EFCAB7	EF-hand calcium binding domain 7
219175_s_at	54946	SLC41A3	solute carrier family 41, member 3
227767_at	1456	CSNK1G3	casein kinase 1, gamma 3
204749_at	4675	NAP1L3	nucleosome assembly protein 1-like 3
223297_at	83607	AMMECR1L	AMMECR1-like
205740_s_at	79171	RBM42	RNA binding motif protein 42
219230_at	55273	TMEM100	transmembrane protein 100
203221_at	7088	TLE1	transducin-like enhancer of split 1 (E(sp1) homolog, Drosophila)
207069_s_at	4091	SMAD6	SMAD family member 6
201827_at	6603	SMARCD2	SWI/SNF related, matrix associated, actin dependent regulator of chromatin, subfamily d, member 2
226994_at	10294	DNAJA2	DnaJ (Hsp40) homolog, subfamily A, member 2
202815_s_at	10614	HEXIM1	hexamethylene bis-acetamide inducible 1
204576_s_at	23059	CLUAP1	clusterin associated protein 1
202163_s_at	9337	CNOT8	CCR4-NOT transcription complex, subunit 8
206621_s_at	7458	EIF4H	eukaryotic translation initiation factor 4H
223470_at	93183	PIGM	phosphatidylinositol glycan anchor biosynthesis, class M
226534_at	4254	KITLG	KIT ligand
209242_at	5178	PEG3	paternally expressed 3
239466_at	344595	LINC00883	long intergenic non-protein coding RNA 883
244767_at	118924	FRA10AC1	fragile site, folic acid type, rare, fra(10)(q23.3) or fra(10)(q24.2) candidate 1
223515_s_at	51805	COQ3	coenzyme Q3 methyltransferase
217286_s_at	57446	NDRG3	NDRG family member 3
224920_x_at	91663	MYADM	myeloid-associated differentiation marker
205074_at	6584	SLC22A5	solute carrier family 22 (organic cation/carnitine transporter), member 5
222421_at	7328	UBE2H	ubiquitin-conjugating enzyme E2H
204512_at	3096	HIVEP1	human immunodeficiency virus type I enhancer binding protein 1
218411_s_at	51562	MBIP	MAP3K12 binding inhibitory protein 1
218496_at	246243	RNASEH1	ribonuclease H1
207624_s_at	6103	RPGR	retinitis pigmentosa GTPase regulator
1556633_at	284677	C1orf204	chromosome 1 open reading frame 204
231736_x_at	4257	MGST1	microsomal glutathione S-transferase 1
200907_s_at	23022	PALLD	palladin, cytoskeletal associated protein
205225_at	2099	ESR1	estrogen receptor 1
201384_s_at	4077	NBR1	neighbor of BRCA1 gene 1
209633_at	5523	PPP2R3A	protein phosphatase 2, regulatory subunit B'', alpha
1564746_at	133308	SLC9B2	solute carrier family 9, subfamily B (NHA2, cation proton antiporter 2), member 2
213285_at	161291	TMEM30B	transmembrane protein 30B

227692_at	2770	GNAI1	guanine nucleotide binding protein (G protein), alpha inhibiting activity polypeptide 1
218813_s_at	56904	SH3GLB2	SH3-domain GRB2-like endophilin B2
1552289_a_at	148113	CILP2	cartilage intermediate layer protein 2
217025_s_at	1627	DBN1	drebrin 1
221235_s_at	9392	TGFBRAP1	transforming growth factor, beta receptor associated protein 1
210473_s_at	166647	GPR125	G protein-coupled receptor 125
212329_at	22937	SCAP	SREBF chaperone
1558345_a_at	439911	LOC439911	uncharacterized LOC439911
238017_at	195814	SDR16C5	short chain dehydrogenase/reductase family 16C, member 5
226010_at	79085	SLC25A23	solute carrier family 25 (mitochondrial carrier; phosphate carrier), member 23
235005_at	115752	DIS3L	DIS3 like exosome 3'-5' exoribonuclease
203886_s_at	2199	FBLN2	fibulin 2
200007_at	6727	SRP14	signal recognition particle 14kDa (homologous Alu RNA binding protein)
219377_at	64762	GAREM	GRB2 associated, regulator of MAPK1
226754_at	90987	ZNF251	zinc finger protein 251
227170_at	100131017	ZNF316	zinc finger protein 316
226331_at	56987	BBX	bobby sox homolog (Drosophila)
219878_s_at	51621	KLF13	Kruppel-like factor 13
201996_s_at	23013	SPEN	spen family transcriptional repressor
209362_at	9412	MED21	mediator complex subunit 21
212417_at	9522	SCAMP1	secretory carrier membrane protein 1
219548_at	7564	ZNF16	zinc finger protein 16
223139_s_at	170506	DHX36	DEAH (Asp-Glu-Ala-His) box polypeptide 36
202781_s_at	51763	INPP5K	inositol polyphosphate-5-phosphatase K
218601_at	55665	URGCP	upregulator of cell proliferation
229910_at	126669	SHE	Src homology 2 domain containing E
230029_x_at	130507	UBR3	ubiquitin protein ligase E3 component n-recognin 3 (putative)
219443_at	55617	TASP1	taspase, threonine aspartase, 1
209711_at	23169	SLC35D1	solute carrier family 35 (UDP-GlcA/UDP-GalNAc transporter), member D1
225175_s_at	57153	SLC44A2	solute carrier family 44 (choline transporter), member 2
205218_at	10621	POLR3F	polymerase (RNA) III (DNA directed) polypeptide F, 39 kDa
233532_x_at	51098	IFT52	intraflagellar transport 52
227331_at	283337	ZNF740	zinc finger protein 740
204589_at	9891	NUAK1	NUAK family, SNF1-like kinase, 1
210117_at	6674	SPAG1	sperm associated antigen 1
226801_s_at	64853	AIDA	axin interactor, dorsalization associated
209139_s_at	8575	PRKRA	protein kinase, interferon-inducible double stranded RNA dependent activator
228525_at	4037	LRP3	low density lipoprotein receptor-related protein 3
203576_at	587	BCAT2	branched chain amino-acid transaminase 2, mitochondrial
203556_at	22882	ZHX2	zinc fingers and homeoboxes 2
209550_at	4692	NDN	necdin, melanoma antigen (MAGE) family member
221135_s_at	28990	ASTE1	asteroid homolog 1 (Drosophila)
209651_at	7041	TGFB1I1	transforming growth factor beta 1 induced transcript 1
221846_s_at	57513	CASKIN2	CASK interacting protein 2
236268_at	9117	SEC22C	SEC22 vesicle trafficking protein homolog C (S. cerevisiae)
1552296_at	266675	BEST4	bestrophin 4
212977_at	57007	ACKR3	atypical chemokine receptor 3
212983_at	3265	HRAS	Harvey rat sarcoma viral oncogene homolog
224836_at	58476	TP53INP2	tumor protein p53 inducible nuclear protein 2
209621_s_at	27295	PDLIM3	PDZ and LIM domain 3
227850_x_at	148170	CDC42EP5	CDC42 effector protein (Rho GTPase binding) 5
221568_s_at	55327	LIN7C	lin-7 homolog C (C. elegans)
229377_at	79774	GRTP1	growth hormone regulated TBC protein 1
221589_s_at	4329	ALDH6A1	aldehyde dehydrogenase 6 family, member A1
213823_at	3207	HOXA11	homeobox A11
220911_s_at	57523	NYNRIN	NYN domain and retroviral integrase containing
202034_x_at	9821	RB1CC1	RB1-inducible coiled-coil 1
225020_at	153090	DAB2IP	DAB2 interacting protein
204388_s_at	4128	MAOA	monoamine oxidase A

202587_s_at	203	AK1	adenylate kinase 1
221208_s_at	79684	MSANTD2	Myb/SANT-like DNA-binding domain containing 2
215749_s_at	64689	GORASP1	golgi reassembly stacking protein 1, 65kDa
221984_s_at	79137	FAM134A	family with sequence similarity 134, member A
218364_at	9209	LRRFIP2	leucine rich repeat (in FLII) interacting protein 2
219372_at	28981	IFT81	intraflagellar transport 81
243042_at	374986	FAM73A	family with sequence similarity 73, member A
219413_at	79777	ACBD4	acyl-CoA binding domain containing 4
219487_at	79738	BBS10	Bardet-Biedl syndrome 10
208940_at	22929	SEPHS1	selenophosphate synthetase 1
204784_s_at	4291	MLF1	myeloid leukemia factor 1
52651_at	1296	COL8A2	collagen, type VIII, alpha 2
202116_at	5977	DPF2	D4, zinc and double PHD fingers family 2
226449_at	153241	CEP120	centrosomal protein 120kDa
211518_s_at	652	BMP4	bone morphogenetic protein 4
244289_at	134466	ZNF300P1	zinc finger protein 300 pseudogene 1 (functional)
213064_at	79882	ZC3H14	zinc finger CCCH-type containing 14
223253_at	54749	EPDR1	ependymin related 1
224658_x_at	55690	PACS1	phosphofurin acidic cluster sorting protein 1
241198_s_at	85016	C11orf70	chromosome 11 open reading frame 70
213242_x_at	283638	CEP170B	centrosomal protein 170B
220276_at	79785	RERGL	RERG/RAS-like
202468_s_at	8727	CTNNAL1	catenin (cadherin-associated protein), alpha-like 1
219421_at	23548	TTC33	tetratricopeptide repeat domain 33
203543_s_at	687	KLF9	Kruppel-like factor 9
208093_s_at	81565	NDEL1	nudE neurodevelopment protein 1-like 1
222103_at	466	ATF1	activating transcription factor 1
229156_s_at	100505483	PRKAG2-AS1	PRKAG2 antisense RNA 1
212104_s_at	23543	RBFOX2	RNA binding protein, fox-1 homolog (C. elegans) 2
203999_at	6857	SYT1	synaptotagmin I
206464_at	660	BMX	BMX non-receptor tyrosine kinase
212409_s_at	26092	TOR1AIP1	torsin A interacting protein 1
1554520_at	283861	LOC283861	uncharacterized LOC283861
218804_at	55107	ANO1	anoctamin 1, calcium activated chloride channel
218756_s_at	79154	DHRS11	dehydrogenase/reductase (SDR family) member 11
221858_at	23232	TBC1D12	TBC1 domain family, member 12
213398_s_at	56948	SDR39U1	short chain dehydrogenase/reductase family 39U, member 1
225886_at	1655	DDX5	DEAD (Asp-Glu-Ala-Asp) box helicase 5
208831_x_at	6830	SUPT6H	suppressor of Ty 6 homolog (S. cerevisiae)
231726_at	56122	PCDHB14	protocadherin beta 14
202017_at	2052	EPHX1	epoxide hydrolase 1, microsomal (xenobiotic)
223141_at	83549	UCK1	uridine-cytidine kinase 1
234338_s_at	92999	ZBTB47	zinc finger and BTB domain containing 47
212697_at	162427	FAM134C	family with sequence similarity 134, member C
229685_at	100134937	LOC100134937	uncharacterized LOC100134937
203592_s_at	10272	FSTL3	follistatin-like 3 (secreted glycoprotein)
209390_at	7248	TSC1	tuberous sclerosis 1
244885_at	64641	EBF2	early B-cell factor 2
212508_at	64112	MOAP1	modulator of apoptosis 1
221643_s_at	473	RERE	arginine-glutamic acid dipeptide (RE) repeats
203172_at	9513	FXR2	fragile X mental retardation, autosomal homolog 2
226303_at	5239	PGM5	phosphoglucomutase 5
225747_at	93058	COQ10A	coenzyme Q10 homolog A (S. cerevisiae)
214782_at	2017	CTTN	cortactin
227047_x_at	57659	ZBTB4	zinc finger and BTB domain containing 4
227111_at	403341	ZBTB34	zinc finger and BTB domain containing 34
213202_at	9739	SETD1A	SET domain containing 1A
1557113_at	283588	LOC283588	uncharacterized LOC283588
1553696_s_at	148266	ZNF569	zinc finger protein 569
202825_at	291	SLC25A4	solute carrier family 25 (mitochondrial carrier; adenine nucleotide translocator), member 4
226750_at	55132	LARP1B	La ribonucleoprotein domain family, member 1B

206377_at	2295	FOXF2	forkhead box F2
215321_at	154661	RUNDC3B	RUN domain containing 3B
223611_s_at	84708	LNX1	ligand of numb-protein X 1, E3 ubiquitin protein ligase
203446_s_at	4952	OCRL	oculocerebrorenal syndrome of Lowe
203855_at	22911	WDR47	WD repeat domain 47
225075_at	81572	PDRG1	p53 and DNA-damage regulated 1
223190_s_at	55904	KMT2E	lysine (K)-specific methyltransferase 2E
226324_s_at	26160	IFT172	intraflagellar transport 172
218675_at	51310	SLC22A17	solute carrier family 22, member 17
208973_at	79033	ERI3	ERI1 exoribonuclease family member 3
238058_at	150381	PRR34-AS1	PRR34 antisense RNA 1
228144_at	91975	ZNF300	zinc finger protein 300
210418_s_at	3420	IDH3B	isocitrate dehydrogenase 3 (NAD+) beta
227873_at	79770	TXNDC15	thioredoxin domain containing 15
208723_at	8237	USP11	ubiquitin specific peptidase 11
209702_at	79068	FTO	fat mass and obesity associated
224808_s_at	51608	GET4	golgi to ER traffic protein 4 homolog (S. cerevisiae)
204862_s_at	4832	NME3	NME/NM23 nucleoside diphosphate kinase 3
227325_at	255783	INAFM1	InaF-motif containing 1
232071_at	9801	MRPL19	mitochondrial ribosomal protein L19
204568_at	22863	ATG14	autophagy related 14
202492_at	79065	ATG9A	autophagy related 9A
201241_at	1653	DDX1	DEAD (Asp-Glu-Ala-Asp) box helicase 1
225677_at	55973	BCAP29	B-cell receptor-associated protein 29
212955_s_at	5438	POLR2I	polymerase (RNA) II (DNA directed) polypeptide I, 14.5kDa
202932_at	7525	YES1	YES proto-oncogene 1, Src family tyrosine kinase
231844_at	157247	MGC27345	uncharacterized protein MGC27345
230438_at	6913	TBX15	T-box 15
213661_at	25891	PAMR1	peptidase domain containing associated with muscle regeneration 1
226352_at	133746	JMY	junction mediating and regulatory protein, p53 cofactor
204835_at	5422	POLA1	polymerase (DNA directed), alpha 1, catalytic subunit
224707_at	84418	CYSTM1	cysteine-rich transmembrane module containing 1
225949_at	340371	NRBP2	nuclear receptor binding protein 2
227894_at	197335	WDR90	WD repeat domain 90
227951_s_at	147965	FAM98C	family with sequence similarity 98, member C
201023_at	6879	TAF7	TAF7 RNA polymerase II, TATA box binding protein (TBP)-associated factor, 55kDa
228523_at	340719	NANOS1	nanos homolog 1 (Drosophila)
229090_at	220930	ZEB1-AS1	ZEB1 antisense RNA 1
220353_at	55199	FAM86C1	family with sequence similarity 86, member C1
209174_s_at	54870	QRICH1	glutamine-rich 1
209178_at	9785	DHX38	DEAH (Asp-Glu-Ala-His) box polypeptide 38
229512_at	54954	FAM120C	family with sequence similarity 120C
227264_at	7189	TRAF6	TNF receptor-associated factor 6, E3 ubiquitin protein ligase
221213_s_at	54816	ZNF280D	zinc finger protein 280D
225731_at	57182	ANKRD50	ankyrin repeat domain 50
203488_at	22859	LPHN1	latrophilin 1
225469_at	144363	LYRM5	LYR motif containing 5
223269_at	84265	POLR3GL	polymerase (RNA) III (DNA directed) polypeptide G (32kD)-like
224873_s_at	64432	MRPS25	mitochondrial ribosomal protein S25
212349_at	23509	POFUT1	protein O-fucosyltransferase 1
219330_at	81839	VANGL1	VANGL planar cell polarity protein 1
209105_at	8648	NCOA1	nuclear receptor coactivator 1
226989_at	285704	RGMB	repulsive guidance molecule family member b
200694_s_at	57062	DDX24	DEAD (Asp-Glu-Ala-Asp) box helicase 24
218648_at	64784	CRTC3	CREB regulated transcription coactivator 3
223152_at	54776	PPP1R12C	protein phosphatase 1, regulatory subunit 12C
213147_at	3206	HOXA10	homeobox A10
205341_at	30846	EHD2	EH-domain containing 2
222481_at	26515	TIMM10B	translocase of inner mitochondrial membrane 10 homolog B (yeast)
205508_at	6324	SCN1B	sodium channel, voltage-gated, type I, beta subunit
203907_s_at	9922	IQSEC1	IQ motif and Sec7 domain 1

222477_s_at	51768	TM7SF3	transmembrane 7 superfamily member 3
222819_at	56474	CTPS2	CTP synthase 2
204433_s_at	9825	SPATA2	spermatogenesis associated 2
228855_at	283927	NUDT7	nudix (nucleoside diphosphate linked moiety X)-type motif 7
204519_s_at	51090	PLLP	plasmolipin
228201_at	200894	ARL13B	ADP-ribosylation factor-like 13B
201757_at	4725	NDUFS5	NADH dehydrogenase (ubiquinone) Fe-S protein 5, 15kDa (NADH-coenzyme Q reductase)
228594_at	133686	NADK2	NAD kinase 2, mitochondrial
201348_at	2878	GPX3	glutathione peroxidase 3 (plasma)
226604_at	160418	TMTC3	transmembrane and tetratricopeptide repeat containing 3
225052_at	94107	TMEM203	transmembrane protein 203
241607_at	730102	LOC730102	quinone oxidoreductase-like protein 2 pseudogene
218024_at	51660	MPC1	mitochondrial pyruvate carrier 1
206941_x_at	9723	SEMA3E	sema domain, immunoglobulin domain (Ig), short basic domain, secreted, (semaphorin) 3E
203069_at	9900	SV2A	synaptic vesicle glycoprotein 2A
210999_s_at	2887	GRB10	growth factor receptor-bound protein 10
202442_at	1176	AP3S1	adaptor-related protein complex 3, sigma 1 subunit
213145_at	144699	FBXL14	F-box and leucine-rich repeat protein 14
230434_at	493911	PHOSPHO2	phosphatase, orphan 2
235264_at	29915	HCFC2	host cell factor C2
203162_s_at	10300	KATNB1	katanin p80 (WD repeat containing) subunit B 1
218712_at	54955	C1orf109	chromosome 1 open reading frame 109
207292_s_at	5598	MAPK7	mitogen-activated protein kinase 7
233326_at	339829	CCDC39	coiled-coil domain containing 39
226842_at	84961	FBXL20	F-box and leucine-rich repeat protein 20
214709_s_at	3895	KTN1	kinectin 1 (kinesin receptor)
213615_at	10162	LPCAT3	lysophosphatidylcholine acyltransferase 3
207034_s_at	2736	GLI2	GLI family zinc finger 2
224702_at	153339	TMEM167A	transmembrane protein 167A
211829_s_at	2852	GPER1	G protein-coupled estrogen receptor 1
202443_x_at	4853	NOTCH2	notch 2
221127_s_at	27122	DKK3	dickkopf WNT signaling pathway inhibitor 3
203657_s_at	8722	CTSF	cathepsin F
212702_s_at	23299	BICD2	bicaudal D homolog 2 (Drosophila)
213660_s_at	8940	TOP3B	topoisomerase (DNA) III beta
224217_s_at	11124	FAF1	Fas (TNFRSF6) associated factor 1
217731_s_at	9445	ITM2B	integral membrane protein 2B
213512_at	122616	C14orf79	chromosome 14 open reading frame 79
223474_at	64207	IRF2BPL	interferon regulatory factor 2 binding protein-like
223370_at	65977	PLEKHA3	pleckstrin homology domain containing, family A (phosphoinositide binding specific) member 3
223231_at	83940	TATDN1	TatD DNase domain containing 1
226180_at	134430	WDR36	WD repeat domain 36
202387_at	573	BAG1	BCL2-associated athanogene
201897_s_at	1163	CKS1B	CDC28 protein kinase regulatory subunit 1B
205728_at	10178	TENM1	teneurin transmembrane protein 1
220111_s_at	57101	ANO2	anoctamin 2, calcium activated chloride channel
235158_at	84928	TMEM209	transmembrane protein 209
223535_at	83594	NUDT12	nudix (nucleoside diphosphate linked moiety X)-type motif 12
219778_at	23414	ZFPM2	zinc finger protein, FOG family member 2
205775_at	26240	FAM50B	family with sequence similarity 50, member B
227031_at	23161	SNX13	sorting nexin 13
205273_s_at	10531	PITRM1	pitrilysin metallopeptidase 1
210198_s_at	5354	PLP1	proteolipid protein 1
41113_at	26048	ZNF500	zinc finger protein 500
212968_at	5986	RFNG	RFNG O-fucosylpeptide 3-beta-N-acetylglucosaminyltransferase
229696_at	2235	FECH	ferrochelatase
206683_at	7718	ZNF165	zinc finger protein 165
225640_at	100506710	EBLN3	endogenous Bornavirus-like nucleoprotein 3
204793_at	9737	GPRASP1	G protein-coupled receptor associated sorting protein 1

1554250_s_at	375593	TRIM73	tripartite motif containing 73
242600_at	257019	FRMD3	FERM domain containing 3
202554_s_at	2947	GSTM3	glutathione S-transferase mu 3 (brain)
221246_x_at	7145	TNS1	tensin 1
208015_at	4086	SMAD1	SMAD family member 1
217380_s_at	100505933	ADD3-AS1	ADD3 antisense RNA 1
222495_at	56900	TMEM167B	transmembrane protein 167B
201270_x_at	23386	NUDCD3	NudC domain containing 3
201136_at	5355	PLP2	proteolipid protein 2 (colonic epithelium-enriched)
233952_s_at	49854	ZBTB21	zinc finger and BTB domain containing 21
202273_at	5159	PDGFRB	platelet-derived growth factor receptor, beta polypeptide
222672_at	57128	LYRM4	LYR motif containing 4
201560_at	25932	CLIC4	chloride intracellular channel 4
227207_x_at	7760	ZNF213	zinc finger protein 213
218878_s_at	23411	SIRT1	sirtuin 1
209068_at	9987	HNRNPDL	heterogeneous nuclear ribonucleoprotein D-like
218124_at	54884	RETSAT	retinol saturase (all-trans-retinol 13,14-reductase)
209674_at	1407	CRY1	cryptochrome circadian clock 1
238868_at	55075	UACA	uveal autoantigen with coiled-coil domains and ankyrin repeats
1557943_at	1267	CNP	2',3'-cyclic nucleotide 3' phosphodiesterase
226545_at	135228	CD109	CD109 molecule
228555_at	817	CAMK2D	calcium/calmodulin-dependent protein kinase II delta
227722_at	6228	RPS23	ribosomal protein S23
227980_at	79692	ZNF322	zinc finger protein 322
210150_s_at	3911	LAMA5	laminin, alpha 5
212703_at	83660	TLN2	talin 2
225718_at	80856	KIAA1715	KIAA1715
200850_s_at	10768	AHCYL1	adenosylhomocysteinase-like 1
228802_at	348093	RBPMS2	RNA binding protein with multiple splicing 2
219013_at	63917	GALNT11	polypeptide N-acetylgalactosaminyltransferase 11
219072_at	9274	BCL7C	B-cell CLL/lymphoma 7C
223084_s_at	23582	CCNDBP1	cyclin D-type binding-protein 1
205627_at	978	CDA	cytidine deaminase
218446_s_at	51030	TVP23B	trans-golgi network vesicle protein 23 homolog B (S. cerevisiae)
232129_s_at	84445	LZTS2	leucine zipper, putative tumor suppressor 2
1558280_s_at	9411	ARHGAP29	Rho GTPase activating protein 29
225560_at	29954	POMT2	protein-O-mannosyltransferase 2
220159_at	79963	ABCA11P	ATP-binding cassette, sub-family A (ABC1), member 11, pseudogene
219067_s_at	54780	NSMCE4A	non-SMC element 4 homolog A (S. cerevisiae)
203456_at	11230	PRAF2	PRA1 domain family, member 2
202798_at	10427	SEC24B	SEC24 family member B
212470_at	9043	SPAG9	sperm associated antigen 9
225351_at	404636	FAM45A	family with sequence similarity 45, member A
236456_at	84867	PTPN5	protein tyrosine phosphatase, non-receptor type 5 (striatum-enriched)
237654_at	145376	PPP1R36	protein phosphatase 1, regulatory subunit 36
201369_s_at	678	ZFP36L2	ZFP36 ring finger protein-like 2
201738_at	10289	EIF1B	eukaryotic translation initiation factor 1B
200761_s_at	10550	ARL6IP5	ADP-ribosylation factor-like 6 interacting protein 5
218646_at	54969	C4orf27	chromosome 4 open reading frame 27
205382_s_at	1675	CFD	complement factor D (adipsin)
224562_at	10163	WASF2	WAS protein family, member 2
227967_at	114791	TUBGCP5	tubulin, gamma complex associated protein 5
220327_at	389136	VGLL3	vestigial-like family member 3
231029_at	2153	F5	coagulation factor V (proaccelerin, labile factor)
224891_at	2309	FOXO3	forkhead box O3
202850_at	5825	ABCD3	ATP-binding cassette, sub-family D (ALD), member 3
226302_at	5205	ATP8B1	ATPase, aminophospholipid transporter, class I, type 8B, member 1
1554085_at	317781	DDX51	DEAD (Asp-Glu-Ala-Asp) box polypeptide 51
236166_at	285147	LOC285147	uncharacterized LOC285147
224886_at	339123	JMJD8	jumonji domain containing 8
232305_at	54511	HMGCLL1	3-hydroxymethyl-3-methylglutaryl-CoA lyase-like 1
202919_at	25843	MOB4	MOB family member 4, phocein

236620_at	55183	RIF1	replication timing regulatory factor 1
225381_at	399959	MIR100HG	mir-100-let-7a-2 cluster host gene (non-protein coding)
211930_at	220988	HNRNPA3	heterogeneous nuclear ribonucleoprotein A3
215481_s_at	5830	PEX5	peroxisomal biogenesis factor 5
231738_at	56129	PCDHB7	protocadherin beta 7
219483_s_at	64840	PORCN	porcupine homolog (Drosophila)
227288_at	285672	SREK1IP1	SREK1-interacting protein 1
224947_at	79102	RNF26	ring finger protein 26
202271_at	23219	FBXO28	F-box protein 28
202092_s_at	23568	ARL2BP	ADP-ribosylation factor-like 2 binding protein
225335_at	84838	ZNF496	zinc finger protein 496

Supplementary Table 4

Lymphoid Panel

Entrez Gene	Symbol
973	CD79A
80342	TRAF3IP3
50615	IL21R
9840	KIAA0748
11262	SP140
50619	DEF6
23231	SEL1L3
83416	FCRL5
3561	IL2RG
931	MS4A1
64098	PARVG
221188	GPR114
3932	LCK
80008	TMEM156
114836	SLAMF6
3738	KCNA3
11040	PIM2
923	CD6
9938	ARHGAP25
4063	LY9
916	CD3E
914	CD2
26279	PLA2G2D
399	RHOH
147138	TMC8
79961	DENND2D
151888	BTLA
79958	DENND1C
81793	TLR10
100505746	LOC100505746
91523	FAM113B
57823	SLAMF7
55423	SIRPG
4064	CD180
374403	TBC1D10C
54440	SASH3
84433	CARD11
11322	TMC6
53347	UBASH3A
5133	PDCD1
84941	HSH2D
201633	TIGIT
9834	KIAA0125
11184	MAP4K1
3112	HLA-DOB
6693	SPN
81030	ZBP1
6689	SPIB
5450	POU2AF1

Myeloid Panel

Entrez Gene	Symbol
54443	ANLN
3687	ITGAX
114548	NLRP3
126014	OSCAR
84034	EMILIN2
3576	IL8
4688	NCF2
5329	PLAUR
8843	HCAR3
1230	CCR1
2921	CXCL3
2203	FBP1
30817	EMR2
23601	CLEC5A
9582	APOBEC3B
6696	SPP1
55357	TBC1D2
2710	GK
29992	PILRA
942	CD86
728	C5AR1
6364	CCL20
55092	TMEM51
58191	CXCL16
64127	NOD2
53831	GPR84

Fibroid Panel

Entrez Gene	Symbol
11328	FKBP9
10810	WASF3
57498	KIDINS220
9627	SNCAIP
8555	CDC14B
23259	DDHD2
84255	SLC37A3
200734	SPRED2
121227	LRIG3
84632	AFAP1L2
57088	PLSCR4
4952	OCRL
23348	DOCK9
858	CAV2
23270	TSPYL4
10129	FRY
79188	TMEM43
256356	GK5
26224	FBXL3
9639	ARHGEF10
56256	SERTAD4
8853	ASAP2
10186	LHFP
7289	TULP3
90	ACVR1
10194	TSHZ1
51439	FAM8A1
57515	SERINC1
260425	MAGI3
57161	PELI2
342371	ATXN1L
3096	HIVEP1
7220	TRPC1
25959	KANK2
91404	SESTD1
55970	GNG12
51421	AMOTL2
8613	PPAP2B
151011	SEPT10
5325	PLAGL1
54682	MANSC1
23328	SASH1
79901	CYBRD1
10427	SEC24B
2059	EPS8
5311	PKD2
79633	FAT4
5066	PAM
8476	CDC42BPA
5358	PLS3

Supplementary Table 4 continued

Inflammation Panel

Entrez Gene	Symbol	Entrez Gene	Symbol
115650	TNFRSF13C	4940	OAS3
7049	TGFBR3	4982	TNFRSF11B
4312	MMP1	3553	IL1B
4599	MX1	7481	WNT11
7494	XBP1	439921	MXRA7
11009	IL24	5154	PDGFA
7078	TIMP3	55801	IL26
640	BLK	959	CD40LG
2331	FMOD	4318	MMP9
929	CD14	6097	RORC
3489	IGFBP6	8792	TNFRSF11A
3543	IGLL1	4322	MMP13
7852	CXCR4	9671	WSCD2
59067	IL21	3512	IGJ
50616	IL22	3429	IFI27
7124	TNFA	3440	IFNA2
5175	PECAM1	53342	IL17D
1311	COMP	11096	ADAMTS5
9235	IL32	27122	DKK3
695	BTK	10563	CXCL13
27177	IL36B	50604	IL20
8600	TNFSF11	930	CD19
3598	IL13RA2	79037	PVRIG
29851	ICOS	4939	OAS2
7076	TIMP1	2919	CXCL1
3439	IFNA1	5803	PTPRZ1
3383	ICAM1	4319	MMP10
57379	AICDA	9241	NOG
6280	S100A9	8483	CILP
6382	SDC1	26525	IL36RN
2247	FGF2	5079	PAX5
3458	IFNG	3624	INHBA
6279	S100A8	3569	IL6
26585	GREM1	2254	FGF9
9510	ADAMTS1	51561	IL23A
29949	IL19	3456	IFNB1
1508	CTSB	60	ACTB
91543	RSAD2		
4049	LTA		
1435	CSF1		
4938	OAS1		
3662	IRF4		
1437	CSF2		
3557	IL1RN		
952	CD38		
6363	CCL19		
1440	CSF3		
4314	MMP3		
4050	LTB		
608	TNFRSF17		
