# Supplementary material for: Synovial cellular and molecular signatures stratify clinical response to csDMARD therapy and predict radiographic progression in early rheumatoid arthritis patients
Source: Ann Rheum Dis. 2019 Mar 16;78(6):761–72. doi: 10.1136/annrheumdis-2018-214539 (PMC6579551; doi:10.1136/annrheumdis-2018-214539)
Supplement: Supplementary data [file annrheumdis-2018-214539supp001.pdf]

Cellular and Molecular Signatures in the Disease Tissue of Early Rheumatoid Arthritis Stratify Clinical Response to csDMARD-Therapy and Predict Radiographic Progression

Frances Humby[1],* Myles Lewis[1],* Nandhini Ramamoorthi[2], Jason Hackney[3], Michael Barnes[1], Michele Bombardieri[1], Francesca Setiadi[2], Stephen Kelly[1], Fabiola Bene[1], Maria di Cicco[1], Sudeh Riahi[1], Vidalba Rocher-Ros[1], Nora Ng[1], Ilias Lazorou[1], Rebecca E. Hands[1], Desiree van der Heijde[4], Robert Landewé[5], Annette van der Helm-van Mil[4], Alberto Cauli[6], Iain B. McInnes[7], Christopher D. Buckley[8], Ernest Choy[9], Peter Taylor[10], Michael J. Townsend[2] & Costantino Pitzalis[1]

[1]Centre for Experimental Medicine and Rheumatology, William Harvey Research Institute, Barts and The London School of Medicine and Dentistry, Queen Mary University of London, Charterhouse Square, London EC1M 6BQ, UK.
Departments of [2]Biomarker Discovery OMNI, [3]Bioinformatics and Computational Biology, Genentech Research and Early Development, South San Francisco, California 94080 USA
[4]Department of Rheumatology, Leiden University Medical Center, The Netherlands
[5]Department of Clinical Immunology & Rheumatology, Amsterdam Rheumatology & Immunology Center, Amsterdam, The Netherlands
[6]Rheumatology Unit, Department of Medical Sciences, Policlinico of the University of Cagliari, Cagliari, Italy
[7]Institute of Infection, Immunity and Inflammation, University of Glasgow, Glasgow G12 8TA, UK
[8]Rheumatology Research Group, Institute of Inflammation and Ageing (IIA), University of Birmingham, Birmingham B15 2WB, UK
[9]Institute of Infection and Immunity, Cardiff University School of Medicine, Cardiff CF14 4XN, UK
[10]Nuffield Department of Orthopaedics, Rheumatology and Musculoskeletal Sciences and the Kennedy Institute of Rheumatology, University of Oxford, Oxford, UK

FH, ML, NR and JH contributed equally to the study

Correspondence: Professor Costantino Pitzalis – c.pitzalis@qmul.ac.uk
Dr. Michael J. Townsend – townsem1@gene.com

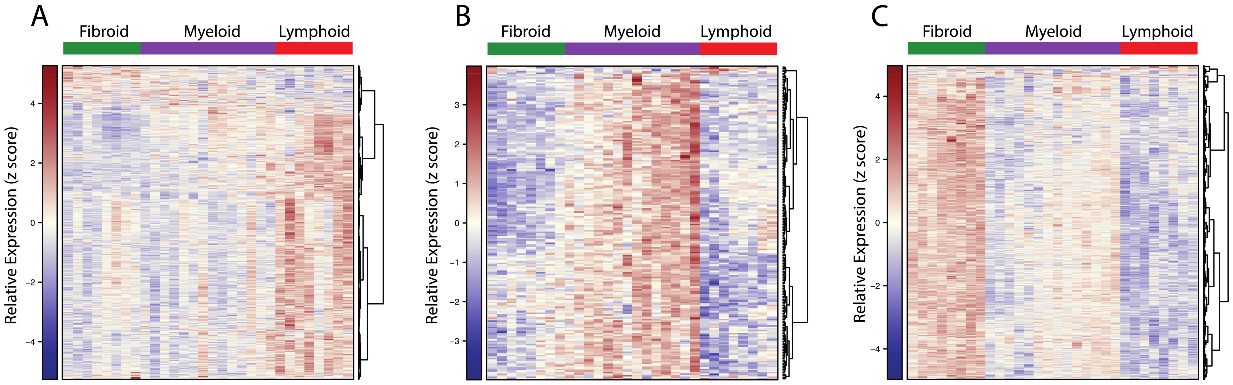

Supplementary Figure 1

Heatmaps of Michigan cohort microarray data. Normalized log2 expression data were normalized per probe to give a mean of 0, and standard deviation of 1 for each gene. Normalized data were clustered by row and column using Euclidean distance and Ward's linkage. Samples are colored according to pathotype assignment (green = Fibroid; purple = Myeloid; red = Lymphoid). **A** Genes significantly different (higher or lower) in lymphoid samples compared to myeloid and fibroid samples. **B** Genes significantly different (higher or lower) in myeloid samples compared to lymphoid and fibroid samples. **C** Genes significantly different (higher or lower) in fibroid samples compared to myeloid and lymphoid samples. Genes were considered significant differentially expressed if they had an adjusted p-value < 0.01. In total 2749 lymphoid-associated, 202 myeloid associated and 1524-fibroid associated genes were identified.

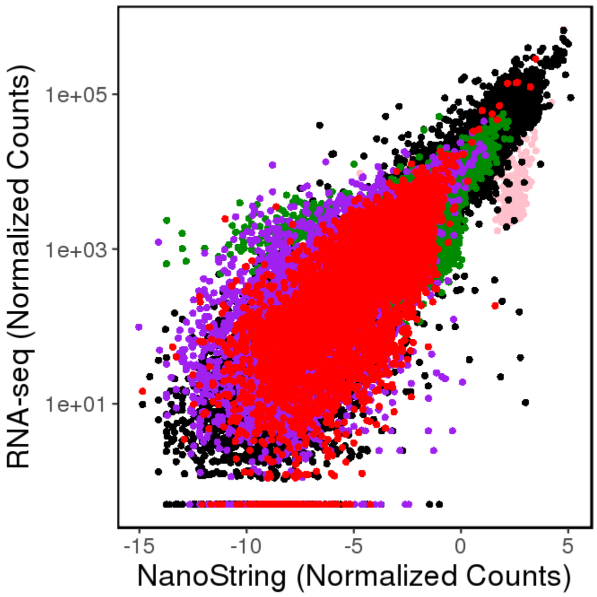

Supplementary Figure 2

Plot of concordance between the NanoString and RNA-sequencing gene expression in the PEAC cohort data. The overall Spearman rho=0.85 between the two measurements. Individual patients are colored based on gene pathotype assignments (red = lymphoid, purple = myeloid, green = fibroid, back = additional biology).

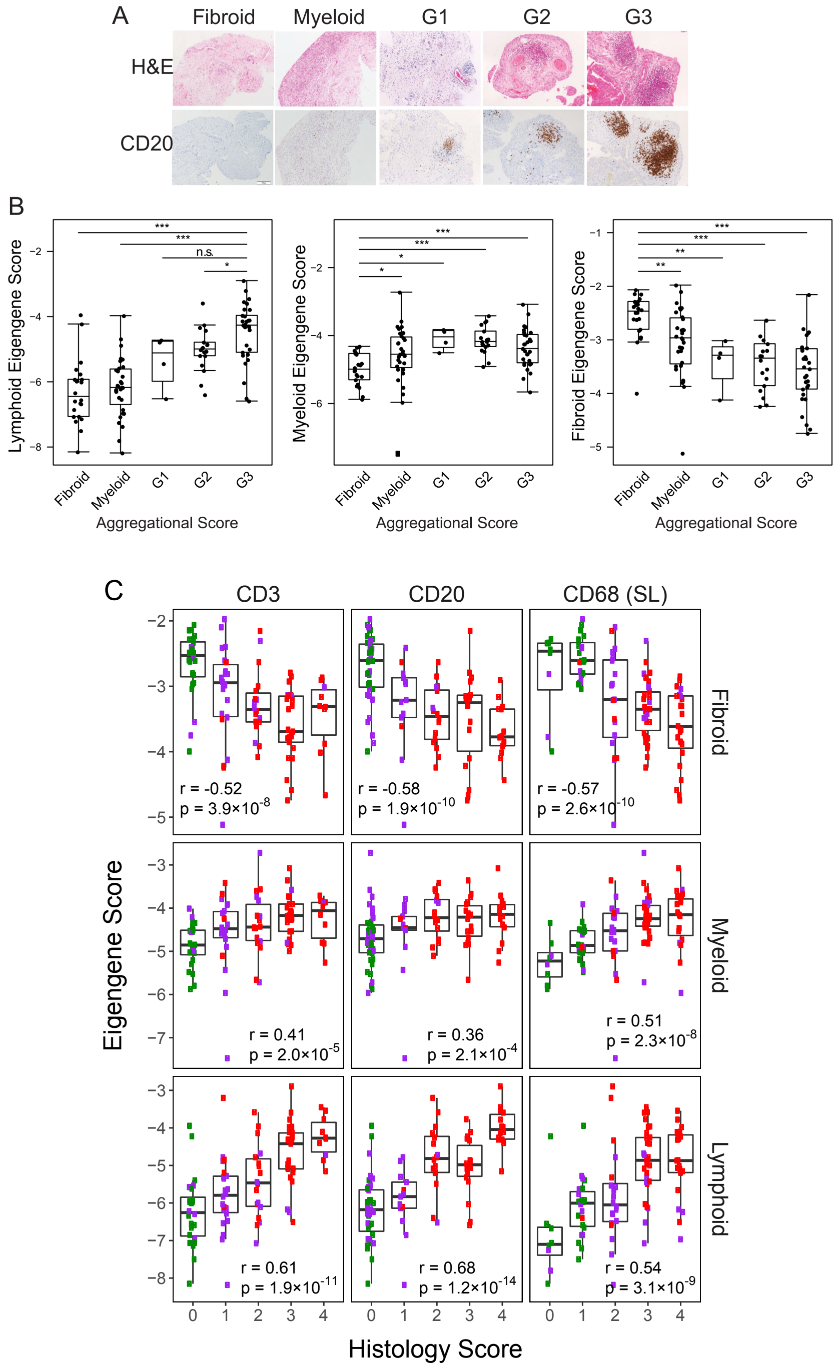

Supplementary Figure 3

A. Representative images of pauciimmune-fibroid, myeloid and grade 1-3 synovial lymphoid aggregates stained with H&E (top) or for CD20 (bottom) at 10x magnification. **B.** Lymphoid, myeloid and pauciimmune-fibroid eigengene scores plotted against synovial aggregational scores (G1, G2, G3 = grade 1, 2 and 3 aggregates respectively). Stars represent statistical significance as determined by linear regression across groups: *=p<0.05, **=p<0.01, ***=p<0.001, n.s. = not significant. **C.** Eigengene scores plotted against histology markers for CD3, CD20 and CD68 (SL). Statistical significance determined by linear regression.

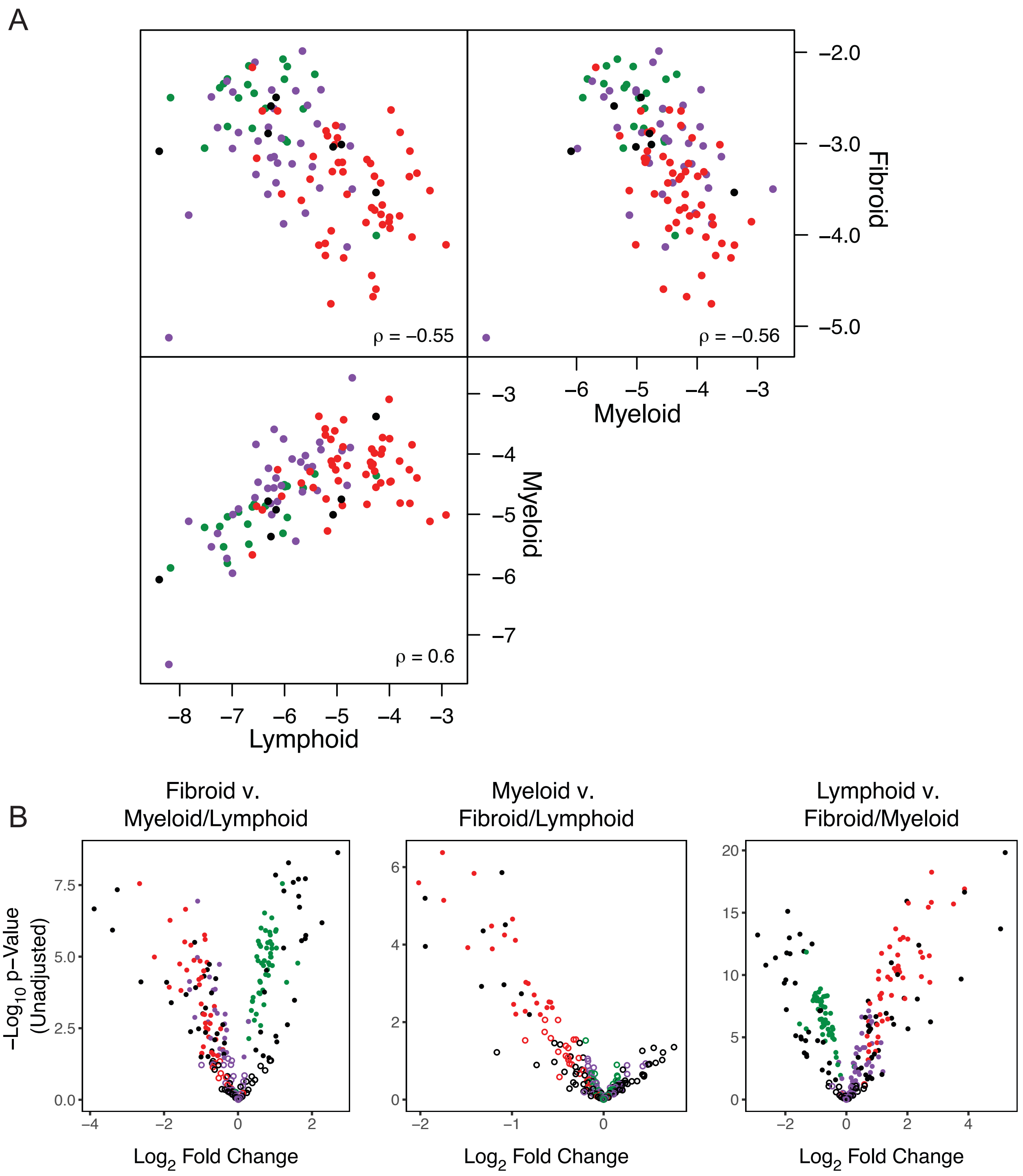

Supplementary Figure 4

A. Pairs plots comparing eigengene scores. Eigengene values for baseline samples are plotting against each other, with samples colored according to IHC-determined pathotype. **B.** Volcano

plots from comparisons between pathotypes. Each pathotype was compared to the other two using linear regression. The log2 fold change and –log10 p-values for these comparisons are shown, with genes colored according to the pathotype to which they were initially assigned, with RA biology-associated genes colored black. Genes that were significant at a Benjamini-Hochberg adjusted p-value < 0.01 are shown as filled dots, while those not meeting this cutoff are shown as open circles.

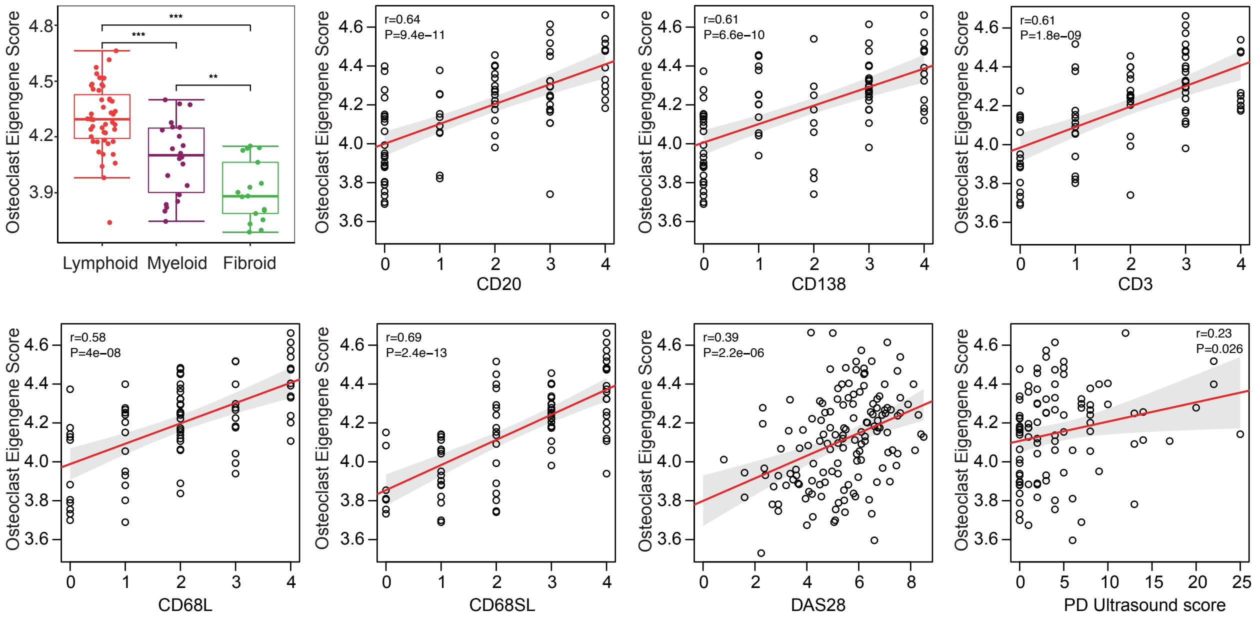

Supplementary Figure 5

Osteoclast genes are elevated in the lymphoid synovial subset. Osteoclast Eigengene scores were calculated for pre-treatment synovial tissue samples are described in materials and methods. Eigengene scores versus synovial pathotypes are presented (stars represent statistical significance as determined by linear regression across groups: **=p<0.01, ***=p<0.001). Correlations (denoted using Spearman rank correlation coefficient) of osteoclast score versus synovial histopathology, DAS28 and Power Doppler Ultrasound score are also presented.

A

	Backward (Clinical only)	LASSO (Clinical + Genes)
Apparent AUC[1]	0.85	0.93
Mean AUC of 500 bootstrap samples[2]	0.91	0.98
Mean AUC of 500 tests in original sample[3]	0.81	0.93
Estimate of optimism[4]	0.1	0.05
Optimism-adjusted AUC[5]	0.75	0.88

B

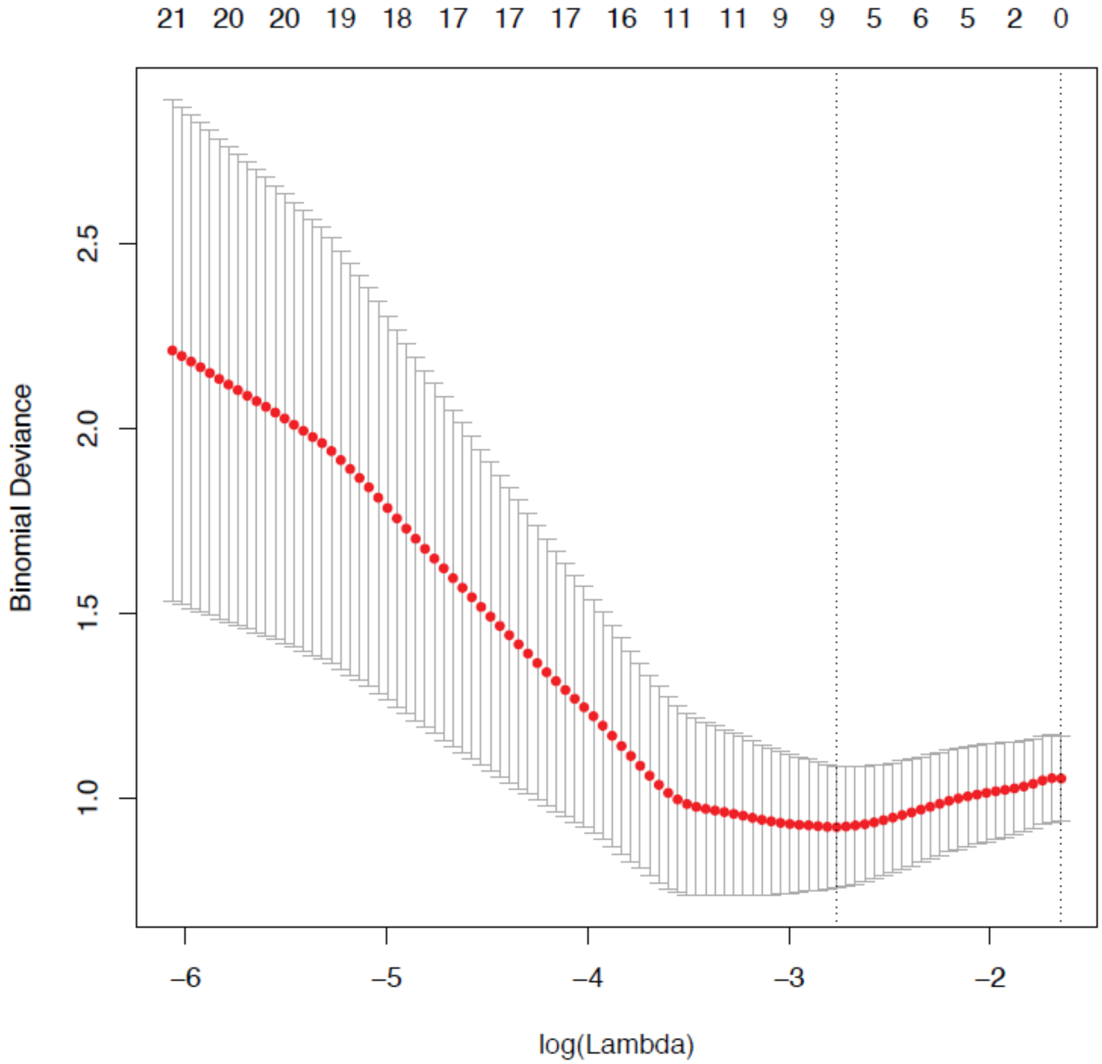

Supplementary Figure 6

A. Evaluation of the performance of the predictive models and estimation of optimism (over-fitting). [1]Performance estimated from the model that was used to develop the prediction model. [2]Performance estimated by developing and validating the model on each of the 500 bootstrap samples. [3]Performance estimated by developing the model on each of the 500 bootstrap samples and validating the model on original sample. [4]Difference between 'Average AUC of 500 bootstrap samples' and 'Average AUC of 500 tests in original sample'. [5]Optimism-adjusted AUC obtained by subtracting 'Average optimism' from 'Apparent AUC'.

B. Lambda training curve from the final glmnet fitted model. The red dots represent mean binomial deviance using 10-fold cross-validation. The error bars represent standard error of binomial deviance. The vertical dotted lines indicate minimum binomial deviance (λ_{min}) and a more regularized model for which the binomial deviance error is within one standard error of the minimum binomial deviance (λ_{1se}). λ_{min} was selected, corresponding to nine non-zero coefficients in the final model.
